# A meta-review demonstrates improved reporting quality of qualitative reviews following the publication of COREQ- and ENTREQ-checklists, regardless of modest uptake

**DOI:** 10.1186/s12874-021-01363-1

**Published:** 2021-09-12

**Authors:** Y. de Jong, E. M. van der Willik, J. Milders, C. G. N. Voorend, Rachael L. Morton, F. W. Dekker, Y. Meuleman, M. van Diepen

**Affiliations:** 1 Department of Clinical Epidemiology, Leiden University Medical Center, Leiden, the Netherlands; 2 Department of Internal Medicine, Leiden University Medical Center, Leiden, the Netherlands; 3 NHMRC Clinical Trials Centre, Faculty of Medicine and Health, The University of Sydney, Sydney, Australia

**Keywords:** Methodology, Appraisal, Qualitative research, Meta-review, Systematic review, COREQ, ENTREQ, Impact study, Uptake

## Abstract

**Background:**

Reviews of qualitative studies allow for deeper understanding of concepts and findings beyond the single qualitative studies. Concerns on study reporting quality led to the publication of the COREQ-guidelines for qualitative studies in 2007, followed by the ENTREQ-guidelines for qualitative reviews in 2012. The aim of this meta-review is to: 1) investigate the uptake of the COREQ- and ENTREQ- checklists in qualitative reviews; and 2) compare the quality of reporting of the primary qualitative studies included within these reviews prior- and post COREQ-publication.

**Methods:**

Reviews were searched on 02-Sept-2020 and categorized as (1) COREQ- or (2) ENTREQ-using, (3) using both, or (4) non-COREQ/ENTREQ. Proportions of usage were calculated over time. COREQ-scores of the primary studies included in these reviews were compared prior- and post COREQ-publication using T-test with Bonferroni correction.

**Results:**

1.695 qualitative reviews were included (222 COREQ, 369 ENTREQ, 62 both COREQ/ENTREQ and 1.042 non-COREQ/ENTREQ), spanning 12 years (2007–2019) demonstrating an exponential publication rate. The uptake of the ENTREQ in reviews is higher than the COREQ (respectively 28% and 17%), and increases over time. COREQ-scores could be extracted from 139 reviews (including 2.775 appraisals). Reporting quality improved following the COREQ-publication with 13 of the 32 signalling questions showing improvement; the average total score increased from 15.15 to 17.74 (*p*-value < 0.001).

**Conclusion:**

The number of qualitative reviews increased exponentially, but the uptake of the COREQ and ENTREQ was modest overall. Primary qualitative studies show a positive trend in reporting quality, which may have been facilitated by the publication of the COREQ.

**Supplementary Information:**

The online version contains supplementary material available at 10.1186/s12874-021-01363-1.

## Key findings

- The usage of the COREQ and ENTREQ in qualitative reviews is moderate, but is increasing for the ENTREQ
- Quality of reporting of single qualitative studies increased following the COREQ-publication, but the overall reporting quality remains modest
- Instead of using the COREQ checklist as published, a large number of reviews omitted signalling questions, or merged existing checklists with the COREQ
- Ongoing discussions on the merits of checklists in qualitative research are facilitated by data on a large sample of qualitative reviews and single qualitative studies
- We present a comprehensive and in-depth analysis of checklist usage and reporting quality on the level of single qualitative studies and qualitative reviews, using a systematic meta-review approach

## Introduction

Qualitative studies allow for a deeper understanding of people’s experiences, beliefs, attitudes or behaviours. These studies usually focus on *why* participants think or act in a certain way, using open ended data gathering methods such as interviews, focus groups or observations [1, 2]. They can be regarded as hypothesis generating research, and while research methods fundamentally differ when compared to quantitative research, they are not necessarily incompatible nor mutually exclusive. Both methods can complement each other, for example hypotheses that originated from qualitative research may be statistically tested in quantitative research, or findings from quantitative research can be explained by qualitative research [3, 4]. As in all fields of research, poorly designed, conducted or reported qualitative studies can lead to inappropriate findings [5].

In 2007, the COREQ (Consolidated criteria for reporting qualitative research) checklist was developed to assess the reporting quality of qualitative studies [6]. Realizing that, in contrast to most other research fields, no widely used comprehensive checklist, nor uniform and accepted requirements for publication of qualitative research existed, the authors aimed to “… *promote complete and transparent reporting among researchers and indirectly improve the rigor, comprehensiveness and credibility of interview and focus-group studies.*” [6] Items from 22 published checklists were compiled into a single 32-item checklist and grouped into three domains (*research team and reflexivity*, *study design* and *data analysis and reporting*), thus creating a comprehensive checklist covering the main aspects of qualitative research.

Though aimed at researchers conducting an interview- or focus group study, the COREQ also became frequently used in reviews on qualitative studies to assess the reporting quality of the included studies in the absence of a checklist specifically developed for this purpose. Qualitative reviews, a novel study design, aims to systematically synthesize the included qualitative studies instead of generating original data to achieve abstraction and transferability at a higher level beyond the included original studies [7, 8]. While in 2007, when the COREQ was published, the number of qualitative reviews was relatively limited, in 2012 this number had increased substantially. Thus, using a similar approach as the COREQ, in 2012 members from the same research team and international experts developed the ENTREQ (Enhancing transparency in reporting the synthesis of qualitative research) checklist, for reviews as opposed to original studies [9]. This 21-item checklist covers five domains (*introduction, methods and methodology, literature search and selection, appraisal,* and *synthesis of findings)* and aims to *“… develop a framework for reporting the synthesis of qualitative health research.*” [9]

Since the publication of both checklists, a large number of reviews of qualitative studies have been published on a wide array of topics. Though it has been argued that reporting checklists for qualitative research would not necessarily result in *better* research [10], and neither checklists were developed following the now accepted methods for developing reporting standards [11], both the COREQ and the ENTREQ are now included in the EQUATOR network [12], and are required by many clinical journals for submission; the high number of citations (respectively over 5.600 and 700 in Web of Science) indeed indicate usage. To this date however, no studies have been conducted to explore the uptake of the COREQ and the ENTREQ in reviews, or the effect on the reporting quality, which for guidelines in other research methods has been the case [13–18]. Therefore, the aim of this meta-review is twofold: 1) to investigate the uptake of the COREQ and ENTREQ checklists in reviews of primary qualitative studies, and 2) to compare the quality of reporting of the original qualitative studies included in these reviews prior- and post-publication of the COREQ.

## Methods

This meta-review was reported in line with the PRISMA (Preferred Reporting Items for Systematic Reviews and Meta-Analyses) guidelines [19].

### Search strategy

Using similar searching methods as in previous studies, we developed three searches: the first search aimed to identify all qualitative reviews that cited the COREQ, the second aimed to identify all reviews that cited the ENTREQ; for these two searches, we used Web of Science and PubMed. Next, using terms encountered in these reviews, and building upon previous studies [20–23], we developed a comprehensive search method in PubMed to identify those reviews that did not specifically mention the COREQ or the ENTREQ. We then refined this broad search in an iterative process described in detail in the supplement, section A, and recoded the query to four other electronic databases: Cochrane library, Embase, Emcare and Web of Science. Searches were designed in collaboration with an experienced medical librarian and conducted on the 2nd of September 2020, including all articles since database inception (which differed per database). We then subtracted the results of the two other searches from this dataset. In the end, we thus obtained three databases: 1) studies citing the COREQ, 2) studies citing the ENTREQ, and 3) studies citing neither COREQ nor ENTREQ.

### Eligibility methods

Studies were eligible for inclusion if they were 1) a review and 2) contained qualitative or mixed-methods research approaches. We created four datasets: reviews using the 1) COREQ, 2) ENTREQ, 3) both the COREQ and ENTREQ and 4) neither the COREQ or ENTREQ. To be included in the respective datasets, reviews using the COREQ were required to appraise their included studies with this checklist; those using the ENTREQ were required to mention adherence to it. Reviews were imported in Endnote (version 9.1) and duplicates were removed. One author (YdJ) screened the titles for obvious irrelevance. Two authors (YdJ and JM) independently selected studies for eligibility based on abstract and full-text; conflicts were resolved after discussion. The selection procedure is explained in more detail in the supplement, section A.

### Data-extraction

Our study aimed to assess the uptake of the COREQ- and ENTREQ-checklists in reviews, but also to explore the effect of the COREQ on the reporting quality of original qualitative studies included in these reviews. For all reviews, we extracted the number of included qualitative studies, studies with mixed-method designs, and other designs (e.g. quantitative, reviews, etc.). For the first aim, we used the publication date of all the reviews from the meta-data of these reviews, rounded down to the month (i.e. MM/YYYY); if unavailable, we searched for the earliest publication date in online sources. For the second aim, we extracted the publication year (i.e. YYYY) and the COREQ scores of the original studies included in these reviews, as scored by the authors of these reviews (i.e. we did not rate the studies ourselves, but used the COREQ score as determined by the authors of these reviews, as illustrated in the supplemental Fig. S1). Data were extracted on three levels based on availability of the data: the score at the level of signalling questions (reported or not reported; 0 or 1), the total score per domain (0–8 for domain 1, 0–15 for domain 2, and 0–9 for domain 3), and the overall total score (0–32), where applicable. If no extractable information (e.g. no review COREQ score, but only an average per domain) was available, the corresponding author of that study was contacted. Data extraction was conducted by YdJ, JM, EvdW, and CV; all experienced in qualitative research, and familiar with both the COREQ and ENTREQ checklists.

### Statistical analysis

**Fig. 1:**
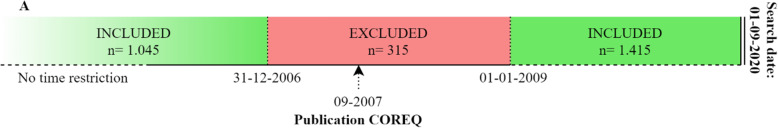
Schematic representation of inclusion periods used to assess the impact of the publication of the COREQ on the quality of reporting of the original qualitative studies (COREQ) included in qualitative reviews. For the COREQ, the COREQ score as assessed by the authors of the review was extracted and plotted over time using the publication date of that original qualitative study. All studies prior 2007 (so until and including 2006) were included, as were all those published after 2008 (so from and including 2009)

For the first aim, to investigate the uptake of the COREQ and the ENTREQ, we plotted the number of qualitative reviews using these checklists compared to those that did not use it over time, starting from the respective publication dates (i.e. 09–2007 and 11–2012). For the second aim, to assess whether the publication of the COREQ influenced the reporting quality of qualitative studies, we compared the average scores at the three levels (total score, domain scores and signalling questions) before publication of the COREQ (pre-COREQ: all studies before 2007) and after publication of the COREQ (post-COREQ: 2009–2019). Articles published in 2007 and 2008 were excluded, as the COREQ was published in September 2007 and this was regarded as a transition period, see Fig. 1. We used this transition period to avoid inclusion of studies that used a preliminary version of the COREQ (which was presented at a congress prior to publication – personal communication with Prof. A. Tong), and also to exclude studies that were in the submission process at the time of the publication date. To visualize the trends of the total COREQ score per domain, we plotted the absolute score over time, using a LOESS curve with a 95% confidence interval, and a span of 0.5. Average scores, as opposed to median scores, were calculated as in similar prior studies [24, 25], as this allows comparison on the level of signalling questions, increase precision of the estimated effect, and, though fundamentally different than LOESS modelling, allows comparison to these curves more than median scores. To compare the average scores prior- and post publication, we used unpaired T-tests. As some COREQ scores were missing, analyses were performed on complete cases. A significance level of *p* ≤ 0.05 was used, which was corrected for multiple testing using the Bonferroni approach. For the COREQ-analyses, we used a significance level of *p* < 0.0014 (0.05 divided by a total of 36 significance tests: 32 signalling questions, three domains and one for the total COREQ score). Analyses were performed in R, version 1.2.5001.

### Sensitivity analyses

We conducted three sensitivity analyses, all related to the second aim. 1) An analysis where we compared the COREQ scores prior- and post-publication without the transition period. 2) An analysis after imputation of missing COREQ scores, since a substantial number of reviews presented an adapted or incomplete COREQ score, usually without explanation. We assumed these missing data to be missing at random (MAR) and conducted five-multiple imputations using the R-package MICE; estimates were pooled according to Rubin’s rules. 3) An analysis of the effect of the inclusion of duplicate studies across reviews. Studies were considered a duplicate if the year of publication and name of the first author were identical. A detailed description of the sensitivity analyses is presented in the supplement, section C.

## Results

### Characteristics of included studies

**Fig. 2:**
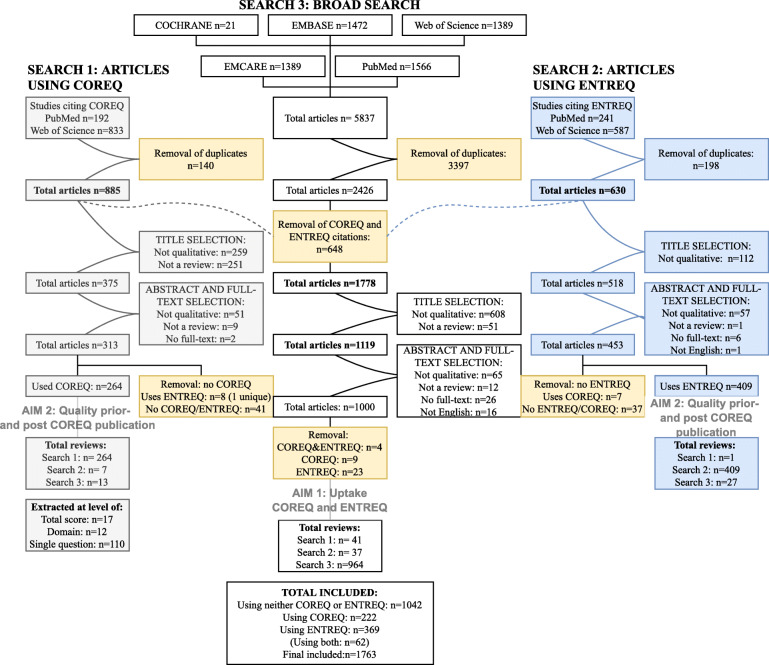
PRISMA flowchart of study inclusion. Three searches were conducted: the first search aimed to identify all reviews on qualitative research. The second and third searches were conducted in PubMed and Web of Science, and aimed to identify all reviews citing the COREQ and ENTREQ respectively. *The number of studies citing the COREQ or ENTREQ were subtracted from the total number of studies of search 1; since some studies cited both COREQ and ENTREQ, the total number of studies subtracted is less than the total numbers identified by the COREQ and ENTREQ searches

The three searches resulted in a total of 1.695 eligible reviews: 222 reviews used the COREQ for appraisal of their included studies, 369 used the ENTREQ, 62 reviews used both the COREQ and ENTREQ, and 1.042 used neither the COREQ or ENTREQ (Fig. 2). These 1.695 reviews included a total of 49.281 studies (median 19 studies per review, IQR 12–32), most of which were qualitative (78%, 38.279; median 14 studies per review, IQR 8–26). The remaining studies were of mixed-methods (4%; 2.177 studies; median 2 studies per review, IQR 1–4) and other methodology (18%; 8.825 studies; median 11 studies per review, IQR 5–22). A summary of the included reviews is presented in Table 1; an overview of all included reviews is given in the Supplement, section D.

**Table 1:** Summary of the 1.695 included qualitative reviews, grouped as COREQ- or ENTREQ using, using both checklists, or using neither checklist. An overview of each included review is presented in the supplement, section D. *Other study design includes all studies that are neither qualitative or mixed methods (e.g. quantitative, reviews, etc.)

	Total	COREQ	ENTREQ	Both COREQ/ENTREQ	Non- COREQ/ENTREQ
Total reviews (% of total)	1.695	222 (13%)	369 (22%)	62 (4%)	1.042 (61%)
Characteristics					
*Studies included in reviews*	49.281	6.069	9.715	2.042	31.455
Median (IQR)	19 (12–32)	20.50 (13–31.75)	18 (11–32)	28 (14.25–42.75)	19 (12–31)
*Qualitative (% of total)*	38.279 (78%)	3.527 (58%)	8.282 (85%)	1.915 (94%)	24.555 (78%)
Median (IQR)	14 (8–26)	10 (5–20)	14 (9–28.50)	26 (13–39.75)	15 (9–25)
*Mixed methods (% of total)*	2.177 (4%)	349 (6%)	453 (5%)	22 (1%)	1.353 (4%)
Median (IQR)	2 (1–4)	2 (2–5)	2 (1–4)	1.5 (1–3.50)	2 (1–4)
Other* (% of total)	8.825 (18%)	2.193 (36%)	980 (10%)	105 (5%)	5.547 (18%)
Median (IQR)	11 (5–22)	12 (7–21.25)	11 (5–19)	12 (8.50–29.75)	11 (5–22)

### Characteristics of reviews using the COREQ

For the 282 reviews that used the COREQ (i.e. 222 reviews using the COREQ alone; 62 using both COREQ and ENTREQ), most reviews presented their appraisal results in a table (*n* = 193; 68%), or textual only (*n* = 37, 13%), or a bar chart (*n =* 3, 1%). A large number of reviews appraised their included studies with the COREQ, but did not present the results (49 reviews; 17%). A total of 139 (49%) of the 282 reviews presented extractable data from individual studies, which was used to explore the trends in COREQ scores over time. Of these 139 reviews, data were presented at the level of signalling questions for 110 (79%), domains for 12 (9%) and total score for 17 (12%) of the reviews. In total, 2.775 COREQ appraisals of qualitative studies were extracted: 2.448 at the level of signalling questions, 200 at domain score, and 127 at overall total score. In more than half of the reviews, the COREQ checklist was adapted for study purposes (e.g. item exclusion) or COREQ-scores were incompletely reported: 47 out of the 110 reviews that reported at the level of signalling questions scored at least one of their included studies on all 32 signalling questions. The median completeness of the 32 COREQ-items was 25 (IQR 23–32; range 1–32), for the completeness of the individual signalling questions, see Table 2. As we used only the complete scores for our analyses (i.e. a complete case analysis), the number of appraisals included in the analysis for COREQ domains 1 to 3 was 1.036, 1.117, 1.086 respectively, and 831 appraisals for the overall total COREQ score.

**Table 2:** A complete-case comparison is made between those studies published prior to 2007 and those published after 2008. Because of this time-window, 315 studies were excluded for this analysis. Differences in mean scores were calculated by unpaired T-tests; significance (*p* < 0.05) is indicated by an asteriks (*); significance after Bonferroni correction (36 significance tests: 32 signalling questions, 3 domains, 1 total score, hence 0.05/36, *p* ≤ 0.0014) is indicated by two asteriks (**). % complete denotes the completeness of reporting for that specific signalling question. Ranges per domain: 0–8 for domain 1, 0–15 for domain 2, and 0–9 for domain 3

	TOTAL			PRE-COREQ (< 2007)			POST-COREQ (> 2008)				*P*
	*n*	*complete*	*Score*	*n*	*Score*	*SE*	*n*	*Score*	*SE*	*Difference*	
**DOMAIN I: RESEARCH TEAM AND REFLEXIVITY**	**1037**		**2.76**	**309**	**2.57**	**0.12**	**621**	**2.86**	**0.08**	**0.29**	**0.048**
Interviewer/facilitator	2218	91%	0.56	771	0.51	0.02	1187	0.58	0.01	0.08	0.001**
Credentials	1227	50%	0.42	379	0.41	0.03	708	0.42	0.02	0.01	0.788
Occupation	2092	85%	0.43	730	0.43	0.02	1121	0.41	0.01	−0.02	0.372
Gender	1300	53%	0.43	460	0.38	0.02	685	0.45	0.02	0.07	0.026*
Experience and training	2262	92%	0.25	799	0.20	0.01	1200	0.26	0.01	0.06	0.001**
Relationship established	2193	90%	0.18	757	0.18	0.01	1181	0.18	0.01	0.00	0.836
Participant knowledge of the interviewer	1022	42%	0.16	308	0.15	0.02	603	0.16	0.02	0.01	0.581
Interviewer characteristics	922	38%	0.20	236	0.16	0.02	585	0.23	0.02	0.07	0.020*
**DOMAIN II: STUDY DESIGN**	**1117**		**8.31**	**351**	**7.97**	**0.15**	**645**	**8.51**	**0.10**	**0.55**	0.007*
Methodological orientation and Theory	1206	49%	0.72	391	0.70	0.02	662	0.74	0.02	0.03	0.258
Sampling	2337	95%	0.77	815	0.73	0.02	1247	0.79	0.01	0.06	0.003*
Method of approach	2241	92%	0.71	785	0.66	0.02	1195	0.74	0.01	0.08	0.000**
Sample size	2384	97%	0.95	838	0.94	0.01	1259	0.96	0.01	0.02	0.023*
Non-participation	2229	91%	0.42	769	0.39	0.02	1203	0.45	0.01	0.06	0.008*
Setting of data collection	2377	97%	0.67	833	0.65	0.02	1258	0.67	0.01	0.02	0.339
Presence of nonparticipants	2237	91%	0.24	772	0.20	0.01	1207	0.26	0.01	0.06	0.001**
Description of sample	2394	98%	0.88	848	0.83	0.01	1259	0.90	0.01	0.07	0.000**
Interview guide	2329	95%	0.72	817	0.66	0.02	1234	0.77	0.01	0.12	0.000**
Repeat interviews	2195	90%	0.30	769	0.34	0.02	1177	0.28	0.01	−0.06	0.005*
Audio/visual recording	2262	92%	0.79	782	0.70	0.02	1219	0.83	0.01	0.13	0.000**
Field notes	2314	95%	0.33	814	0.29	0.02	1224	0.34	0.01	0.05	0.022*
Duration	2288	93%	0.61	793	0.57	0.02	1223	0.63	0.01	0.06	0.007*
Data saturation	2157	88%	0.29	753	0.20	0.01	1147	0.35	0.01	0.16	0.000**
Transcripts returned	1450	59%	0.13	494	0.11	0.01	789	0.15	0.01	0.04	0.028*
**DOMAIN III: ANALYSIS AND FINDINGS**	**1086**		**5.94**	**339**	**5.42**	**0.10**	**625**	**6.20**	**0.07**	**0.78**	0.000**
Number of data coders	2304	94%	0.60	785	0.49	0.02	1248	0.66	0.01	0.17	0.000**
Description of the coding tree	1333	54%	0.43	474	0.39	0.02	703	0.47	0.02	0.08	0.004*
Derivation of themes	2298	94%	0.80	800	0.74	0.02	1222	0.84	0.01	0.10	0.000**
Software	2312	94%	0.39	801	0.28	0.02	1234	0.45	0.01	0.17	0.000**
Participant checking	2259	92%	0.19	775	0.19	0.01	1225	0.19	0.01	0.01	0.752
Quotations presented	2384	97%	0.89	839	0.86	0.01	1260	0.92	0.01	0.06	0.000**
Data and findings consistent	2187	89%	0.77	749	0.72	0.02	1186	0.79	0.01	0.08	0.000**
Clarity of major themes	1085	44%	0.94	335	0.90	0.02	611	0.95	0.01	0.05	0.009*
Clarity of minor themes	993	41%	0.65	309	0.62	0.03	565	0.66	0.02	0.04	0.303
**TOTAL COREQ SCORE**	**831**		**16.99**	**232**	**15.51**	**0.31**	**509**	**17.74**	**0.20**	**2.23**	0.000**

### First aim: trends over time: uptake of COREQ and ENTREQ over time

**Fig. 3:**
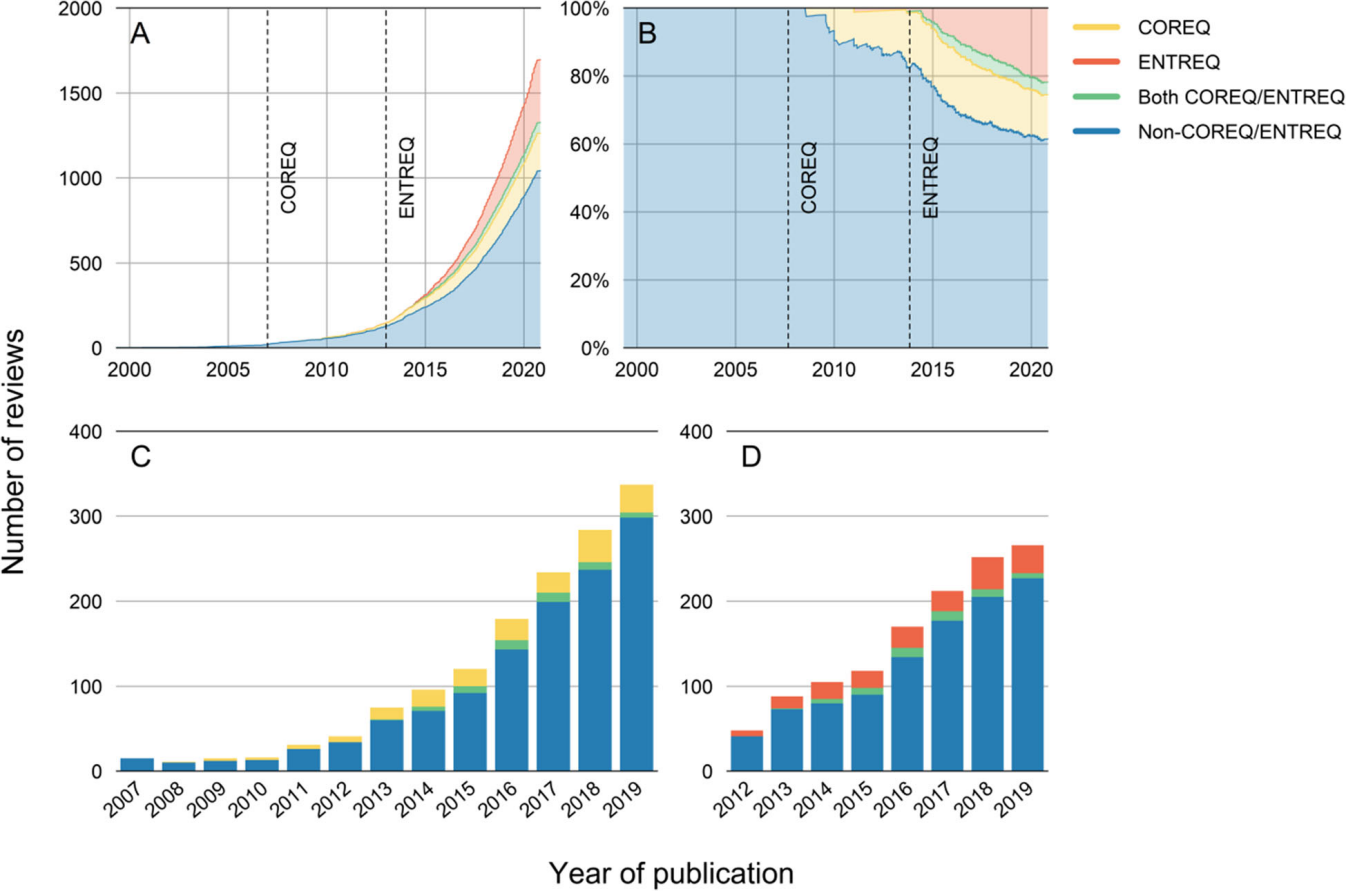
Uptake of the COREQ and the ENTREQ. **3A** Stacked chart of qualitative reviews over time. **3B** Percent stacked chart, showing the (cumulative) proportion of COREQ, ENTREQ and non-COREQ/ENTREQ reviews over time. **3C** Absolute number of COREQ versus non-COREQ (including ENTREQ and non-COREQ/ENTREQ reviews) stratified per year since the publication of the COREQ in 2007. **3D** Absolute number of ENTREQ versus non-ENTREQ (including COREQ and non-COREQ/ENTREQ reviews) stratified per year since the publication of the ENTREQ in 2012

The total number of reviews on qualitative studies increased exponentially over time (Fig. 3A). Until the publication of the COREQ in September 2007, only 31 reviews were identified; this number increased to 141 at the publication of the ENTREQ in November 2012. Of the total of 1.664 reviews published since the COREQ publication, 284 (17%) used the COREQ to assess the reporting quality of their included studies, this proportion remaining stable over time (Fig. 3B and C). For the ENTREQ, 431 reviews (28%) used this checklist out of the 1.554 reviews published since its publication, with this proportion increasing over time (Fig. 3B and D).

### Second aim: reporting quality prior- and post-publication of the COREQ

**Fig. 4:**
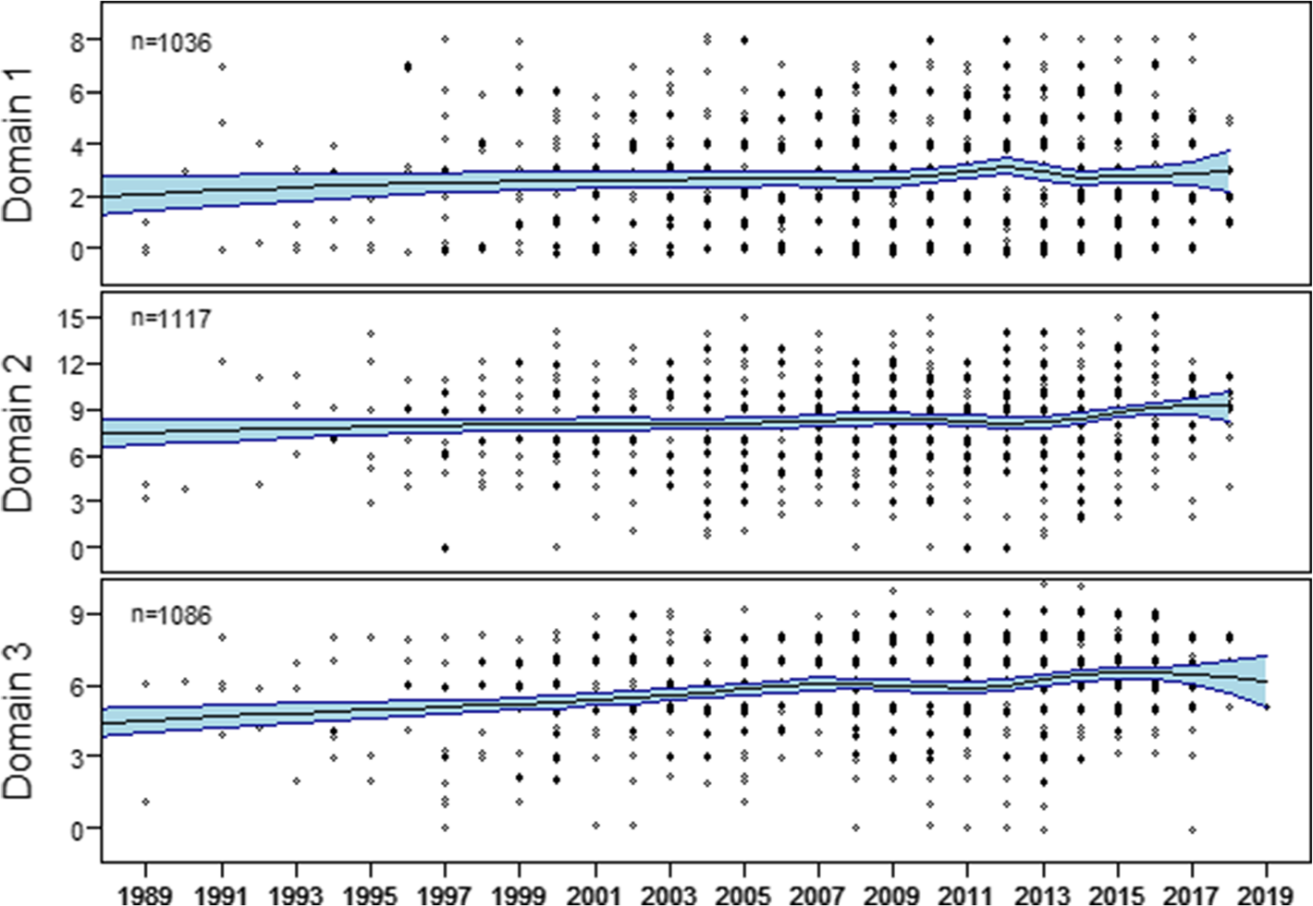
Trends for the three domains of the COREQ (domain 1: *research team and reflexivity;* domain 2: *study design* and domain 3: *data analysis and reporting)*, plotted over time, with a smoothed LOESS curve and 95% confidence interval (light blue). Y-axis differs per domain, as the number of signalling questions per domain is different (ranges per domain: 0–8 for domain 1, 0–15 for domain 2, and 0–9 for domain 3). For clarity, data points are jittered on the y-axis, by adding a Gaussian error with a standard deviation of 0.1

Of the 2.775 studies that were appraised with the COREQ, a total of 1.045 (39%) were published before 2007 and 1.415 (51%) after 2008; we thus excluded 315 (11%) studies for this analysis. The total COREQ score increased from 15.51 (SE 0.31) to 17.74 (SE 0.20, *p*-value < 0.001). The average scores per domain prior- and post-publication all increased: *research team and reflexivity:* 2.57, SE 0.12 before 2007 and 2.86, SE 0.08 after 2008 (difference 0.29, *p*-value 0.048), *study design*: 7.97, SE 0.15 before 2007 and 8.51, SE 0.10 after 2008 (difference 0.55, *p-*value 0.007), and *data analysis and reporting*: 5.42, SE 0.10 before 2007 and 6.20, SE 0.07 after 2008 (difference 0.78, *p-*value < 0.001). After Bonferroni correction, 13 out of the 32 signalling questions showed improvement. An overview of the average scores per signalling questions both prior- and post-publication of the COREQ is presented in Table 2, the positive trendline for each of the three domains is visualized in Fig. 4.

### Sensitivity analyses

When comparing the COREQ without the transition period, the improvement was less pronounced with 11 out of the 32 signalling questions showing changes after Bonferroni correction (one negative, the others positive; Table S3 in the supplement). For the second sensitivity analysis, we imputed the missing data assuming MAR. The results were similar, with 11 signalling questions showing a positive change (Table S4 in the supplement). Of the 2.775 studies included for the second aim, there were 185 (7%) studies included more than once (142 included two times, 31 included three times, and 12 included four or more times), resulting in a total duplicate count of 430. The results were similar to the main analysis, with 14 signalling questions showing a positive change (Table S5 in the supplement).

## Discussion

In this meta-review, we explored the uptake of the COREQ- and ENTREQ-checklists in qualitative reviews, and compared the reporting quality of original qualitive studies prior- and post COREQ publication. Though reviews of qualitative research are a novel methodology to achieve abstraction beyond the original qualitative studies, we demonstrated an exponential publication trend over the past twenty years. By including 1.695 reviews, that in turn included 49.281 studies, we were able to present an in-depth overview of current qualitative research – both at the level of reviews, as well as the level of individual studies included within these reviews. Answering the first research question, we found that the COREQ, published in 2007 to score the quality of reporting of original qualitive studies, was used in 17% of the reviews to appraise the reporting quality of their included studies. The ENTREQ, published in 2012 specifically for systematic reviews, showed a better uptake with 28% of the reviews using the checklist. Finally, using the COREQ-scores of 2.775 studies within these reviews, we demonstrated a positive trend in reporting quality since the publication of the COREQ, with 13 out of the 32 signalling questions showing improvement.

The uptake of the COREQ in qualitative reviews may be explained by the original aim of the COREQ, namely to improve quality of reporting in original interview- or focus-group studies [6]. In the absence of a comprehensive checklist for reporting the quality of qualitative reviews, the usage of the COREQ to appraise the reporting quality of studies within reviews may have followed naturally with the increasing numbers of qualitative reviews since its publication. The ENTREQ, specifically designed for reviews, showed a higher uptake [9]. Yet, appraising qualitative studies remains a debated topic. While some argue that adhering to checklists improves transparency and validity of findings, others feel endorsement as a limitation, arguing that a ‘one size fits all’ -set of criteria cannot encompass the broadness of qualitative research as a whole [5, 26–29]. In our study, this unresolved debate is clearly illustrated by the large number of reviews that adapted the COREQ for their purposes: more than half of the studies assessed their included studies with a selection of COREQ-items, or combined it with other checklists, both designed for reporting- or overall quality assessment, such as the CASP [30], QualSyst [31], GRADE-CREQual [32], MMAT [33], amongst others. The incomplete reporting, or the limited uptake of the COREQ and ENTREQ is not unique for qualitative research. For example, impact-studies on guidelines used for quantitative reviews [19], clinical trials [13, 34], observational studies [15, 16], prediction- or prognostic studies [14, 17], show that, even with endorsement of journals, the completeness of reporting remains suboptimal although for some, reporting quality improved.

By extracting the COREQ-scores of 2.775 appraisals included in these reviews, we were able to observe changes in the quality of reporting over time. On average, the total score, one of the three domains, and nearly half of the 32 signalling questions showed improvement when comparing studies published prior- versus post-publication of the COREQ. Though causal inferences cannot be made, this improvement, especially viewed in combination with the exponential trend of qualitative review publications, reflects the maturation and increasing acceptance of qualitative research. Although the overall quality of reporting improved, the scores of some items remained remarkably low: 16 out of the 32 signalling questions scored lower than an average score of 0.5. For example, in the first domain (“*research team and reflexivity*”), the items “*experience and training*”, “*relationship established*” and “*participant knowledge of the interviewer*” were reported poorly and did not improve markedly, with an average score of 0.25, 0.18 and 0.16, meaning that only 25, 18 and 16% of the articles reported these items, respectively. For the second domain (“*study design*”), most items were reported better than in the first domain, and improvements were even stronger. Nearly all items improved, and almost half remained significant after Bonferroni correction for multiple testing. The third domain (“*analysis and findings*”) showed good reporting on nearly all items, except for “*software*” and “*participant checking*”, though the first showed the largest improvement of all 32 items of the COREQ. These findings are in line with the two other studies that graded qualitative studies for the same purpose: Al-Moghrabi et al graded 100 qualitative studies, and demonstrated poor quality of reporting for most signalling questions [31]. In the second study, Godinho et al confirms this poor completeness of reporting in 246 Indian qualitative studies [24, 25]. When plotting the results over time, completeness of reporting remained modest, but increased over time, possibly facilitated by the publication of the COREQ and subsequent endorsement of journals [30].

The strengths of this study are the large sample size and comprehensive search methods. We conducted our study on reviews of qualitative studies (i.e. a meta-review). This method allowed for exploration of checklist usage in the same study type, namely reviews. Furthermore, the original qualitative studies included in these reviews are independently assessed for reporting quality by the authors of these reviews, assuring independent quality assessment and allowing for a large number of study appraisals to be included. We aimed to include as many studies as possible, tin order to present a comprehensive overview of all qualitative reviews. However, because of this large sample size, we did not perform complete cross-checking at two levels: title selection and data-extraction. We did cross-check the abstract- and full-texts for inclusion, showing excellent agreement (Cohen’s kappa coefficient for inter-rater reliability of 0.86 and 1.00 respectively). Data-extraction was cross-checked for 10 reviews, showing no errors. Furthermore, nearly all COREQ-studies could be extracted directly by recoding the COREQ-tables to our format, instead of typing the scores in our datasystem, thus reducing the risk of errors. Next, though misclassification of study type could be a more serious issue (e.g. misclassify a qualitative study design as mixed methods), all authors used the same methodology to classify the study types, as detailed in the supplement. Another limitation related to the COREQ-score is selection bias: studies of higher quality may have been easier to find in database-searches than those that are of lower quality (e.g. because of the use of identifiable terms as ‘thematic synthesis’ or ‘grounded theory’), possibly resulting in overestimation of the average COREQ scores. Furthermore, some review authors might have excluded studies based on their COREQ-score, which will result in an overestimation of the COREQ scores. Since the publication of the COREQ and ENTREQ, various new checklists have been published, both for appraising the reporting- and the overall study quality (e.g. the CASP in 2013 [30], the SRQR checklist in 2014, the eMERGe in 2019), underlining the developments in this research field since these guidelines. The use of these guidelines might partly explain the limited uptake of the COREQ and the ENTREQ, however we believe this to be to a limited extent since most reviews that did not use the COREQ or ENTREQ did not use any other checklist. Another explanation of the limited uptake may be improved retrievability of the post-COREQ and ENTREQ studies: including terms as ‘adhering to’, ‘appraising’, or naming these checklists likely increased the likelihood of inclusion in our review, compared to studies published prior these guidelines. Because of this, we based our search on previous studies [22, 23], designed our queries together with an experienced medical librarian, and conducted iterative search methods, and we thus believe this effect to be minimal. Lastly, it cannot be inferred that differences prior- and post-publication of the COREQ and ENTREQ are causally related to the publication of these checklists.

## Implications and conclusion

Our study highlights several points that may further improve the quality of reporting. First, surprisingly, almost a fifth of the reviews that used the COREQ did not present the results of their quality appraisal. Given that four out of the 21 ENTREQ-items, but also four of the 27 PRISMA-items concern study appraisal, at least reporting appraisal results should be the minimum. Ideally however, to facilitate meta-reviews of this kind, and to increase transparency and reproducibility, reporting appraisal results per individual study at the level of signalling questions is essential. Next, though we did not explore the characteristics of the authors of our included reviews, it can reasonably be assumed that the exponential publication trend may be explained by an increasing number of unique authors. Whether or not articles should be scored instead of appraised in a descriptive way remains open for discussion. However, the use of these checklists might be beneficial for new or inexperienced authors designing a qualitative study: checklists may guide those unfamiliar with qualitative research with hints and directions to avoid commonly made mistakes [5, 10, 27, 35]. The same holds true for reviewers assessing a qualitative review for publication, particularly if the reviewer has content expertise but not methodological expertise. A final implication concerns the poor reporting of several signalling questions of the COREQ. Whether or not these items are intentionally or unintentionally underreported, our study clearly points towards items that might either actually improve qualitative research if reported, or be left out from the checklist in a possible later or updated version. By providing this information on a large number of qualitative studies, our study might thus facilitate the ongoing discussions by providing factual data on both the use of checklists, and the completeness of reporting.

## Supplementary Information

**Additional file 1.**

## Data Availability

The datasets generated and/or analysed during the current study are not publicly available as the study is a systematic review and thus includes data from other studies, most with copyrights, but are available from the corresponding author on reasonable request.

## References

[1] Kuper A, Reeves S, Levinson W (2008). An introduction to reading and appraising qualitative research. BMJ.

[2] Giacomini MK, Cook DJ (2000). Users' guides to the medical literature: XXIII. Qualitative research in health care B. What are the results and how do they help me care for my patients? Evidence-Based Medicine Working Group. JAMA.

[3] O'Cathain A, Thomas KJ, Drabble SJ, et al. What can qualitative research do for randomised controlled trials? A systematic mapping review. BMJ Open. 2013;3(6). 10.1136/bmjopen-2013-002889 [published Online First: 2013/06/26]. 10.1136/bmjopen-2013-002889 PMC3669723 23794542

[4] Lewin S, Glenton C, Oxman AD (2009). Use of qualitative methods alongside randomised controlled trials of complex healthcare interventions: methodological study. BMJ.

[5] Reynolds J, Kizito J, Ezumah N (2011). Quality assurance of qualitative research: a review of the discourse. Health Res Policy Syst.

[6] Tong A, Sainsbury P, Craig J (2007). Consolidated criteria for reporting qualitative research (COREQ): a 32-item checklist for interviews and focus groups. Int J Qual Health Care.

[7] Dixon-Woods M, Agarwal S, Jones D (2005). Synthesising qualitative and quantitative evidence: a review of possible methods. J Health Serv Res Policy.

[8] Butler A, Hall H, Copnell B (2016). A Guide to Writing a Qualitative Systematic Review Protocol to Enhance Evidence-Based Practice in Nursing and Health Care. Worldviews Evid Based Nurs.

[9] Tong A, Flemming K, McInnes E (2012). Enhancing transparency in reporting the synthesis of qualitative research: ENTREQ. BMC Med Res Methodol.

[10] Hannes K, Heyvaert M, Slegers K (2015). Exploring the potential for a consolidated standard for reporting guidelines for qualitative research: an argument Delphi approach. Int J Qual Methods.

[11] Moher D, Schulz KF, Simera I (2010). Guidance for developers of Health Research reporting guidelines. PLoS Med.

[12] EQUATOR Network: Enhancing the QUAlity and Transparency Of health Research [Available from: https://www.equator-network.org/ accessed 11-11-2020.

[13] Moher D, Jones A, Lepage L (2001). Use of the CONSORT statement and quality of reports of randomized trials: a comparative before-and-after evaluation. JAMA.

[14] Zamanipoor Najafabadi AH, Ramspek CL, Dekker FW (2020). TRIPOD statement: a preliminary pre-post analysis of reporting and methods of prediction models. BMJ Open.

[15] Bastuji-Garin S, Sbidian E, Gaudy-Marqueste C (2013). Impact of STROBE statement publication on quality of observational study reporting: interrupted time series versus before-after analysis. PLoS One.

[16] Poorolajal J, Cheraghi Z, Irani AD (2011). Quality of Cohort Studies Reporting Post the Strengthening the Reporting of Observational Studies in Epidemiology (STROBE) Statement. Epidemiol Health.

[17] Sekula P, Mallett S, Altman DG (2017). Did the reporting of prognostic studies of tumour markers improve since the introduction of REMARK guideline? A comparison of reporting in published articles. PLoS One.

[18] Smidt N, Rutjes AW, van der Windt DA (2006). The quality of diagnostic accuracy studies since the STARD statement: has it improved?. Neurology.

[19] Moher D, Liberati A, Tetzlaff J (2009). Preferred reporting items for systematic reviews and meta-analyses: the PRISMA statement. PLoS Med.

[20] Ype, Jong Chava L., Ramspek Carmine, Zoccali Kitty J., Jager Friedo W., Dekker Merel, Diepen Appraising prediction research: a guide and meta‐review on bias and applicability assessment using the Prediction model Risk Of Bias ASsessment Tool (PROBAST). Nephrology. 10.1111/nep.13913. 10.1111/nep.13913 PMC9291738 34138495

[21] Ype Jong, Esmee M. Willik, Jet Milders, Yvette Meuleman, Rachael L. Morton, Friedo W. Dekker, Merel Diepen Person centred care provision and care planning in chronic kidney disease: which outcomes matter? A systematic review and thematic synthesis of qualitative studies. BMC Nephrology. 10.1186/s12882-021-02489-6. 10.1186/s12882-021-02489-6 PMC8438879 34517825

[22] Walters LA, Wilczynski NL, Haynes RB (2006). Developing optimal search strategies for retrieving clinically relevant qualitative studies in EMBASE. Qual Health Res.

[23] Barroso J, Gollop CJ, Sandelowski M (2003). The challenges of searching for and retrieving qualitative studies. West J Nurs Res.

[24] Godinho MA, Gudi N, Milkowska M (2019). Completeness of reporting in Indian qualitative public health research: a systematic review of 20 years of literature. J Public Health.

[25] Al-Moghrabi D, Tsichlaki A, Alkadi S (2019). How well are dental qualitative studies involving interviews and focus groups reported?. J Dent.

[26] Buus N, Agdal R (2013). Can the use of reporting guidelines in peer-review damage the quality and contribution of qualitative health care research?. Int J Nurs Stud.

[27] Barbour RS (2001). Checklists for improving rigour in qualitative research: a case of the tail wagging the dog?. BMJ.

[28] Dixon-Woods M, Shaw RL, Agarwal S (2004). The problem of appraising qualitative research. Qual Saf Health Care.

[29] Kuper A, Lingard L, Levinson W (2008). Critically appraising qualitative research. BMJ.

[30] Critical Appraisal Skills Programme (2018) CASP Qualitative checklist [Available from: https://casp-uk.net/wp-content/uploads/2018/03/CASP-Qualitative-Checklist-2018_fillable_form.pdf accessed accessed 14-10-2020.

[31] Kmet L, Lee R. Standard quality assessment criteria for evaluating primary research papers from a variety of FieldsAHFMRHTA Initiative20040213. HTA Initiative. 2004;2. https://www.ihe.ca/publications/standard-quality-assessment-criteria-for-evaluating-primary-research-papers-froma-variety-of-fields.

[32] Lewin S, Glenton C, Munthe-Kaas H (2015). Using qualitative evidence in decision making for health and social interventions: an approach to assess confidence in findings from qualitative evidence syntheses (GRADE-CERQual). PLoS Med.

[33] Hong QN, Fàbregues S, Bartlett G (2018). The mixed methods appraisal tool (MMAT) version 2018 for information professionals and researchers. Educ Inf.

[34] Turner L, Shamseer L, Altman DG (2012). Does use of the CONSORT Statement impact the completeness of reporting of randomised controlled trials published in medical journals? A Cochrane review. Syst Rev.

[35] Vandenbroucke JP (2009). STREGA, STROBE, STARD, SQUIRE, MOOSE, PRISMA, GNOSIS, TREND, ORION, COREQ, QUOROM, REMARK... and CONSORT: for whom does the guideline toll?. J Clin Epidemiol.

