# Supplementary material for: A meta-review demonstrates improved reporting quality of qualitative reviews following the publication of COREQ- and ENTREQ-checklists, regardless of modest uptake
Source: BMC Med Res Methodol. 2021 Sep 12;21:184. doi: 10.1186/s12874-021-01363-1 (PMC8436506; doi:10.1186/s12874-021-01363-1)
Supplement: Supplementary file 1 — Additional file 1. [file 12874_2021_1363_MOESM1_ESM.docx]

SUPPLEMENTARY INFORMATION FOR

**A meta-review demonstrates improved reporting quality of qualitative reviews following the publication of COREQ- and ENTREQ-checklists, regardless of modest uptake.**

Y. de Jong^1,2^, E.M. van der Willik^1^, J. Milders^1^, C.G.N. Voorend^2^, Rachael L Morton^3^, F.W. Dekker, Y. Meuleman^1^, M. van Diepen^1^

1. _­_Department of Clinical Epidemiology, Leiden University Medical Center, Leiden, the Netherlands
2. Department of Internal Medicine, Leiden University Medical Center, Leiden, the Netherlands
3. NHMRC Clinical Trials Centre, Faculty of Medicine and Health, The University of Sydney, Australia

Corresponding author: Y.de Jong, Leiden University Medical Center, Leiden the Netherlands. Department of Clinical Epidemiology, tel: +31 (0)71 526 4037; fax: +31 (0)71 526 6994, [y.de_jong@lumc.nl](mailto:y.de_jong@lumc.nl)

INDEX

Pages Section Description

2-8 **SECTION** **A:** **DETAILED SEARCH METHODS**

2 **Table S1** PubMed ID’s (PMID) of the target articles,

3-4 **Table S2** Iterative search refinement

5 Search queries for Embase, Emcare, Web of Science and Cochrane

6 - 8 **SECTION** **B:** **DETAILED SELECTION- AND DATA EXTRACTION METHODS**

6 Detailed study selection methods

7 Detailed data-extraction methods

8 **Figure S1** Schematic illustration of within-study COREQ score extraction from systematic reviews.

9-13 **SECTION C: SENSITIVITY ANALYSES**

9 Sensitivity analysis 1: no time-window for COREQ

9 Sensitivity analysis 2: multiple imputation of missing COREQ scores

10 **Table S3** Results for sensitivity analysis 1 (no time-window) 11 **Table S4** Results for sensitivity analysis 2 (multiple imputation)

12 Sensitivity analysis 3: duplicates check

13 **Table S5** Results for sensitivity analysis 3 (duplicate check)

14-102 **SECTION D: INCLUDED STUDIES**

14-25 Included reviews using COREQ (n=222)

26-45 Included reviews adhering to ENTREQ (n=369)

46-48 Included reviews using both COREQ and ENTREQ (n=62)

49-103 Included reviews using neither COREQ or ENTREQ (n=1042)

**SECTION** **A:** **DETAILED SEARCH METHODS**

Identifying qualitative studies is not without difficulties[sources]. We first drafted a broad search in PubMed, using terms commonly used in qualitative studies:

(ENTREQ[tiab] or COREQ[tiab] or "Qualitative studies"[tiab] or "Qualitative research"[tiab] or "Qualitative study"[tiab] or "qualitative evidence"[tiab] or "Thematic synthesis"[tiab] or Interview*[tiab] or "Focus group"[tiab] or "Audio-taped"[tiab] or "Data saturation"[tiab] or Ethnography[tiab] or Grounded-theory[tiab] or Content-analysis[tiab] or "lived experiences"[tiab] or "barrier"[tiab] or "facilitator"[tiab] or Field-studies[tiab]) and systematic review

**6.508 results (26-08-2020)**

From the results of this search, we selected 200 systematic reviews, using the ‘best match’ function of PubMed which sorts the results based on the number of search terms in the search fields:

| **PMID** | | | | | | | | | |
| --- | --- | --- | --- | --- | --- | --- | --- | --- | --- |
| 32829240 | 32349324 | 32335745 | 26341941 | 30813814 | 27696658 | 28614902 | 29672772 | 31657028 | 29914880 |
| 32827840 | 32007696 | 32164668 | 24003093 | 29808788 | 31617635 | 29863969 | 30095380 | 31578904 | 28267031 |
| 32826079 | 31834894 | 31888651 | 23865484 | 30390463 | 29248887 | 31262231 | 29802515 | 31931359 | 30992291 |
| 32823026 | 31429996 | 31739182 | 31794099 | 28820617 | 31787102 | 28204992 | 22972487 | 31147982 | 29666134 |
| 32819218 | 30614026 | 31690308 | 31793217 | 28069561 | 30180769 | 26372087 | 30848703 | 29768417 | 27235303 |
| 32813456 | 30589972 | 31118025 | 29202313 | 31184529 | 31201537 | 27531844 | 31046852 | 28041680 | 32040864 |
| 32813421 | 30563360 | 31099064 | 29793522 | 31726329 | 31875238 | 31233990 | 29203682 | 30798317 | 24843053 |
| 32813338 | 30461103 | 30471947 | 29141619 | 21192795 | 29867117 | 25847387 | 30140728 | 30975677 | 31219555 |
| 32803732 | 30389756 | 30010883 | 28780549 | 30125834 | 28932919 | 31358686 | 30948566 | 28089317 | 27401366 |
| 22008151 | 30293048 | 29998024 | 30474491 | 31694299 | 31862528 | 31874867 | 27693084 | 31390122 | 29194120 |
| 32799278 | 30227279 | 29879993 | 28960514 | 29210501 | 30955947 | 30618105 | 29762072 | 32020314 | 22673091 |
| 32798080 | 30094015 | 29696304 | 31745818 | 25525194 | 30404576 | 30745355 | 29669089 | 31235220 | 27729203 |
| 32791822 | 29966826 | 29625768 | 30139629 | 29729652 | 30029092 | 28993623 | 30629043 | 26839015 | 27810971 |
| 32773496 | 29804879 | 29326196 | 31492446 | 30561053 | 30843587 | 32677182 | 31566788 | 25847569 | 26684278 |
| 32772787 | 29685175 | 28715467 | 28285304 | 29669583 | 30066602 | 30175881 | 30709399 | 24612855 | 31147364 |
| 32722680 | 28424546 | 28695098 | 28131997 | 30144269 | 28726274 | 31869772 | 27758957 | 26988351 | 27245753 |
| 32720899 | 28167377 | 28545539 | 30584788 | 30812068 | 31514690 | 30782686 | 28776896 | 24506971 | 28601818 |
| 32711229 | 27030251 | 27542889 | 29488844 | 29327140 | 31714251 | 27432023 | 27246010 | 30328172 | 29954045 |
| 32699130 | 26335935 | 26440647 | 27417197 | 31003992 | 29057842 | 28402040 | 29736627 | 30886960 | 28363703 |
| 32658362 | 32796529 | 26367769 | 26314587 | 29947357 | 31332283 | 27134259 | 30384191 | 30594168 | 27907161 |

**Table S1** PubMed ID’s (PMID) of the target articles, i.e. systematic reviews on qualitative studies used for finetuning the final search.

*Iterative search refinement*

Next, we modified the search iterative. We aimed to develop a search that would at least identify 95% (n=190) of the target articles. In the first iteration, we checked the added value of each term by each time removing one term. We then checked the number of articles found, the number of target articles found, and the ratio of the reduction (6508 minus the number of articles found) and loss (200 minus the number of target articles found).

In the **second iteration**, we removed all search terms that did not have added value (i.e. removing the term did not result in loss of target article identification).

In the **third to final iteration**, we repeated the steps of the first iteration, and selected the search term with the highest reduction:loss ratio (i.e. the total number of results of the previous iteration minus number of articles found, divided by the total number of target articles found by the previous iteration minus the number of target articles found). See **Table S2** for the results.

The final search string in PubMed, after seven iterations, was:

(ENTREQ[tiab] or COREQ[tiab] or "Qualitative research"[tiab] or "Thematic synthesis"[tiab] ) and systematic review

**1.562 results (26-08-2020); 193 target articles (96.5% of the 200)**

| Search term removed | Found | Target | Reduction / loss | Ratio reduction:loss | % loss |  |
| --- | --- | --- | --- | --- | --- | --- |
| **ITERATION 1** |  |  | Compared to  6508 (100%) / 200 (100%) |  | Compared to  200 |  |
| ENTREQ[tiab] | 6507 | 199 | 1 / 1 | 1.00 | 99.50% |  |
| COREQ[tiab] | 6506 | 197 | 2 / 3 | 0.67 | 98.50% |  |
| "Qualitative studies"[tiab] | 5548 | 199 | 960 / 1 | 960.00 | 99.50% |  |
| "Qualitative research"[tiab] | 6074 | 180 | 434 / 20 | 21.70 | 90.00% |  |
| "Qualitative study"[tiab] | 6403 | 200 | 105 / 0 | no loss | 100.00% |  |
| "qualitative evidence"[tiab] | 6289 | 200 | 219 / 0 | no loss | 100.00% |  |
| Thematic synthesis[tiab] | 6341 | 197 | 167 / 3 | 55.67 | 98.50% |  |
| Interview*[tiab] | 4991 | 196 | 1517 / 4 | 379.25 | 98.00% |  |
| "Focus group"[tiab] | 6431 | 195 | 77 / 5 | 15.40 | 97.50% |  |
| "Audio-taped"[tiab] | 6507 | 200 | 1 / 0 | no loss | 100.00% |  |
| "Data saturation"[tiab] | 6506 | 200 | 2 / 0 | no loss | 100.00% |  |
| Ethnography[tiab] | 6442 | 200 | 66 / 0 | no loss | 100.00% |  |
| Grounded-theory[tiab] | 6485 | 200 | 23 / 0 | no loss | 100.00% |  |
| Content-analysis[tiab] | 6059 | 199 | 449 / 1 | 449.00 | 99.50% |  |
| lived experiences"[tiab] | 6494 | 200 | 14 / 0 | no loss | 100.00% |  |
| "barrier"[tiab] | 5475 | 200 | 1033 / 0 | no loss | 100.00% |  |
| facilitator"[tiab] | 6433 | 200 | 75 / 0 | no loss | 100.00% |  |
| or Field-studies[tiab] | 6452 | 200 | 56 / 0 | no loss | 100.00% |  |
| **ITERATION 2** |  |  | Compared to  6508 (100%) / 200 (100%) |  | Compared to  200 |  |
| "Qualitative study"[tiab]  "qualitative evidence"[tiab]  "Audio-taped"[tiab]  "Data saturation"[tiab]  Ethnography[tiab]  Grounded-theory[tiab]  lived experiences"[tiab]  "barrier"[tiab]  facilitator"[tiab]  or Field-studies[tiab] | 5043 | 198 | 1465 / 2 | 732.50 | 99.00% |  |
| **ITERATION 3: removed above** | | | Compared to  5043 (77%) / 198 (99%) |  | Compared to  200 |  |
| ENTREQ[tiab] | 5040 | 196 | 3 / 2 | 1.50 | 98.00% |  |
| COREQ[tiab] | 5040 | 195 | 3 / 3 | 1.00 | 97.50% |  |
| "Qualitative studies"[tiab] | 3910 | 196 | 1133 / 2 | 566.50 | 98.00% |  |
| "Qualitative research"[tiab] | 4453 | 165 | 590 / 33 | 17.88 | 82.50% |  |
| Thematic synthesis[tiab] | 4884 | 195 | 159 / 3 | 53.00 | 97.50% |  |
| Interview*[tiab] | 3444 | 198 | 1599 / 0 | no loss | 99.00% |  |
| "Focus group"[tiab] | 4964 | 197 | 79 / 1 | 79.00 | 98.50% |  |
| Content-analysis[tiab] | 4752 | 197 | 291 / 1 | 291.00 | 98.50% |  |
| **ITERATION 4: removed “interview*”** | | | Compared to  3444 (53%) / 198 (99%) |  | Compared to  200 | |
| ENTREQ[tiab] | 3441 | 196 | 3 / 2 | 1.50 | 98.00% |  |
| COREQ[tiab] | 3441 | 195 | 3 / 3 | 1.00 | 97.50% |  |
| "Qualitative studies"[tiab] | 2254 | 195 | 1190 / 3 | 396.67 | 97.50% |  |
| "Qualitative research"[tiab] | 2796 | 162 | 648 / 36 | 18.00 | 81.00% |  |
| Thematic synthesis[tiab] | 3239 | 195 | 205 / 3 | 68.33 | 97.50% |  |
| "Focus group"[tiab] | 3309 | 197 | 135 / 1 | 135.00 | 98.50% |  |
| Content-analysis[tiab] | 2942 | 197 | 502 / 1 | 502.00 | 98.50% |  |
| **ITERATION 5: removed “content-analysis”** | | | Compared to  2942 (45%) / 197 (98.5%) |  | Compared to  200 | |
| ENTREQ[tiab] | 2939 | 195 | 3 / 2 | 1.50 | 97.50% |  |
| COREQ[tiab] | 2939 | 194 | 3 / 3 | 1.00 | 97.00% |  |
| "Qualitative studies"[tiab] | 1717 | 194 | 1225 / 3 | 408.33 | 97.00% |  |
| "Qualitative research"[tiab] | 2279 | 160 | 663 / 37 | 17.92 | 80.00% |  |
| Thematic synthesis[tiab] | 2736 | 193 | 206 / 4 | 51.50 | 96.50% |  |
| "Focus group"[tiab] | 2800 | 196 | 142 / 1 | 142.00 | 98.00% |  |
| **ITERATION 6: removed “qualitative studies”** | | | Compared to  1717 (26%) / 194 (97.0%) |  | Compared to 200 | |
| ENTREQ[tiab] | 1710 | 189 | 7 / 5 | 1.40 | 94.50% |  |
| COREQ[tiab] | 1709 | 186 | 8 / 8 | 1.00 | 93.00% |  |
| "Qualitative research"[tiab] | 727 | 136 | 990 / 58 | 17.07 | 68.00% |  |
| Thematic synthesis[tiab] | 1368 | 189 | 349 / 5 | 69.80 | 94.50% |  |
| "Focus group"[tiab] | 1562 | 193 | 155 / 1 | 155.00 | 96.50% |  |
| **ITERATION 7: removed “focus group”** | | | Compared to  1562 (24%) / 193 (96.5%) |  | Compared to 200 | |
| ENTREQ[tiab] | 1555 | 188 | 7 / 5 | 27.00 | 94.00% |  |
| COREQ[tiab] | 1554 | 185 | 8 / 8 | 18.11 | 92.50% |  |
| "Qualitative research"[tiab] | 559 | 135 | 1003 / 58 | 19.63 | 67.50% |  |
| Thematic synthesis[tiab] | 1221 | 188 | 341 / 5 | 82.67 | 94.00% |  |

**Table S2** Iterative refining of the search strategy for PubMed.

We then transposed this search string to four other bibliographic databases: Cochrane library, Embase, Emcare, PubMed and Web of Science

**SECTION** **A:** **DETAILED SEARCH METHODS**

**Search string Cochrane Library**

("ENTREQ" OR "COREQ" OR "Qualitative research" OR "Thematic synthesis"):ti,ab

**Search string Embase and Emcare**

("ENTREQ".ti,ab OR "COREQ".ti,ab OR "Qualitative research".ti,ab OR "Thematic synthesis".ti,ab) AND (exp "systematic review"/ OR "systematic review".ti,ab) NOT ((conference review or conference abstract).pt OR "case report"/ OR "case report".ti)

**Search string Web of Science**

(TI=("ENTREQ" OR "COREQ" OR "Qualitative research" OR "Thematic synthesis") OR AB=("ENTREQ" OR "COREQ" OR "Qualitative research" OR "Thematic synthesis")) AND (TI=("systematic review") OR AB=("systematic review")) NOT (dt=(meeting abstract) OR ti=("case report"))

**SECTION** **B:** **DETAILED SELECTION- AND DATA EXTRACTION METHODS**

**Detailed study selection methods**

Using the search methods described above, we constructed three databases:

1. A database with articles citing the COREQ
2. A database with articles citing the ENTREQ
3. A database with #1 and #2, in combination with articles that did not cite the COREQ or ENTREQ. The references from #1 and #2 were subtracted from this dataset, resulting in a dataset with largely non-COREQ/ENTREQ articles.

Next, we checked these articles for eligibility. This consisted of multiple stages:

1. The references were checked for obvious irrelevance by one reviewer (YDJ). Articles were labelled as
   1. YES, which would include the article for the next stage (abstract selection)
   2. NO, which would then exclude the article. The reviewer had to choose a pre-defined reason:
      1. Not qualitative. This meant that the study was not (a review) of qualitative studies. This could also include reviews of reviews that were of qualitative studies, editorials, letters to the editor, commentaries on e.g. the COREQ or ENTREQ, etc.
      2. Not a review. This meant that the study was qualitative, but not a review. So this included only primary qualitative studies.
      3. No abstract or full-text. This meant that no abstract or full-text could be retrieved.
      4. Not of English language.
2. The abstracts were then checked by two reviewers (YDJ, JML), following the same procedure as above. Conflicts were discussed and resolved.
3. Then, the full-text articles were retrieved and checked for relevance following the same procedure.

We used this method for the COREQ and ENTREQ databases; for the non-COREQ/ENTREQ database, the abstract- and full-text eligibility was performed in one step, as for most articles eligibility was clear based on title.

**SECTION** **B:** **DETAILED SELECTION- AND DATA EXTRACTION METHODS**

**Detailed data-extraction methods**

After the study selection, we constructed an excel sheet for every dataset. We extracted the following information:

- **Date of publication**: the publication date of the review as stated in the meta-data of the article. If missing, we looked it up, rounded down to the month of the year (i.e. MM-YYYY).
- **Total number of studies**: the total number of studies included in the review. Usually, this information was presented in a flow-chart or in the first paragraph of the result section. If not present, we counted the number of articles in tables.
- **Type of study**: we classified the studies as ‘qualitative’, ‘mixed-methods’, or ‘other’
  - Qualitative: this included all types of qualitative studies, e.g. interviews, focus groups, observations, ethnographic research, content analysis, case study research, amongst others. Usually, the number of qualitative studies was well defined and presented in the articles.
  - Mixed-methods: we searched the articles for mixed-methods articles, by using phrases as ‘mixed’ or ‘quantitative’: in some cases, the author defined a mixed-method study as ‘qualitative and quantitative’. If the article stated clearly that mixed-methods studies were included, but these search methods did not yield results, we looked more precise in the article for mixed-methods studies, e.g. ‘interviews and surveys’ was a common description of mixed-methods studies. In general, mixed-methods studies were specified in the articles, and searching for ‘mixed-methods’ proved sufficient.
  - ‘Other’: this large group includes all articles that are not qualitative or mixed-methods.
- **Cites COREQ or ENTREQ**: this was a validation of our search. For every article, we checked whether it cited the COREQ or/and ENTREQ. We plainly searched for ‘COREQ’ or ‘ENTREQ’ – both references have this abbreviation in their title. If the article cited the COREQ or ENTREQ, but the abbreviation was not writing in the main draft of the article, we looked for ‘consolidated’ (for the COREQ) or ‘enhanced’ (for the ENTREQ).

For the COREQ, more information was extracted:

- **Presentation form**: the authors of the included review could present the COREQ on five different ways. These presentation forms are hierarchical (i.e. if presented in a table and bar chart, the presentation form will be ‘table’):
  1. Table: the results of study appraisal were presented in a table
  2. Textual only: the results were given textually. This could include a summary of the results, but also, in some cases, a paragraph with the scores per signalling question.
  3. Bar chart: the results were presented graphically, in the form of a bar chart
  4. Other: in some cases, none of above applied. Usually, it was stated that ‘the results can be obtained by contacting the author’, which was then done.
  5. Not reported: the authors score their included articles, but do not present any results.
- **Level of presentation**: either at the level of signalling questions, domain or total score.
- **COREQ score**: the within-study COREQ-score was extracted, as illustrated in Figure S1 on the next page.


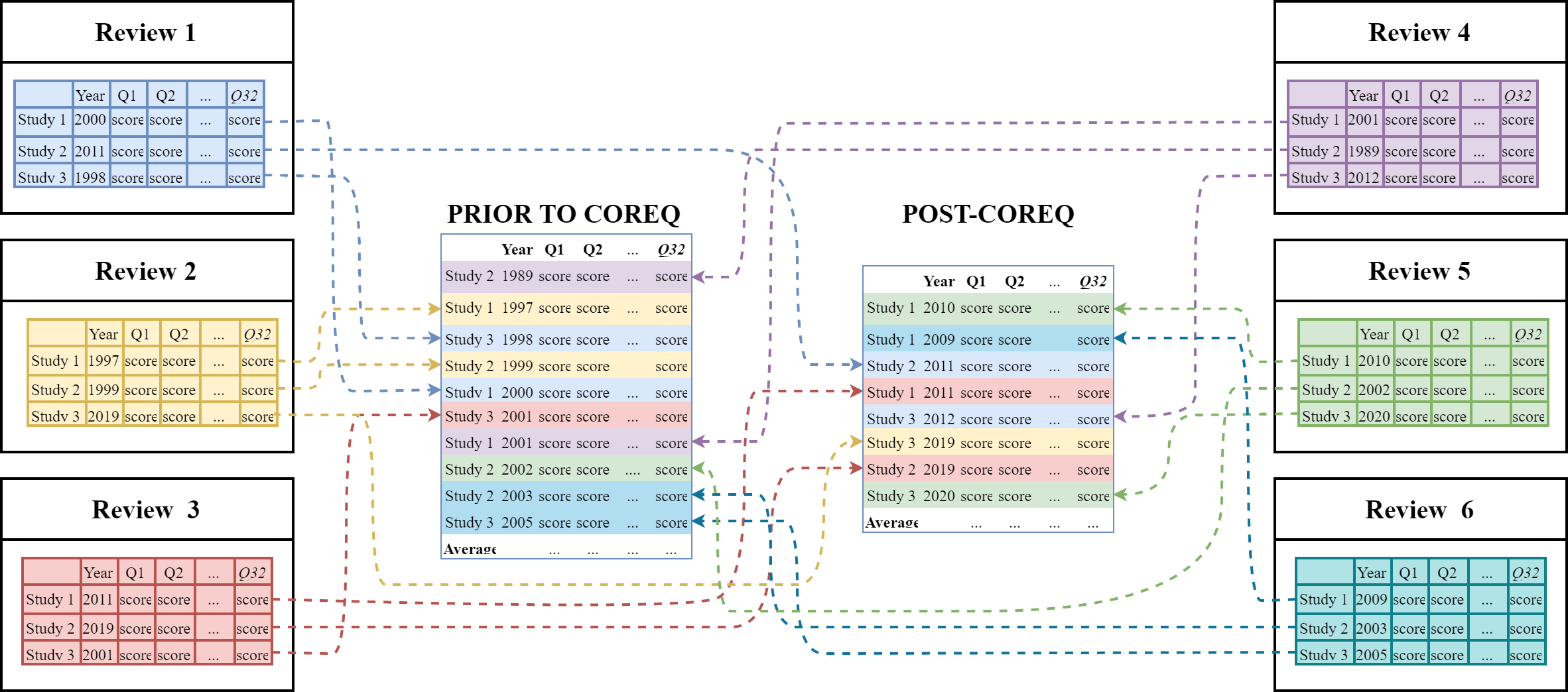


**Figure S1** Schematic illustration of within-study COREQ score extraction from systematic reviews. Of these six (hypothetical) systematic reviews using the COREQ to appraise the reporting quality of the included studies, the within-study COREQ scores on the 32 questions in the COREQ were extracted and stratified per publication year of the original study (so not the publication year of the systematic review).

**SECTION C: SENSITIVITY ANALYSES**

**Sensitivity analysis 1 and 2: no time-window for the COREQ**

For the main analysis, we used a time window prior- and post-publication of the COREQ. The reason for this time-window is that some authors might have known about the COREQ (e.g. from conferences) while others had not. Or, alternatively, an author might have submitted his article around publication, and would otherwise have included the COREQ in his study. The downside is that this time-window omits a portion of the articles. In this sensitivity analysis, we analyse the COREQ scores prior- and post publication without a time-interval. It should be noted that the year of publication of the within-study COREQ scores is rounded down to the year (e.g. 2017 includes all studies from the 1^st^ of January to the 31th of December). In the main analysis, we used all articles prior to 2007 (not including 2007) and after 2008 (not including 2008), creating a time window of at least two years and at maximum four years. In the present analysis, we will use a cut-off of 2008, so all articles published until 2008 (including 2007) and after 2008 (including 2008). See results in **Table S3** on the next page.

For the second sensitivity analysis, we imputed the missing data, assuming missingness at random (MAR). Using R-package MICE, we conducted a ten-fold imputation using all data as predictors. Results are presented in **Table S4**.

|  | **TOTAL** | | | **PRE-COREQ (<2007)** | | | **POST-COREQ (≥2008)** | | | |  |
| --- | --- | --- | --- | --- | --- | --- | --- | --- | --- | --- | --- |
|  | *n* | *\complete* | *Score* | *n* | *Score* | *SE* | *n* | *Score* | *SE* | *Difference* | *P* |
| **DOMAIN I:  RESEARCH TEAM AND REFLEXIVITY** | **1037** |  | **2.76** | **354** | **2.59** | **0.11** | **682** | **2.84** | **0.08** | **0.25** | **0.071** |
| Interviewer/facilitator | 2218 | 91% | 0.56 | 900 | 0.53 | 0.02 | 1318 | 0.58 | 0.01 | 0.06 | 0.010 |
| Credentials | 1227 | 50% | 0.42 | 446 | 0.41 | 0.02 | 781 | 0.42 | 0.02 | 0.01 | 0.644 |
| Occupation | 2092 | 85% | 0.43 | 850 | 0.44 | 0.02 | 1242 | 0.42 | 0.01 | -0.03 | 0.220 |
| Gender | 1300 | 53% | 0.43 | 535 | 0.41 | 0.02 | 765 | 0.44 | 0.02 | 0.04 | 0.180 |
| Experience and training | 2262 | 92% | 0.25 | 928 | 0.22 | 0.01 | 1334 | 0.27 | 0.01 | 0.05 | 0.007 |
| Relationship established | 2193 | 90% | 0.18 | 885 | 0.17 | 0.01 | 1308 | 0.18 | 0.01 | 0.01 | 0.732 |
| Participant knowledge of the interviewer | 1022 | 42% | 0.16 | 354 | 0.16 | 0.02 | 668 | 0.15 | 0.01 | -0.01 | 0.799 |
| Interviewer characteristics | 922 | 38% | 0.20 | 279 | 0.17 | 0.02 | 643 | 0.22 | 0.02 | 0.05 | 0.089 |
| **DOMAIN II:  STUDY DESIGN** | **1117** |  | **8.31** | **408** | **8.01** | **0.14** | **709** | **8.45** | **0.10** | **0.44** | **0.025** |
| Methodological orientation and Theory | 1206 | 49% | 0.72 | 464 | 0.70 | 0.02 | 742 | 0.73 | 0.02 | 0.03 | 0.332 |
| Sampling | 2337 | 95% | 0.77 | 951 | 0.74 | 0.01 | 1386 | 0.79 | 0.01 | 0.04 | 0.020 |
| Method of approach | 2241 | 92% | 0.71 | 912 | 0.67 | 0.02 | 1329 | 0.74 | 0.01 | 0.07 | 0.001 |
| Sample size | 2384 | 97% | 0.95 | 980 | 0.95 | 0.01 | 1404 | 0.96 | 0.01 | 0.01 | 0.114 |
| Non-participation | 2229 | 91% | 0.42 | 895 | 0.40 | 0.02 | 1334 | 0.44 | 0.01 | 0.03 | 0.124 |
| Setting of data collection | 2377 | 97% | 0.67 | 975 | 0.66 | 0.02 | 1402 | 0.67 | 0.01 | 0.02 | 0.432 |
| Presence of nonparticipants | 2237 | 91% | 0.24 | 901 | 0.20 | 0.01 | 1336 | 0.26 | 0.01 | 0.06 | 0.001 |
| Description of sample | 2394 | 98% | 0.88 | 989 | 0.84 | 0.01 | 1405 | 0.90 | 0.01 | 0.06 | 0.000 |
| Interview guide | 2329 | 95% | 0.72 | 951 | 0.66 | 0.02 | 1378 | 0.77 | 0.01 | 0.10 | 0.000 |
| Repeat interviews | 2195 | 90% | 0.30 | 891 | 0.34 | 0.02 | 1304 | 0.28 | 0.01 | -0.07 | 0.001 |
| Audio/visual recording | 2262 | 92% | 0.79 | 908 | 0.72 | 0.01 | 1354 | 0.83 | 0.01 | 0.11 | 0.000 |
| Field notes | 2314 | 95% | 0.33 | 948 | 0.30 | 0.01 | 1366 | 0.34 | 0.01 | 0.04 | 0.071 |
| Duration | 2288 | 93% | 0.61 | 927 | 0.59 | 0.02 | 1361 | 0.62 | 0.01 | 0.03 | 0.104 |
| Data saturation | 2157 | 88% | 0.29 | 877 | 0.21 | 0.01 | 1280 | 0.34 | 0.01 | 0.14 | 0.000 |
| Transcripts returned | 1450 | 59% | 0.13 | 572 | 0.11 | 0.01 | 878 | 0.14 | 0.01 | 0.03 | 0.060 |
| **DOMAIN III:  ANALYSIS AND FINDINGS** | **1086** |  | **5.94** | **395** | **5.54** | **0.09** | **691** | **6.16** | **0.07** | **0.63** | **0.000** |
| Number of data coders | 2304 | 94% | 0.60 | 917 | 0.51 | 0.02 | 1387 | 0.65 | 0.01 | 0.14 | 0.000 |
| Description of the coding tree | 1333 | 54% | 0.43 | 548 | 0.39 | 0.02 | 785 | 0.46 | 0.02 | 0.07 | 0.007 |
| Derivation of themes | 2298 | 94% | 0.80 | 933 | 0.75 | 0.01 | 1365 | 0.84 | 0.01 | 0.09 | 0.000 |
| Software | 2312 | 94% | 0.39 | 936 | 0.30 | 0.01 | 1376 | 0.45 | 0.01 | 0.15 | 0.000 |
| Participant checking | 2259 | 92% | 0.19 | 899 | 0.19 | 0.01 | 1360 | 0.18 | 0.01 | 0.00 | 0.772 |
| Quotations presented | 2384 | 97% | 0.89 | 979 | 0.86 | 0.01 | 1405 | 0.92 | 0.01 | 0.05 | 0.000 |
| Data and findings consistent | 2187 | 89% | 0.77 | 873 | 0.74 | 0.01 | 1314 | 0.79 | 0.01 | 0.06 | 0.003 |
| Clarity of major themes | 1085 | 44% | 0.94 | 401 | 0.92 | 0.01 | 684 | 0.95 | 0.01 | 0.03 | 0.032 |
| Clarity of minor themes |  |  |  |  |  |  |  |  |  |  |  |
| **TOTAL COREQ SCORE** | **831** |  | **16.99** | **273** | **15.82** | **0.28** | **558** | **17.56** | **0.20** | **1.74** | 0.000 |

**Table S3** Results for sensitivity analysis 1, where no time window is used.

|  | **TOTAL** | | | **PRE-COREQ (<2007)** | | | **POST-COREQ (≥2008)** | | | |  | |  |
| --- | --- | --- | --- | --- | --- | --- | --- | --- | --- | --- | --- | --- | --- |
|  | *n* | *Score* | *SE* | *n* | *Score* | *SE* | *n* | *Score* | *SE* | *Difference* | | *P* | |
| **DOMAIN I:  RESEARCH TEAM AND REFLEXIVITY** |  |  |  |  |  |  |  |  |  |  | |  | |
| Interviewer/facilitator | 2382 | 0.55 | 0.010 | 848 | 0.51 | 0.017 | 1249 | 0.58 | 0.017 | 0.07 | | 0.004 | |
| Credentials | 2382 | 0.41 | 0.010 | 848 | 0.41 | 0.017 | 1249 | 0.41 | 0.017 | 0.00 | | 0.884 | |
| Occupation | 2382 | 0.43 | 0.010 | 848 | 0.43 | 0.017 | 1249 | 0.42 | 0.017 | -0.01 | | 0.621 | |
| Gender | 2382 | 0.42 | 0.010 | 848 | 0.38 | 0.017 | 1249 | 0.44 | 0.017 | 0.06 | | 0.033 | |
| Experience and training | 2382 | 0.25 | 0.009 | 848 | 0.2 | 0.014 | 1249 | 0.27 | 0.015 | 0.07 | | 0.001 | |
| Relationship established | 2382 | 0.18 | 0.008 | 848 | 0.18 | 0.013 | 1249 | 0.19 | 0.013 | 0.01 | | 0.728 | |
| Participant knowledge of the interviewer | 2382 | 0.14 | 0.007 | 848 | 0.13 | 0.012 | 1249 | 0.15 | 0.012 | 0.02 | | 0.393 | |
| Interviewer characteristics | 2382 | 0.21 | 0.008 | 848 | 0.19 | 0.013 | 1249 | 0.22 | 0.014 | 0.03 | | 0.247 | |
| **DOMAIN II:  STUDY DESIGN** |  |  |  |  |  |  |  |  |  |  | |  | |
| Methodological orientation and Theory | 2382 | 0.70 | 0.009 | 848 | 0.69 | 0.016 | 1249 | 0.72 | 0.015 | 0.03 | | 0.176 | |
| Sampling | 2382 | 0.77 | 0.009 | 848 | 0.73 | 0.015 | 1249 | 0.78 | 0.014 | 0.05 | | 0.004 | |
| Method of approach | 2382 | 0.71 | 0.009 | 848 | 0.66 | 0.016 | 1249 | 0.74 | 0.015 | 0.08 | | 0.000 | |
| Sample size | 2382 | 0.95 | 0.004 | 848 | 0.94 | 0.008 | 1249 | 0.96 | 0.007 | 0.02 | | 0.012 | |
| Non-participation | 2382 | 0.43 | 0.010 | 848 | 0.39 | 0.017 | 1249 | 0.45 | 0.017 | 0.06 | | 0.008 | |
| Setting of data collection | 2382 | 0.66 | 0.010 | 848 | 0.65 | 0.016 | 1249 | 0.67 | 0.016 | 0.02 | | 0.444 | |
| Presence of nonparticipants | 2382 | 0.23 | 0.009 | 848 | 0.2 | 0.014 | 1249 | 0.26 | 0.015 | 0.06 | | 0.005 | |
| Description of sample | 2382 | 0.87 | 0.007 | 848 | 0.83 | 0.013 | 1249 | 0.9 | 0.01 | 0.07 | | 0.000 | |
| Interview guide | 2382 | 0.72 | 0.009 | 848 | 0.65 | 0.016 | 1249 | 0.77 | 0.015 | 0.12 | | 0.000 | |
| Repeat interviews | 2382 | 0.30 | 0.009 | 848 | 0.34 | 0.016 | 1249 | 0.28 | 0.015 | -0.06 | | 0.006 | |
| Audio/visual recording | 2382 | 0.78 | 0.008 | 848 | 0.71 | 0.016 | 1249 | 0.83 | 0.013 | 0.12 | | 0.000 | |
| Field notes | 2382 | 0.32 | 0.010 | 848 | 0.29 | 0.016 | 1249 | 0.34 | 0.016 | 0.05 | | 0.018 | |
| Duration | 2382 | 0.60 | 0.010 | 848 | 0.57 | 0.017 | 1249 | 0.63 | 0.017 | 0.06 | | 0.006 | |
| Data saturation | 2382 | 0.29 | 0.009 | 848 | 0.2 | 0.014 | 1249 | 0.35 | 0.016 | 0.15 | | 0.000 | |
| Transcripts returned | 2382 | 0.13 | 0.007 | 848 | 0.12 | 0.011 | 1249 | 0.15 | 0.012 | 0.03 | | 0.100 | |
| **DOMAIN III:  ANALYSIS AND FINDINGS** |  |  |  |  |  |  |  |  |  |  | |  | |
| Number of data coders | 2382 | 0.60 | 0.010 | 848 | 0.5 | 0.017 | 1249 | 0.66 | 0.016 | 0.16 | | 0.000 | |
| Description of the coding tree | 2382 | 0.47 | 0.010 | 848 | 0.42 | 0.017 | 1249 | 0.51 | 0.017 | 0.09 | | 0.007 | |
| Derivation of themes | 2382 | 0.80 | 0.008 | 848 | 0.74 | 0.015 | 1249 | 0.84 | 0.013 | 0.10 | | 0.000 | |
| Software | 2382 | 0.39 | 0.010 | 848 | 0.29 | 0.016 | 1249 | 0.45 | 0.017 | 0.16 | | 0.000 | |
| Participant checking | 2382 | 0.18 | 0.008 | 848 | 0.18 | 0.013 | 1249 | 0.19 | 0.013 | 0.01 | | 0.779 | |
| Quotations presented | 2382 | 0.89 | 0.006 | 848 | 0.86 | 0.012 | 1249 | 0.92 | 0.009 | 0.06 | | 0.000 | |
| Data and findings consistent | 2382 | 0.78 | 0.009 | 848 | 0.72 | 0.015 | 1249 | 0.81 | 0.014 | 0.09 | | 0.000 | |
| Clarity of major themes | 2382 | 0.87 | 0.007 | 848 | 0.83 | 0.013 | 1249 | 0.88 | 0.011 | 0.05 | | 0.003 | |
| Clarity of minor themes | 2382 | 0.62 | 0.010 | 848 | 0.6 | 0.017 | 1249 | 0.63 | 0.017 | 0.03 | | 0.240 | |
|  |  |  |  |  |  |  |  |  |  |  | |  | |

**Table S4** Results for the pre- and post-COREQ analysis on imputed data.

**SECTION C: SENSITIVITY ANALYSES**

**Sensitivity analysis 3: removal of duplicates**

Duplicates were identified by matching the author name and year of publication of within-review articles. First, duplicates within a study were not regarded as duplicates, since we considered it unlikely that the author that review would include duplicate studies. We thus assumed that within-review duplicates were different articles (e.g. a prolific author might publish one or more articles in one year).

Next, duplicates between-review were matched using the same method (author and year of publication), and mean scores were calculated. The results are given in **Table S5** on the next page.

|  | **PRE-COREQ (<2007)** | | | **POST-COREQ (≥2008)** | | | |  | |  |
| --- | --- | --- | --- | --- | --- | --- | --- | --- | --- | --- |
|  | *n* | *Score* | *SE* | *n* | *Score* | *SE* | *Difference* | | *P* | |
| **DOMAIN I:  RESEARCH TEAM AND REFLEXIVITY** |  |  |  |  |  |  |  | |  | |
| Interviewer/facilitator | 680 | 0.49 | 0.02 | 1068 | 0.58 | 0.02 | 0.08 | | 0.000 | |
| Credentials | 369 | 0.41 | 0.03 | 671 | 0.42 | 0.02 | 0.01 | | 0.761 | |
| Occupation | 639 | 0.42 | 0.02 | 1004 | 0.41 | 0.02 | -0.01 | | 0.671 | |
| Gender | 436 | 0.38 | 0.02 | 656 | 0.45 | 0.02 | 0.07 | | 0.027 | |
| Experience and training | 708 | 0.21 | 0.01 | 1082 | 0.27 | 0.01 | 0.07 | | 0.001 | |
| Relationship established | 665 | 0.18 | 0.01 | 1067 | 0.18 | 0.01 | 0.01 | | 0.764 | |
| Participant knowledge of the interviewer | 305 | 0.15 | 0.02 | 580 | 0.16 | 0.02 | 0.02 | | 0.552 | |
| Interviewer characteristics | 234 | 0.16 | 0.02 | 565 | 0.22 | 0.02 | 0.06 | | 0.029 | |
| **DOMAIN II:  STUDY DESIGN** |  |  |  |  |  |  |  | |  | |
| Methodological orientation and Theory | 380 | 0.70 | 0.02 | 634 | 0.74 | 0.02 | 0.04 | | 0.175 | |
| Sampling | 721 | 0.74 | 0.02 | 1127 | 0.79 | 0.01 | 0.05 | | 0.011 | |
| Method of approach | 694 | 0.66 | 0.02 | 1076 | 0.73 | 0.01 | 0.08 | | 0.001 | |
| Sample size | 743 | 0.94 | 0.01 | 1139 | 0.96 | 0.01 | 0.02 | | 0.071 | |
| Non-participation | 697 | 0.39 | 0.02 | 1092 | 0.44 | 0.01 | 0.05 | | 0.036 | |
| Setting of data collection | 739 | 0.64 | 0.02 | 1139 | 0.66 | 0.01 | 0.02 | | 0.290 | |
| Presence of nonparticipants | 682 | 0.18 | 0.01 | 1088 | 0.25 | 0.01 | 0.07 | | 0.000 | |
| Description of sample | 756 | 0.83 | 0.01 | 1143 | 0.90 | 0.01 | 0.06 | | 0.000 | |
| Interview guide | 728 | 0.65 | 0.02 | 1117 | 0.76 | 0.01 | 0.12 | | 0.000 | |
| Repeat interviews | 681 | 0.33 | 0.02 | 1062 | 0.27 | 0.01 | -0.05 | | 0.016 | |
| Audio/visual recording | 694 | 0.70 | 0.02 | 1102 | 0.82 | 0.01 | 0.12 | | 0.000 | |
| Field notes | 725 | 0.29 | 0.02 | 1107 | 0.33 | 0.01 | 0.05 | | 0.039 | |
| Duration | 705 | 0.55 | 0.02 | 1106 | 0.63 | 0.01 | 0.08 | | 0.001 | |
| Data saturation | 672 | 0.19 | 0.02 | 1048 | 0.35 | 0.01 | 0.16 | | 0.000 | |
| Transcripts returned | 470 | 0.11 | 0.01 | 754 | 0.14 | 0.01 | 0.04 | | 0.053 | |
| **DOMAIN III:  ANALYSIS AND FINDINGS** |  |  |  |  |  |  |  | |  | |
| Number of data coders | 697 | 0.50 | 0.02 | 1130 | 0.66 | 0.01 | 0.16 | | 0.000 | |
| Description of the coding tree | 445 | 0.38 | 0.02 | 670 | 0.46 | 0.02 | 0.09 | | 0.003 | |
| Derivation of themes | 722 | 0.73 | 0.02 | 1111 | 0.83 | 0.01 | 0.10 | | 0.000 | |
| Software | 714 | 0.30 | 0.02 | 1117 | 0.45 | 0.01 | 0.15 | | 0.000 | |
| Participant checking | 689 | 0.17 | 0.01 | 1110 | 0.19 | 0.01 | 0.01 | | 0.439 | |
| Quotations presented | 753 | 0.85 | 0.01 | 1145 | 0.91 | 0.01 | 0.06 | | 0.000 | |
| Data and findings consistent | 670 | 0.73 | 0.02 | 1079 | 0.80 | 0.01 | 0.08 | | 0.000 | |
| Clarity of major themes | 334 | 0.90 | 0.02 | 594 | 0.95 | 0.01 | 0.05 | | 0.010 | |
| Clarity of minor themes | 309 | 0.63 | 0.03 | 548 | 0.66 | 0.02 | 0.03 | | 0.374 | |
|  |  |  |  |  |  |  |  | |  | |

**Table S5** Results for the pre- and post-COREQ analysis excluding duplicates in signalling questions.

**SECTION D: INCLUDED REVIEWS**

Included reviews using COREQ (n=222)

1. Aghaei F, Shaghaghi A, Sarbakhsh P. A systematic review of the research evidence on cross-country features of illegal abortions. Health Promot Perspect. 2017;7(3):117-23.

2. Agius NM, Wilkinson A. Students' and teachers' views of written feedback at undergraduate level: A literature review. Nurse Education Today. 2014;34(4):552-9.

3. Albuquerque S, Pereira M, Narciso I. Couple's Relationship After the Death of a Child: A Systematic Review. Journal of Child and Family Studies. 2016;25(1):30-53.

4. Alsaeed D, Jamieson E, Gul MO, Smith FJ. Challenges to optimal medicines use in people living with dementia and their caregivers: A literature review. International Journal of Pharmaceutics. 2016;512(2):396-404.

5. Andriessen K, Krysinska K, Dransart DAC, Dargis L, Mishara BL. Grief After Euthanasia and Physician-Assisted Suicide A Systematic Review. Crisis-the Journal of Crisis Intervention and Suicide Prevention. 2020;41(4):255-72.

6. Archibald D, Douglas F, Hoddinott P, van Teijlingen E, Stewart F, Robertson C, et al. A qualitative evidence synthesis on the management of male obesity. BMJ Open. 2015;5(10):e008372.

7. Atchan M, Davis D, Foureur M. A methodological review of qualitative case study methodology in midwifery research. Journal of Advanced Nursing. 2016;72(10):2259-71.

8. Barlow EM, Dickens GL. Systematic review of therapeutic leave in inpatient mental health services. Archives of Psychiatric Nursing. 2018;32(4):638-49.

9. Barnes LAJ, Barclay L, McCaffery K, Aslani P. Complementary medicine products used in pregnancy and lactation and an examination of the information sources accessed pertaining to maternal health literacy: a systematic review of qualitative studies. BMC Complement Altern Med. 2018;18(1):229.

10. Bas-Sarmiento P, Fernandez-Gutierrez M, Baena-Banos M, Correro-Bermejo A, Soler-Martins PS, de la Torre-Moyano S. Empathy training in health sciences: A systematic review. Nurse Education in Practice. 2020;44.

11. Bastawrous M, Gignac MA, Kapral MK, Cameron JI. Factors that contribute to adult children caregivers' well-being: a scoping review. Health & Social Care in the Community. 2015;23(5):449-66.

12. Batchelor F, Hwang K, Haralamhous B, Fearn M, Mackell P, Nolte L, et al. Facilitators and barriers to advance care planning implementation in Australian aged care settings: A systematic review and thematic analysis. Australasian Journal on Ageing. 2019;38(3):173-81.

13. Bee P, Price O, Baker J, Lovell K. Systematic synthesis of barriers and facilitators to service user-led care planning. Br J Psychiatry. 2015;207(2):104-14.

14. Behan L, Rubbo B, Lucas JS, Dunn Galvin A. The patient's experience of primary ciliary dyskinesia: a systematic review. Qual Life Res. 2017;26(9):2265-85.

15. Berndtsson I, Dahlborg E, Pennbrant S. Work-integrated learning as a pedagogical tool to integrate theory and practice in nursing education - An integrative literature review. Nurse Education in Practice. 2020;42.

16. Blaschke S. The role of nature in cancer patients' lives: a systematic review and qualitative meta-synthesis. BMC Cancer. 2017;17(1):370.

17. Bokhof B, Junius-Walker U. Reducing Polypharmacy from the Perspectives of General Practitioners and Older Patients: A Synthesis of Qualitative Studies. Drugs Aging. 2016;33(4):249-66.

18. Bongaerts THG, Buchner FL, Middelkoop BJC, Guicherit OR, Numans ME. Determinants of (non-)attendance at the Dutch cancer screening programmes: A systematic review. Journal of Medical Screening.

19. Boruszak-Kiziukiewicz J, Kmita G. Parenting Self-Efficacy in Immigrant Families-A Systematic Review. Front Psychol. 2020;11:985.

20. Boye LK, Mogensen CB, Mechlenborg T, Waldorff FB, Andersen PT. Older multimorbid patients' experiences on integration of services: a systematic review. BMC Health Serv Res. 2019;19(1):795.

21. Bressan V, Bagnasco A, Aleo G, Catania G, Zanini MP, Timmins F, et al. The life experience of nutrition impact symptoms during treatment for head and neck cancer patients: a systematic review and meta-synthesis. Support Care Cancer. 2017;25(5):1699-712.

22. Brisset C, Leanza Y, Laforest K. Working with interpreters in health care: A systematic review and meta-ethnography of qualitative studies. Patient Education and Counseling. 2013;91(2):131-40.

23. Bristowe K, Marshall S, Harding R. The bereavement experiences of lesbian, gay, bisexual and/or trans* people who have lost a partner: A systematic review, thematic synthesis and modelling of the literature. Palliat Med. 2016;30(8):730-44.

24. Bromley EG, May FP, Federer L, Spiegel BM, van Oijen MG. Explaining persistent under-use of colonoscopic cancer screening in African Americans: a systematic review. Prev Med. 2015;71:40-8.

25. Brunstad A, Aasekj?r K, Aune I, Nilsen ABV. Fathers' experiences during the first postnatal week at home after early discharge of mother and baby from the maternity unit: A meta-synthesis. Scandinavian Journal of Public Health. 2020;48(4):362-75.

26. Buus N, Gonge H. Empirical studies of clinical supervision in psychiatric nursing: A systematic literature review and methodological critique. International Journal of Mental Health Nursing. 2009;18(4):250-64.

27. Callinan J, Coyne I. Arts-based interventions to promote transition outcomes for young people with long-term conditions: A review. Chronic Illness. 2020;16(1):23-40.

28. Carroll C, Booth A, Cooper K. A worked example of "best fit" framework synthesis: a systematic review of views concerning the taking of some potential chemopreventive agents. BMC Med Res Methodol. 2011;11:29.

29. Carroll C, Booth A, Papaioannou D, Sutton A, Wong R. UK Health-Care Professionals' Experience of On-Line Learning Techniques: A Systematic Review of Qualitative Data. Journal of Continuing Education in the Health Professions. 2009;29(4):235-41.

30. Choudhry V, Dayal R, Pillai D, Kalokhe AS, Beier K, Patel V. Child sexual abuse in India: A systematic review. PLoS One. 2018;13(10):e0205086.

31. Cooke A, Bedwell C, Campbell M, McGowan L, Ersser SJ, Ersser SJ. Skin care for healthy babies at term: A systematic review of the evidence. Midwifery. 2018;56:29-43.

32. Coussens M, Van Driessen E, De Baets S, Van Regenmortel J, Desoete A, Oostra A, et al. Parents' perspectives on participation of young children with attention deficit hyperactivity disorder, developmental coordination disorder, and/ or autism spectrum disorder: A systematic scoping review. Child Care Health and Development. 2020;46(2):232-43.

33. Crawford K, Wilson C, Low JK, Manias E, Williams A. Transitioning adolescents to adult nephrology care: a systematic review of the experiences of adolescents, parents, and health professionals. Pediatr Nephrol. 2020;35(4):555-67.

34. Cubis L, Ownsworth T, Pinkham MB, Chambers S. The social trajectory of brain tumor: a qualitative metasynthesis. Disability and Rehabilitation. 2018;40(16):1857-69.

35. Danielsen AK, Burcharth J, Rosenberg J. Spouses of patients with a stoma lack information and support and are restricted in their social and sexual life: a systematic review. Int J Colorectal Dis. 2013;28(12):1603-12.

36. de Lacy-Vawdon CJ, Klein R, Schwarzman J, Nolan G, de Silva R, Menzies D, et al. Facilitators of Attendance and Adherence to Group-Based Physical Activity for Older Adults: A Literature Synthesis. Journal of Aging and Physical Activity. 2018;26(1):155-67.

37. Delicate A, Ayers S, Easter A, McMullen S. The impact of childbirth-related post-traumatic stress on a couple's relationship: a systematic review and meta-synthesis. Journal of Reproductive and Infant Psychology. 2018;36(1):102-15.

38. Delicate A, Ayers S, McMullen S. A systematic review and meta-synthesis of the impact of becoming parents on the couple relationship. Midwifery. 2018;61:88-96.

39. Denham AMJ, Wynne O, Baker AL, Spratt NJ, Loh M, Turner A, et al. The long-term unmet needs of informal carers of stroke survivors at home: a systematic review of qualitative and quantitative studies. Disability and Rehabilitation.

40. Dickens GL, Lamont E, Gray S. Mental health nurses' attitudes, behaviour, experience and knowledge regarding adults with a diagnosis of borderline personality disorder: systematic, integrative literature review. Journal of Clinical Nursing. 2016;25(13-14):1848-75.

41. Doedens P, Vermeulen J, Boyette LL, Latour C, de Haan L. Influence of nursing staff attitudes and characteristics on the use of coercive measures in acute mental health services-A systematic review. Journal of Psychiatric and Mental Health Nursing. 2020;27(4):446-59.

42. du Preez J, Richmond J, Marquis R. Issues affecting Australian grandparents who are primary caregivers of grandchildren: a review. Journal of Family Studies. 2017;23(1):142-59.

43. Dunphy K, Baker FA, Dumaresq E, Carroll-Haskins K, Eickholt J, Ercole M, et al. Creative Arts Interventions to Address Depression in Older Adults: A Systematic Review of Outcomes, Processes, and Mechanisms. Front Psychol. 2018;9:2655.

44. Economides JM, Choi YK, Fan KL, Kanuri AP, Song DH. Are We Witnessing a Paradigm Shift?: A Systematic Review of Social Media in Residency. Plast Reconstr Surg Glob Open. 2019;7(8):e2288.

45. Edwards MW, Schweitzer RD, Shakespeare-Finch J, Byrne A, Gordon-King K. Living with nuclear energy: A systematic review of the psychological consequences of nuclear power. Energy Research & Social Science. 2019;47:1-15.

46. Ekberg S, Bradford NK, Herbert A, Danby S, Yates P. Healthcare Users' Experiences of Communicating with Healthcare Professionals About Children Who Have Life-Limiting Conditions: A Qualitative Systematic Review. Journal of Palliative Medicine. 2018;21(10):1518-28.

47. El-Gabri D, McDow AD, Quamme SP, Hooper-Lane C, Greenberg CC, Long KL. Surgical Coaching for Advancement of Global Surgical Skills and Capacity: A Systematic Review. Journal of Surgical Research. 2020;246:499-505.

48. Escobar-Viera CG, Whitfield DL, Wessel CB, Shensa A, Sidani JE, Brown AL, et al. For Better or for Worse? A Systematic Review of the Evidence on Social Media Use and Depression Among Lesbian, Gay, and Bisexual Minorities. JMIR Ment Health. 2018;5(3):e10496.

49. Esteban E, Coenen M, Ito E, Gruber S, Scaratti C, Leonardi M, et al. Views and Experiences of Persons with Chronic Diseases about Strategies that Aim to Integrate and Re-Integrate Them into Work: A Systematic Review of Qualitative Studies. Int J Environ Res Public Health. 2018;15(5).

50. Evripidou M, Charalambous A, Middleton N, Papastavrou E. Nurses' knowledge and attitudes about dementia care: Systematic literature review. Perspectives in Psychiatric Care. 2019;55(1):48-60.

51. Ewen C, Jenkins H, Jackson C, Jutley-Neilson J, Galvin J. Well-being, job satisfaction, stress and burnout in speech-language pathologists: A review. International Journal of Speech-Language Pathology.

52. Fahner JC, Beunders AJM, van der Heide A, Rietjens JAC, Vanderschuren MM, van Delden JJM, et al. Interventions Guiding Advance Care Planning Conversations: A Systematic Review. Journal of the American Medical Directors Association. 2019;20(3):227-48.

53. Fontes CMB, de Menezes DV, Borgato MH, Luiz MR. Communicating bad news: an integrative review of the nursing literature. Revista Brasileira De Enfermagem. 2017;70(5):1089-95.

54. Forcina V, Vakeesan B, Paulo C, Mitchell L, Bell JA, Tam S, et al. Perceptions and attitudes toward clinical trials in adolescent and young adults with cancer: a systematic review. Adolesc Health Med Ther. 2018;9:87-94.

55. Froud R, Patterson S, Eldridge S, Seale C, Pincus T, Rajendran D, et al. A systematic review and meta-synthesis of the impact of low back pain on people's lives. BMC Musculoskelet Disord. 2014;15:50.

56. Garcia MC, Chapman JR, Shaw PJ, Gottlieb DJ, Ralph A, Craig JC, et al. Motivations, Experiences, and Perspectives of Bone Marrow and Peripheral Blood Stem Cell Donors: Thematic Synthesis of Qualitative Studies. Biology of Blood and Marrow Transplantation. 2013;19(7):1046-58.

57. Garrison NA, Sathe NA, Antommaria AH, Holm IA, Sanderson SC, Smith ME, et al. A systematic literature review of individuals' perspectives on broad consent and data sharing in the United States. Genet Med. 2016;18(7):663-71.

58. Generalova D, Cunningham S, Leslie SJ, Rushworth GF, McIver L, Stewart D. A systematic review of clinicians' views and experiences of direct-acting oral anticoagulants in the management of nonvalvular atrial fibrillation. Br J Clin Pharmacol. 2018;84(12):2692-703.

59. Ghimire S, Hallett J, Gray C, Lobo R, Crawford G. What Works? Prevention and Control of Sexually Transmitted Infections and Blood-Borne Viruses in Migrants from Sub-Saharan Africa, Northeast Asia and Southeast Asia Living in High-Income Countries: A Systematic Review. Int J Environ Res Public Health. 2019;16(7).

60. Godinho MA, Gudi N, Milkowska M, Murthy S, Bailey A, Nair NS. Completeness of reporting in Indian qualitative public health research: a systematic review of 20 years of literature. Journal of Public Health. 2019;41(2):405-11.

61. Golfenshtein N, Srulovici E, Medoff-Cooper B. Investigating Parenting Stress Across Pediatric Health Conditions - A Systematic Review. Comprehensive Child and Adolescent Nursing-Buildng Evidence for Practice. 2016;39(1):41-79.

62. Gonçalves V, Sehovic I, Quinn G. Childbearing attitudes and decisions of young breast cancer survivors: a systematic review. Hum Reprod Update. 2014;20(2):279-92.

63. Gordon EJ, Daud A, Caicedo JC, Cameron KA, Jay C, Fryer J, et al. Informed Consent and Decision-Making About Adult-to-Adult Living Donor Liver Transplantation: A Systematic Review of Empirical Research. Transplantation. 2011;92(12):1285-96.

64. Gray WN, Netz M, McConville A, Fedele D, Wagoner ST, Schaefer MR. Medication adherence in pediatric asthma: A systematic review of the literature. Pediatric Pulmonology. 2018;53(5):668-84.

65. Gray WN, Schaefer MR, Resmini-Rawlinson A, Wagoner ST. Barriers to Transition From Pediatric to Adult Care: A Systematic Review. Journal of Pediatric Psychology. 2018;43(5):488-502.

66. Guo P, Watts K, Wharrad H. An integrative review of the impact of mobile technologies used by healthcare professionals to support education and practice. Nurs Open. 2016;3(2):66-78.

67. Hadgkiss EJ, Renzaho AMN. The physical health status, service utilisation and barriers to accessing care for asylum seekers residing in the community: a systematic review of the literature. Australian Health Review. 2014;38(2):142-59.

68. Hallett N, Huber JW, Dickens GL. Violence prevention in inpatient psychiatric settings: Systematic review of studies about the perceptions of care staff and patients. Aggression and Violent Behavior. 2014;19(5):502-14.

69. Hamel AV, Gaugler JE, Porta CM, Hadidi NN. Complex Decision-Making in Heart Failure A Systematic Review and Thematic Analysis. Journal of Cardiovascular Nursing. 2018;33(3):225-31.

70. Hansen JO, Bjerge B. What role does employment play in dual recovery? A qualitative meta-synthesis of cross-cutting studies treating substance use treatment, psychiatry and unemployment services. Advances in Dual Diagnosis. 2017;10(3):105-19.

71. Harvie A, Steel A, Wardle J. Traditional Chinese Medicine Self-Care and Lifestyle Medicine Outside of Asia: A Systematic Literature Review. Journal of Alternative and Complementary Medicine. 2019;25(8):789-808.

72. Hesse M, Forstmeier S, Mochamat M, Radbruch L. A Review of Biographical Work in Palliative Care. Indian J Palliat Care. 2019;25(3):445-54.

73. Hole E, Stubbs B, Roskell C, Soundy A. The patient's experience of the psychosocial process that influences identity following stroke rehabilitation: a metaethnography. ScientificWorldJournal. 2014;2014:349151.

74. Holmen H, Larsen MH, Sallinen MH, Thoresen L, Ahlsen B, Andersen MH, et al. Working with patients suffering from chronic diseases can be a balancing act for health care professionals - a meta-synthesis of qualitative studies. BMC Health Serv Res. 2020;20(1):98.

75. Husbands S, Mitchell PM, Coast J. A Systematic Review of the Use and Quality of Qualitative Methods in Concept Elicitation for Measures with Children and Young People. Patient. 2020;13(3):257-88.

76. Hussein S, Dahlen HG, Ogunsiji O, Schmied V. Women's experiences of childbirth in Middle Eastern countries: A narrative review. Midwifery. 2018;59:100-11.

77. Ion R, Smith K, Dickens G. Nursing and midwifery students' encounters with poor clinical practice: A systematic review. Nurse Education in Practice. 2017;23:67-75.

78. Irving MJ, Tong A, Jan S, Cass A, Rose J, Chadban S, et al. Factors that influence the decision to be an organ donor: a systematic review of the qualitative literature. Nephrology Dialysis Transplantation. 2012;27(6):2526-33.

79. Jabbarian LJ, Zwakman M, van der Heide A, Kars MC, Janssen DJA, van Delden JJ, et al. Advance care planning for patients with chronic respiratory diseases: a systematic review of preferences and practices. Thorax. 2018;73(3):222-30.

80. Janssen-Niemeijer AJ, Visse M, Van Leeuwen R, Leget C, Cusveller BS. The Role of Spirituality in Lifestyle Changing Among Patients with Chronic Cardiovascular Diseases: A Literature Review of Qualitative Studies. J Relig Health. 2017;56(4):1460-77.

81. Johnson JM, Hermosura BJ, Price SL, Gougeon L. Factors influencing interprofessional team collaboration when delivering care to community-dwelling seniors: A metasynthesis of Canadian interventions. Journal of Interprofessional Care.

82. Roozbeh N, Nahidi F, Hajiyan S. Barriers related to prenatal care utilization among women. Saudi Medical Journal. 2016;37(12):1319-27.

83. Burckhardt M, Belzner M, Berg A, Fleischer S. Living with breast cancer-related lymphedema: a synthesis of qualitative research. Oncol Nurs Forum. 2014;41(4):E220-37.

84. Medway M, Rhodes P. Young people's experience of family therapy for anorexia nervosa: a qualitative meta-synthesis. Advances in Eating Disorders: Theory, Research and Practice. 2016;4(2):189-207.

85. Park K, Caine V, Wimmer R. The Experiences of Advanced Placement and International Baccalaureate Diploma Program Participants: A Systematic Review of Qualitative Research. J Adv Acad. 2014;25(2):129-53.

86. Pawlow PC, Doherty CL, Blumenthal NP, Matura LA, Christie JD, Ersek M. An Integrative Review of the Role of Palliative Care in Lung Transplantation. Progress in transplantation (Aliso Viejo, Calif). 2020;30(2):147-54.

87. Rutherford C, Müller F, Faiz N, King MT, White K. Patient-reported outcomes and experiences from the perspective of colorectal cancer survivors: meta-synthesis of qualitative studies. J Patient Rep Outcomes. 2020;4(1):27.

88. Yang FC, Chen HM, Huang CM, Hsieh PL, Wang SS, Chen CM. The Difficulties and Needs of Organ Transplant Recipients during Postoperative Care at Home: A Systematic Review. Int J Environ Res Public Health. 2020;17(16).

89. Young RE, Broom D, Sage K, Crossland K, Smith C. Experiences of venue based exercise interventions for people with stroke in the UK: a systematic review and thematic synthesis of qualitative research. Physiotherapy. 2019.

90. Zhang K, Hannan E, Scholes-Robertson N, Baumgart A, Guha C, Kerklaan J, et al. Patients' perspectives of pain in dialysis: systematic review and thematic synthesis of qualitative studies. Pain. 2020.

91. Weaver MS, Heinze KE, Bell CJ, Wiener L, Garee AM, Kelly KP, et al. Establishing psychosocial palliative care standards for children and adolescents with cancer and their families: An integrative review. Palliative Medicine. 2016;30(3):212-23.

92. Fathnezhad Kazemi A, Hajian S, Ebrahimi-Mameghani M, Khob MK. The perspectives of pregnant women on health-promoting behaviors: An integrative systematic review. International Journal of Women's Health and Reproduction Sciences. 2018;6(2):97-105.

93. Elias CE, Day T. Experiences of Newly Qualified Nurses in Critical Care: A qualitative systematic review. Journal of the Intensive Care Society. 2020.

94. Kahan D. Critical Appraisal of Qualitative Studies of Muslim Females' Perceptions of Physical Activity Barriers and Facilitators. Int J Environ Res Public Health. 2019;16(24).

95. Karman P, Kool N, Poslawsky IE, Van Meijel B. Nurses' attitudes towards self-harm: a literature review. Journal of Psychiatric and Mental Health Nursing. 2015;22(1):65-75.

96. Kelly M, McCarthy S, Sahm LJ. Knowledge, attitudes and beliefs of patients and carers regarding medication adherence: a review of qualitative literature. Eur J Clin Pharmacol. 2014;70(12):1423-31.

97. Klinkenberg EF, Huis In't Veld EMJ, de Wit PD, van Dongen A, Daams JG, de Kort W, et al. Blood donation barriers and facilitators of Sub-Saharan African migrants and minorities in Western high-income countries: a systematic review of the literature. Transfus Med. 2019;29 Suppl 1(Suppl 1):28-41.

98. Kochen EM, Jenken F, Boelen PA, Deben LMA, Fahner JC, van den Hoogen A, et al. When a child dies: a systematic review of well-defined parent-focused bereavement interventions and their alignment with grief- and loss theories. BMC Palliat Care. 2020;19(1):28.

99. Kruithof K, Willems D, van Etten-Jamaludin F, Olsman E. Parents' knowledge of their child with profound intellectual and multiple disabilities: An interpretative synthesis. Journal of Applied Research in Intellectual Disabilities.

100. Kuithan P. Evidence-Based Practice in Physiotherapy: A Systematic Review of Barriers, Enablers and Intervention. Physioscience. 2016;12(2):79-U38.

101. Kuteesa MO, Seeley J, Cook S, Webb EL. Multi-level experiences and determinants of alcohol misuse and illicit drug use among occupational groups at high-risk of HIV in sub-Saharan Africa: A thematic synthesis of qualitative findings. Global Public Health. 2020;15(5):715-33.

102. Lacombe-Duncan A, Newman PA, Baiden P. Human papillomavirus vaccine acceptability and decision-making among adolescent boys and parents: A meta-ethnography of qualitative studies. Vaccine. 2018;36(19):2545-58.

103. Lamont E, Dickens GL. Mental health services, care provision, and professional support for people diagnosed with borderline personality disorder: systematic review of service-user, family, and carer perspectives. Journal of Mental Health.

104. Lemoyne SE, Herbots HH, De Blick D, Remmen R, Monsieurs KG, Van Bogaert P. Appropriateness of transferring nursing home residents to emergency departments: a systematic review. BMC Geriatr. 2019;19(1):17.

105. Leppala S, Lamminpaa R, Gissler M, Vehvilainen-Julkunen K. Humanitarian migrant women's experiences of maternity care in Nordic countries: A systematic integrative review of qualitative research. Midwifery. 2020;80.

106. Lerner JE, Robles G. Perceived Barriers and Facilitators to Health Care Utilization in the United States for Transgender People: A Review of Recent Literature. Journal of Health Care for the Poor and Underserved. 2017;28(1):127-52.

107. Lester R, Prescott L, McCormack M, Sampson M, North West Boroughs Healthcare NHS. Service users' experiences of receiving a diagnosis of borderline personality disorder: A systematic review. Personality and Mental Health. 2020;14(3):263-83.

108. Liefbroer AI, Olsman E, Ganzevoort RR, van Etten-Jamaludin FS. Interfaith Spiritual Care: A Systematic Review. J Relig Health. 2017;56(5):1776-93.

109. Liu SH, Morais SA, Lapane KL, Kay J. Physical activity and attitudes and perceptions towards physical activity in patients with spondyloarthritis: A systematic review. Seminars in Arthritis and Rheumatism. 2020;50(2):289-302.

110. Loewen PS, Ji A, Kapanen A, McClean A. Patient values and preferences for antithrombotic therapy in atrial fibrillation A Narrative Systematic Review. Thrombosis and Haemostasis. 2017;117(6):1007-22.

111. Lor M. Systematic Review: Health Promotion and Disease Prevention Among Hmong Adults in the USA. J Racial Ethn Health Disparities. 2018;5(3):638-61.

112. Macedo DR, Neris RR, dos Anjos ACY. Radiotherapy Experiences from the Perspective of Head and Neck Cancer Patients: Integrative Literature Review. Revista De Pesquisa-Cuidado E Fundamental Online. 2019;11(3):785-91.

113. Mackley MP, Fletcher B, Parker M, Watkins H, Ormondroyd E. Stakeholder views on secondary findings in whole-genome and whole-exome sequencing: a systematic review of quantitative and qualitative studies. Genet Med. 2017;19(3):283-93.

114. Magny-Normilus C, Whittemore R. Haitian Immigrants and Type 2 Diabetes: An Integrative Review. J Immigr Minor Health. 2020;22(2):399-409.

115. Maund E, Dewar-Haggart R, Williams S, Bowers H, Geraghty AWA, Leydon G, et al. Barriers and facilitators to discontinuing antidepressant use: A systematic review and thematic synthesis. Journal of Affective Disorders. 2019;245:38-62.

116. McFadden A, Siebelt L, Gavine A, Atkin K, Bell K, Innes N, et al. Gypsy, Roma and Traveller access to and engagement with health services: a systematic review. European Journal of Public Health. 2018;28(1):74-81.

117. McMahon S, Talley KM, Wyman JF. Older people's perspectives on fall risk and fall prevention programs: a literature review. Int J Older People Nurs. 2011;6(4):289-98.

118. Meiboom AA, de Vries H, Hertogh CM, Scheele F. Why medical students do not choose a career in geriatrics: a systematic review. BMC Med Educ. 2015;15:101.

119. Mellado BH, Pilger TL, Poli-Neto OB, Silva J, Nogueira AA, dos Reis FJC. Current usage of qualitative research in female pelvic pain: a systematic review. Archives of Gynecology and Obstetrics. 2019;300(3):495-501.

120. Mezini S, Soundy A. A Thematic Synthesis Considering the Factors which Influence Multiple Sclerosis Related Fatigue during Physical Activity. Behav Sci (Basel). 2019;9(7).

121. Michalski K, Farhan N, Motschall E, Vach W, Boeker M. Dealing with foreign cultural paradigms: A systematic review on intercultural challenges of international medical graduates. PLoS One. 2017;12(7):e0181330.

122. Morton RL, Tong A, Howard K, Snelling P, Webster AC. The views of patients and carers in treatment decision making for chronic kidney disease: systematic review and thematic synthesis of qualitative studies. Bmj. 2010;340:c112.

123. Murray J, Fenton G, Honey S, Bara AC, Hill KM, House A. A qualitative synthesis of factors influencing maintenance of lifestyle behaviour change in individuals with high cardiovascular risk. BMC Cardiovasc Disord. 2013;13:48.

124. Murray J, Hardicre N, Birks Y, O'Hara J, Lawton R. How older people enact care involvement during transition from hospital to home: A systematic review and model. Health Expect. 2019;22(5):883-93.

125. Murray J, Honey S, Hill K, Craigs C, House A. Individual influences on lifestyle change to reduce vascular risk: a qualitative literature review. Br J Gen Pract. 2012;62(599):e403-10.

126. Nelson KE, Lacombe-Duncan A, Cohen E, Nicholas DB, Rosella LC, Guttmann A, et al. Family Experiences With Feeding Tubes in Neurologic Impairment: A Systematic Review. Pediatrics. 2015;136(1):E140-E51.

127. Neubeck L, Ben Freedman S, Clark AM, Briffa T, Bauman A, Redfern J. Participating in cardiac rehabilitation: a systematic review and meta-synthesis of qualitative data. European Journal of Preventive Cardiology. 2012;19(3):494-503.

128. Nickel B, Barratt A, Copp T, Moynihan R, McCaffery K. Words do matter: a systematic review on how different terminology for the same condition influences management preferences. BMJ Open. 2017;7(7):e014129.

129. Nieuwboer MS, van der Sande R, van der Marck MA, Olde Rikkert MGM, Perry M. Clinical leadership and integrated primary care: A systematic literature review. Eur J Gen Pract. 2019;25(1):7-18.

130. Nnko S, Kuringe E, Nyato D, Drake M, Casalini C, Shao A, et al. Determinants of access to HIV testing and counselling services among female sex workers in sub-Saharan Africa: a systematic review. BMC Public Health. 2019;19(1):15.

131. O'Connell TS, Bedford KJ, Thiede M, McIntyre D. Synthesizing qualitative and quantitative evidence on non-financial access barriers: implications for assessment at the district level. Int J Equity Health. 2015;14:54.

132. O'Connor S, Glover L. Hospital staff experiences of their relationships with adults who self-harm: A meta-synthesis. Psychology and Psychotherapy-Theory Research and Practice. 2017;90(3):480-501.

133. Oedingen C, Bartling T, Mühlbacher AC, Schrem H, Krauth C. Systematic Review of Public Preferences for the Allocation of Donor Organs for Transplantation: Principles of Distributive Justice. Patient. 2019;12(5):475-89.

134. Oga-Omenka C, Tseja-Akinrin A, Sen P, Mac-Seing M, Agbaje A, Menzies D, et al. Factors influencing diagnosis and treatment initiation for multidrug-resistant/rifampicin-resistant tuberculosis in six sub-Saharan African countries: a mixed-methods systematic review. BMJ Glob Health. 2020;5(7).

135. Olaussen SJ, Renzaho AMN. Establishing components of cultural competence healthcare models to better cater for the needs of migrants with disability: a systematic review. Australian Journal of Primary Health. 2016;22(2):100-12.

136. Olza I, Leahy-Warren P, Benyamini Y, Kazmierczak M, Karlsdottir SI, Spyridou A, et al. Women's psychological experiences of physiological childbirth: a meta-synthesis. BMJ Open. 2018;8(10):e020347.

137. Pangas J, Ogunsiji O, Elmir R, Raman S, Liamputtong P, Burns E, et al. Refugee women's experiences negotiating motherhood and maternity care in a new country: A meta-ethnographic review. International Journal of Nursing Studies. 2019;90:31-45.

138. Parratt KA, Pina A. From "real rape" to real justice: A systematic review of police officers' rape myth beliefs. Aggression and Violent Behavior. 2017;34:68-83.

139. Pedley R, McWilliams C, Lovell K, Brooks H, Rushton K, Drake RJ, et al. Qualitative systematic review of barriers and facilitators to patient-involved antipsychotic prescribing. BJPsych Open. 2018;4(1):5-14.

140. Perkins A, Ridler J, Browes D, Peryer G, Notley C, Hackmann C. Experiencing mental health diagnosis: a systematic review of service user, clinician, and carer perspectives across clinical settings. Lancet Psychiatry. 2018;5(9):747-64.

141. Perrier L, Blondal E, MacDonald H. Exploring the experiences of academic libraries with research data management: A meta-ethnographic analysis of qualitative studies. Library & Information Science Research. 2018;40(3-4):173-83.

142. Pimentel CB, Lapane KL, Briesacher BA. Medicare part D and long-term care: a systematic review of quantitative and qualitative evidence. Drugs Aging. 2013;30(9):701-20.

143. Poikkeus T, Numminen O, Suhonen R, Leino-Kilpi H. A mixed-method systematic review: support for ethical competence of nurses. Journal of Advanced Nursing. 2014;70(2):256-71.

144. Powell MJ, Froggatt K, Giga S. Resilience in inpatient palliative care nursing: a qualitative systematic review. Bmj Supportive & Palliative Care. 2020;10(1):79-90.

145. Prorok JC, Horgan S, Seitz DP. Health care experiences of people with dementia and their caregivers: a meta-ethnographic analysis of qualitative studies. Cmaj. 2013;185(14):E669-80.

146. Punch R, Horstmanshof L. Hearing loss and its impact on residents in long term care facilities: A systematic review of literature. Geriatric Nursing. 2019;40(2):138-47.

147. Raman S, Nicholls R, Ritchie J, Razee H, Shafiee S. Eating soup with nails of pig: thematic synthesis of the qualitative literature on cultural practices and beliefs influencing perinatal nutrition in low and middle income countries. BMC Pregnancy Childbirth. 2016;16(1):192.

148. Raman S, Nicholls R, Ritchie J, Razee H, Shafiee S. How natural is the supernatural? Synthesis of the qualitative literature from low and middle income countries on cultural practices and traditional beliefs influencing the perinatal period. Midwifery. 2016;39:87-97.

149. Ratti V, Hassiotis A, Crabtree J, Deb S, Gallagher P, Unwin G. The effectiveness of person-centred planning for people with intellectual disabilities: A systematic review. Research in Developmental Disabilities. 2016;57:63-84.

150. Reeve E, To J, Hendrix I, Shakib S, Roberts MS, Wiese MD. Patient barriers to and enablers of deprescribing: a systematic review. Drugs Aging. 2013;30(10):793-807.

151. Resurreccion DM, Motrico E, Rigabert A, Rubio-Valera M, Conejo-Ceron S, Pastor L, et al. Barriers for Nonparticipation and Dropout of Women in Cardiac Rehabilitation Programs: A Systematic Review. Journal of Womens Health. 2017;26(8):849-59.

152. Riahi S, Khajehei M. Palliative Care: A Systematic Review of Evidence-Based Interventions. Critical Care Nursing Quarterly. 2019;42(3):315-28.

153. Rich ZC, Chu C, Mao J, Zhou K, Cai W, Ma Q, et al. Facilitators of HCV treatment adherence among people who inject drugs: a systematic qualitative review and implications for scale up of direct acting antivirals. BMC Public Health. 2016;16:994.

154. Robinson L, Gemski A, Abley C, Bond J, Keady J, Campbell S, et al. The transition to dementia - individual and family experiences of receiving a diagnosis: a review. International Psychogeriatrics. 2011;23(7):1026-43.

155. Rocque R, Leanza Y. A Systematic Review of Patients' Experiences in Communicating with Primary Care Physicians: Intercultural Encounters and a Balance between Vulnerability and Integrity. PLoS One. 2015;10(10):e0139577.

156. Romaniuk M, Kidd C. The Psychological Adjustment Experience of Reintegration Following Discharge from Military Service: A Systemic Review. Journal of Military and Veterans Health. 2018;26(2):60-73.

157. Romeiro J, Caldeira S. The Human Responses and Nursing Diagnoses of Those Living With Infertility: A Qualitative Systematic Review. International Journal of Nursing Knowledge. 2019;30(3):173-89.

158. Romeiro J, Caldeira S, Brady V, Timmins F, Hall J. Spiritual aspects of living with infertility: A synthesis of qualitative studies. Journal of Clinical Nursing. 2017;26(23-24):3917-35.

159. Rose A, Rosewilliam S, Soundy A. Shared decision making within goal setting in rehabilitation settings: A systematic review. Patient Education and Counseling. 2017;100(1):65-75.

160. Rubincam C, Lacombe-Duncan A, Newman PA. Taking culture seriously in biomedical HIV prevention trials: a meta-synthesis of qualitative studies. Expert Review of Vaccines. 2016;15(3):331-47.

161. Ryan T, M-Amen K, McKeown J. The advance care planning experiences of people with dementia, family caregivers and professionals: a synthesis of the qualitative literature. Annals of Palliative Medicine. 2017;6(4):380-9.

162. Rychetnik L, McCaffery K, Morton R, Irwig L. Psychosocial aspects of post-treatment follow-up for stage I/II melanoma: a systematic review of the literature. Psycho-Oncology. 2013;22(4):721-36.

163. Sabot K, Wickremasinghe D, Blanchet K, Avan B, Schellenberg J. Use of social network analysis methods to study professional advice and performance among healthcare providers: a systematic review. Syst Rev. 2017;6(1):208.

164. Salmasi S, De Vera MA, Barry A, Bansback N, Harrison M, Lynd LD, et al. Assessment of Condition and Medication Knowledge Gaps Among Atrial Fibrillation Patients: A Systematic Review and Meta-analysis. Annals of Pharmacotherapy. 2019;53(8):773-85.

165. Sarnola K, Merikoski M, Jyrkkä J, Hämeen-Anttila K. Physicians' perceptions of the uptake of biosimilars: a systematic review. BMJ Open. 2020;10(5):e034183.

166. Sartain SA, Stressing S, Prieto J. Patients' views on the effectiveness of patient-held records: a systematic review and thematic synthesis of qualitative studies. Health Expect. 2015;18(6):2666-77.

167. Sattoe JNT, Bal MI, Roelofs P, Bal R, Miedema HS, van Staa A. Self-management interventions for young people with chronic conditions: A systematic overview. Patient Education and Counseling. 2015;98(6):704-15.

168. Schveitzer MC, Zoboli EL, Vieira MM. Nursing challenges for universal health coverage: a systematic review. Rev Lat Am Enfermagem. 2016;24:e2676.

169. Scurlock-Evans L, Upton D. The Role and Nature of Evidence: A Systematic Review of Social Workers' Evidence-Based Practice Orientation, Attitudes, and Implementation. Journal of Evidence-Informed Social Work. 2015;12(4):369-99.

170. Scurlock-Evans L, Upton P, Upton D. Evidence-Based Practice in physiotherapy: a systematic review of barriers, enablers and interventions. Physiotherapy. 2014;100(3):208-19.

171. Sekse RJT, Dunberger G, Olesen ML, Østerbye M, Seibaek L. Lived experiences and quality of life after gynaecological cancer-An integrative review. J Clin Nurs. 2019;28(9-10):1393-421.

172. Sekse RJT, Hunskar I, Ellingsen S. The nurse's role in palliative care: A qualitative meta-synthesis. Journal of Clinical Nursing. 2018;27(1-2):E21-E38.

173. Serbin JW, Donnelly E. The Impact of Racism and Midwifery's Lack of Racial Diversity: A Literature Review. Journal of Midwifery & Womens Health. 2016;61(6):694-706.

174. Shakespeare C, Merriel A, Bakhbakhi D, Baneszova R, Barnard K, Lynch M, et al. Parents' and healthcare professionals' experiences of care after stillbirth in low- and middle-income countries: a systematic review and meta-summary. Bjog-an International Journal of Obstetrics and Gynaecology. 2019;126(1):12-21.

175. Sheehan AM, While AE, Coyne I. The experiences and impact of transition from child to adult healthcare services for young people with Type 1 diabetes: a systematic review. Diabetic Medicine. 2015;32(4):440-58.

176. Soundy A, Condon N. Patients experiences of maintaining mental well-being and hope within motor neuron disease: a thematic synthesis. Front Psychol. 2015;6:606.

177. Soundy A, Freeman P, Stubbs B, Probst M, Coffee P, Vancampfort D. The transcending benefits of physical activity for individuals with schizophrenia: A systematic review and meta-ethnography. Psychiatry Research. 2014;220(1-2):11-9.

178. Soundy A, Liles C, Stubbs B, Roskell C. Identifying a Framework for Hope in Order to Establish the Importance of Generalised Hopes for Individuals Who Have Suffered a Stroke. Adv Med. 2014;2014:471874.

179. Soundy A, Roskell C, Stubbs B, Probst M, Vancampfort D. INVESTIGATING THE BENEFITS OF SPORT PARTICIPATION FOR INDIVIDUALS WITH SCHIZOPHRENIA: A SYSTEMATIC REVIEW. Psychiatria Danubina. 2015;27(1):2-13.

180. Soundy A, Stubbs B, Freeman P, Coffee P, Roskell C. Factors influencing patients' hope in stroke and spinal cord injury: A narrative review. International Journal of Therapy and Rehabilitation. 2014;21(5):210-8.

181. Soundy A, Stubbs B, Roskell C. The experience of Parkinson's disease: a systematic review and meta-ethnography. ScientificWorldJournal. 2014;2014:613592.

182. Soundy A, Stubbs B, Roskell C, Williams SE, Fox A, Vancampfort D. Identifying the facilitators and processes which influence recovery in individuals with schizophrenia: a systematic review and thematic synthesis. Journal of Mental Health. 2015;24(2):103-10.

183. Staneva AA, Bogossian F, Wittkowski A. The experience of psychological distress, depression, and anxiety during pregnancy: A meta-synthesis of qualitative research. Midwifery. 2015;31(6):563-73.

184. Sutanto B, Singh-Grewal D, McNeil HP, O'Neill S, Craig JC, Jones J, et al. Experiences and Perspectives of Adults Living With Systemic Lupus Erythematosus: Thematic Synthesis of Qualitative Studies. Arthritis Care & Research. 2013;65(11):1752-65.

185. Taminiau-Bloem EF, Visser MR, Tishelman C, Koeneman MA, van Zuuren FJ, Sprangers MA. Somatically ill persons' self-nominated quality of life domains: review of the literature and guidelines for future studies. Qual Life Res. 2010;19(2):253-91.

186. Tan KK, Lopez V, Wong ML, Koh GC. Uncovering the barriers to undergoing screening among first degree relatives of colorectal cancer patients: a review of qualitative literature. J Gastrointest Oncol. 2018;9(3):579-88.

187. Timba-Emmanuel T, Kroll T, Renfrew MJ, MacGillivray S. Understanding the experiences of married Southern African women in protecting themselves from HIV/AIDS: a systematic review and meta-synthesis. Ethnicity & Health. 2019;24(6):623-44.

188. Tomlinson D, Zupanec S, Jones H, O'Sullivan C, Hesser T, Sung L. The lived experience of fatigue in children and adolescents with cancer: a systematic review. Support Care Cancer. 2016;24(8):3623-31.

189. Tong A, Chapman JR, Wong G, Cross NB, Batabyal P, Craig JC. The experiences of commercial kidney donors: thematic synthesis of qualitative research. Transplant International. 2012;25(11):1138-49.

190. Tong A, Chapman JR, Wong G, Josephson MA, Craig JC. Public Awareness and Attitudes to Living Organ Donation: Systematic Review and Integrative Synthesis. Transplantation. 2013;96(5):429-37.

191. Tong A, Chapman JR, Wong G, Kanellis J, McCarthy G, Craig JC. The Motivations and Experiences of Living Kidney Donors: A Thematic Synthesis. American Journal of Kidney Diseases. 2012;60(1):15-26.

192. Tong A, Hanson CS, Chapman JR, Halleck F, Budde K, Papachristou C, et al. The Preferences and Perspectives of Nephrologists on Patients' Access to Kidney Transplantation: A Systematic Review. Transplantation. 2014;98(7):682-91.

193. Tong A, Howard K, Jan S, Cass A, Rose J, Chadban S, et al. Community Preferences for the Allocation of Solid Organs for Transplantation: A Systematic Review. Transplantation. 2010;89(7):796-805.

194. Tong A, Howell M, Wong G, Webster AC, Howard K, Craig JC. The perspectives of kidney transplant recipients on medicine taking: a systematic review of qualitative studies. Nephrology Dialysis Transplantation. 2011;26(1):344-54.

195. Tong A, Jones J, Craig JC, Singh-Grewal D. Children's experiences of living with juvenile idiopathic arthritis: A thematic synthesis of qualitative studies. Arthritis Care & Research. 2012;64(9):1392-404.

196. Tong A, Lesmana B, Johnson DW, Wong G, Campbell D, Craig JC. The Perspectives of Adults Living With Peritoneal Dialysis: Thematic Synthesis of Qualitative Studies. American Journal of Kidney Diseases. 2013;61(6):873-88.

197. Tong A, Morton R, Howard K, Craig JC. Adolescent Experiences Following Organ Transplantation: A Systematic Review of Qualitative Studies. Journal of Pediatrics. 2009;155(4):542-9.

198. Tong A, Wong G, Hodson E, Walker RG, Tjaden L, Craig JC. Adolescent views on transition in diabetes and nephrology. Eur J Pediatr. 2013;172(3):293-304.

199. Traa MJ, De Vries J, Bodenmann G, Den Oudsten BL. Dyadic coping and relationship functioning in couples coping with cancer: A systematic review. British Journal of Health Psychology. 2015;20(1):85-114.

200. Turner S, D'Lima D, Hudson E, Morris S, Sheringham J, Swart N, et al. Evidence use in decision-making on introducing innovations: a systematic scoping review with stakeholder feedback. Implement Sci. 2017;12(1):145.

201. Ullgren H, Tsitsi T, Papastavrou E, Charalambous A. How family caregivers of cancer patients manage symptoms at home: A systematic review. International Journal of Nursing Studies. 2018;85:68-79.

202. Vaismoradi M, Wang IL, Turunen H, Bondas T. Older people's experiences of care in nursing homes: a meta-synthesis. International Nursing Review. 2016;63(1):111-21.

203. van den Berg MMJ, Dancet EAF, Erlikh T, van der Veen F, Goddijn M, Hajenius PJ. Patient-centered early pregnancy care: a systematic review of quantitative and qualitative studies on the perspectives of women and their partners. Human Reproduction Update. 2018;24(1):106-18.

204. van der Watt ASJ, van de Water T, Nortje G, Oladeji BD, Seedat S, Gureje O. The perceived effectiveness of traditional and faith healing in the treatment of mental illness: a systematic review of qualitative studies. Soc Psychiatry Psychiatr Epidemiol. 2018;53(6):555-66.

205. van Dongen SI, de Nooijer K, Cramm JM, Francke AL, Oldenmenger WH, Korfage IJ, et al. Self-management of patients with advanced cancer: A systematic review of experiences and attitudes. Palliative Medicine. 2020;34(2):160-78.

206. van Houten-Schat MA, Berkhout JJ, van Dijk N, Endedijk MD, Jaarsma ADC, Diemers AD. Self-regulated learning in the clinical context: a systematic review. Med Educ. 2018;52(10):1008-15.

207. Van Wyk NC, Leech R. Becoming the mother of a child with disabilities: a systematic literature review. Community Work & Family. 2016;19(5):554-68.

208. Vatnar SKB, Leer-Salvesen K, Bjorkly S. Mandatory Reporting of Intimate Partner Violence: A Mixed Methods Systematic Review. Trauma Violence & Abuse.

209. Vryonides S, Papastavrou E, Charalambous A, Andreou P, Merkouris A. The ethical dimension of nursing care rationing: A thematic synthesis of qualitative studies. Nursing Ethics. 2015;22(8):881-900.

210. Wangoo L, Ray RA, Ho YH. Compliance and Surgical Team Perceptions of WHO Surgical Safety Checklist; Systematic Review. International Surgery. 2016;101(1-2):35-49.

211. Weetch J, O'Dwyer S, Clare L. The involvement of people with dementia in advocacy: a systematic narrative review. Aging & Mental Health.

212. Wei AC, Urbach DR, Devitt KS, Wiebe M, Bathe OF, McLeod RS, et al. Improving quality through process change: a scoping review of process improvement tools in cancer surgery. BMC Surg. 2014;14:45.

213. Wells M, Williams B, Firnigl D, Lang HD, Coyle J, Kroll T, et al. Supporting 'work-related goals' rather than 'return to work' after cancer? A systematic review and meta-synthesis of 25 qualitative studies. Psycho-Oncology. 2013;22(6):1208-19.

214. Wijma AJ, Bletterman AN, Clark JR, Vervoort S, Beetsma A, Keizer D, et al. Patient-centeredness in physiotherapy: What does it entail? A systematic review of qualitative studies. Physiotherapy Theory and Practice. 2017;33(11):825-40.

215. Williams E, Dingle GA, Clift S. A systematic review of mental health and wellbeing outcomes of group singing for adults with a mental health condition. European Journal of Public Health. 2018;28(6):1035-42.

216. Wilson A, Hillman S, Rosato M, Skelton J, Costello A, Hussein J, et al. A systematic review and thematic synthesis of qualitative studies on maternal emergency transport in low- and middle-income countries. International Journal of Gynecology & Obstetrics. 2013;122(3):192-201.

217. Witzel TC, Lora W, Lees S, Desmond N. Uptake contexts and perceived impacts of HIV testing and counselling among adults in East and Southern Africa: A meta-ethnographic review. PLoS One. 2017;12(2):e0170588.

218. Wong JN, McAuley E, Trinh L. Physical activity programming and counseling preferences among cancer survivors: a systematic review. Int J Behav Nutr Phys Act. 2018;15(1):48.

219. Yang GM, Neo SHS, Lim SZZ, Krishna LKR. Effectiveness of Hospital Palliative Care Teams for Cancer Inpatients: A Systematic Review. Journal of Palliative Medicine. 2016;19(11):1156-65.

220. Ylonen M, Stolt M, Leino-Kilpi H, Suhonen R. Nurses' knowledge about venous leg ulcer care: a literature review. International Nursing Review. 2014;61(2):194-202.

221. Zhang W, Barriball KL, While AE. Nurses' attitudes towards medical devices in healthcare delivery: a systematic review. Journal of Clinical Nursing. 2014;23(19-20):2725-39.

222. Zupančič V, Pahor M, Kogovšek T. Focus Group in Community Mental Health Research: Need for Adaption. Community Ment Health J. 2019;55(1):168-79.

**Included reviews adhering to ENTREQ (n=369)**

1. Hazell CM, Chapman L, Valeix SF, Roberts P, Niven JE, Berry C. Understanding the mental health of doctoral researchers: a mixed methods systematic review with meta-analysis and meta-synthesis. Syst Rev. 2020;9(1):197.

2. Jain SR, Sui Y, Ng CH, Chen ZX, Goh LH, Shorey S. Patients' and healthcare professionals' perspectives towards technology-assisted diabetes self-management education. A qualitative systematic review. PLoS One. 2020;15(8):e0237647.

3. Sion KYJ, Verbeek H, Zwakhalen SMG, Odekerken-Schröder G, Schols J, Hamers JPH. Themes Related to Experienced Quality of Care in Nursing Homes From the Resident's Perspective: A Systematic Literature Review and Thematic Synthesis. Gerontol Geriatr Med. 2020;6:2333721420931964.

4. Cooper K, Russell A, Mandy W, Butler C. The phenomenology of gender dysphoria in adults: A systematic review and meta-synthesis. Clin Psychol Rev. 2020;80:101875.

5. Patel S, Akhtar A, Malins S, Wright N, Rowley E, Young E, et al. The Acceptability and Usability of Digital Health Interventions for Adults With Depression, Anxiety, and Somatoform Disorders: Qualitative Systematic Review and Meta-Synthesis. J Med Internet Res. 2020;22(7):e16228.

6. Damarell RA, Morgan DD, Tieman JJ. General practitioner strategies for managing patients with multimorbidity: a systematic review and thematic synthesis of qualitative research. BMC Fam Pract. 2020;21(1):131.

7. Pritchett RV, Clarke JL, Jolly K, Clarkesmith D, Bem D, Turner GM, et al. Clinicians' views and experiences of prescribing oral anticoagulants for stroke prevention in atrial fibrillation: A qualitative meta-synthesis. PLoS One. 2020;15(5):e0232484.

8. Dattilo AM, Carvalho RS, Feferbaum R, Forsyth S, Zhao A. Hidden Realities of Infant Feeding: Systematic Review of Qualitative Findings from Parents. Behav Sci (Basel). 2020;10(5).

9. Olson RM, García-Moreno C, Colombini M. The implementation and effectiveness of the one stop centre model for intimate partner and sexual violence in low- and middle-income countries: a systematic review of barriers and enablers. BMJ Glob Health. 2020;5(3):e001883.

10. Mathias A, Alves Dos Santos L, Grangeiro A, Couto MT. Thematic synthesis HIV prevention qualitative studies in men who have sex with men (MSM). Colomb Med (Cali). 2019;50(3):201-14.

11. Serrano-Gemes G, Rich-Ruiz M, Serrano-Del-Rosal R. Systematic review of qualitative studies on participants in the decision-making process about the location of care of the elderly. BMJ Open. 2020;10(3):e036551.

12. Firdous T, Darwin Z, Hassan SM. Muslim women's experiences of maternity services in the UK: qualitative systematic review and thematic synthesis. BMC Pregnancy Childbirth. 2020;20(1):115.

13. Craig L, Sims R, Glasziou P, Thomas R. Women's experiences of a diagnosis of gestational diabetes mellitus: a systematic review. BMC Pregnancy Childbirth. 2020;20(1):76.

14. Collingridge Moore D, Payne S, Van den Block L, Ling J, Froggatt K. Strategies for the implementation of palliative care education and organizational interventions in long-term care facilities: A scoping review. Palliat Med. 2020;34(5):558-70.

15. Arias-Colmenero T, Pérez-Morente M, Ramos-Morcillo AJ, Capilla-Díaz C, Ruzafa-Martínez M, Hueso-Montoro C. Experiences and Attitudes of People with HIV/AIDS: A Systematic Review of Qualitative Studies. Int J Environ Res Public Health. 2020;17(2).

16. Wolstenholme H, Kelly C, Hennessy M, Heary C. Childhood fussy/picky eating behaviours: a systematic review and synthesis of qualitative studies. Int J Behav Nutr Phys Act. 2020;17(1):2.

17. Chamberlain C, Ralph N, Hokke S, Clark Y, Gee G, Stansfield C, et al. Healing The Past By Nurturing The Future: A qualitative systematic review and meta-synthesis of pregnancy, birth and early postpartum experiences and views of parents with a history of childhood maltreatment. PLoS One. 2019;14(12):e0225441.

18. Butler J, Gregg L, Calam R, Wittkowski A. Parents' Perceptions and Experiences of Parenting Programmes: A Systematic Review and Metasynthesis of the Qualitative Literature. Clin Child Fam Psychol Rev. 2020;23(2):176-204.

19. Njau B, Covin C, Lisasi E, Damian D, Mushi D, Boulle A, et al. A systematic review of qualitative evidence on factors enabling and deterring uptake of HIV self-testing in Africa. BMC Public Health. 2019;19(1):1289.

20. Tuohy E, Rawdon C, Gallagher P, Glacken M, Murphy N, Swallow V, et al. Children and young people's experiences and perceptions of self-management of type 1 diabetes: A qualitative meta-synthesis. Health Psychol Open. 2019;6(2):2055102919877105.

21. Williams HM, Topping A, Coomarasamy A, Jones LL. Men and Miscarriage: A Systematic Review and Thematic Synthesis. Qual Health Res. 2020;30(1):133-45.

22. Causer H, Muse K, Smith J, Bradley E. What Is the Experience of Practitioners in Health, Education or Social Care Roles Following a Death by Suicide? A Qualitative Research Synthesis. Int J Environ Res Public Health. 2019;16(18).

23. Pluye P, El Sherif R, Granikov V, Hong QN, Vedel I, Galvao MCB, et al. Health outcomes of online consumer health information: A systematic mixed studies review with framework synthesis. J Assoc Inf Sci Technol. 2019;70(7):643-59.

24. Rotevatn TA, Melendez-Torres GJ, Overgaard C, Peven K, Hyldgaard Nilsen J, Bøggild H, et al. Understanding rapid infant weight gain prevention: a systematic review of quantitative and qualitative evidence. Eur J Public Health. 2020;30(4):703-12.

25. Kinnear FJ, Wainwright E, Perry R, Lithander FE, Bayly G, Huntley A, et al. Enablers and barriers to treatment adherence in heterozygous familial hypercholesterolaemia: a qualitative evidence synthesis. BMJ Open. 2019;9(7):e030290.

26. Farre A, Heath G, Shaw K, Bem D, Cummins C. How do stakeholders experience the adoption of electronic prescribing systems in hospitals? A systematic review and thematic synthesis of qualitative studies. BMJ Qual Saf. 2019;28(12):1021-31.

27. Shaw L, Moore D, Nunns M, Thompson Coon J, Ford T, Berry V, et al. Experiences of interventions aiming to improve the mental health and well-being of children and young people with a long-term physical condition: A systematic review and meta-ethnography. Child Care Health Dev. 2019;45(6):832-49.

28. Long H, Brooks JM, Harvie M, Maxwell A, French DP. How do women experience a false-positive test result from breast screening? A systematic review and thematic synthesis of qualitative studies. Br J Cancer. 2019;121(4):351-8.

29. Au M, Anandakumar AD, Preston R, Ray RA, Davis M. A model explaining refugee experiences of the Australian healthcare system: a systematic review of refugee perceptions. BMC Int Health Hum Rights. 2019;19(1):22.

30. Wadsworth KH, Archibald TG, Payne AE, Cleary AK, Haney BL, Hoverman AS. Shared medical appointments and patient-centered experience: a mixed-methods systematic review. BMC Fam Pract. 2019;20(1):97.

31. Dodd M, Ivers R, Zwi AB, Rahman A, Jagnoor J. Investigating the process of evidence-informed health policymaking in Bangladesh: a systematic review. Health Policy Plan. 2019;34(6):469-78.

32. van den Berg MEL, Winsall M, Dyer SM, Breen F, Gresham M, Crotty M. Understanding the Barriers and Enablers to Using Outdoor Spaces in Nursing Homes: A Systematic Review. Gerontologist. 2020;60(4):e254-e69.

33. Grimmond J, Kornhaber R, Visentin D, Cleary M. A qualitative systematic review of experiences and perceptions of youth suicide. PLoS One. 2019;14(6):e0217568.

34. Heinen M, van Oostveen C, Peters J, Vermeulen H, Huis A. An integrative review of leadership competencies and attributes in advanced nursing practice. J Adv Nurs. 2019;75(11):2378-92.

35. Sibeoni J, Bellon-Champel L, Mousty A, Manolios E, Verneuil L, Revah-Levy A. Physicians' Perspectives About Burnout: a Systematic Review and Metasynthesis. J Gen Intern Med. 2019;34(8):1578-90.

36. Hudson BF, Best S, Stone P, Noble TB. Impact of informational and relational continuity for people with palliative care needs: a mixed methods rapid review. BMJ Open. 2019;9(5):e027323.

37. Dibble KE, Maksut JL, Siembida EJ, Hutchison M, Bellizzi KM. A Systematic Literature Review of HPV Vaccination Barriers Among Adolescent and Young Adult Males. J Adolesc Young Adult Oncol. 2019;8(5):495-511.

38. Abbott R, Orr N, McGill P, Whear R, Bethel A, Garside R, et al. How do "robopets" impact the health and well-being of residents in care homes? A systematic review of qualitative and quantitative evidence. Int J Older People Nurs. 2019;14(3):e12239.

39. Armstrong M, Flemming K, Kupeli N, Stone P, Wilkinson S, Candy B. Aromatherapy, massage and reflexology: A systematic review and thematic synthesis of the perspectives from people with palliative care needs. Palliat Med. 2019;33(7):757-69.

40. Bazzano AN, Felker-Kantor E, Eragoda S, Kaji A, Horlick R. Parent and family perspectives on home-based newborn care practices in lower-income countries: a systematic review of qualitative studies. BMJ Open. 2019;9(4):e025471.

41. Tarver WL, Haggstrom DA. The Use of Cancer-Specific Patient-Centered Technologies Among Underserved Populations in the United States: Systematic Review. J Med Internet Res. 2019;21(4):e10256.

42. Finnegan S, Bruce J, Seers K. What enables older people to continue with their falls prevention exercises? A qualitative systematic review. BMJ Open. 2019;9(4):e026074.

43. Manning M, MacFarlane A, Hickey A, Franklin S. Perspectives of people with aphasia post-stroke towards personal recovery and living successfully: A systematic review and thematic synthesis. PLoS One. 2019;14(3):e0214200.

44. Zhu Z, Xing W, Lizarondo L, Guo M, Hu Y. Nursing students' experiences with faculty incivility in the clinical education context: a qualitative systematic review and meta-synthesis. BMJ Open. 2019;9(2):e024383.

45. Kannan A, Kirkman M, Ruseckaite R, Evans SM. Prostate care and prostate cancer from the perspectives of undiagnosed men: a systematic review of qualitative research. BMJ Open. 2019;9(1):e022842.

46. Hughes NM, Noyes J, Eckley L, Pritchard T. What do patients and family-caregivers value from hospice care? A systematic mixed studies review. BMC Palliat Care. 2019;18(1):18.

47. Göstemeyer G, Baker SR, Schwendicke F. Barriers and facilitators for provision of oral health care in dependent older people: a systematic review. Clin Oral Investig. 2019;23(3):979-93.

48. Behzadifar M, Bragazzi NL, Arab-Zozani M, Bakhtiari A, Behzadifar M, Beyranvand T, et al. The challenges of implementation of clinical governance in Iran: a meta-synthesis of qualitative studies. Health Res Policy Syst. 2019;17(1):3.

49. Aluri J, Moran D, Kironji AG, Carroll B, Cox J, Chen CCG, et al. The ethical experiences of trainees on short-term international trips: a systematic qualitative synthesis. BMC Med Educ. 2018;18(1):324.

50. Carter DD, Robinson K, Forbes J, Hayes S. Experiences of mobile health in promoting physical activity: A qualitative systematic review and meta-ethnography. PLoS One. 2018;13(12):e0208759.

51. Long Q, Kingdon C, Yang F, Renecle MD, Jahanfar S, Bohren MA, et al. Prevalence of and reasons for women's, family members', and health professionals' preferences for cesarean section in China: A mixed-methods systematic review. PLoS Med. 2018;15(10):e1002672.

52. Lim RH, Sharmeen T. Medicines management issues in dementia and coping strategies used by people living with dementia and family carers: A systematic review. Int J Geriatr Psychiatry. 2018;33(12):1562-81.

53. Tan WS, Beatty L, Koczwara B. Do cancer patients use the term resilience? A systematic review of qualitative studies. Support Care Cancer. 2019;27(1):43-56.

54. Roberti J, Cummings A, Myall M, Harvey J, Lippiett K, Hunt K, et al. Work of being an adult patient with chronic kidney disease: a systematic review of qualitative studies. BMJ Open. 2018;8(9):e023507.

55. Mechta Nielsen T, Frøjk Juhl M, Feldt-Rasmussen B, Thomsen T. Adherence to medication in patients with chronic kidney disease: a systematic review of qualitative research. Clin Kidney J. 2018;11(4):513-27.

56. Somes E, Dukes J, Brungardt A, Jordan S, DeSanto K, Jones CD, et al. Perceptions of trained laypersons in end-of-life or advance care planning conversations: a qualitative meta-synthesis. BMC Palliat Care. 2018;17(1):98.

57. Seaman AT, Steffen M, Doo T, Healy HS, Solimeo SL. Metasynthesis of Patient Attitudes Toward Bone Densitometry. J Gen Intern Med. 2018;33(10):1796-804.

58. Lazzerini M, Ciuch M, Rusconi S, Covi B. Facilitators and barriers to the effective implementation of the individual maternal near-miss case reviews in low/middle-income countries: a systematic review of qualitative studies. BMJ Open. 2018;8(6):e021281.

59. Schloemer T, Schröder-Bäck P. Criteria for evaluating transferability of health interventions: a systematic review and thematic synthesis. Implement Sci. 2018;13(1):88.

60. Germeni E, Frost J, Garside R, Rogers M, Valderas JM, Britten N. Antibiotic prescribing for acute respiratory tract infections in primary care: an updated and expanded meta-ethnography. Br J Gen Pract. 2018;68(674):e633-e45.

61. Jennings AA, Foley T, Walsh KA, Coffey A, Browne JP, Bradley CP. General practitioners' knowledge, attitudes, and experiences of managing behavioural and psychological symptoms of dementia: A mixed-methods systematic review. Int J Geriatr Psychiatry. 2018;33(9):1163-76.

62. Robinson H, Williams V, Curtis F, Bridle C, Jones AW. Facilitators and barriers to physical activity following pulmonary rehabilitation in COPD: a systematic review of qualitative studies. NPJ Prim Care Respir Med. 2018;28(1):19.

63. Neelakantan L, Hetrick S, Michelson D. Users' experiences of trauma-focused cognitive behavioural therapy for children and adolescents: a systematic review and metasynthesis of qualitative research. Eur Child Adolesc Psychiatry. 2019;28(7):877-97.

64. Ellis K, Mulnier H, Forbes A. Perceptions of insulin use in type 2 diabetes in primary care: a thematic synthesis. BMC Fam Pract. 2018;19(1):70.

65. Fogarty S, Elmir R, Hay P, Schmied V. The experience of women with an eating disorder in the perinatal period: a meta-ethnographic study. BMC Pregnancy Childbirth. 2018;18(1):121.

66. Graham-Clarke E, Rushton A, Noblet T, Marriott J. Facilitators and barriers to non-medical prescribing - A systematic review and thematic synthesis. PLoS One. 2018;13(4):e0196471.

67. Slade SC, Philip K, Morris ME. Frameworks for embedding a research culture in allied health practice: a rapid review. Health Res Policy Syst. 2018;16(1):29.

68. Drew S, Gooberman-Hill R, Lavy C. What factors impact on the implementation of clubfoot treatment services in low and middle-income countries?: a narrative synthesis of existing qualitative studies. BMC Musculoskelet Disord. 2018;19(1):72.

69. Walker LE, Nestler DM, Laack TA, Clements CM, Erwin PJ, Scanlan-Hanson L, et al. Clinical care review systems in healthcare: a systematic review. Int J Emerg Med. 2018;11(1):6.

70. Boehmer KR, Abu Dabrh AM, Gionfriddo MR, Erwin P, Montori VM. Does the chronic care model meet the emerging needs of people living with multimorbidity? A systematic review and thematic synthesis. PLoS One. 2018;13(2):e0190852.

71. Suthar AB, Allen LG, Cifuentes S, Dye C, Nagata JM. Lessons learnt from implementation of the International Health Regulations: a systematic review. Bull World Health Organ. 2018;96(2):110-21e.

72. Morgan JE, Cleminson J, Stewart LA, Phillips RS, Atkin K. Meta-ethnography of experiences of early discharge, with a focus on paediatric febrile neutropenia. Support Care Cancer. 2018;26(4):1039-50.

73. Shea EO, Timmons S, Shea EO, Fox S, Irving K. Key stakeholders' experiences of respite services for people with dementia and their perspectives on respite service development: a qualitative systematic review. BMC Geriatr. 2017;17(1):282.

74. Soofi AY, Bello-Haas VD, Kho ME, Letts L. The impact of rehabilitative interventions on quality of life: a qualitative evidence synthesis of personal experiences of individuals with amyotrophic lateral sclerosis. Qual Life Res. 2018;27(4):845-56.

75. Broad KL, Sandhu VK, Sunderji N, Charach A. Youth experiences of transition from child mental health services to adult mental health services: a qualitative thematic synthesis. BMC Psychiatry. 2017;17(1):380.

76. Stomski NJ, Morrison P. Participation in mental healthcare: a qualitative meta-synthesis. Int J Ment Health Syst. 2017;11:67.

77. Bazzano AN, Kaji A, Felker-Kantor E, Bazzano LA, Potts KS. Qualitative Studies of Infant and Young Child Feeding in Lower-Income Countries: A Systematic Review and Synthesis of Dietary Patterns. Nutrients. 2017;9(10).

78. McTavish JR, Kimber M, Devries K, Colombini M, MacGregor JCD, Wathen CN, et al. Mandated reporters' experiences with reporting child maltreatment: a meta-synthesis of qualitative studies. BMJ Open. 2017;7(10):e013942.

79. Hossain LN, Fernandez-Llimos F, Luckett T, Moullin JC, Durks D, Franco-Trigo L, et al. Qualitative meta-synthesis of barriers and facilitators that influence the implementation of community pharmacy services: perspectives of patients, nurses and general medical practitioners. BMJ Open. 2017;7(9):e015471.

80. Robertshaw L, Dhesi S, Jones LL. Challenges and facilitators for health professionals providing primary healthcare for refugees and asylum seekers in high-income countries: a systematic review and thematic synthesis of qualitative research. BMJ Open. 2017;7(8):e015981.

81. Booker MJ, Purdy S, Shaw ARG. Seeking ambulance treatment for 'primary care' problems: a qualitative systematic review of patient, carer and professional perspectives. BMJ Open. 2017;7(8):e016832.

82. Guise A, Horyniak D, Melo J, McNeil R, Werb D. The experience of initiating injection drug use and its social context: a qualitative systematic review and thematic synthesis. Addiction. 2017;112(12):2098-111.

83. Golder S, Ahmed S, Norman G, Booth A. Attitudes Toward the Ethics of Research Using Social Media: A Systematic Review. J Med Internet Res. 2017;19(6):e195.

84. Schwarz B, Claros-Salinas D, Streibelt M. Meta-Synthesis of Qualitative Research on Facilitators and Barriers of Return to Work After Stroke. J Occup Rehabil. 2018;28(1):28-44.

85. Dowsett LE, Coward S, Lorenzetti DL, MacKean G, Clement F. Living with Hepatitis C Virus: A Systematic Review and Narrative Synthesis of Qualitative Literature. Can J Gastroenterol Hepatol. 2017;2017:3268650.

86. Coxon K, Chisholm A, Malouf R, Rowe R, Hollowell J. What influences birth place preferences, choices and decision-making amongst healthy women with straightforward pregnancies in the UK? A qualitative evidence synthesis using a 'best fit' framework approach. BMC Pregnancy Childbirth. 2017;17(1):103.

87. Tucker JD, Tso LS, Hall B, Ma Q, Beanland R, Best J, et al. Enhancing Public Health HIV Interventions: A Qualitative Meta-Synthesis and Systematic Review of Studies to Improve Linkage to Care, Adherence, and Retention. EBioMedicine. 2017;17:163-71.

88. Cox A, Lucas G, Marcu A, Piano M, Grosvenor W, Mold F, et al. Cancer Survivors' Experience With Telehealth: A Systematic Review and Thematic Synthesis. J Med Internet Res. 2017;19(1):e11.

89. Morton K, Dennison L, May C, Murray E, Little P, McManus RJ, et al. Using digital interventions for self-management of chronic physical health conditions: A meta-ethnography review of published studies. Patient Educ Couns. 2017;100(4):616-35.

90. Forster AS, Rockliffe L, Chorley AJ, Marlow LA, Bedford H, Smith SG, et al. A qualitative systematic review of factors influencing parents' vaccination decision-making in the United Kingdom. SSM Popul Health. 2016;2:603-12.

91. Hudson BF, Flemming K, Shulman C, Candy B. Challenges to access and provision of palliative care for people who are homeless: a systematic review of qualitative research. BMC Palliat Care. 2016;15(1):96.

92. Young I, Waddell L. Barriers and Facilitators to Safe Food Handling among Consumers: A Systematic Review and Thematic Synthesis of Qualitative Research Studies. PLoS One. 2016;11(12):e0167695.

93. Morgan H. 'Pushed' self-tracking using digital technologies for chronic health condition management: a critical interpretive synthesis. Digit Health. 2016;2:2055207616678498.

94. Coffey L, Mooney O, Dunne S, Sharp L, Timmons A, Desmond D, et al. Cancer survivors' perspectives on adjustment-focused self-management interventions: a qualitative meta-synthesis. J Cancer Surviv. 2016;10(6):1012-34.

95. Ma Q, Tso LS, Rich ZC, Hall BJ, Beanland R, Li H, et al. Barriers and facilitators of interventions for improving antiretroviral therapy adherence: a systematic review of global qualitative evidence. J Int AIDS Soc. 2016;19(1):21166.

96. Li H, Marley G, Ma W, Wei C, Lackey M, Ma Q, et al. The Role of ARV Associated Adverse Drug Reactions in Influencing Adherence Among HIV-Infected Individuals: A Systematic Review and Qualitative Meta-Synthesis. AIDS Behav. 2017;21(2):341-51.

97. Boehmer KR, Gionfriddo MR, Rodriguez-Gutierrez R, Dabrh AM, Leppin AL, Hargraves I, et al. Patient capacity and constraints in the experience of chronic disease: a qualitative systematic review and thematic synthesis. BMC Fam Pract. 2016;17(1):127.

98. Plant SE, Tyson SF, Kirk S, Parsons J. What are the barriers and facilitators to goal-setting during rehabilitation for stroke and other acquired brain injuries? A systematic review and meta-synthesis. Clin Rehabil. 2016;30(9):921-30.

99. Flores D, Leblanc N, Barroso J. Enroling and retaining human immunodeficiency virus (HIV) patients in their care: A metasynthesis of qualitative studies. Int J Nurs Stud. 2016;62:126-36.

100. Kågesten A, Gibbs S, Blum RW, Moreau C, Chandra-Mouli V, Herbert A, et al. Understanding Factors that Shape Gender Attitudes in Early Adolescence Globally: A Mixed-Methods Systematic Review. PLoS One. 2016;11(6):e0157805.

101. Reid C, Seymour J, Jones C. A Thematic Synthesis of the Experiences of Adults Living with Hemodialysis. Clin J Am Soc Nephrol. 2016;11(7):1206-18.

102. Chorley AJ, Marlow LA, Forster AS, Haddrell JB, Waller J. Experiences of cervical screening and barriers to participation in the context of an organised programme: a systematic review and thematic synthesis. Psychooncology. 2017;26(2):161-72.

103. Flemming K, Graham H, McCaughan D, Angus K, Sinclair L, Bauld L. Health professionals' perceptions of the barriers and facilitators to providing smoking cessation advice to women in pregnancy and during the post-partum period: a systematic review of qualitative research. BMC Public Health. 2016;16:290.

104. Passey ME, Longman JM, Robinson J, Wiggers J, Jones LL. Smoke-free homes: what are the barriers, motivators and enablers? A qualitative systematic review and thematic synthesis. BMJ Open. 2016;6(3):e010260.

105. Surmon L, Bialocerkowski A, Hu W. Perceptions of preparedness for the first medical clerkship: a systematic review and synthesis. BMC Med Educ. 2016;16:89.

106. Tsimicalis A, Denis-Larocque G, Michalovic A, Lepage C, Williams K, Yao TR, et al. The psychosocial experience of individuals living with osteogenesis imperfecta: a mixed-methods systematic review. Qual Life Res. 2016;25(8):1877-96.

107. Sampson R, Cooper J, Barbour R, Polson R, Wilson P. Patients' perspectives on the medical primary-secondary care interface: systematic review and synthesis of qualitative research. BMJ Open. 2015;5(10):e008708.

108. Flemming K, Graham H, McCaughan D, Angus K, Bauld L. The barriers and facilitators to smoking cessation experienced by women's partners during pregnancy and the post-partum period: a systematic review of qualitative research. BMC Public Health. 2015;15:849.

109. Koshoedo SA, Paul-Ebhohimhen VA, Jepson RG, Watson MC. Understanding the complex interplay of barriers to physical activity amongst black and minority ethnic groups in the United Kingdom: a qualitative synthesis using meta-ethnography. BMC Public Health. 2015;15:643.

110. Bohren MA, Vogel JP, Hunter EC, Lutsiv O, Makh SK, Souza JP, et al. The Mistreatment of Women during Childbirth in Health Facilities Globally: A Mixed-Methods Systematic Review. PLoS Med. 2015;12(6):e1001847; discussion e.

111. Taylor N, Clay-Williams R, Hogden E, Braithwaite J, Groene O. High performing hospitals: a qualitative systematic review of associated factors and practical strategies for improvement. BMC Health Serv Res. 2015;15:244.

112. Farrer L, Marinetti C, Cavaco YK, Costongs C. Advocacy for health equity: a synthesis review. Milbank Q. 2015;93(2):392-437.

113. Lachal J, Orri M, Sibeoni J, Moro MR, Revah-Levy A. Metasynthesis of youth suicidal behaviours: perspectives of youth, parents, and health care professionals. PLoS One. 2015;10(5):e0127359.

114. Hussain JA, Flemming K, Murtagh FE, Johnson MJ. Patient and health care professional decision-making to commence and withdraw from renal dialysis: a systematic review of qualitative research. Clin J Am Soc Nephrol. 2015;10(7):1201-15.

115. Fleming A, Bradley C, Cullinan S, Byrne S. Antibiotic prescribing in long-term care facilities: a meta-synthesis of qualitative research. Drugs Aging. 2015;32(4):295-303.

116. Lucas PJ, Cabral C, Hay AD, Horwood J. A systematic review of parent and clinician views and perceptions that influence prescribing decisions in relation to acute childhood infections in primary care. Scand J Prim Health Care. 2015;33(1):11-20.

117. Twyman L, Bonevski B, Paul C, Bryant J. Perceived barriers to smoking cessation in selected vulnerable groups: a systematic review of the qualitative and quantitative literature. BMJ Open. 2014;4(12):e006414.

118. Bohren MA, Hunter EC, Munthe-Kaas HM, Souza JP, Vogel JP, Gülmezoglu AM. Facilitators and barriers to facility-based delivery in low- and middle-income countries: a qualitative evidence synthesis. Reprod Health. 2014;11(1):71.

119. Lins S, Hayder-Beichel D, Rücker G, Motschall E, Antes G, Meyer G, et al. Efficacy and experiences of telephone counselling for informal carers of people with dementia. Cochrane Database Syst Rev. 2014;2014(9):Cd009126.

120. Frost J, Garside R, Cooper C, Britten N. A qualitative synthesis of diabetes self-management strategies for long term medical outcomes and quality of life in the UK. BMC Health Serv Res. 2014;14:348.

121. Loudon K, Santesso N, Callaghan M, Thornton J, Harbour J, Graham K, et al. Patient and public attitudes to and awareness of clinical practice guidelines: a systematic review with thematic and narrative syntheses. BMC Health Serv Res. 2014;14:321.

122. Mavhu W, Mupambireyi Z, Hart G, Cowan FM. Factors associated with parental non-adoption of infant male circumcision for HIV prevention in Sub-Saharan Africa: a systematic review and thematic synthesis. AIDS Behav. 2014;18(9):1776-84.

123. Cullinan S, O'Mahony D, Fleming A, Byrne S. A meta-synthesis of potentially inappropriate prescribing in older patients. Drugs Aging. 2014;31(8):631-8.

124. Teasdale E, Santer M, Geraghty AW, Little P, Yardley L. Public perceptions of non-pharmaceutical interventions for reducing transmission of respiratory infection: systematic review and synthesis of qualitative studies. BMC Public Health. 2014;14:589.

125. Sinnott C, Mc Hugh S, Browne J, Bradley C. GPs' perspectives on the management of patients with multimorbidity: systematic review and synthesis of qualitative research. BMJ Open. 2013;3(9):e003610.

126. McGeough C, Walsh A, Clyne B. Barriers and facilitators perceived by women while homeless and pregnant in accessing antenatal and or postnatal healthcare: A qualitative evidence synthesis. Health & Social Care in the Community. 2020;28(5):1380-93.

127. Neris RR, Nascimento LC, Leite A, Alvarenga WD, Polita NB, Zago MMF. The experience of health-related quality of life in extended and permanent cancer survivors: A qualitative systematic review. Psycho-Oncology.

128. Weisbeck SJ, Bright KS, Ginn CS, Smith JM, Hayden KA, Ringham C. Perceptions about cannabis use during pregnancy: a rapid best-framework qualitative synthesis. Canadian Journal of Public Health-Revue Canadienne De Sante Publique.

129. McCarron TL, MacKean G, Dowsett LE, Saini M, Clement F. Patients' experience with and perspectives on neuromodulation for pain: a systematic review of the qualitative research literature. Pain. 2020;161(8):1708-15.

130. Polita NB, de Montigny F, Neris RR, Alvarenga WD, Silva-Rodrigues FM, Leite A, et al. The Experiences of Bereaved Parents After the Loss of a Child to Cancer: A Qualitative Metasynthesis. Journal of Pediatric Oncology Nursing.

131. Corbett T, Cummings A, Calman L, Farrington N, Fenerty V, Foster C, et al. Self-management in older people living with cancer and multi-morbidity: A systematic review and synthesis of qualitative studies. Psycho-Oncology.

132. Erdos T, de Haan E, Heusinkveld S. Coaching: client factors & contextual dynamics in the change process A qualitative meta-synthesis. Coaching-an International Journal of Theory Research and Practice.

133. MacGregor JCD, Naeemzadah N, Oliver CL, Javan T, MacQuarrie BJ, Wathen CN. Women's Experiences of the Intersections of Work and Intimate Partner Violence: A Review of Qualitative Research. Trauma Violence & Abuse.

134. Woods TJ, Windt JM, Carter O. Silence in Shamatha, Transcendental, and Stillness Meditation: An Evidence Synthesis Based on Expert Texts. Frontiers in Psychology. 2020;11.

135. Scott J, Weatherhead S, Daker-White G, Manthorpe J, Mawson M. Practitioners' experiences of the mental capacity act: a systematic review. Journal of Adult Protection.

136. Barisone M, Bagnasco A, Hayter M, Rossi S, Aleo G, Zanini M, et al. Dermatological diseases, sexuality and intimate relationships: A qualitative meta-synthesis. Journal of Clinical Nursing. 2020;29(17-18):3136-53.

137. Conti A, Clari M, Kangasniemi M, Martin B, Borraccino A, Campagna S. What self-care behaviours are essential for people with spinal cord injury? A systematic review and meta-synthesis. Disability and Rehabilitation.

138. Lordon RJ, Mikles SP, Kneale L, Evans HL, Munson SA, Backonja U, et al. How patient-generated health data and patient-reported outcomes affect patient-clinician relationships: A systematic review. Health Informatics Journal.

139. Hanrahan V, Gillies K, Biesty L. Recruiters' perspectives of recruiting women during pregnancy and childbirth to clinical trials: A qualitative evidence synthesis. Plos One. 2020;15(6).

140. Bressan V, Visintini C, Palese A. What do family caregivers of people with dementia need? A mixed-method systematic review. Health & Social Care in the Community.

141. Holopainen R, Simpson P, Piirainen A, Karppinen J, Schutze R, Smith A, et al. Physiotherapists' perceptions of learning and implementing a biopsychosocial intervention to treat musculoskeletal pain conditions: a systematic review and metasynthesis of qualitative studies. Pain. 2020;161(6):1150-68.

142. O'Connor M, O'Donovan B, Waller J, Ceilleachair AO, Gallagher P, Martin C, et al. The role of healthcare professionals in HPV communication with head and neck cancer patients: A narrative synthesis of qualitative studies. European Journal of Cancer Care. 2020;29(4).

143. Bartley N, Napier C, Best M, Butow P. Patient experience of uncertainty in cancer genomics: a systematic review. Genetics in Medicine.

144. Alsaggaf F, Coyne I. A systematic review of the impact of chronic pain on adolescents' school functioning and school personnel responses to managing pain in the schools. Journal of Advanced Nursing.

145. Foong HF, Kyaw BM, Upton Z, Car LT. Facilitators and barriers of using digital technology for the management of diabetic foot ulcers: A qualitative systematic review. International Wound Journal.

146. Bechange S, Jolley E, Virendrakumar B, Pente V, Milgate J, Schmidt E. Strengths and weaknesses of eye care services in sub-Saharan Africa: a meta-synthesis of eye health system assessments. Bmc Health Services Research. 2020;20(1).

147. Skorka K, McBryde C, Copley J, Meredith PJ, Reid N. Experiences of Children with Fetal Alcohol Spectrum Disorder and Their Families: A Critical Review. Alcoholism-Clinical and Experimental Research. 2020;44(6):1175-88.

148. Zhu Z, Xing WJ, Zhang XJ, Hu Y, So WKW. Cancer survivors' experiences with financial toxicity: A systematic review and meta-synthesis of qualitative studies. Psycho-Oncology. 2020;29(6):945-59.

149. Fee A, McIlfatrick S, Ryan A. Examining the support needs of older male spousal caregivers of people with a long-term condition: A systematic review of the literature. International Journal of Older People Nursing.

150. Sweeney-Magee M, Moustaqim-Barrette A, Gotay C, Dummer T. A systematic mixed studies review of health behaviour change interventions in colorectal cancer survivors. Journal of Advanced Nursing.

151. Tang MT, Liu XL, Wu Q, Shi Y. The Lived Experience of Work-Related Issues Among Oncology Nurses: A Metasynthesis. Cancer Nursing. 2020;43(3):222-37.

152. Martin B, Jones J, Miller M, Johnson-Koenke R. Health Care Professionals' Perceptions of Pay-for-Performance in Practice: A Qualitative Metasynthesis. Inquiry-the Journal of Health Care Organization Provision and Financing. 2020;57.

153. Phelps EE, Tutton E, Griffin X, Baird J. A mixed-methods systematic review of patients' experience of being invited to participate in surgical randomised controlled trials. Social Science & Medicine. 2020;253.

154. Mughal A, Evans C. Views and experiences of nurses in providing end-of-life care to patients in an ED context: a qualitative systematic review. Emergency Medicine Journal. 2020;37(5):265-72.

155. Tanganhito DD, Bick D, Chang YS. Breastfeeding experiences and perspectives among women with postnatal depression: A qualitative evidence synthesis. Women and Birth. 2020;33(3):231-9.

156. Karim N, Boyle B, Lohan M, Kerr C. Immigrant parents' experiences of accessing child healthcare services in a host country: A qualitative thematic synthesis. Journal of Advanced Nursing. 2020;76(7):1509-19.

157. Danielis M, Povoli A, Mattiussi E, Palese A. Understanding patients' experiences of being mechanically ventilated in the Intensive Care Unit: Findings from a meta-synthesis and meta-summary. Journal of Clinical Nursing. 2020;29(13-14):2107-24.

158. Dash K, Goyder EC, Quirk H. A qualitative synthesis of the perceived factors that affect participation in physical activity among children and adolescents with type 1 diabetes. Diabetic Medicine. 2020;37(6):934-44.

159. Flemming K, Turner V, Bolsher S, Hulme B, McHugh E, Watt I. The experiences of, and need for, palliative care for people with motor neurone disease and their informal caregivers: A qualitative systematic review. Palliative Medicine. 2020;34(6):708-30.

160. Cleary M, West S, Hunt GE, McLean L, Kornhaber R. A Qualitative Systematic Review of Caregivers' Experiences of Caring for Family Diagnosed with Schizophrenia. Issues in Mental Health Nursing. 2020;41(8):667-83.

161. Graham H, de Bell S, Flemming K, Sowden A, White P, Wright K. Older people's experiences of everyday travel in the urban environment: a thematic synthesis of qualitative studies in the United Kingdom. Ageing & Society. 2020;40(4):842-68.

162. Cooley SJ, Jones CR, Kurtz A, Robertson N. 'Into the Wild': A meta-synthesis of talking therapy in natural outdoor spaces. Clinical Psychology Review. 2020;77.

163. Onozato T, Cruz CFD, Farre A, Silvestre CC, Silva RDS, Santos GA, et al. Factors influencing the implementation of clinical pharmacy services for hospitalized patients: A mixed-methods systematic review. Research in Social & Administrative Pharmacy. 2020;16(4):437-49.

164. Brown A, O'Connor S. Mobile health applications for people with dementia: a systematic review and synthesis of qualitative studies. Informatics for Health & Social Care.

165. Rodriguez-Garrido P, Pino-Moran JA, Goberna-Tricas J. Exploring social and health care representations about home birth: An Integrative Literature Review. Public Health Nursing. 2020;37(3):422-38.

166. Wells K, Hancock N, Honey A. The experience of living after ECT: a qualitative meta-synthesis. Journal of Mental Health.

167. Rosenwohl-Mack A, Schumacher K, Fang ML, Fukuoka Y. A new conceptual model of experiences of aging in place in the United States: Results of a systematic review and meta-ethnography of qualitative studies. International Journal of Nursing Studies. 2020;103.

168. Rada MP, Jones S, Falconi G, Haddad JM, Betschart C, Pergialiotis V, et al. A systematic review and meta-synthesis of qualitative studies on pelvic organ prolapse for the development of core outcome sets. Neurourology and Urodynamics. 2020;39(3):880-9.

169. Ortega DG, Papathanassoglou E, Norris CM. The lived experience of delirium in intensive care unit patients: A meta-ethnography. Australian Critical Care. 2020;33(2):193-202.

170. Shorey S, Chan VL. Paternal mental health during the perinatal period: A qualitative systematic review. Journal of Advanced Nursing. 2020;76(6):1307-19.

171. Nevin M, Hynes G, Smith V. Healthcare providers' views and experiences of non-specialist palliative care in hospitals: A qualitative systematic review and thematic synthesis. Palliative Medicine. 2020;34(5):605-18.

172. Gibson JME, Miller C, Coupe J, Jones SP. Medication-taking after stroke: a qualitative meta-synthesis of the perspectives of stroke survivors, informal carers and health professionals. Family Practice. 2020;37(1):4-14.

173. Clancy C, Lynch J, Oconnor P, Dowling M. Breast cancer patients' experiences of adherence and persistence to oral endocrine therapy: A qualitative evidence synthesis. European Journal of Oncology Nursing. 2020;44.

174. Karadzhov D, Yuan YQ, Bond L. Coping amidst an assemblage of disadvantage: A qualitative metasynthesis of first-person accounts of managing severe mental illness while homeless. Journal of Psychiatric and Mental Health Nursing. 2020;27(1):4-24.

175. Naidoo N, Nguyen VT, Ravaud P, Young B, Amiel P, Schante D, et al. The research burden of randomized controlled trial participation: a systematic thematic synthesis of qualitative evidence. Bmc Medicine. 2020;18(1).

176. Tynewydd IM, North S, Rushworth I. The Experiences of Mental Health Professionals Supporting Forced Migrants: A Qualitative Systematic Review. Refuge. 2020;36(1):50-65.

177. Hernon O, Dalton R, Dowling M. Clinical research nurses' expectations and realities of their role: A qualitative evidence synthesis. Journal of Clinical Nursing. 2020;29(5-6):667-83.

178. Nowell L, Ovic G, Kenny N, Hayden KA, Jacobsen M. Professional learning and development initiatives for postdoctoral scholars. Studies in Graduate and Postdoctoral Education.

179. Ananthakumar T, Jones NR, Hinton L, Aveyard P. Clinical encounters about obesity: Systematic review of patients' perspectives. Clinical Obesity. 2020;10(1).

180. Nevedal AL, Ayalon L, Briller SH. A Qualitative Evidence Synthesis Review of Longitudinal Qualitative Research in Gerontology. Gerontologist. 2019;59(6):E791-E801.

181. Leite A, Garcia-Vivar C, Neris RR, Alvarenga WD, Nascimento LC. The experience of hope in families of children and adolescents living with chronic illness: A thematic synthesis of qualitative studies(sic)(sic)(sic)(sic)(sic)(sic)(sic)(sic)(sic)(sic)<SIC>(sic)(sic) (sic)(sic)(sic):(sic)(sic)(sic)(sic)(sic)(sic)(sic)(sic)(sic)(sic). Journal of Advanced Nursing. 2019;75(12):3246-62.

182. Davenport K, Hardy G, Tai S, Mansell W. Individual experiences of psychological-based interventions for bipolar disorder: A systematic review and thematic synthesis. Psychology and Psychotherapy-Theory Research and Practice. 2019;92(4):499-522.

183. Kokorelias KM, Lu FKT, Santos JR, Xu Y, Leung R, Cameron JI. "Caregiving is a full-time job" impacting stroke caregivers' health and well-being: A qualitative meta-synthesis. Health & Social Care in the Community. 2020;28(2):325-40.

184. Mentrup S, Harris E, Gomersall T, Kopke S, Astin F. Patients' Experiences of Cardiovascular Health Education and Risk Communication: A Qualitative Synthesis. Qualitative Health Research. 2020;30(1):88-104.

185. Xu H, Zhang XJ, Wang DQ, Xu L, Wang AP. Factors influencing medication-taking behaviour with adjuvant endocrine therapy in women with breast cancer: A qualitative systematic review. Journal of Advanced Nursing. 2020;76(2):445-58.

186. Capon H, O'Shea M, McIver S. Yoga and mental health: A synthesis of qualitative findings. Complementary Therapies in Clinical Practice. 2019;37:122-32.

187. Daley C, Al-Abdulmunem M, Holden RJ. Knowledge among patients with heart failure: A narrative synthesis of qualitative research. Heart & Lung. 2019;49(6):477-85.

188. Brooks LA, Bloomer MJ, Manias E. Culturally sensitive communication at the end-of-life in the intensive care unit: A systematic review. Australian Critical Care. 2019;32(6):516-23.

189. Coffey A, Saab MM, Landers M, Cornally N, Hegarty J, Drennan J, et al. The impact of compassionate care education on nurses: A mixed-method systematic review. Journal of Advanced Nursing. 2019;75(11):2340-51.

190. Olsen CF, Bergland A, Debesay J, Bye A, Langaas AG. Striking a balance: Health care providers' experiences with home-based, patient-centered care for older people-A meta-synthesis of qualitative studies. Patient Education and Counseling. 2019;102(11):1991-2000.

191. Ryan S, Ward M, Vaughan D, Murray B, Zena M, O'Connor T, et al. Do safety briefings improve patient safety in the acute hospital setting? A systematic review. Journal of Advanced Nursing. 2019;75(10):2085-98.

192. Crilly G, Dowling M, Delaunois I, Flavin M, Biesty L. Critical care nurses' experiences of providing care for adults in a highly technological environment: A qualitative evidence synthesis. Journal of Clinical Nursing. 2019;28(23-24):4250-63.

193. Murray CM, Van Kessel G, Guerin M, Hillier S, Stanley M. Exercising Choice and Control: A Qualitative Meta-synthesis of Perspectives of People With a Spinal Cord Injury. Archives of Physical Medicine and Rehabilitation. 2019;100(9):1752-62.

194. Bressan V, Cadorin L, Stevanin S, Palese A. Patients experiences of bedside handover: findings from a meta-synthesis. Scandinavian Journal of Caring Sciences. 2019;33(3):556-68.

195. Inga-Britt L, Kerstin B. Challenges faced in daily life by persons with type 2 diabetes: A meta-synthesis. Chronic Illness. 2019;15(3):167-86.

196. O'Leary N, Salmon N, Clifford A, O'Donoghue M, Reeves S. 'Bumping along': a qualitative metasynthesis of challenges to interprofessional placements. Medical Education. 2019;53(9):903-15.

197. O'Connell S, McCarthy VJC, Savage E. Self-management support preferences of people with asthma or chronic obstructive pulmonary disease: A systematic review and meta-synthesis of qualitative studies. Chronic Illness.

198. Allison R, Flemming K. Mental health patients' experiences of softer coercion and its effects on their interactions with practitioners: A qualitative evidence synthesis. Journal of Advanced Nursing. 2019;75(11):2274-84.

199. Barken TL, Soderhamn U, Thygesen E. A sense of belonging: A meta-ethnography of the experience of patients with chronic obstructive pulmonary disease receiving care through telemedicine. Journal of Advanced Nursing. 2019;75(12):3219-30.

200. Page MJ, O'Connor DA, Malek M, Haas R, Beaton D, Huang HM, et al. Patients' experience of shoulder disorders: a systematic review of qualitative studies for the OMERACT Shoulder Core Domain Set. Rheumatology. 2019;58(8):1410-21.

201. Danet AD, Cardoso PMJ. Emotional experiences of health professionals in organ procurement and transplantation. A systematic review. Cirugia Espanola. 2019;97(7):364-76.

202. Reilly JC, Houghton C. The experiences and perceptions of care in acute settings for patients living with dementia: A qualitative evidence synthesis. International Journal of Nursing Studies. 2019;96:82-90.

203. Sanakova S, Cap J. Dignity from the nurses' and older patients' perspective: A qualitative literature review. Nursing Ethics. 2019;26(5):1292-309.

204. Kennedy MC, Pallotti P, Dickinson R, Harley C. 'If you can't see a dilemma in this situation you should probably regard it as a warning': a metasynthesis and theoretical modelling of general practitioners' opioid prescription experiences in primary care. British Journal of Pain. 2019;13(3):159-76.

205. Treacy M, Stayt LC. To identify the factors that influence the recognizing and responding to adult patient deterioration in acute hospitals. Journal of Advanced Nursing. 2019;75(12):3272-85.

206. Hamilton C, Lovarini M, McCloskey A, de Campos TF, Hassett L. Experiences of therapists using feedback-based technology to improve physical function in rehabilitation settings: a qualitative systematic review. Disability and Rehabilitation. 2019;41(15):1739-50.

207. Brown P, Hewison A, Newham R. What are research nurses' experiences of obtaining consent from or for patients participating in emergency care research? A qualitative review. Journal of Clinical Nursing. 2019;28(23-24):4155-65.

208. Hodson N, Bewley S. Abuse in assisted reproductive technology: A systematic qualitative review and typology. European Journal of Obstetrics & Gynecology and Reproductive Biology. 2019;238:170-7.

209. Gessler D, Juraskova I, Sansom-Daly UM, Shepherd HL, Patterson P, Muscat DM. Clinician-patient-family decision-making and health literacy in adolescents and young adults with cancer and their families: A systematic review of qualitative studies. Psycho-Oncology. 2019;28(7):1408-19.

210. Boshoff K, Gibbs D, Phillips RL, Wiles L, Porter L. A meta-synthesis of how parents of children with autism describe their experience of advocating for their children during the process of diagnosis. Health & Social Care in the Community. 2019;27(4):E143-E57.

211. Maurer C, Draganescu S, Mayer H, Gattinger H. Attitudes and needs of residents in long-term care facilities regarding physical activity-A systematic review and synthesis of qualitative studies. Journal of Clinical Nursing. 2019;28(13-14):2386-400.

212. Capilla-Diaz C, Bonill-de las Nieves C, Hernandez-Zambrano SM, Montoya-Juarez R, Morales-Asencio JM, Perez-Marfil MN, et al. Living With an Intestinal Stoma: A Qualitative Systematic Review. Qualitative Health Research. 2019;29(9):1255-65.

213. Unger J, Singh H, Mansfield A, Hitzig SL, Lenton E, Musselman KE. The experiences of physical rehabilitation in individuals with spinal cord injuries: a qualitative thematic synthesis. Disability and Rehabilitation. 2019;41(12):1367-83.

214. Cahill M, Pettigrew J, Robinson K, Galvin R. The Transition to Retirement Experiences of Academics in "Higher Education": A Meta-Ethnography. Gerontologist. 2019;59(3):E177-E95.

215. Cohen R, Gooberman-Hill R. Staff experiences of enhanced recovery after surgery: systematic review of qualitative studies. Bmj Open. 2019;9(2).

216. Gonella S, Basso I, De Marinis MG, Campagna S, Di Giulio P. Good end-of-life care in nursing home according to the family carers' perspective: A systematic review of qualitative findings. Palliative Medicine. 2019;33(6):589-606.

217. Turner V, Flemming K. Socioeconomic factors affecting access to preferred place of death: A qualitative evidence synthesis. Palliative Medicine. 2019;33(6):607-17.

218. Corr M, McSharry J, Murtagh EM. Adolescent Girls' Perceptions of Physical Activity: A Systematic Review of Qualitative Studies. American Journal of Health Promotion. 2019;33(5):806-19.

219. Ryan L, Jackson D, Woods C, Usher K. Intentional rounding - An integrative literature review. Journal of Advanced Nursing. 2019;75(6):1151-61.

220. Teoh SL, Ngorsuraches S, Lai NM, Bangpan M, Chaiyakunapruk N. Factors affecting consumers' decisions on the use of nutraceuticals: a systematic review. International Journal of Food Sciences and Nutrition. 2019;70(4):491-512.

221. Evans C, Tweheyo R, McGarry J, Eldridge J, Albert J, Nkoyo V, et al. Seeking culturally safe care: a qualitative systematic review of the healthcare experiences of women and girls who have undergone female genital mutilation/cutting. Bmj Open. 2019;9(5).

222. Feeley C, Thomson G, Downe S. Caring for women making unconventional birth choices: A meta-ethnography exploring the views, attitudes, and experiences of midwives. Midwifery. 2019;72:50-9.

223. Kaur J, Farley A, Jolly K, Jones LL. Primary Care Healthcare Professionals' Knowledge, Attitudes, and Practices Towards Promoting the Reduction of Children's Secondhand Smoke Exposure: A Mixed-Methods Review and Synthesis. Nicotine & Tobacco Research. 2019;21(4):398-408.

224. Marshall S, Fleming A, Moore AC, Sahm LJ. Views of parents regarding human papillomavirus vaccination: A systematic review and meta-ethnographic synthesis of qualitative literature. Research in Social & Administrative Pharmacy. 2019;15(4):331-7.

225. Lumley E, Phillips P, Aber A, Buckley-Woods H, Jones GL, Michaels JA. Experiences of living with varicose veins: A systematic review of qualitative research. Journal of Clinical Nursing. 2019;28(7-8):1085-99.

226. Morgan K, Campbell KL, Reidlinger DP. Dietetics students' experiences of dietetics workforce preparation and preparedness: a systematic review and qualitative synthesis. Journal of Human Nutrition and Dietetics. 2019;32(2):226-46.

227. Lees-Deutsch L, Robinson J. A Systematic Review of Criteria-Led Patient Discharge. Journal of Nursing Care Quality. 2019;34(2):121-6.

228. Warwick H, Mansell W, Porter C, Tai S. 'What people diagnosed with bipolar disorder experience as distressing': A meta-synthesis of qualitative research. Journal of Affective Disorders. 2019;248:108-30.

229. Evans C, Tweheyo R, McGarry J, Eldridge J, Albert J, Nkoyo V, et al. Crossing cultural divides: A qualitative systematic review of factors influencing the provision of healthcare related to female genital mutilation from the perspective of health professionals. Plos One. 2019;14(3).

230. Baker E, Fatoye F. Patient perceived impact of nurse-led self-management interventions for COPD: A systematic review of qualitative research. International Journal of Nursing Studies. 2019;91:22-34.

231. Welk B, McGarry P, Kennelly M, Myers J, Neurogenic Bladder Res G. Bladder management experiences among people living with neurologic disease: A systematic review and meta-synthesis of qualitative research. Neurourology and Urodynamics. 2019;38(2):668-76.

232. Ghirotto L, Busani E, Salvati M, Di Marco V, Caldarelli V, Artioli G. Researching children's perspectives in pediatric palliative care: A systematic review and meta-summary of qualitative research. Palliative & Supportive Care. 2019;17(1):107-18.

233. Hildebrandt C, Mayer H, Koller A. Experiences of patients with colorectal cancer from diagnosis until completion of treatment: A meta-ethnography approach. Psycho-Oncology. 2019;28(2):219-27.

234. Li HH, Shao YP, Xing ZJ, Li Y, Wang SQ, Zhang ML, et al. Napping on night-shifts among nursing staff: A mixed-methods systematic review????????:?????????? Journal of Advanced Nursing. 2019;75(2):291-312.

235. Young L, Murata L, McPherson C, Jacob JD, Vandyk AD. Exploring the Experiences of Parent Caregivers of Schizophrenia: A Systematic Review. Archives of Psychiatric Nursing. 2019;33(1):93-103.

236. Pattni N, Arzola C, Malavade A, Varmani S, Krimus L, Friedman Z. Challenging authority and speaking up in the operating room environment: a narrative synthesis. British Journal of Anaesthesia. 2019;122(2):233-44.

237. McGrath M, Lever S, McCluskey A, Power E. How is sexuality after stroke experienced by stroke survivors and partners of stroke survivors? A systematic review of qualitative studies. Clinical Rehabilitation. 2019;33(2):293-303.

238. Sisson H, Wilkinson Y. An Integrative Review of the Influences on Decision-Making of Young People About Human Papillomavirus Vaccine. Journal of School Nursing. 2019;35(1):39-50.

239. Coates R, Cupples G, Scamell A, McCourt C. Women's experiences of induction of labour: Qualitative systematic review and thematic synthesis. Midwifery. 2019;69:17-28.

240. Araujo JS, Zago MMF. Masculinities of prostate cancer survivors: a qualitative metasynthesis. Revista Brasileira De Enfermagem. 2019;72(1):231-40.

241. Parretti HM, Hughes CA, Jones LL. 'The rollercoaster of follow-up care' after bariatric surgery: a rapid review and qualitative synthesis. Obesity Reviews. 2019;20(1):88-107.

242. Franklin P, Arber A, Reed L, Ream E. Health and social care professionals' experiences of supporting parents and their dependent children during, and following, the death of a parent: A qualitative review and thematic synthesis. Palliative Medicine. 2019;33(1):49-65.

243. Griffiths R, Mansell W, Edge D, Tai S. Sources of Distress in First-Episode Psychosis: A Systematic Review and Qualitative Metasynthesis. Qualitative Health Research. 2019;29(1):107-23.

244. Chong PH, Walshe C, Hughes S. Perceptions of a Good Death in Children with Life-Shortening Conditions: An Integrative Review. Journal of Palliative Medicine. 2019;22(6):714-23.

245. Chen CQ, Chow AYM, Tang SQ. Bereavement process of professional caregivers after deaths of their patients: A meta-ethnographic synthesis of qualitative studies and an integrated model. International Journal of Nursing Studies. 2018;88:104-13.

246. Kharicha K, Manthorpe J, Iliffe S, Davies N, Walters K. Strategies employed by older people to manage loneliness: systematic review of qualitative studies and model development. International Psychogeriatrics. 2018;30(12):1767-81.

247. Nybakken S, Strandas M, Bondas T. Caregivers' perceptions of aggressive behaviour in nursing home residents living with dementia: A meta-ethnography. Journal of Advanced Nursing. 2018;74(12):2713-26.

248. Nelwati, Abdullah KL, Chan CM. A systematic review of qualitative studies exploring peer learning experiences of undergraduate nursing students. Nurse Education Today. 2018;71:185-92.

249. Dube E, Gagnon D, MacDonald N, Bocquier A, Peretti-Watel P, Verger P. Underlying factors impacting vaccine hesitancy in high income countries: a review of qualitative studies. Expert Review of Vaccines. 2018;17(11):989-1004.

250. Fourie S, Jackson D, Aveyard H. Living with Inflammatory Bowel Disease: A review of qualitative research studies. International Journal of Nursing Studies. 2018;87:149-56.

251. Hall NJ, Donovan G, Wilkes S. A qualitative synthesis of pharmacist, other health professional and lay perspectives on the role of community pharmacy in facilitating care for people with long-term conditions. Research in Social & Administrative Pharmacy. 2018;14(11):1043-57.

252. Bright KS, Norris JM, Letourneau NL, Rosario MK, Premji SS. Prenatal Maternal Anxiety in South Asia: A Rapid Best-Fit Framework Synthesis. Frontiers in Psychiatry. 2018;9.

253. O'Connor S, Jolliffe S, Stanmore E, Renwick L, Booth R. Social media in nursing and midwifery education: A mixed study systematic review. Journal of Advanced Nursing. 2018;74(10):2273-89.

254. Carruthers H, Gomersall T, Astin F. The work undertaken by mechanically ventilated patients in Intensive Care: A qualitative meta-ethnography of survivors' experiences. International Journal of Nursing Studies. 2018;86:60-73.

255. Ferreira GE, Traeger AC, O'Keeffe M, Maher CG. Staff and patients have mostly positive perceptions of physiotherapists working in emergency departments: a systematic review. Journal of Physiotherapy. 2018;64(4):229-36.

256. Cooper E, Hutchinson A, Sheikh Z, Taylor P, Townend W, Johnson MJ. Palliative care in the emergency department: A systematic literature qualitative review and thematic synthesis. Palliative Medicine. 2018;32(9):1443-54.

257. Atienzo EE, Kaltenthaler E, Baxter SK. Barriers and Facilitators to the Implementation of Interventions to Prevent Youth Violence in Latin America: A Systematic Review and Qualitative Evidence Synthesis. Trauma Violence & Abuse. 2018;19(4):420-30.

258. Angarita FA, Elmi M, Zhang YM, Hong NJL. Patient-reported factors influencing the treatment decision-making process of older women with non-metastatic breast cancer: a systematic review of qualitative evidence. Breast Cancer Research and Treatment. 2018;171(3):545-64.

259. Bjornnes AK, Parry M, Leegaard M, Ayala AP, Lenton E, Harvey P, et al. Self-Management of Cardiac Pain in Women: A Meta-Summary of the Qualitative Literature. Qualitative Health Research. 2018;28(11):1769-87.

260. Wride JM, Bannigan K. 'If you can't help me, so help me God I will cut it off myself ...' The experience of living with knee pain: a qualitative meta-synthesis. Physiotherapy. 2018;104(3):299-310.

261. Kornhaber R, Childs C, Cleary M. Experiences of guilt, shame and blame in those affected by burns: A qualitative systematic review. Burns. 2018;44(5):1026-39.

262. Grimston M, Butler AE, Copnell B. Critical care nurses' experiences of caring for a dying child: A qualitative evidence synthesis. Journal of Advanced Nursing. 2018;74(8):1752-68.

263. Forbes Shepherd R, Lewis A, Keogh LA, Werner-Lin A, Delatycki MB, Forrest LE. A Systematic Review of How Young People Live with Inherited Disease: What Can We Learn for Li-Fraumeni Syndrome? Journal of Adolescent and Young Adult Oncology. 2018;7(5):525-45.

264. Thaivalappil A, Waddell L, Greig J, Meldrum R, Young I. A systematic review and thematic synthesis of qualitative research studies on factors affecting safe food handling at retail and food service. Food Control. 2018;89:97-107.

265. Aylagas-Crespillo M, Garcia-Barbero O, Rodriguez-Martin B. Barriers in the social and healthcare assistance for transgender persons: A systematic review of qualitative studies. Enfermeria Clinica. 2018;28(4):247-59.

266. Signorelli F, Sela S, Gesualdo L, Chevrel S, Tollet F, Pailler-Mattei C, et al. Hemodynamic Stress, Inflammation, and Intracranial Aneurysm Development and Rupture: A Systematic Review. World Neurosurgery. 2018;115:234-44.

267. Stevanin S, Palese A, Bressan V, Vehvilainen-Julkunen K, Kvist T. Workplace-related generational characteristics of nurses: A mixed-method systematic review. Journal of Advanced Nursing. 2018;74(6):1245-63.

268. Aristidou M, Vouzavali F, Karanikola MN, Lambrinou E, Papathanassoglou E. A Meta-ethnography of Out-of-Hospital Cardiac Arrest Survivors' Meanings on Life and Death. Journal of Cardiovascular Nursing. 2018;33(3):E10-E20.

269. Alburquerque-Sendin F, Ferrari AV, Rodrigues-de-Souza DP, Paras-Bravo P, Velarde-Garcia JF, Palacios-Cena D. The experience of being a psychiatric nurse in South Africa: A qualitative systematic review. Nursing Outlook. 2018;66(3):293-310.

270. Devan H, Hale L, Hempel D, Saipe B, Perry MA. What Works and Does Not Work in a Self-Management Intervention for People With Chronic Pain? Qualitative Systematic Review and Meta-Synthesis. Physical Therapy. 2018;98(5):381-97.

271. Kisch AM, Forsberg A, Fridh I, Almgren M, Lundmark M, Loven C, et al. The Meaning of Being a Living Kidney, Liver, or Stem Cell Donor-A Meta-Ethnography. Transplantation. 2018;102(5):744-56.

272. Messer LH, Johnson R, Driscoll KA, Jones J. Best friend or spy: a qualitative meta-synthesis on the impact of continuous glucose monitoring on life with Type 1 diabetes. Diabetic Medicine. 2018;35(4):409-18.

273. Lewis NV, Feder GS, Howarth E, Szilassy E, McTavish JR, MacMillan HL, et al. Identification and initial response to children's exposure to intimate partner violence: a qualitative synthesis of the perspectives of children, mothers and professionals. Bmj Open. 2018;8(4).

274. Matvienko-Sikar K, Kelly C, Sinnott C, McSharry J, Houghton C, Heary C, et al. Parental experiences and perceptions of infant complementary feeding: a qualitative evidence synthesis. Obesity Reviews. 2018;19(4):501-17.

275. Rothmann MJ, Jakobsen PR, Jensen CM, Hermann AP, Smith AC, Clemensen J. Experiences of being diagnosed with osteoporosis: a meta-synthesis. Archives of Osteoporosis. 2018;13(1).

276. Bergner EM, Cornish EK, Horne K, Griffith DM. A qualitative meta-synthesis examining the role of women in African American men's prostate cancer screening and treatment decision making. Psycho-Oncology. 2018;27(3):781-90.

277. Phillips P, Lumley E, Duncan R, Aber A, Woods HB, Jones GL, et al. A systematic review of qualitative research into people's experiences of living with venous leg ulcers. Journal of Advanced Nursing. 2018;74(3):550-63.

278. Wilson L, Rubens-Augustson T, Murphy M, Jardine C, Crowcroft N, Hui C, et al. Barriers to immunization among newcomers: A systematic review. Vaccine. 2018;36(8):1055-62.

279. Stephen C, McInnes S, Halcomb E. The feasibility and acceptability of nurse-led chronic disease management interventions in primary care: An integrative review. Journal of Advanced Nursing. 2018;74(2):279-88.

280. Hutchinson A, Barclay-Klingle N, Galvin K, Johnson MJ. Living with breathlessness: a systematic literature review and qualitative synthesis. European Respiratory Journal. 2018;51(2).

281. Moran GM, Nairn S. How does role transition affect the experience of trainee Advanced Clinical Practitioners: Qualitative evidence synthesis. Journal of Advanced Nursing. 2018;74(2):251-62.

282. Nowell L, Ovie G, Berenson C, Kenny N, Hayden KA. Professional Learning and Development of Postdoctoral Scholars: A Systematic Review of the Literature. Education Research International. 2018.

283. Rocha JS, Arima L, Chibinski AC, Werneck RI, Moyses SJ, Baldani MH. Barriers and facilitators to dental care during pregnancy: a systematic review and meta-synthesis of qualitative studies. Cadernos De Saude Publica. 2018;34(8).

284. Hilton G, Unsworth C, Murphy G. The experience of attempting to return to work following spinal cord injury: a systematic review of the qualitative literature. Disability and Rehabilitation. 2018;40(15):1745-53.

285. Panagiotopoulou N, Ghuman N, Sandher R, Herbert M, Stewart JA. Barriers and facilitators towards fertility preservation care for cancer patients: a meta-synthesis. European Journal of Cancer Care. 2018;27(1).

286. Kornhaber R, McLean L, Betihavas V, Cleary M. Resilience and the rehabilitation of adult spinal cord injury survivors: A qualitative systematic review. Journal of Advanced Nursing. 2018;74(1):23-33.

287. Polita NB, Alvarenga WD, Leite A, Araujo JS, dos Santos L, Zago MMF, et al. Care provided by the father to the child with cancer under the influence of masculinities: qualitative meta-synthesis. Revista Brasileira De Enfermagem. 2018;71(1):185-94.

288. Clari M, Ivziku D, Casciaro R, Matarese M. The Unmet Needs of People with Chronic Obstructive Pulmonary Disease: A Systematic Review of Qualitative Findings. Copd-Journal of Chronic Obstructive Pulmonary Disease. 2018;15(1):79-88.

289. Vanderspank-Wright B, Efstathiou N, Vandyk AD. Critical care nurses' experiences of withdrawal of treatment: A systematic review of qualitative evidence. International Journal of Nursing Studies. 2018;77:15-26.

290. Harrison AL, Taylor NF, Shields N, Frawley HC. Attitudes, barriers and enablers to physical activity in pregnant women: a systematic review. Journal of Physiotherapy. 2018;64(1):24-32.

291. Boshoff K, Gibbs D, Phillips RL, Wiles L, Porter L. Parents' voices: "Our process of advocating for our child with autism." A meta-synthesis of parents' perspectives. Child Care Health and Development. 2018;44(1):147-60.

292. Gardiner S, Glogowska M, Stoddart C, Pendlebury S, Lasserson D, Jackson D. Older people's experiences of falling and perceived risk of falls in the community: A narrative synthesis of qualitative research. International Journal of Older People Nursing. 2017;12(4).

293. Bastounis A, Callaghan P, Lykomitrou F, Aubeeluck A, Michail M. Exploring Students' Participation in Universal, Depression and Anxiety, Prevention Programmes at School: A Meta-aggregation. School Mental Health. 2017;9(4):372-85.

294. Walsh KA, Dennehy R, Sinnott C, Browne J, Byrne S, McSharry J, et al. Influences on Decision-Making Regarding Antipsychotic Prescribing in Nursing Home Residents With Dementia: A Systematic Review and Synthesis of Qualitative Evidence. Journal of the American Medical Directors Association. 2017;18(10).

295. Kong EH, Deatrick JA, Bradway CK. Men's experiences after prostatectomy: A meta-synthesis. International Journal of Nursing Studies. 2017;74:162-71.

296. Tanay MAL, Armes J, Ream E. The experience of chemotherapy-induced peripheral neuropathy in adult cancer patients: a qualitative thematic synthesis. European Journal of Cancer Care. 2017;26(5).

297. Bath-Hextall F, Nalubega S, Evans C. The needs and experiences of patients with skin cancer: a qualitative systematic review with metasynthesis. British Journal of Dermatology. 2017;177(3):666-87.

298. Nowell L, Norris JM, Mrklas K, White DE. A literature review of mentorship programs in academic nursing. Journal of Professional Nursing. 2017;33(5):334-44.

299. Rankin D, Harden J, Jepson R, Lawton J. Children's experiences of managing Type 1 diabetes in everyday life: a thematic synthesis of qualitative studies. Diabetic Medicine. 2017;34(8):1050-60.

300. McKendy KM, Watanabe Y, Lee L, Bilgic E, Enani G, Feldman LS, et al. Perioperative feedback in surgical training: A systematic review. American Journal of Surgery. 2017;214(1):117-26.

301. Virdun C, Luckett T, Lorenz K, Davidson PM, Phillips J. Dying in the hospital setting: A meta-synthesis identifying the elements of end-of-life care that patients and their families describe as being important. Palliative Medicine. 2017;31(7):587-601.

302. Mc Gillicuddy A, Kelly M, Crean AM, Sahm LJ. The knowledge, attitudes and beliefs of patients and their healthcare professionals around oral dosage form modification: A systematic review of the qualitative literature. Research in Social & Administrative Pharmacy. 2017;13(4):717-26.

303. Bauld L, Graham H, Sinclair L, Flemming K, Naughton F, Ford A, et al. Barriers to and facilitators of smoking cessation in pregnancy and following childbirth: literature review and qualitative study. Health Technology Assessment. 2017;21(36):1-+.

304. Templeton M, Lohan M, Kelly C, Lundy L. A systematic review and qualitative synthesis of adolescents' views of sexual readiness. Journal of Advanced Nursing. 2017;73(6):1288-301.

305. Hall BJ, Sou KL, Beanland R, Lacky M, Tso LS, Ma QY, et al. Barriers and Facilitators to Interventions Improving Retention in HIV Care: A Qualitative Evidence Meta-Synthesis. Aids and Behavior. 2017;21(6):1755-67.

306. Egerton T, Diamond LE, Buchbinder R, Bennell KL, Slade SC. A systematic review and evidence synthesis of qualitative studies to identify primary care clinicians' barriers and enablers to the management of osteoarthritis. Osteoarthritis and Cartilage. 2017;25(5):625-38.

307. Hall H, Leach M, Brosnan C, Collins M. Nurses' attitudes towards complementary therapies: A systematic review and meta-synthesis. International Journal of Nursing Studies. 2017;69:47-56.

308. Herbert AC, Ramirez AM, Lee G, North SJ, Askari MS, West RL, et al. Puberty Experiences of Low-Income Girls in the United States: A Systematic Review of Qualitative Literature From 2000 to 2014. Journal of Adolescent Health. 2017;60(4):363-79.

309. Noonan M, Galvin R, Doody O, Jomeen J. A qualitative meta-synthesis: public health nurses role in the identification and management of perinatal mental health problems. Journal of Advanced Nursing. 2017;73(3):545-57.

310. Divanoglou A, Georgiou M. Perceived effectiveness and mechanisms of community peer-based programmes for Spinal Cord Injuries- a systematic review of qualitative findings. Spinal Cord. 2017;55(3):225-34.

311. Nowell L, Norris JM, Mrklas K, White DE. Mixed methods systematic review exploring mentorship outcomes in nursing academia. Journal of Advanced Nursing. 2017;73(3):527-44.

312. Sibeoni J, Orri M, Valentin M, Podlipski MA, Colin S, Pradere J, et al. Metasynthesis of the Views about Treatment of Anorexia Nervosa in Adolescents: Perspectives of Adolescents, Parents, and Professionals. Plos One. 2017;12(1).

313. Sibeoni J, Orri M, Colin S, Valentin M, Pradere J, Revah-Levy A. The lived experience of anorexia nervosa in adolescence, comparison of the points of view of adolescents, parents, and professionals: A metasynthesis. International Journal of Nursing Studies. 2017;65:25-34.

314. Kong EH, Choi H, Evans LK. Staff perceptions of barriers to physical restraint-reduction in long-term care: a meta-synthesis. Journal of Clinical Nursing. 2017;26(1-2):49-60.

315. Trainor K, Leavey G. Barriers and Facilitators to Smoking Cessation Among People With Severe Mental Illness: A Critical Appraisal of Qualitative Studies. Nicotine & Tobacco Research. 2017;19(1):14-23.

316. dos Reis SMG, Leite A, Alvarenga WD, Araujo JS, Zago MMF, Nascimento LC. Meta-synthesis about man as a father and caregiver for a hospitalized child. Revista Latino-Americana De Enfermagem. 2017;25.

317. Greaves C, Poltawski L, Garside R, Briscoe S. Understanding the challenge of weight loss maintenance: a systematic review and synthesis of qualitative research on weight loss maintenance. Health Psychology Review. 2017;11(2):145-63.

318. French B, Thomas LH, Harrison J, Coupe J, Roe B, Booth J, et al. Client and clinical staff perceptions of barriers to and enablers of the uptake and delivery of behavioural interventions for urinary incontinence: qualitative evidence synthesis. Journal of Advanced Nursing. 2017;73(1):21-38.

319. Moore DA, Goodwin TL, Brocklehurst PR, Armitage CJ, Glenny AM. When Are Caregivers More Likely to Offer Sugary Drinks and Snacks to Infants? A Qualitative Thematic Synthesis. Qualitative Health Research. 2017;27(1):74-88.

320. McSharry J, McGowan L, Farmer AJ, French DP. Perceptions and experiences of taking oral medications for the treatment of Type 2 diabetes mellitus: a systematic review and meta-synthesis of qualitative studies. Diabetic Medicine. 2016;33(10):1330-8.

321. Olano-Lizarraga M, Oroviogoicoechea C, Errasti-Ibarrondo B, Saracibar-Razquin M. The personal experience of living with chronic heart failure: a qualitative meta-synthesis of the literature. Journal of Clinical Nursing. 2016;25(17-18):2413-29.

322. Slade SC, Kent P, Patel S, Bucknall T, Buchbinder R. Barriers to Primary Care Clinician Adherence to Clinical Guidelines for the Management of Low Back Pain A Systematic Review and Metasynthesis of Qualitative Studies. Clinical Journal of Pain. 2016;32(9):800-16.

323. Holten L, de Miranda E. Women's motivations for having unassisted childbirth or high-risk homebirth: An exploration of the literature on 'birthing outside the system'. Midwifery. 2016;38:55-62.

324. Waldemar AK, Arnfred SM, Petersen L, Korsbek L. Recovery-Oriented Practice in Mental Health Inpatient Settings: A Literature Review. Psychiatric Services. 2016;67(6):595-601.

325. Swerts M, Westhof E, Bogaerts A, Lemiengre J. Supporting breast-feeding women from the perspective of the midwife: A systematic review of the literature. Midwifery. 2016;37:32-40.

326. Honein-AbouHaidar GN, Kastner M, Vuong V, Perrier L, Daly C, Rabeneck L, et al. Systematic Review and Meta-study Synthesis of Qualitative Studies Evaluating Facilitators and Barriers to Participation in Colorectal Cancer Screening. Cancer Epidemiology Biomarkers & Prevention. 2016;25(6):907-17.

327. O'Keeffe M, Cullinane P, Hurley J, Leahy I, Bunzli S, O'Sullivan PB, et al. What Influences Patient-Therapist Interactions in Musculoskeletal Physical Therapy? Qualitative Systematic Review and Meta-Synthesis. Physical Therapy. 2016;96(5):609-22.

328. Drew S, Lavy C, Gooberman-Hill R. What factors affect patient access and engagement with clubfoot treatment in low- and middle-income countries? Meta-synthesis of existing qualitative studies using a social ecological model. Tropical Medicine & International Health. 2016;21(5):570-89.

329. Tan SY, Melendez-Torres GJ. A systematic review and metasynthesis of barriers and facilitators to negotiating consistent condom use among sex workers in Asia. Culture Health & Sexuality. 2016;18(3):249-64.

330. Garcia-Escamilla E, Rodriguez-Martin B, Martinez-Vizcaino V. Integration of acupuncture into conventional medicine from health professionals' perspective: A thematic synthesis of qualitative studies. Health. 2016;20(2):176-200.

331. Mikkonen K, Elo S, Kuivila HM, Tuomikoski AM, Kaariainen M. Culturally and linguistically diverse healthcare students' experiences of learning in a clinical environment: A systematic review of qualitative studies. International Journal of Nursing Studies. 2016;54:173-87.

332. Shepherd A, Doyle M, Sanders C, Shaw J. Personal recovery within forensic settings - Systematic review and meta-synthesis of qualitative methods studies. Criminal Behaviour and Mental Health. 2016;26(1):59-75.

333. Shepherd A, Sanders C, Doyle M, Shaw J. Personal recovery in personality disorder: Systematic review and meta-synthesis of qualitative methods studies. International Journal of Social Psychiatry. 2016;62(1):41-50.

334. Wao H, Aluoch M, Odondi GO, Tenge E, Iznaga T. MSM's Versus Health Care Providers' Perceptions of Barriers to Uptake of HIV/AIDS-Related Interventions: Systematic Review and Meta-Synthesis of Qualitative and Quantitative Evidence. International Journal of Sexual Health. 2016;28(2):151-62.

335. Riley R, Weiss MC. A qualitative thematic review: emotional labour in healthcare settings. Journal of Advanced Nursing. 2016;72(1):6-17.

336. Supper I, Catala O, Lustman M, Chemla C, Bourgueil Y, Letrilliart L. Interprofessional collaboration in primary health care: a review of facilitators and barriers perceived by involved actors. Journal of Public Health. 2015;37(4):716-27.

337. Johnson M, Tod AM, Brummell S, Collins K. Prognostic communication in cancer: A critical interpretive synthesis of the literature. European Journal of Oncology Nursing. 2015;19(5):554-67.

338. Butler AE, Hall H, Willetts G, Copnell B. Family Experience and PICU Death: A Meta-Synthesis. Pediatrics. 2015;136(4):E961-E73.

339. Bousquet G, Orri M, Winterman S, Brugiere C, Verneuil L, Revah-Levy A. Breaking Bad News in Oncology: A Metasynthesis. Journal of Clinical Oncology. 2015;33(22):2437-U44.

340. van der Kleij R, Coster N, Verbiest M, van Assema P, Paulussen T, Reis R, et al. Implementation of intersectoral community approaches targeting childhood obesity: a systematic review. Obesity Reviews. 2015;16(6):454-72.

341. Flemming K, McCaughan D, Angus K, Graham H. Qualitative systematic review: barriers and facilitators to smoking cessation experienced by women in pregnancy and following childbirth. Journal of Advanced Nursing. 2015;71(6):1210-26.

342. Rimstad R, Braut GS. Literature Review on Medical Incident Command. Prehospital and Disaster Medicine. 2015;30(2):205-15.

343. Livingstone R, Field D. The child and family experience of power mobility: a qualitative synthesis. Developmental Medicine and Child Neurology. 2015;57(4):317-27.

344. Synnott A, O'Keeffe M, Bunzli S, Dankaerts W, O'Sullivan P, O'Sullivan K. Physiotherapists may stigmatise or feel unprepared to treat people with low back pain and psychosocial factors that influence recovery: a systematic review. Journal of Physiotherapy. 2015;61(2):68-76.

345. Hughes-Morley A, Young B, Waheed W, Small N, Bower P. Factors affecting recruitment into depression trials: Systematic review, meta-synthesis and conceptual framework. Journal of Affective Disorders. 2015;172:274-90.

346. Mudge S, Kayes N, McPherson K. Who is in control? Clinicians' view on their role in self-management approaches: a qualitative metasynthesis. Bmj Open. 2015;5(5).

347. Perrar KM, Schmidt H, Eisenmann Y, Cremer B, Voltz R. Needs of People with Severe Dementia at the End-of-Life: A Systematic Review. Journal of Alzheimers Disease. 2015;43(2):397-413.

348. Toye F, Seers K, Barker K. A meta-ethnography of patients' experiences of chronic pelvic pain: struggling to construct chronic pelvic pain as 'real'. Journal of Advanced Nursing. 2014;70(12):2713-27.

349. Perez GM, Aznar CT, Bagnol B. LABIA MINORA ELONGATION AND ITS IMPLICATIONS ON THE HEALTH OF WOMEN: A SYSTEMATIC REVIEW. International Journal of Sexual Health. 2014;26(3):155-71.

350. Best M, Butow P, Olver I. Do patients want doctors to talk about spirituality? A systematic literature review. Patient Education and Counseling. 2015;98(11):1320-8.

351. Albury C, Hall A, Syed A, Ziebland S, Stokoe E, Roberts N, et al. Communication practices for delivering health behaviour change conversations in primary care: a systematic review and thematic synthesis. BMC Fam Pract. 2019;20(1):111.

352. AlOmeir O, Patel N, Donyai P. Adherence to adjuvant endocrine therapy among breast cancer survivors: a systematic review and meta-synthesis of the qualitative literature using grounded theory. Support Care Cancer. 2020.

353. Alvarenga WDA, de Montigny F, Zeghiche S, Polita NB, Verdon C, Nascimento LC. Understanding the spirituality of parents following stillbirth: A qualitative meta-synthesis. Death Studies. 2019.

354. Barker J, Kumar A, Stanton W, Bath-Hextall F. The needs and experiences of people with a diagnosis of skin cancer: a systematic review. JBI Libr Syst Rev. 2011;9(4):104-21.

355. Beentjes TAA, van Gaal BGI, van Achterberg T, Goossens PJJ. Self-Management Support Needs From the Perspectives of Persons With Severe Mental Illness: A Systematic Review and Thematic Synthesis of Qualitative Research. J Am Psychiatr Nurses Assoc. 2019:1078390319877953.

356. Clyne B, O'Neill SM, Nuzum D, O'Neill M, Larkin J, Ryan M, et al. Patient's spirituality perspectives at the end of life: A qualitative evidence synthesis. BMJ Supportive and Palliative Care. 2019;(no pagination).

357. Compernolle S, De Cocker K, Cardon G, De Bourdeaudhuij I, Van Dyck D. Older Adults' Perceptions of Sedentary Behavior: A Systematic Review and Thematic Synthesis of Qualitative Studies. Gerontologist. 2019.

358. Hewison A, Atkin K, McCaughan D, Roman E, Smith A, Smith G, et al. Experiences of living with chronic myeloid leukaemia and adhering to tyrosine kinase inhibitors: A thematic synthesis of qualitative studies. European journal of oncology nursing : the official journal of European Oncology Nursing Society. 2020;45:101730.

359. Ho RS, Wong CH, Wu JC, Wong SY, Chung VC. Non-specific effects of acupuncture and sham acupuncture in clinical trials from the patient's perspective: a systematic review of qualitative evidence. Acupunct Med. 2020:964528420920299.

360. Leahy E, Chipchase L, Calo M, Blackstock FC. Which Learning Activities Enhance Physical Therapist Practice? Part 2: Systematic Review of Qualitative Studies and Thematic Synthesis. Phys Ther. 2020;100(9):1484-501.

361. Leite ACAB, Garcia-Vivar C, Neris RR, Alvarenga WDA, Nascimento LC. The experience of hope in families of children and adolescents living with chronic illness: A thematic synthesis of qualitative studies. Journal of Advanced Nursing. 2019;75(12):3246-62.

362. Liu C, Wang D, Liu C, Jiang J, Wang X, Chen H, et al. What is the meaning of health literacy? A systematic review and qualitative synthesis. Fam Med Community Health. 2020;8(2).

363. Mitchell KR, Brassil KJ, Rodriguez SA, Tsai E, Fujimoto K, Krause KJ, et al. Operationalizing patient-centered cancer care: A systematic review and synthesis of the qualitative literature on cancer patients' needs, values, and preferences. Psychooncology. 2020.

364. Paisi M, Kay E, Plessas A, Burns L, Quinn C, Brennan N, et al. Barriers and enablers to accessing dental services for people experiencing homelessness: A systematic review. Community Dent Oral Epidemiol. 2019;47(2):103-11.

365. Sampson R, Cooper J, Barbour R. Patients' perspectives on the medical primary-secondary care interface: systematic review and synthesis of qualitative research (vol 5, e008708, 2015). Bmj Open. 2018;8(1):1.

366. Savage M, Savage C, Brommels M, Mazzocato P. Medical leadership: boon or barrier to organisational performance? A thematic synthesis of the literature. BMJ Open. 2020;10(7):e035542.

367. Schichtel M, Wee B, MacArtney JI, Collins S. Clinician barriers and facilitators to heart failure advance care plans: a systematic literature review and qualitative evidence synthesis. BMJ supportive & palliative care. 2019;22.

368. Sharma S, Traeger AC, Reed B, Hamilton M, O'Connor DA, Hoffmann TC, et al. Clinician and patient beliefs about diagnostic imaging for low back pain: a systematic qualitative evidence synthesis. BMJ open. 2020;10(8):e037820.

369. Skea ZC, Aceves-Martins M, Robertson C, De Bruin M, Avenell A. Acceptability and feasibility of weight management programmes for adults with severe obesity: a qualitative systematic review. BMJ Open. 2019;9(9):e029473.

**Included reviews using both COREQ and ENTREQ (n=62)**

1. Scott MD, McQueen S, Richardson L. Teaching Health Advocacy: A Systematic Review of Educational Interventions for Postgraduate Medical Trainees. Academic Medicine. 2020;95(4):644-56.

2. Kanzow P, Wiegand A, Gostemeyer G, Schwendicke F. Understanding the management and teaching of dental restoration repair: Systematic review and meta-analysis of surveys. Journal of Dentistry. 2018;69:1-21.

3. Bailey PK, Hamilton AJ, Clissold RL, Inward CD, Caskey FJ, Ben-Shlomo Y, et al. Young adults' perspectives on living with kidney failure: a systematic review and thematic synthesis of qualitative studies. BMJ Open. 2018;8(1):e019926.

4. Aristidou M, Vouzavali F, Karanikola MN, Lambrinou E, Papathanassoglou E. A Meta-ethnography of Out-of-Hospital Cardiac Arrest Survivors' Meanings on Life and Death. Journal of Cardiovascular Nursing. 2018;33(3):E10-E20.

5. Chando S, Young C, Craig JC, Gunasekera H, Tong A. Parental views on otitis media: systematic review of qualitative studies. Eur J Pediatr. 2016;175(10):1295-305.

6. Somes E, Dukes J, Brungardt A, Jordan S, DeSanto K, Jones CD, et al. Perceptions of trained laypersons in end-of-life or advance care planning conversations: a qualitative meta-synthesis. BMC Palliat Care. 2018;17(1):98.

7. Kinnear FJ, Perry R, Searle A, Hamilton-Shield JP, Lithander FE. How do the experiences and beliefs of adults and children with heterozygous familial hypercholesterolaemia influence their adherence to treatment? A systematic review of qualitative evidence protocol. Syst Rev. 2018;7(1):120.

8. Christensen ME, Brincks J, Schnieber A, Soerensen D. The intention to exercise and the execution of exercise among persons with multiple sclerosis - a qualitative metasynthesis. Disability and Rehabilitation. 2016;38(11):1023-33.

9. Toye F, Seers K, Hannink E, Barker K. A mega-ethnography of eleven qualitative evidence syntheses exploring the experience of living with chronic non-malignant pain. Bmc Medical Research Methodology. 2017;17.

10. Fletcher BR, Hinton L, Hartmann-Boyce J, Roberts NW, Bobrovitz N, McManus RJ. Self-monitoring blood pressure in hypertension, patient and provider perspectives: A systematic review and thematic synthesis. Patient Education and Counseling. 2016;99(2):210-9.

11. Franco MR, Tong A, Howard K, Sherrington C, Ferreira PH, Pinto RZ, et al. Older people's perspectives on participation in physical activity: a systematic review and thematic synthesis of qualitative literature. British Journal of Sports Medicine. 2015;49(19):1268-76.

12. Freeman JL, Caldwell PHY, Bennett PA, Scott KM. How Adolescents Search for and Appraise Online Health Information: A Systematic Review. Journal of Pediatrics. 2018;195:244-+.

13. Jacobson J, Ju A, Baumgart A, Unruh M, O'Donoghue D, Obrador G, et al. Patient Perspectives on the Meaning and Impact of Fatigue in Hemodialysis: A Systematic Review and Thematic Analysis of Qualitative Studies. American Journal of Kidney Diseases. 2019;74(2):179-92.

14. Granger CL, Connolly B, Denehy L, Hart N, Antippa P, Lin KY, et al. Understanding factors influencing physical activity and exercise in lung cancer: a systematic review. Support Care Cancer. 2017;25(3):983-99.

15. Hall AM, Scurrey SR, Pike AE, Albury C, Richmond HL, Matthews J, et al. Physician-reported barriers to using evidence-based recommendations for low back pain in clinical practice: a systematic review and synthesis of qualitative studies using the Theoretical Domains Framework. Implement Sci. 2019;14(1):49.

16. Polita NB, de Montigny F, Neris RR, Alvarenga WD, Silva-Rodrigues FM, Leite A, et al. The Experiences of Bereaved Parents After the Loss of a Child to Cancer: A Qualitative Metasynthesis. Journal of Pediatric Oncology Nursing.

17. Acero AR, Cano-Prous A, Castellanos G, Martin-Lanas R, Canga-Armayor A. Family identity and severe mental illness: A thematic synthesis of qualitative studies. European Journal of Social Psychology. 2017;47(5):611-27.

18. Cleary M, West S, Hunt GE, McLean L, Kornhaber R. A Qualitative Systematic Review of Caregivers' Experiences of Caring for Family Diagnosed with Schizophrenia. Issues in Mental Health Nursing. 2020;41(8):667-83.

19. Erdos T, de Haan E, Heusinkveld S. Coaching: client factors & contextual dynamics in the change process A qualitative meta-synthesis. Coaching-an International Journal of Theory Research and Practice.

20. Jibb LA, Croal L, Wang JT, Yuan CR, Foster J, Cheung V, et al. Children's Experiences of Cancer Care: A Systematic Review and Thematic Synthesis of Qualitative Studies. Oncology Nursing Forum. 2018;45(4):527-44.

21. Kaur J, Farley A, Jolly K, Jones LL. Primary Care Healthcare Professionals' Knowledge, Attitudes, and Practices Towards Promoting the Reduction of Children's Secondhand Smoke Exposure: A Mixed-Methods Review and Synthesis. Nicotine & Tobacco Research. 2019;21(4):398-408.

22. Mechta Nielsen T, Frøjk Juhl M, Feldt-Rasmussen B, Thomsen T. Adherence to medication in patients with chronic kidney disease: a systematic review of qualitative research. Clin Kidney J. 2018;11(4):513-27.

23. Franklin P, Arber A, Reed L, Ream E. Health and social care professionals' experiences of supporting parents and their dependent children during, and following, the death of a parent: A qualitative review and thematic synthesis. Palliative Medicine. 2019;33(1):49-65.

24. Simpson-Adkins GJ, Daiches A. How Do Children Make Sense of their Parent's Mental Health Difficulties: A Meta-Synthesis. J Child Fam Stud. 2018;27(9):2705-16.

25. Joseph PD, Caldwell PHY, Tong A, Hanson CS, Craig JC. Stakeholder Views of Clinical Trials in Low- and Middle-Income Countries: A Systematic Review. Pediatrics. 2016;137(2).

26. Grimston M, Butler AE, Copnell B. Critical care nurses' experiences of caring for a dying child: A qualitative evidence synthesis. Journal of Advanced Nursing. 2018;74(8):1752-68.

27. Ju I, Banks E, Calabria B, Ju A, Agostino J, Korda RJ, et al. General practitioners' perspectives on the prevention of cardiovascular disease: systematic review and thematic synthesis of qualitative studies. BMJ Open. 2018;8(11):e021137.

28. Kelly A, Tymms K, Tunnicliffe DJ, Sumpton D, Perera C, Fallon K, et al. Patients' Attitudes and Experiences of Disease-Modifying Antirheumatic Drugs in Rheumatoid Arthritis and Spondyloarthritis: A Qualitative Synthesis. Arthritis Care Res (Hoboken). 2018;70(4):525-32.

29. Luker J, Murray C, Lynch E, Bernhardsson S, Shannon M, Bernhardt J. Carers' Experiences, Needs, and Preferences During Inpatient Stroke Rehabilitation: A Systematic Review of Qualitative Studies. Archives of Physical Medicine and Rehabilitation. 2017;98(9):1852-62.

30. Jamieson NJ, Hanson CS, Josephson MA, Gordon EJ, Craig JC, Halleck F, et al. Motivations, Challenges, and Attitudes to Self-management in Kidney Transplant Recipients: A Systematic Review of Qualitative Studies. American Journal of Kidney Diseases. 2016;67(3):461-78.

31. Whittle EL, Fogarty AS, Tugendrajch S, Player MJ, Christensen H, Wilhelm K, et al. Men, Depression, and Coping: Are We on the Right Path? Psychology of Men & Masculinity. 2015;16(4):426-38.

32. Luker J, Lynch E, Bernhardsson S, Bennett L, Bernhardt J. Stroke Survivors' Experiences of Physical Rehabilitation: A Systematic Review of Qualitative Studies. Archives of Physical Medicine and Rehabilitation. 2015;96(9):1698-708.

33. Kelly A, Niddrie F, Tunnicliffe DJ, Matus Gonzalez A, Hanson C, Jiang I, et al. Patients' attitudes and experiences of transition from paediatric to adult healthcare in rheumatology: a qualitative systematic review. Rheumatology (Oxford). 2020.

34. Ralph AF, Butow P, Hanson CS, Chadban SJ, Chapman JR, Craig JC, et al. Donor and Recipient Views on Their Relationship in Living Kidney Donation: Thematic Synthesis of Qualitative Studies. American Journal of Kidney Diseases. 2017;69(5):602-16.

35. Slade SC, Philip K, Morris ME. Frameworks for embedding a research culture in allied health practice: a rapid review. Health Res Policy Syst. 2018;16(1):29.

36. Lundby C, Graabaek T, Ryg J, Søndergaard J, Pottegård A, Nielsen DS. Health care professionals' attitudes towards deprescribing in older patients with limited life expectancy: A systematic review. Br J Clin Pharmacol. 2019;85(5):868-92.

37. McHale S, Astin F, Neubeck L, Dawkes S, Hanson CL. A systematic review and thematic synthesis exploring how a previous experience of physical activity influences engagement with cardiac rehabilitation. European Journal of Cardiovascular Nursing. 2020;19(1):31-43.

38. Matvienko-Sikar K, Kelly C, Sinnott C, McSharry J, Houghton C, Heary C, et al. Parental experiences and perceptions of infant complementary feeding: a qualitative evidence synthesis. Obesity Reviews. 2018;19(4):501-17.

39. Flemming K, Booth A, Hannes K, Cargo M, Noyes J. Cochrane Qualitative and Implementation Methods Group guidance series-paper 6: reporting guidelines for qualitative, implementation, and process evaluation evidence syntheses. Journal of Clinical Epidemiology. 2018;97:79-85.

40. O'Connor S, Hanlon P, O'Donnell CA, Garcia S, Glanville J, Mair FS. Understanding factors affecting patient and public engagement and recruitment to digital health interventions: a systematic review of qualitative studies. BMC Med Inform Decis Mak. 2016;16(1):120.

41. Luker JA, Bernhardsson S, Lynch E, Murray C, Hill OP, Bernhardt J. Carers' experiences, needs and preferences during inpatient stroke rehabilitation: a protocol for a systematic review of qualitative studies. Syst Rev. 2015;4:108.

42. Palmer SC, Gray H, Huria T, Lacey C, Beckert L, Pitama SG. Reported Māori consumer experiences of health systems and programs in qualitative research: a systematic review with meta-synthesis. Int J Equity Health. 2019;18(1):163.

43. Flores D, Leblanc N, Barroso J. Enroling and retaining human immunodeficiency virus (HIV) patients in their care: A metasynthesis of qualitative studies. Int J Nurs Stud. 2016;62:126-36.

44. Tanganhito DD, Bick D, Chang YS. Breastfeeding experiences and perspectives among women with postnatal depression: A qualitative evidence synthesis. Women and Birth. 2020;33(3):231-9.

45. Graham H, de Bell S, Flemming K, Sowden A, White P, Wright K. Older people's experiences of everyday travel in the urban environment: a thematic synthesis of qualitative studies in the United Kingdom. Ageing & Society. 2020;40(4):842-68.

46. Sellars M, Chung O, Nolte L, Tong A, Pond D, Fetherstonhaugh D, et al. Perspectives of people with dementia and carers on advance care planning and end-of-life care: A systematic review and thematic synthesis of qualitative studies. Palliat Med. 2019;33(3):274-90.

47. Koshoedo SA, Paul-Ebhohimhen VA, Jepson RG, Watson MC. Understanding the complex interplay of barriers to physical activity amongst black and minority ethnic groups in the United Kingdom: a qualitative synthesis using meta-ethnography. BMC Public Health. 2015;15:643.

48. Sumankuuro J, Crockett J, Wang S. Sociocultural barriers to maternity services delivery: a qualitative meta-synthesis of the literature. Public Health. 2018;157:77-85.

49. Harris JL, Booth A, Cargo M, Hannes K, Harden A, Flemming K, et al. Cochrane Qualitative and Implementation Methods Group guidance series-paper 2: methods for question formulation, searching, and protocol development for qualitative evidence synthesis. Journal of Clinical Epidemiology. 2018;97:39-48.

50. Sondaal SF, Browne JL, Amoakoh-Coleman M, Borgstein A, Miltenburg AS, Verwijs M, et al. Assessing the Effect of mHealth Interventions in Improving Maternal and Neonatal Care in Low- and Middle-Income Countries: A Systematic Review. PLoS One. 2016;11(5):e0154664.

51. Perez GM, Aznar CT, Bagnol B. LABIA MINORA ELONGATION AND ITS IMPLICATIONS ON THE HEALTH OF WOMEN: A SYSTEMATIC REVIEW. International Journal of Sexual Health. 2014;26(3):155-71.

52. Teasdale EJ, Leydon G, Fraser S, Roderick P, Taal MW, Tonkin-Crine S. Patients' Experiences After CKD Diagnosis: A Meta- ethnographic Study and Systematic Review. American Journal of Kidney Diseases. 2017;70(5):656-65.

53. Teng C, Loy CT, Sellars M, Pond D, Latt MD, Waite LM, et al. Making Decisions About Long-Term Institutional Care Placement Among People With Dementia and Their Caregivers: Systematic Review of Qualitative Studies. Gerontologist. 2020;60(4):E329-E46.

54. Tong A, Hanson CS, Chapman JR, Halleck F, Budde K, Josephson MA, et al. Suspended in a paradox'patient attitudes to wait-listing for kidney transplantation: systematic review and thematic synthesis of qualitative studies. Transplant International. 2015;28(7):771-87.

55. Nye E, Melendez-Torres GJ, Bonell C. Origins, methods and advances in qualitative meta-synthesis. Review of Education. 2016;4(1):57-79.

56. Tong A, Jesudason S, Craig JC, Winkelmayer WC. Perspectives on pregnancy in women with chronic kidney disease: systematic review of qualitative studies. Nephrology Dialysis Transplantation. 2015;30(4):652-61.

57. Atienzo EE, Kaltenthaler E, Baxter SK. Barriers and Facilitators to the Implementation of Interventions to Prevent Youth Violence in Latin America: A Systematic Review and Qualitative Evidence Synthesis. Trauma Violence & Abuse. 2018;19(4):420-30.

58. Waldecker A, Malpass A, King A, Ridd MJ. Written action plans for children with long-term conditions: A systematic review and synthesis of qualitative data. Health Expect. 2018;21(3):585-96.

59. Sweeney L, Clarke C, Wolverson E. The use of everyday technologies to enhance well-being and enjoyment for people living with dementia: A systematic literature review and narrative synthesis. Dementia-International Journal of Social Research and Practice.

60. Toye F, Seers K, Barker K. A meta-ethnography of patients' experiences of chronic pelvic pain: struggling to construct chronic pelvic pain as 'real'. Journal of Advanced Nursing. 2014;70(12):2713-27.

61. May CF. Discovering new areas of veterinary science through qualitative research interviews: introductory concepts for veterinarians. Australian Veterinary Journal. 2018;96(8):278-84.

62. Holkham L, Soundy A. The experience of informal caregivers of patients with motor neurone disease: A thematic synthesis. Palliative & Supportive Care. 2018;16(4):487-96.

Included reviews using neither COREQ or ENTREQ (n=1042)

1. Aagaard H, Hall EOC, Ludvigsen MS, Uhrenfeldt L, Fegran L. Parents' experiences of neonatal transfer. A meta-study of qualitative research 2000-2017. Nurs Inq. 2018;25(3):e12231.

2. Abaraogu UO, Ezenwankwo EF, Dall PM, Seenan CA. Living a burdensome and demanding life: A qualitative systematic review of the patients experiences of peripheral arterial disease. PLoS One. 2018;13(11):e0207456.

3. Abiodun RO, Daniels F, Pimmer C, Chipps J. Nurse graduates' experiences and support needs: A qualitative systematic review of South Africa's community service programme. Curationis. 2019;42(1):e1-e12.

4. Achterbergh L, Pitman A, Birken M, Pearce E, Sno H, Johnson S. The experience of loneliness among young people with depression: a qualitative meta-synthesis of the literature. BMC Psychiatry. 2020;20(1):415.

5. Adams AMN, Chamberlain D, Giles TM. The perceived and experienced role of the nurse unit manager in supporting the wellbeing of intensive care unit nurses: An integrative literature review. Aust Crit Care. 2019;32(4):319-29.

6. Afrouz R, Crisp BR, Taket A. Seeking Help in Domestic Violence Among Muslim Women in Muslim-Majority and Non-Muslim-Majority Countries: A Literature Review. Trauma Violence Abuse. 2020;21(3):551-66.

7. Agner J, Braun KL. Patient empowerment: A critique of individualism and systematic review of patient perspectives. Patient Educ Couns. 2018;101(12):2054-64.

8. Agudelo-Suárez AA, Gil-González D, Vives-Cases C, Love JG, Wimpenny P, Ronda-Pérez E. A metasynthesis of qualitative studies regarding opinions and perceptions about barriers and determinants of health services' accessibility in economic migrants. BMC Health Serv Res. 2012;12:461.

9. Ahmed S, Autrey J, Katz IT, Fox MP, Rosen S, Onoya D, et al. Why do people living with HIV not initiate treatment? A systematic review of qualitative evidence from low- and middle-income countries. Soc Sci Med. 2018;213:72-84.

10. Aitken M, de St Jorre J, Pagliari C, Jepson R, Cunningham-Burley S. Public responses to the sharing and linkage of health data for research purposes: a systematic review and thematic synthesis of qualitative studies. BMC Med Ethics. 2016;17(1):73.

11. Akther SF, Molyneaux E, Stuart R, Johnson S, Simpson A, Oram S. Patients' experiences of assessment and detention under mental health legislation: systematic review and qualitative meta-synthesis. BJPsych Open. 2019;5(3):e37.

12. Al Hamid A, Ghaleb M, Aljadhey H, Aslanpour Z. A systematic review of qualitative research on the contributory factors leading to medicine-related problems from the perspectives of adult patients with cardiovascular diseases and diabetes mellitus. BMJ Open. 2014;4(9):e005992.

13. Albert JS, Younas A, Sana S. Nursing students' ethical dilemmas regarding patient care: An integrative review. Nurse education today. 2020;88:104389.

14. Ali TS, Farhan R, Ayub M. Intimate partner violence against women in Pakistan: a review of qualitative research. Jpma. 2020;The Journal of the Pakistan Medical Association. 70(5):892-903.

15. Alsharaydeh EA, Alqudah M, Lee RLT, Chan SWC. Challenges, Coping, and Resilience Among Immigrant Parents Caring for a Child With a Disability: An Integrative Review. Journal of Nursing Scholarship. 2019;51(6):670-9.

16. Alspaugh A, Barroso J, Reibel M, Phillips S. Women's Contraceptive Perceptions, Beliefs, and Attitudes: An Integrative Review of Qualitative Research. J Midwifery Womens Health. 2020;65(1):64-84.

17. Alves E, Rodrigues C, Fraga S, Barros H, Silva S. Parents' views on factors that help or hinder breast milk supply in neonatal care units: systematic review. Arch Dis Child Fetal Neonatal Ed. 2013;98(6):F511-7.

18. Alves VLP, Carniel AQ, Costallat LTL, Turato ER. Meanings of the sickening process for patients with systemic lupus erythematosus: A review of the literature. Revista Brasileira de Reumatologia. 2015;55(6):522-7.

19. Ames HM, Glenton C, Lewin S. Parents' and informal caregivers' views and experiences of communication about routine childhood vaccination: a synthesis of qualitative evidence. Cochrane Database Syst Rev. 2017;2(2):Cd011787.

20. Ames HMR, Glenton C, Lewin S, Tamrat T, Akama E, Leon N. Clients' perceptions and experiences of targeted digital communication accessible via mobile devices for reproductive, maternal, newborn, child, and adolescent health: A qualitative evidence synthesis. Cochrane Database of Systematic Reviews. 2019;2019(10).

21. Ames HMR, Zuske M, King JD, Steinmann P, Bosch-Capblanch X. Community and Drug Distributor Perceptions and Experiences of Mass Drug Administration for the Elimination of Lymphatic Filariasis: A Rapid Review of Qualitative Research. Adv Parasitol. 2019;103:117-49.

22. Amsrud KE, Lyberg A, Severinsson E. Development of resilience in nursing students: A systematic qualitative review and thematic synthesis. Nurse Educ Pract. 2019;41:102621.

23. Anastas JW. What's the Story? Views of Pregnant Teens in Qualitative Research. Affil J Women Soc Work. 2017;32(2):133-70.

24. Andersen MF, Nielsen KM, Brinkmann S. Meta-synthesis of qualitative research on return to work among employees with common mental disorders. Scand J Work Environ Health. 2012;38(2):93-104.

25. Anderson AJ. A Qualitative Systematic Review of Youth Participatory Action Research Implementation in U.S. High Schools. Am J Community Psychol. 2020;65(1-2):242-57.

26. Anderson RJ, Bloch S, Armstrong M, Stone PC, Low JT. Communication between healthcare professionals and relatives of patients approaching the end-of-life: A systematic review of qualitative evidence. Palliat Med. 2019;33(8):926-41.

27. Andersson K, Bellon M, Walker R. Parents' experiences of their child's return to school following acquired brain injury (ABI): A systematic review of qualitative studies. Brain Inj. 2016;30(7):829-38.

28. Anderzén-Carlsson A, Lamy ZC, Tingvall M, Eriksson M. Parental experiences of providing skin-to-skin care to their newborn infant--part 2: a qualitative meta-synthesis. Int J Qual Stud Health Well-being. 2014;9:24907.

29. Andrade FMR, Simões Figueiredo A, Capelas ML, Charepe Z, Deodato S. Experiences of Homeless Families in Parenthood: A Systematic Review and Synthesis of Qualitative Evidence. Int J Environ Res Public Health. 2020;17(8).

30. Ang BH, Jennifer O, Chen WS, Lee SWH. Factors and challenges of driving reduction and cessation: A systematic review and meta-synthesis of qualitative studies on self-regulation. J Safety Res. 2019;69:101-8.

31. Annear MJ, Shimizu Y, Kidokoro T. Sports mega-event legacies and adult physical activity: A systematic literature review and research agenda. Eur J Sport Sci. 2019;19(5):671-85.

32. Anonymous. Ontario health technology assessment series: Perspectives of pregnant people and clinicians on noninvasive prenatal testing: A systematic review and qualitative meta-synthesis. Ontario Health Technology Assessment Series. 2019;19(5).

33. Appelgren M, Bahtsevani C, Persson K, Borglin G. Nurses' experiences of caring for patients with intellectual developmental disorders: a systematic review using a meta-ethnographic approach. BMC Nurs. 2018;17:51.

34. Arai L, Heawood A, Feder G, Howarth E, MacMillan H, Moore THM, et al. Hope, Agency, and the Lived Experience of Violence: A Qualitative Systematic Review of Children's Perspectives on Domestic Violence and Abuse. Trauma Violence Abuse. 2019:1524838019849582.

35. Arendts G, Quine S, Howard K. Decision to transfer to an emergency department from residential aged care: a systematic review of qualitative research. Geriatr Gerontol Int. 2013;13(4):825-33.

36. Armoogum J, Harcourt D, Foster C, Llewellyn A, McCabe CS. The experience of persistent pain in adult cancer survivors: A qualitative evidence synthesis. European journal of cancer care. 2019:e13192.

37. Arnold JL, Baker C. The role of mental health nurses in supporting young people's mental health: a review of the literature. Mental Health Review Journal. 2018;23(3):197-220.

38. Asgari Z, Naghavi A. Explaining Post-Traumatic Growth: Thematic Synthesis of Qualitative Research. Iran J Psychiat Clin Psychol. 2019;25(2):222-35.

39. Askew L, Fisher P, Beazley P. What are adult psychiatric inpatients' experience of seclusion: A systematic review of qualitative studies. J Psychiatr Ment Health Nurs. 2019;26(7-8):274-85.

40. Atherton K, Young B, Salmon P. Understanding the information needs of people with haematological cancers. A meta-ethnography of quantitative and qualitative research. European Journal of Cancer Care. 2017.

41. Atkinson JA, Vallely A, Fitzgerald L, Whittaker M, Tanner M. The architecture and effect of participation: a systematic review of community participation for communicable disease control and elimination. Implications for malaria elimination. Malar J. 2011;10:225.

42. Atwal A, Spiliotopoulou G, Plastow N, McIntyre A, McKay EA. Older adults' experiences of occupational therapy predischarge home visits: A systematic thematic synthesis of qualitative research. British Journal of Occupational Therapy. 2012;75(3):118-27.

43. Aung SHH, White K, Bloomfield J. The Experiences and the Needs of Caregivers of Patients With Head and Neck Cancer: An Integrative Review. Cancer nursing. 2020;12.

44. Bach-Mortensen AM, Lange BCL, Montgomery P. Barriers and facilitators to implementing evidence-based interventions among third sector organisations: a systematic review. Implement Sci. 2018;13(1):103.

45. Backes BL, Fedina L, Holmes JL. The Criminal Justice System Response to Intimate Partner Stalking: a Systematic Review of Quantitative and Qualitative Research. J Fam Violence.14.

46. Backhouse A, Richards DA, McCabe R, Watkins R, Dickens C. Stakeholders perspectives on the key components of community-based interventions coordinating care in dementia: a qualitative systematic review. BMC Health Serv Res. 2017;17(1):767.

47. Bagnasco A, Hayter M, Rossi S, Zanini MP, Pellegrini R, Aleo G, et al. Experiences of participating in intergenerational interventions in older people's care settings: A systematic review and meta-synthesis of qualitative literature. J Adv Nurs. 2020;76(1):22-33.

48. Baker N, Lawn S, Gordon SJ, George S. Older Adults' Experiences of Goals in Health: A Systematic Review and Metasynthesis. J Appl Gerontol. 2020:733464820918134.

49. Balaam MC, Akerjordet K, Lyberg A, Kaiser B, Schoening E, Fredriksen AM, et al. A qualitative review of migrant women's perceptions of their needs and experiences related to pregnancy and childbirth. J Adv Nurs. 2013;69(9):1919-30.

50. Baldwin S, Malone M, Sandall J, Bick D. Mental health and wellbeing during the transition to fatherhood: a systematic review of first time fathers' experiences. JBI Database System Rev Implement Rep. 2018;16(11):2118-91.

51. Balmer C, Griffiths F, Dunn J. A qualitative systematic review exploring lay understanding of cancer by adults without a cancer diagnosis. J Adv Nurs. 2014;70(8):1688-701.

52. Bamidele O, McGarvey H, Lagan BM, Ali N, Chinegwundoh Mbe F, Parahoo K, et al. Life after prostate cancer: A systematic literature review and thematic synthesis of the post-treatment experiences of Black African and Black Caribbean men. Eur J Cancer Care (Engl). 2018;27(1).

53. Bangpan M, Operario D. Understanding the role of family on sexual-risk decisions of young women: a systematic review. AIDS Care. 2012;24(9):1163-72.

54. Bareham BK, Kaner E, Spencer L, Hanratty B. Health and social care providers' perspectives of older people's drinking: A systematic review and thematic synthesis of qualitative studies. Age Ageing. 2020;49(3):453-67.

55. Bareham BK, Kaner E, Spencer LP, Hanratty B. Drinking in later life: a systematic review and thematic synthesis of qualitative studies exploring older people's perceptions and experiences. Age Ageing. 2019;48(1):134-46.

56. Barker KL, Toye F, Lowe CJ. A qualitative systematic review of patients' experience of osteoporosis using meta-ethnography. Arch Osteoporos. 2016;11(1):33.

57. Barlow T, Griffin D, Barlow D, Realpe A. Patients' decision making in total knee arthroplasty: a systematic review of qualitative research. Bone Joint Res. 2015;4(10):163-9.

58. Bartoș SE, Hegarty P. Negotiating Theory When Doing Practice: A Systematic Review of Qualitative Research on Interventions to Reduce Homophobia. J Homosex. 2019;66(9):1262-86.

59. Bastemeijer CM, Voogt L, van Ewijk JP, Hazelzet JA. What do patient values and preferences mean? A taxonomy based on a systematic review of qualitative papers. Patient Educ Couns. 2017;100(5):871-81.

60. Bateman L, Jones C, Jomeen J. A Narrative Synthesis of Women's Out-of-Body Experiences During Childbirth. Journal of Midwifery and Women's Health. 2017;62(4):442-51.

61. Bauer M, Haesler E, Fetherstonhaugh D. Organisational enablers and barriers to the recognition of sexuality in aged care: A systematic review. J Nurs Manag. 2019;27(4):858-68.

62. Baxter S, Blank L, Guillaume L, Squires H, Payne N. Views of contraceptive service delivery to young people in the UK: a systematic review and thematic synthesis. J Fam Plann Reprod Health Care. 2011;37(2):71-84.

63. Baxter S, Blank L, Guillaume L, Squires H, Payne N. Views regarding the use of contraception amongst young people in the UK: a systematic review and thematic synthesis. Eur J Contracept Reprod Health Care. 2011;16(3):149-60.

64. Beardon S, Patel K, Davies B, Ward H. Informal carers' perspectives on the delivery of acute hospital care for patients with dementia: a systematic review. BMC Geriatr. 2018;18(1):23.

65. Benelhaj NB, Hutchinson A, Maraveyas AM, Seymour JD, Ilyas MW, Johnson MJ. Cancer patients' experiences of living with venous thromboembolism: A systematic review and qualitative thematic synthesis. Palliat Med. 2018;32(5):1010-20.

66. Bennett KF, Waller J, Ryan M, Bailey JV, Marlow LAV. Concerns about disclosing a high-risk cervical human papillomavirus (HPV) infection to a sexual partner: a systematic review and thematic synthesis. BMJ Sex Reprod Health. 2020.

67. Bennett SE, Walsh N, Moss T, Palmer S. The lived experience of Joint Hypermobility and Ehlers-Danlos Syndromes: a systematic review and thematic synthesis. Physical Therapy Reviews. 2019;24(1-2):12-28.

68. Bennion AE, Molassiotis A. Qualitative research into the symptom experiences of adult cancer patients after treatments: a systematic review and meta-synthesis. Support Care Cancer. 2013;21(1):9-25.

69. Bennion AE, Shaw RL, Gibson JM. What do we know about the experience of age related macular degeneration? A systematic review and meta-synthesis of qualitative research. Soc Sci Med. 2012;75(6):976-85.

70. Berg RC, Taraldsen S, Said MA, Sørbye IK, Vangen S. Reasons for and Experiences With Surgical Interventions for Female Genital Mutilation/Cutting (FGM/C): A Systematic Review. J Sex Med. 2017;14(8):977-90.

71. Bergs J, Lambrechts F, Simons P, Vlayen A, Marneffe W, Hellings J, et al. Barriers and facilitators related to the implementation of surgical safety checklists: a systematic review of the qualitative evidence. BMJ Qual Saf. 2015;24(12):776-86.

72. Bhaumik S, Hunter K, Matzopoulos R, Prinsloo M, Ivers RQ, Peden M. Facilitators and barriers to child restraint use in motor vehicles: a qualitative evidence synthesis. Inj Prev. 2020.

73. Bilby C, Brooks-Gordon B, Wells H. A systematic review of psychological interventions for sexual offenders II: Quasi-experimental and qualitative data. Journal of Forensic Psychiatry and Psychology. 2006;17(3):467-84.

74. Bjerrum MB, Pedersen PU, Larsen P. Living with symptoms of attention deficit hyperactivity disorder in adulthood: a systematic review of qualitative evidence. JBI Database System Rev Implement Rep. 2017;15(4):1080-153.

75. Bjornestad J, Lavik KO, Davidson L, Hjeltnes A, Moltu C, Veseth M. Antipsychotic treatment - a systematic literature review and meta-analysis of qualitative studies. Journal of mental health (Abingdon, England). 2019:1-11.

76. Blakeley C, Smith DM, Johnstone ED, Wittkowski A. Parental decision-making following a prenatal diagnosis that is lethal, life-limiting, or has long term implications for the future child and family: a meta-synthesis of qualitative literature. BMC Med Ethics. 2019;20(1):56.

77. Blatchford L, Cook J. Patient Perspectives about Mild Cognitive Impairment: A Systematic Review. Clin Gerontol. 2020:1-13.

78. Blower S, Swallow V, Maturana C, Stones S, Phillips R, Dimitri P, et al. Children and young people's concerns and needs relating to their use of health technology to self-manage long-term conditions: A scoping review. Archives of Disease in Childhood. 2020.

79. Boyce MB, Browne JP, Greenhalgh J. The experiences of professionals with using information from patient-reported outcome measures to improve the quality of healthcare: a systematic review of qualitative research. BMJ Qual Saf. 2014;23(6):508-18.

80. Boyle C. 'What is the impact of birth family contact on children in adoption and long-term foster care?' A systematic review. Child Fam Soc Work. 2017;22:22-33.

81. Boyle J, Vukicevic M, Koklanis K, Itsiopoulos C. Experiences of patients undergoing anti-VEGF treatment for neovascular age-related macular degeneration: a systematic review. Psychol Health Med. 2015;20(3):296-310.

82. Bradley C, McGowan J, Michelson D. How Does Homelessness Affect Parenting Behaviour? A Systematic Critical Review and Thematic Synthesis of Qualitative Research. Clin Child Fam Psychol Rev. 2018;21(1):94-108.

83. Bradley S, McCourt C, Rayment J, Parmar D. Disrespectful intrapartum care during facility-based delivery in sub-Saharan Africa: A qualitative systematic review and thematic synthesis of women's perceptions and experiences. Soc Sci Med. 2016;169:157-70.

84. Breeksema JJ, Niemeijer AR, Krediet E, Vermetten E, Schoevers RA. Psychedelic Treatments for Psychiatric Disorders: A Systematic Review and Thematic Synthesis of Patient Experiences in Qualitative Studies. CNS Drugs. 2020;34(9):925-46.

85. Bridges J, Collins P, Flatley M, Hope J, Young A. Older people's experiences in acute care settings: Systematic review and synthesis of qualitative studies. Int J Nurs Stud. 2020;102:103469.

86. Brooke J, Ojo O. Contemporary views on dementia as witchcraft in sub-Saharan Africa: A systematic literature review. J Clin Nurs. 2020;29(1-2):20-30.

87. Brookfield S, Dean J, Forrest C, Jones J, Fitzgerald L. Barriers to Accessing Sexual Health Services for Transgender and Male Sex Workers: A Systematic Qualitative Meta-summary. AIDS Behav. 2020;24(3):682-96.

88. Brossard Saxell T, Ingvert M, Lethin C. Facilitators for person-centred care of inpatients with dementia: A meta-synthesis of registered nurses' experiences. Dementia. 2019.

89. Brown I, Gould J. Decisions about weight management: a synthesis of qualitative studies of obesity. Clin Obes. 2011;1(2-3):99-109.

90. Brown S, Lhussier M, Dalkin SM, Eaton S. Care Planning: What Works, for Whom, and in What Circumstances? A Rapid Realist Review. Qual Health Res. 2018;28(14):2250-66.

91. Brundisini F, Giacomini M, DeJean D, Vanstone M, Winsor S, Smith A. Chronic disease patients' experiences with accessing health care in rural and remote areas: a systematic review and qualitative meta-synthesis. Ont Health Technol Assess Ser. 2013;13(15):1-33.

92. Brunero S, Ramjan LM, Salamonson Y, Nicholls D. Generalist health professional's interactions with consumers who have a mental illness in nonmental health settings: A systematic review of the qualitative research. Int J Ment Health Nurs. 2018;27(6):1634-49.

93. Bulthuis SE, Kok MC, Raven J, Dieleman MA. Factors influencing the scale-up of public health interventions in low- and middle-income countries: a qualitative systematic literature review. Health Policy Plan. 2020;35(2):219-34.

94. Bunn F, Goodman C, Sworn K, Rait G, Brayne C, Robinson L, et al. Psychosocial factors that shape patient and carer experiences of dementia diagnosis and treatment: a systematic review of qualitative studies. PLoS Med. 2012;9(10):e1001331.

95. Burbeck R, Candy B, Low J, Rees R. Understanding the role of the volunteer in specialist palliative care: a systematic review and thematic synthesis of qualitative studies. BMC Palliat Care. 2014;13(1):3.

96. Burgstaller M, Mayer H, Schiess C, Saxer S. Experiences and needs of relatives of people with dementia in acute hospitals-A meta-synthesis of qualitative studies. Journal of Clinical Nursing. 2018;27(3-4):502-15.

97. Burke S, Wurz A, Bradshaw A, Saunders S, West MA, Brunet J. Physical activity and quality of life in cancer survivors: A meta-synthesis of qualitative research. Cancers. 2017;9(5).

98. Burns T, Fernandez R, Stephens M. The experiences of adults who are on dialysis and waiting for a renal transplant from a deceased donor: a systematic review. JBI Database System Rev Implement Rep. 2015;13(2):169-211.

99. Burrell A, Selman LE. How do Funeral Practices impact Bereaved Relatives' Mental Health, Grief and Bereavement? A Mixed Methods Review with Implications for COVID-19. Omega. 2020:30222820941296.

100. Burton C, Doyle E, Humber K, Rouxel C, Worner S, Colman R, et al. The biopsychosocial barriers and enablers to being physically active following childbirth: a systematic literature review. Physical Therapy Reviews. 2019;24(3-4):143-55.

101. Busch IM, Moretti F, Travaini G, Wu AW, Rimondini M. Humanization of Care: Key Elements Identified by Patients, Caregivers, and Healthcare Providers. A Systematic Review. Patient. 2019;12(5):461-74.

102. Butenko S, Lockwood C, McArthur A. Patient experiences of partnering with healthcare professionals for hand hygiene compliance: a systematic review. JBI Database System Rev Implement Rep. 2017;15(6):1645-70.

103. Buus N, Hoeck B, Hamilton BE. Nurses' shift reports: a systematic literature search and critical review of qualitative field studies. J Clin Nurs. 2017;26(19-20):2891-906.

104. Bylicki O, Didier M, Riviere F, Margery J, Grassin F, Chouaid C. Lung cancer and end-of-life care: a systematic review and thematic synthesis of aggressive inpatient care. BMJ Support Palliat Care. 2019;9(4):413-24.

105. Byron C, Cornally N, Burton A, Savage E. Challenges of living with and managing inflammatory bowel disease: A meta-synthesis of patients' experiences. Journal of Clinical Nursing. 2020;29(3-4):305-19.

106. Byrth J, Aromataris E. Health professionals' perceptions and experiences of open disclosure: A systematic review of qualitative evidence. JBI Database of Systematic Reviews and Implementation Reports. 2014;12(5):237-318.

107. Cabote CJ, Bramble M, McCann D. Family Caregivers' Experiences of Caring for a Relative With Younger Onset Dementia: A Qualitative Systematic Review. J Fam Nurs. 2015;21(3):443-68.

108. Cairns VA, Reid GS, Murray C. Family members' experience of seeking help for first-episode psychosis on behalf of a loved one: a meta-synthesis of qualitative research. Early Interv Psychiatry. 2015;9(3):185-99.

109. Campbell F, Johnson M, Messina J, Guillaume L, Goyder E. Behavioural interventions for weight management in pregnancy: a systematic review of quantitative and qualitative data. BMC Public Health. 2011;11:491.

110. Campbell K, Coleman-Haynes T, Bowker K, Cooper SE, Connelly S, Coleman T. Factors influencing the uptake and use of nicotine replacement therapy and e-cigarettes in pregnant women who smoke: a qualitative evidence synthesis. Cochrane Database Syst Rev. 2020;5(5):Cd013629.

111. Campbell M, Thomson H, Fenton C, Gibson M. Lone parents, health, wellbeing and welfare to work: a systematic review of qualitative studies. BMC Public Health. 2016;16:188.

112. Campbell R, Pound P, Pope C, Britten N, Pill R, Morgan M, et al. Evaluating meta-ethnography: a synthesis of qualitative research on lay experiences of diabetes and diabetes care. Soc Sci Med. 2003;56(4):671-84.

113. Campbell-Enns HJ, Woodgate RL. The psychosocial experiences of women with breast cancer across the lifespan: a systematic review. Psychooncology. 2017;26(11):1711-21.

114. Capilla-Diaz C, Duran-Lopez MI, Martinez-Guerrero JM, Reina-Leal LM, Gomez-Urquiza JL, Galvez-Gonzalez M, et al. Bibliometric analysis of qualitative research on patients' experiences of intestinal stoma published between 2002 - 2018. Journal of advanced nursing. 2020;76(5):1182-91.

115. Capper T, Muurlink O, Williamson M. Midwifery students' experiences of bullying and workplace violence: A systematic review. Midwifery. 2020;90:102819.

116. Cardona-Arias JA, Salas-Zapata W, Carmona-Fonseca J. Systematic review of qualitative studies about malaria in Colombia. Heliyon. 2020;6(5):11.

117. Carey MC, Kent B, Latour JM. Experiences of undergraduate nursing students in peer assisted learning in clinical practice: a qualitative systematic review. JBI Database System Rev Implement Rep. 2018;16(5):1190-219.

118. Carlsen B, Glenton C. The swine flu vaccine, public attitudes, and researcher interpretations: a systematic review of qualitative research. BMC Health Serv Res. 2016;16:203.

119. Carlsen B, Glenton C, Pope C. Thou shalt versus thou shalt not: a meta-synthesis of GPs' attitudes to clinical practice guidelines. Br J Gen Pract. 2007;57(545):971-8.

120. Carlson L, Hutton S, Priest H, Melia Y. Reunification of looked-after children with their birth parents in the United Kingdom: A literature review and thematic synthesis. Child and Family Social Work. 2020;25(1):192-205.

121. Carrier J, Edwards D, Harden J. Men's perceptions of the impact of the physical consequences of a radical prostatectomy on their quality of life: a qualitative systematic review. JBI Database System Rev Implement Rep. 2018;16(4):892-972.

122. Carroll C, Lloyd-Jones M, Cooke J, Owen J. Reasons for the use and non-use of school sexual health services: a systematic review of young people's views. J Public Health (Oxf). 2012;34(3):403-10.

123. Carstensen K, Lou S, Groth Jensen L, Konstantin Nissen N, Ortenblad L, Pfau M, et al. Psychiatric service users' experiences of emergency departments: a CERQual review of qualitative studies. Nordic Journal of Psychiatry. 2017;71(4):315-23.

124. Carver H, Ring N, Miler J, Parkes T. What constitutes effective problematic substance use treatment from the perspective of people who are homeless? A systematic review and meta-ethnography. Harm Reduct J. 2020;17(1):10.

125. Cassim S, Chepulis L, Keenan R, Kidd J, Firth M, Lawrenson R. Patient and carer perceived barriers to early presentation and diagnosis of lung cancer: a systematic review. BMC Cancer. 2019;19(1):25.

126. Castelino F, Prabhu M, Pai MS, Kamath A, Mohapatra AK, Devi ES. Lived experiences of patients with chronic obstructive pulmonary diseases (COPD)-qualitative review. Indian Journal of Public Health Research and Development. 2018;9(4):262-5.

127. Cavers D, Habets L, Cunningham-Burley S, Watson E, Banks E, Campbell C. Living with and beyond cancer with comorbid illness: a qualitative systematic review and evidence synthesis. J Cancer Surviv. 2019;13(1):148-59.

128. Cernat A, De Freitas C, Majid U, Trivedi F, Higgins C, Vanstone M. Facilitating informed choice about non-invasive prenatal testing (NIPT): a systematic review and qualitative meta-synthesis of women's experiences. BMC Pregnancy Childbirth. 2019;19(1):27.

129. Chakrabarti S. Treatment Attitudes and Adherence Among Patients with Bipolar Disorder: A Systematic Review of Quantitative and Qualitative Studies. Harv Rev Psychiatry. 2019;27(5):290-302.

130. Chang YS, Glaria AA, Davie P, Beake S, Bick D. Breastfeeding experiences and support for women who are overweight or obese: A mixed-methods systematic review. Matern Child Nutr. 2020;16(1):e12865.

131. Charlton K, Murray CM, Kumar S. Perspectives of older people about contingency planning for falls in the community: A qualitative meta-synthesis. PLoS ONE. 2017;12(5).

132. Chemlal S, Russo G. Why do they take the risk? A systematic review of the qualitative literature on informal sector abortions in settings where abortion is legal. BMC Womens Health. 2019;19(1):55.

133. Chernomas WM, Rieger KL, Karpa JV, Clarke DE, Marchinko S, Demczuk L. Young women's experiences of psychotic illness: a systematic review of qualitative research. JBI Database System Rev Implement Rep. 2017;15(3):694-737.

134. Child S, Goodwin V, Garside R, Jones-Hughes T, Boddy K, Stein K. Factors influencing the implementation of fall-prevention programmes: a systematic review and synthesis of qualitative studies. Implement Sci. 2012;7:91.

135. Chivers Seymour K, Addington-Hall J, Lucassen AM, Foster CL. What facilitates or impedes family communication following genetic testing for cancer risk? A systematic review and meta-synthesis of primary qualitative research. J Genet Couns. 2010;19(4):330-42.

136. Choo PY, Tan-Ho G, Dutta O, Patinadan PV, Ho AHY. Reciprocal Dynamics of Dignity in End-of-Life Care: A Multiperspective Systematic Review of Qualitative and Mixed Methods Research. Am J Hosp Palliat Care. 2020;37(5):385-98.

137. Christensen LF, Moller AM, Hansen JP, Nielsen CT, Gildberg FA. Patients' and providers' experiences with video consultations used in the treatment of older patients with unipolar depression: A systematic review. J Psychiatr Ment Health Nurs. 2020;27(3):258-71.

138. Chung VC, Ma PH, Lau CH, Wong SY, Yeoh EK, Griffiths SM. Views on traditional Chinese medicine amongst Chinese population: a systematic review of qualitative and quantitative studies. Health Expect. 2014;17(5):622-36.

139. Clark AM, King-Shier KM, Spaling MA, Duncan AS, Stone JA, Jaglal SB, et al. Factors influencing participation in cardiac rehabilitation programmes after referral and initial attendance: qualitative systematic review and meta-synthesis. Clin Rehabil. 2013;27(10):948-59.

140. Clark AM, King-Shier KM, Thompson DR, Spaling MA, Duncan AS, Stone JA, et al. A qualitative systematic review of influences on attendance at cardiac rehabilitation programs after referral. Am Heart J. 2012;164(6):835-45.e2.

141. Clarke C, Lumbard D, Sambrook S, Kerr K. What does recovery mean to a forensic mental health patient? A systematic review and narrative synthesis of the qualitative literature. Journal of Forensic Psychiatry and Psychology. 2016;27(1):38-54.

142. Clarke G, Harrison K, Holland A, Kuhn I, Barclay S. How are treatment decisions made about artificial nutrition for individuals at risk of lacking capacity? A systematic literature review. PLoS One. 2013;8(4):e61475.

143. Cleary M, Kornhaber R, Sayers J, Gray R. Mental health nurse prescribing: A qualitative, systematic review. Int J Ment Health Nurs. 2017;26(6):541-53.

144. Clews C, Church S, Ekberg M. Women and waterbirth: A systematic meta-synthesis of qualitative studies. Women Birth. 2019.

145. Clifford BK, Mizrahi D, Sandler CX, Barry BK, Simar D, Wakefield CE, et al. Barriers and facilitators of exercise experienced by cancer survivors: a mixed methods systematic review. Support Care Cancer. 2018;26(3):685-700.

146. Cohn I, Raman J, Sui Z. Patient motivations and expectations prior to bariatric surgery: A qualitative systematic review. Obes Rev. 2019;20(11):1608-18.

147. Coleman SJ, Stevelink SAM, Hatch SL, Denny JA, Greenberg N. Stigma-related barriers and facilitators to help seeking for mental health issues in the armed forces: a systematic review and thematic synthesis of qualitative literature. Psychol Med. 2017;47(11):1880-92.

148. Colvin CJ, de Heer J, Winterton L, Mellenkamp M, Glenton C, Noyes J, et al. A systematic review of qualitative evidence on barriers and facilitators to the implementation of task-shifting in midwifery services. Midwifery. 2013;29(10):1211-21.

149. Colvin CJ, Smith HJ, Swartz A, Ahs JW, de Heer J, Opiyo N, et al. Understanding careseeking for child illness in sub-Saharan Africa: a systematic review and conceptual framework based on qualitative research of household recognition and response to child diarrhoea, pneumonia and malaria. Soc Sci Med. 2013;86:66-78.

150. Connell J, Brazier J, O'Cathain A, Lloyd-Jones M, Paisley S. Quality of life of people with mental health problems: a synthesis of qualitative research. Health Qual Life Outcomes. 2012;10:138.

151. Connolly JA, Joly LE. Outreach with street-involved youth: a quantitative and qualitative review of the literature. Clin Psychol Rev. 2012;32(6):524-34.

152. Cook DJ, Meade MO, Perry AG. Qualitative studies on the patient's experience of weaning from mechanical ventilation. Chest. 2001;120(6 SUPPL.):469S-73S.

153. Cooper C, Burden ST, Cheng H, Molassiotis A. Understanding and managing cancer-related weight loss and anorexia: insights from a systematic review of qualitative research. J Cachexia Sarcopenia Muscle. 2015;6(1):99-111.

154. Cosco TD, Prina AM, Perales J, Stephan BC, Brayne C. Lay perspectives of successful ageing: a systematic review and meta-ethnography. BMJ Open. 2013;3(6).

155. Costi L, Lockwood C, Munn Z, Jordan Z. Women's experience of diabetes and diabetes management in pregnancy: A systematic review of qualitative literature. JBI Database of Systematic Reviews and Implementation Reports. 2014;12(1):176-280.

156. Coulman KD, MacKichan F, Blazeby JM, Owen-Smith A. Patient experiences of outcomes of bariatric surgery: a systematic review and qualitative synthesis. Obes Rev. 2017;18(5):547-59.

157. Cowley A, Evans C, Bath-Hextall F, Cooper J. Patient, nursing and medical staff experiences and perceptions of the care of people with palliative esophagogastric cancer: a systematic review of the qualitative evidence. JBI Database System Rev Implement Rep. 2016;14(10):134-66.

158. Cronin T, Sheppard J, de Wildt G. Health-seeking behaviour for schistosomiasis: a systematic review of qualitative and quantitative literature. Pan Afr Med J. 2013;16:130.

159. Crookall R, Fowler G, Wood C, Slade P. A systematic mixed studies review of women's experiences of perineal trauma sustained during childbirth. J Adv Nurs. 2018.

160. Cross AJ, Garip G, Sheffield D. The psychosocial impact of caregiving in dementia and quality of life: a systematic review and meta-synthesis of qualitative research. Psychol Health. 2018;33(11):1321-42.

161. Crowe M, Gillon D, Jordan J, McCall C. Older peoples' strategies for coping with chronic non-malignant pain: A qualitative meta-synthesis. Int J Nurs Stud. 2017;68:40-50.

162. Crowe M, Whitehead L, Seaton P, Jordan J, McCall C, Maskill V, et al. Qualitative meta-synthesis: The experience of chronic pain across conditions. Journal of Advanced Nursing. 2016.

163. Cullen DL, Stiffler D. Long-term oxygen therapy: Review from the patientsg perspective. Chronic Respiratory Disease. 2009;6(3):141-7.

164. Currie K, Melone L, Stewart S, King C, Holopainen A, Clark AM, et al. Understanding the patient experience of health care-associated infection: A qualitative systematic review. Am J Infect Control. 2018;46(8):936-42.

165. Currie K, Strachan PH, Spaling M, Harkness K, Barber D, Clark AM. The importance of interactions between patients and healthcare professionals for heart failure self-care: A systematic review of qualitative research into patient perspectives. Eur J Cardiovasc Nurs. 2015;14(6):525-35.

166. D'Cruz K, Douglas J, Serry T. Personal narrative approaches in rehabilitation following traumatic brain injury: A synthesis of qualitative research. Neuropsychol Rehabil. 2019;29(7):985-1004.

167. Dadouch R, Hall C, Du Mont J, D'Souza R. Obesity in Pregnancy - Patient-Reported Outcomes in Qualitative Research: A Systematic Review. J Obstet Gynaecol Can. 2020;42(8):1001-11.

168. Dahlberg EE, Hamilton SJ, Hamid F, Thompson SC. Indigenous Australians Perceptions' of Physical Activity: A Qualitative Systematic Review. Int J Environ Res Public Health. 2018;15(7).

169. Davis K, White S, Stephenson M. The influence of workplace culture on nurses' learning experiences: a systematic review of qualitative evidence. JBI Database System Rev Implement Rep. 2016;14(6):274-346.

170. Daykin N, Mansfield L, Meads C, Gray K, Golding A, Tomlinson A, et al. The role of social capital in participatory arts for wellbeing: findings from a qualitative systematic review. Arts Health. 2020:1-24.

171. de Haan E. A systematic review of qualitative studies in workplace and executive coaching: The emergence of a body of research. Consulting Psychology Journal. 2019;71(4):227-48.

172. De Ruysscher C, Vandevelde S, Vanderplasschen W, De Maeyer J, Vanheule S. The Concept of Recovery as Experienced by Persons with Dual Diagnosis: A Systematic Review of Qualitative Research From a First-Person Perspective. J Dual Diagn. 2017;13(4):264-79.

173. de São José J, Barros R, Samitca S, Teixeira A. Older persons' experiences and perspectives of receiving social care: a systematic review of the qualitative literature. Health Soc Care Community. 2016;24(1):1-11.

174. de Sousa Pinto JM, Martín-Nogueras AM, Morano MT, Macêdo TE, Arenillas JI, Troosters T. Chronic obstructive pulmonary disease patients' experience with pulmonary rehabilitation: a systematic review of qualitative research. Chron Respir Dis. 2013;10(3):141-57.

175. de Vos JA, LaMarre A, Radstaak M, Bijkerk CA, Bohlmeijer ET, Westerhof GJ. Identifying fundamental criteria for eating disorder recovery: a systematic review and qualitative meta-analysis. J Eat Disord. 2017;5:34.

176. Debbi S, Elisa P, Nigel B, Dan P, Eva R. Factors influencing household uptake of improved solid fuel stoves in low- and middle-income countries: a qualitative systematic review. Int J Environ Res Public Health. 2014;11(8):8228-50.

177. Degrie L, Gastmans C, Mahieu L, Dierckx de Casterlé B, Denier Y. "How do ethnic minority patients experience the intercultural care encounter in hospitals? a systematic review of qualitative research". BMC Med Ethics. 2017;18(1):2.

178. DeJean D, Giacomini M, Vanstone M, Brundisini F. Patient experiences of depression and anxiety with chronic disease: a systematic review and qualitative meta-synthesis. Ont Health Technol Assess Ser. 2013;13(16):1-33.

179. Demain S, Gonçalves AC, Areia C, Oliveira R, Marcos AJ, Marques A, et al. Living with, managing and minimising treatment burden in long term conditions: a systematic review of qualitative research. PLoS One. 2015;10(5):e0125457.

180. Denford S, van Beurden S, O'Halloran P, Williams CA. Barriers and facilitators to physical activity among children, adolescents, and young adults with cystic fibrosis: a systematic review and thematic synthesis of qualitative research. BMJ Open. 2020;10(2):e035261.

181. Dennehy R, Meaney S, Walsh KA, Sinnott C, Cronin M, Arensman E. Young people's conceptualizations of the nature of cyberbullying: A systematic review and synthesis of qualitative research. Aggression and Violent Behavior. 2020;51 (no pagination).

182. Dennison RA, Fox RA, Ward RJ, Griffin SJ, Usher-Smith JA. Women's views on screening for Type 2 diabetes after gestational diabetes: a systematic review, qualitative synthesis and recommendations for increasing uptake. Diabet Med. 2020;37(1):29-43.

183. Dennison RA, Ward RJ, Griffin SJ, Usher-Smith JA. Women's views on lifestyle changes to reduce the risk of developing Type 2 diabetes after gestational diabetes: a systematic review, qualitative synthesis and recommendations for practice. Diabet Med. 2019;36(6):702-17.

184. Denny E, Khan KS. Systematic reviews of qualitative evidence: what are the experiences of women with endometriosis? J Obstet Gynaecol. 2006;26(6):501-6.

185. Glenton C, Colvin CJ, Carlsen B, Swartz A, Lewin S, Noyes J, et al. Barriers and facilitators to the implementation of lay health worker programmes to improve access to maternal and child health: qualitative evidence synthesis. Cochrane Database Syst Rev. 2013;2013(10):Cd010414.

186. Godfrey CM, Harrison MB, Lang A, Macdonald M, Leung T, Swab M. Homecare safety and medication management with older adults: A scoping review of the quantitative and qualitative evidence. JBI Database of Systematic Reviews and Implementation Reports. 2013;11(7):82-130.

187. Goldsmith MR, Bankhead CR, Austoker J. Synthesising quantitative and qualitative research in evidence-based patient information. J Epidemiol Community Health. 2007;61(3):262-70.

188. Gonsalves CA, McGannon KR, Schinke RJ, Pegoraro A. Mass media narratives of women's cardiovascular disease: a qualitative meta-synthesis. Health Psychology Review. 2017;11(2):164-78.

189. Goodwin J, Savage E, Horgan A. Adolescents' and Young Adults' Beliefs about Mental Health Services and Care: A Systematic Review. Arch Psychiatr Nurs. 2016;30(5):636-44.

190. Graham B, Endacott R, Smith JE, Latour JM. 'They do not care how much you know until they know how much you care': a qualitative meta-synthesis of patient experience in the emergency department. Emerg Med J. 2019;36(6):355-63.

191. Graham H, de Bell S, Flemming K, Sowden A, White P, Wright K. The experiences of everyday travel for older people in rural areas: A systematic review of UK qualitative studies. Journal of Transport & Health. 2018;11:141-52.

192. Graham H, Flemming K, Fox D, Heirs M, Sowden A. Cutting down: insights from qualitative studies of smoking in pregnancy. Health Soc Care Community. 2014;22(3):259-67.

193. Graham H, McDermott E. Qualitative research and the evidence base of policy: Insights from studies of teenage mothers in the UK. J Soc Policy. 2006;35:21-37.

194. Graham MR, Tierney S, Chisholm A, Fox JRE. The lived experience of working with people with eating disorders: A meta-ethnography. Int J Eat Disord. 2020;53(3):422-41.

195. Graham N, Nye C, Mandy A, Clarke C, Morriss-Roberts C. The meaning of play for children and young people with physical disabilities: A systematic thematic synthesis. Child Care Health Dev. 2018;44(2):173-82.

196. Graham R, Masters-Awatere B. Experiences of Māori of Aotearoa New Zealand's public health system: a systematic review of two decades of published qualitative research. Aust N Z J Public Health. 2020;44(3):193-200.

197. Grant JS, Graven LJ. Problems experienced by informal caregivers of individuals with heart failure: An integrative review. International Journal of Nursing Studies. 2018;80:41-66.

198. Grant L, Seiding Larsen L, Burrows K, Belsito DV, Weisshaar E, Diepgen T, et al. Development of a Conceptual Model of Chronic Hand Eczema (CHE) Based on Qualitative Interviews with Patients and Expert Dermatologists. Advances in Therapy. 2020;37(2):692-706.

199. Grant M, J OB-E, Froud R, Underwood M, Seers K. The work of return to work. Challenges of returning to work when you have chronic pain: A meta-ethnography. BMJ Open. 2019;9(6).

200. Greenfield K, Holley S, Schoth DE, Harrop E, Howard RF, Bayliss J, et al. A mixed-methods systematic review and meta-analysis of barriers and facilitators to paediatric symptom management at end of life. Palliat Med. 2020;34(6):689-707.

201. Greenwood L, Kelly C. A systematic literature review to explore how staff in schools describe how a sense of belonging is created for their pupils. Emotional and Behavioural Difficulties. 2019;24(1):3-19.

202. Greenwood N, Mackenzie A, Cloud GC, Wilson N. Informal primary carers of stroke survivors living at home-challenges, satisfactions and coping: a systematic review of qualitative studies. Disabil Rehabil. 2009;31(5):337-51.

203. Greenwood N, Menzies-Gow E, Nilsson D, Aubrey D, Emery CL, Richardson A. Experiences of older people dying in nursing homes: a narrative systematic review of qualitative studies. BMJ Open. 2018;8(6):e021285.

204. Gregersen TA, Birkelund R, Wolderslund M, Netsey-Afedo ML, Steffensen KD, Ammentorp J. What matters in clinical trial decision-making: a systematic review of interviews exploring cancer patients' experiences. Scand J Caring Sci. 2019;33(2):266-78.

205. Gregory A, Mackintosh S, Kumar S, Grech C. Experiences of health care for older people who need support to live at home: A systematic review of the qualitative literature. Geriatr Nurs. 2017;38(4):315-24.

206. Grime J, Blenkinsopp A, Raynor DK, Pollock K, Knapp P. The role and value of written information for patients about individual medicines: a systematic review. Health Expect. 2007;10(3):286-98.

207. Gronholm PC, Nye E, Michelson D. Stigma related to targeted school-based mental health interventions: A systematic review of qualitative evidence. J Affect Disord. 2018;240:17-26.

208. Grose DN, O'Brien CL, Castle DJ. Type 1 diabetes and an insulin pump: an iterative review of qualitative literature. Practical Diabetes. 2017;34(8):281-7.

209. Gudde CB, Olsø TM, Whittington R, Vatne S. Service users' experiences and views of aggressive situations in mental health care: a systematic review and thematic synthesis of qualitative studies. J Multidiscip Healthc. 2015;8:449-62.

210. Guise A, Seguin M, Mburu G, McLean S, Grenfell P, Islam Z, et al. Integrated opioid substitution therapy and HIV care: a qualitative systematic review and synthesis of client and provider experiences. AIDS Care. 2017;29(9):1119-28.

211. Gulliver A, Griffiths KM, Christensen H. Perceived barriers and facilitators to mental health help-seeking in young people: a systematic review. BMC Psychiatry. 2010;10:113.

212. Gunawan J, Aungsuroch Y, Fisher ML. Factors contributing to managerial competence of first-line nurse managers: A systematic review. Int J Nurs Pract. 2018;24(1).

213. Gwernan-Jones R, Abbott R, Lourida I, Rogers M, Green C, Ball S, et al. The experiences of hospital staff who provide care for people living with dementia: A systematic review and synthesis of qualitative studies. Int J Older People Nurs. 2020:e12325.

214. Gwernan-Jones R, Moore DA, Cooper P, Russell AE, Richardson M, Rogers M, et al. A systematic review and synthesis of qualitative research: the influence of school context on symptoms of attention deficit hyperactivity disorder. Emotional and Behavioural Difficulties. 2016;21(1):83-100.

215. Gysels M, Pell C, Straus L, Pool R. End of life care in sub-Saharan Africa: a systematic review of the qualitative literature. BMC Palliat Care. 2011;10:6.

216. Haag H, Liang T, Avina-Zubieta JA, De Vera MA. How do patients with systemic autoimmune rheumatic disease perceive the use of their medications: a systematic review and thematic synthesis of qualitative research. BMC Rheumatol. 2018;2:9.

217. Haddaway NR, McConville J, Piniewski M. How is the term 'ecotechnology' used in the research literature? A systematic review with thematic synthesis. Ecohydrol Hydrobiol. 2018;18(3):247-61.

218. Hadfield H, Wittkowski A. Women's Experiences of Seeking and Receiving Psychological and Psychosocial Interventions for Postpartum Depression: A Systematic Review and Thematic Synthesis of the Qualitative Literature. J Midwifery Womens Health. 2017;62(6):723-36.

219. Hadgraft NT, Brakenridge CL, Dunstan DW, Owen N, Healy GN, Lawler SP. Perceptions of the acceptability and feasibility of reducing occupational sitting: Review and thematic synthesis 11 Medical and Health Sciences 1117 Public Health and Health Services. International Journal of Behavioral Nutrition and Physical Activity. 2018;15(1).

220. Haesler E, Bauer M, Nay R. Constructive staff-family relationships in the care of older adults in the institutional setting: A systematic review. JBI Libr Syst Rev. 2004;2(10 Suppl):1-76.

221. Hammer CC, Brainard J, Hunter PR. Risk factors and risk factor cascades for communicable disease outbreaks in complex humanitarian emergencies: a qualitative systematic review. BMJ Glob Health. 2018;3(4):e000647.

222. Han CS, Ogrodniczuk JS, Oliffe JL. Qualitative research on suicide in East Asia: A scoping review. Journal of Mental Health. 2013;22(4):372-83.

223. Handberg C, Nielsen CV, Lomborg K. Men's reflections on participating in cancer rehabilitation: a systematic review of qualitative studies 2000-2013. Eur J Cancer Care (Engl). 2014;23(2):159-72.

224. Hanghøj S, Boisen KA. Self-reported barriers to medication adherence among chronically ill adolescents: a systematic review. J Adolesc Health. 2014;54(2):121-38.

225. Hann KEJ, Freeman M, Fraser L, Waller J, Sanderson SC, Rahman B, et al. Awareness, knowledge, perceptions, and attitudes towards genetic testing for cancer risk among ethnic minority groups: a systematic review. BMC Public Health. 2017;17(1):503.

226. Harcourt S, Jasperse M, Green VA. "We were Sad and We were Angry": A Systematic Review of Parents' Perspectives on Bullying. Child Youth Care Forum. 2014;43(3):373-91.

227. Harden A, Brunton G, Fletcher A, Oakley A. Teenage pregnancy and social disadvantage: systematic review integrating controlled trials and qualitative studies. Bmj. 2009;339:b4254.

228. Harden J, Black R, Chin RFM. Families' experiences of living with pediatric epilepsy: A qualitative systematic review. Epilepsy Behav. 2016;60:225-37.

229. Harper C, Maher J, Grunseit A, Seimon RV, Sainsbury A. Experiences of using very low energy diets for weight loss by people with overweight or obesity: a review of qualitative research. Obes Rev. 2018;19(10):1412-23.

230. Harris BM, Harris ML, Rae K, Chojenta C. Barriers and facilitators to smoking cessation within pregnant Aboriginal and/or Torres Strait Islander women: An integrative review. Midwifery. 2019;73:49-61.

231. Harrison HL, Daker-White G. Beliefs and challenges held by medical staff about providing emergency care to migrants: an international systematic review and translation of findings to the UK context. BMJ Open. 2019;9(7):e028748.

232. Harrop E, Morgan F, Longo M, Semedo L, Fitzgibbon J, Pickett S, et al. The impacts and effectiveness of support for people bereaved through advanced illness: A systematic review and thematic synthesis. Palliat Med. 2020;34(7):871-88.

233. Harte E, MacLure C, Martin A, Saunders CL, Meads C, Walter FM, et al. Reasons why people do not attend NHS Health Checks: a systematic review and qualitative synthesis. Br J Gen Pract. 2018;68(666):e28-e35.

234. Hartmann-Boyce J, Boylan AM, Jebb SA, Aveyard P. Experiences of Self-Monitoring in Self-Directed Weight Loss and Weight Loss Maintenance: Systematic Review of Qualitative Studies. Qual Health Res. 2019;29(1):124-34.

235. Hartmann-Boyce J, Nourse R, Boylan AM, Jebb SA, Aveyard P. Experiences of Reframing during Self-Directed Weight Loss and Weight Loss Maintenance: Systematic Review of Qualitative Studies. Appl Psychol Health Well Being. 2018;10(2):309-29.

236. Hashem MD, Nallagangula A, Nalamalapu S, Nunna K, Nausran U, Robinson KA, et al. Patient outcomes after critical illness: a systematic review of qualitative studies following hospital discharge. Crit Care. 2016;20(1):345.

237. Hazzard E, Gulliver S, Walton K, McMahon AT, Milosavljevic M, Tapsell L. The patient experience of having a feeding tube during treatment for head and neck cancer: A systematic literature review. Clin Nutr ESPEN. 2019;33:66-85.

238. Heath G, Farre A, Shaw K. Parenting a child with chronic illness as they transition into adulthood: A systematic review and thematic synthesis of parents' experiences. Patient Educ Couns. 2017;100(1):76-92.

239. Heery E, Sheehan AM, While AE, Coyne I. Experiences and Outcomes of Transition from Pediatric to Adult Health Care Services for Young People with Congenital Heart Disease: A Systematic Review. Congenit Heart Dis. 2015;10(5):413-27.

240. Hefler M, Chapman S. Disadvantaged youth and smoking in mature tobacco control contexts: a systematic review and synthesis of qualitative research. Tob Control. 2015;24(5):429-35.

241. Hegarty K, McKibbin G, Hameed M, Koziol-McLain J, Feder G, Tarzia L, et al. Health practitioners' readiness to address domestic violence and abuse: A qualitative meta-synthesis. PLoS ONE. 2020;15(6).

242. Heidari H, Mardani-Hamooleh M, Amiri M. Perceived factors to providing palliative care for patients with cancer - a qualitative systematic review. Oncol Rev. 2020;14(1):463.

243. Henderson P, Fisher NR, Ball J, Sellwood W. Mental health practitioner experiences of engaging with service users in community mental health settings: A systematic review and thematic synthesis of qualitative evidence. J Psychiatr Ment Health Nurs. 2020.

244. Henderson S, Cain M, Istvandity L, Lakhani A. The role of music participation in positive health and wellbeing outcomes for migrant populations: A systematic review. Psychol Music. 2017;45(4):459-78.

245. Henderson ZB, Fox JRE, Trayner P, Wittkowski A. Emotional development in eating disorders: A qualitative metasynthesis. Clin Psychol Psychother. 2019;26(4):440-57.

246. Hengelaar AH, van Hartingsveldt M, Wittenberg Y, van Etten-Jamaludin F, Kwekkeboom R, Satink T. Exploring the collaboration between formal and informal care from the professional perspective-A thematic synthesis. Health and Social Care in the Community. 2017.

247. Hennegan J, Shannon AK, Rubli J, Schwab KJ, Melendez-Torres GJ. Women's and girls' experiences of menstruation in low- and middle-income countries: A systematic review and qualitative metasynthesis. PLoS Med. 2019;16(5):e1002803.

248. Hennink MM, Kaiser BN, Sekar S, Griswold EP, Ali MK. How are qualitative methods used in diabetes research? A 30-year systematic review. Glob Public Health. 2017;12(2):200-19.

249. Henry C, Ekeroma A, Filoche S. Barriers to seeking consultation for abnormal uterine bleeding: systematic review of qualitative research. BMC Womens Health. 2020;20(1):123.

250. Hesamzadeh A, Dalvandi A, Bagher Maddah S, Fallahi Khoshknab M, Ahmadi F. Family adaptation to stroke: A metasynthesis of qualitative research based on double ABCX model. Asian Nursing Research. 2015;9(3):177-84.

251. Hestevik CH, Molin M, Debesay J, Bergland A, Bye A. Older persons' experiences of adapting to daily life at home after hospital discharge: a qualitative metasummary. BMC Health Serv Res. 2019;19(1):224.

252. Hiatt JS, Brown TE, Banks M, Lewis CA, Bauer J. Patient and carer experience of nutrition care throughout treatment for head and neck cancer: a systematic qualitative review and thematic synthesis. Support Care Cancer. 2020.

253. Hoang H, Le Q, Ogden K. Women's maternity care needs and related service models in rural areas: A comprehensive systematic review of qualitative evidence. Women Birth. 2014;27(4):233-41.

254. Hoare T, Vidgen A, Roberts N. In their own words: a synthesis of the qualitative research on the experiences of adults seeking asylum. A systematic review of qualitative findings in forced migration. Med Confl Surviv. 2017;33(4):273-98.

255. Hodgkinson EL, Smith DM, Wittkowski A. Women's experiences of their pregnancy and postpartum body image: a systematic review and meta-synthesis. BMC Pregnancy Childbirth. 2014;14:330.

256. Hoga L, Rodolpho J, Gonçalves B, Quirino B. Women's experience of menopause: a systematic review of qualitative evidence. JBI Database System Rev Implement Rep. 2015;13(8):250-337.

257. Hoga LAK, Gouveia LMR, Higashi AB, Zamo-Roth FS. The experience and role of a companion during normal labor and childbirth: A systematic review of qualitative evidence. JBI Database of Systematic Reviews and Implementation Reports. 2013;11(12):121-56.

258. Hokka M, Martins Pereira S, Polkki T, Kyngas H, Hernandez-Marrero P. Nursing competencies across different levels of palliative care provision: A systematic integrative review with thematic synthesis. Palliative Medicine. 2020;34(7):851-70.

259. Hollingdrake O, Lui CW, Mutch A, Dean J, Howard C, Fitzgerald L. Factors affecting the decision to initiate antiretroviral therapy in the era of treatment-as-prevention: synthesis of evidence from qualitative research in high-income settings. AIDS Care - Psychological and Socio-Medical Aspects of AIDS/HIV. 2019;31(4):397-402.

260. Holopainen A, Hakulinen T. New parents' experiences of postpartum depression: a systematic review of qualitative evidence. JBI Database System Rev Implement Rep. 2019;17(9):1731-69.

261. Holtslander L, Baxter S, Mills K, Bocking S, Dadgostari T, Duggleby W, et al. Honoring the voices of bereaved caregivers: A Metasummary of qualitative research. BMC Palliative Care. 2017;16(1).

262. Hooper EK, Collins T. An occupational perspective of the lived experience of familial dementia caregivers: A thematic review of qualitative literature. Dementia (London). 2019;18(1):323-46.

263. Hordern A. Intimacy and sexuality after cancer: A critical review of the literature. Cancer Nursing. 2008;31(2):E9-E17.

264. Horne M, Tierney S. What are the barriers and facilitators to exercise and physical activity uptake and adherence among South Asian older adults: a systematic review of qualitative studies. Prev Med. 2012;55(4):276-84.

265. Hossain M, Crossland J, Stores R, Dewey A, Hakak Y. Awareness and understanding of dementia in South Asians: A synthesis of qualitative evidence. Dementia (London, England). 2020;19(5):1441-73.

266. Houghton C, Meskell P, Delaney H, Smalle M, Glenton C, Booth A, et al. Barriers and facilitators to healthcare workers' adherence with infection prevention and control (IPC) guidelines for respiratory infectious diseases: a rapid qualitative evidence synthesis. Cochrane Database Syst Rev. 2020;4(4):Cd013582.

267. Howard AF, Balneaves LG, Bottorff JL. Women's decision making about risk-reducing strategies in the context of hereditary breast and ovarian cancer: a systematic review. J Genet Couns. 2009;18(6):578-97.

268. Howard J, Fisher Z, Kemp AH, Lindsay S, Tasker LH, Tree JJ. Exploring the barriers to using assistive technology for individuals with chronic conditions: a meta-synthesis review. Disability and rehabilitation. 2020;Assistive technology.:1-19.

269. Howe N, Giles E, Newbury-Birch D, McColl E. Systematic review of participants' attitudes towards data sharing: a thematic synthesis. J Health Serv Res Policy. 2018;23(2):123-33.

270. Howell BM, Peterson JR. "With Age Comes Wisdom:" a Qualitative Review of Elder Perspectives on Healthy Aging in the Circumpolar North. J Cross Cult Gerontol. 2020;35(2):113-31.

271. Huang X, O'Connor M, Lee S. School-aged and adolescent children's experience when a parent has non-terminal cancer: a systematic review and meta-synthesis of qualitative studies. Psychooncology. 2014;23(5):493-506.

272. Huang Y, Martinez-Alvarez M, Shallcross D, Pi L, Tian F, Pan J, et al. Barriers to accessing maternal healthcare among ethnic minority women in Western China: a qualitative evidence synthesis. Health Policy Plan. 2019;34(5):384-400.

273. Hughes S, Lewis S, Willis K, Rogers A, Wyke S, Smith L. The experience of facilitators and participants of long term condition self-management group programmes: A qualitative synthesis. Patient Education and Counseling. 2017;100(12):2244-54.

274. Hunter H, Lovegrove C, Haas B, Freeman J, Gunn H. Experiences of people with Parkinson's disease and their views on physical activity interventions: a qualitative systematic review. JBI Database System Rev Implement Rep. 2019;17(4):548-613.

275. Huntingdon B, de Wit J, Duracinsky M, Juraskova I. The current state of qualitative research on sexual functioning with HIV in developed nations: a thematic synthesis. Sexual and Relationship Therapy. 2018.

276. Huntley AL, Potter L, Williamson E, Malpass A, Szilassy E, Feder G. Help-seeking by male victims of domestic violence and abuse (DVA): a systematic review and qualitative evidence synthesis. BMJ Open. 2019;9(6):e021960.

277. Husk K, Lovell R, Cooper C, Stahl-Timmins W, Garside R. Participation in environmental enhancement and conservation activities for health and well-being in adults: a review of quantitative and qualitative evidence. Cochrane Database Syst Rev. 2016;2016(5):Cd010351.

278. Igai Y. End-of-life trajectory of coping and self-care of patients with idiopathic pulmonary fibrosis: A meta-synthesis using meta-ethnography. Japan Journal of Nursing Science. 2018.

279. Jackson LJ, Roberts TE. Conceptualising quality of life outcomes for women participating in testing for sexually transmitted infections: A systematic review and meta-synthesis of qualitative research. Soc Sci Med. 2015;143:162-70.

280. Jacobson J, Gomersall JS, Campbell J, Hughes M. Carers' experiences when the person for whom they have been caring enters a residential aged care facility permanently: a systematic review. JBI Database System Rev Implement Rep. 2015;13(7):241-317.

281. Jager M, de Zeeuw J, Tullius J, Papa R, Giammarchi C, Whittal A, et al. Patient Perspectives to Inform a Health Literacy Educational Program: A Systematic Review and Thematic Synthesis of Qualitative Studies. Int J Environ Res Public Health. 2019;16(21).

282. Jakimowicz M, Williams D, Stankiewicz G. A systematic review of experiences of advanced practice nursing in general practice. BMC Nurs. 2017;16:6.

283. Jamal F, Fletcher A, Harden A, Wells H, Thomas J, Bonell C. The school environment and student health: a systematic review and meta-ethnography of qualitative research. BMC Public Health. 2013;13:798.

284. Jennekens N, de Casterlé BD, Dobbels F. A systematic review of care needs of people with traumatic brain injury (TBI) on a cognitive, emotional and behavioural level. J Clin Nurs. 2010;19(9-10):1198-206.

285. Johns G, Taylor B, John A, Tan J. Current eating disorder healthcare services - the perspectives and experiences of individuals with eating disorders, their families and health professionals: systematic review and thematic synthesis. BJPsych Open. 2019;5(4):e59.

286. Johnson GA, Vindrola-Padros C. Rapid qualitative research methods during complex health emergencies: A systematic review of the literature. Soc Sci Med. 2017;189:63-75.

287. Johnson M, Baxter S, Blank L, Cantrell A, Brumfitt S, Enderby P, et al. The state of the art in non-pharmacological interventions for developmental stuttering. Part 2: qualitative evidence synthesis of views and experiences. Int J Lang Commun Disord. 2016;51(1):3-17.

288. Johnson SF, Woodgate RL. Qualitative research in teen experiences living with food-induced anaphylaxis: A meta-aggregation. J Adv Nurs. 2017;73(11):2534-46.

289. Johnston L, Young J, Campbell K. The implementation and impact of Holistic Needs Assessments for people affected by cancer: A systematic review and thematic synthesis of the literature. Eur J Cancer Care (Engl). 2019;28(3):e13087.

290. Johnstone MJ, Turale S. Nurses' experiences of ethical preparedness for public health emergencies and healthcare disasters: a systematic review of qualitative evidence. Nurs Health Sci. 2014;16(1):67-77.

291. Jones CE, Maben J, Jack RH, Davies EA, Forbes LJ, Lucas G, et al. A systematic review of barriers to early presentation and diagnosis with breast cancer among black women. BMJ Open. 2014;4(2):e004076.

292. Jones CH, Howick J, Roberts NW, Price CP, Heneghan C, Plüddemann A, et al. Primary care clinicians' attitudes towards point-of-care blood testing: a systematic review of qualitative studies. BMC Fam Pract. 2013;14:117.

293. Jones FA, Knights DP, Sinclair VF, Baraitser P. Do health partnerships with organisations in lower income countries benefit the UK partner? A review of the literature. Global Health. 2013;9:38.

294. Jones HM, Al-Khudairy L, Melendez-Torres GJ, Oyebode O. Viewpoints of adolescents with overweight and obesity attending lifestyle obesity treatment interventions: a qualitative systematic review. Obes Rev. 2019;20(1):156-69.

295. Jones L, Atkinson A, Bates G, McCoy E, Porcellato L, Beynon C, et al. Views and experiences of hepatitis C testing and diagnosis among people who inject drugs: systematic review of qualitative research. Int J Drug Policy. 2014;25(2):204-11.

296. Jones ML. Role development and effective practice in specialist and advanced practice roles in acute hospital settings: systematic review and meta-synthesis. J Adv Nurs. 2005;49(2):191-209.

297. Joo JY, Huber DL. Barriers in Case Managers' Roles: A Qualitative Systematic Review. West J Nurs Res. 2018;40(10):1522-42.

298. Joo JY, Liu MF. Experiences of case management with chronic illnesses: a qualitative systematic review. Int Nurs Rev. 2018;65(1):102-13.

299. Joo JY, Liu MF. Experience of Culturally-Tailored Diabetes Interventions for Ethnic Minorities: A Qualitative Systematic Review. Clin Nurs Res. 2019:1054773819885952.

300. Joo JY, Liu MF. Nurses' Barriers to Care of Ethnic Minorities: A Qualitative Systematic Review. West J Nurs Res. 2020;42(9):760-71.

301. Jordan G, MacDonald K, Pope MA, Schorr E, Malla AK, Iyer SN. Positive Changes Experienced After a First Episode of Psychosis: A Systematic Review. Psychiatr Serv. 2018;69(1):84-99.

302. Jordan J, Rose L, Dainty KN, Noyes J, Blackwood B. Factors that impact on the use of mechanical ventilation weaning protocols in critically ill adults and children: a qualitative evidence-synthesis. Cochrane Database Syst Rev. 2016;10(10):Cd011812.

303. Jørgensen CR, Thomsen TG, Ross L, Dietz SM, Therkildsen S, Groenvold M, et al. What Facilitates "Patient Empowerment" in Cancer Patients During Follow-Up: A Qualitative Systematic Review of the Literature. Qual Health Res. 2018;28(2):292-304.

304. Joseph-Williams N, Elwyn G, Edwards A. Knowledge is not power for patients: a systematic review and thematic synthesis of patient-reported barriers and facilitators to shared decision making. Patient Educ Couns. 2014;94(3):291-309.

305. Ka'apu K, Burnette CE. A Culturally Informed Systematic Review of Mental Health Disparities Among Adult Indigenous Men and Women of the USA: What is known? Br J Soc Work. 2019;49(4):880-98.

306. Kaldal MH, Kristiansen J, Uhrenfeldt L. Nursing students experienced personal inadequacy, vulnerability and transformation during their patient care encounter: A qualitative meta-synthesis. Nurse Educ Today. 2018;64:99-107.

307. Kamga KK, De Vries J, Nguefack S, Munung SN, Wonkam A. Lived Experiences of Fragile X Syndrome Caregivers: A Scoping Review of Qualitative Studies. Frontiers in Neurology. 2020;11 (no pagination).

308. Kaminskiy E, Senner S, Hamann J. Attitudes towards shared decision making in mental health: A qualitative synthesis. Mental Health Review Journal. 2017;22(3):233-56.

309. Kanavaki AM, Rushton A, Efstathiou N, Alrushud A, Klocke R, Abhishek A, et al. Barriers and facilitators of physical activity in knee and hip osteoarthritis: a systematic review of qualitative evidence. BMJ Open. 2017;7(12):e017042.

310. Kandasamy S, Khalid AF, Majid U, Vanstone M. Prostate Cancer Patient Perspectives on the Use of Information in Treatment Decision-Making: A Systematic Review and Qualitative Meta-synthesis. Ont Health Technol Assess Ser. 2017;17(7):1-32.

311. Kane GA, Wood VA, Barlow J. Parenting programmes: a systematic review and synthesis of qualitative research. Child Care Health Dev. 2007;33(6):784-93.

312. Kapadia D, Brooks HL, Nazroo J, Tranmer M. Pakistani women's use of mental health services and the role of social networks: a systematic review of quantitative and qualitative research. Health Soc Care Community. 2017;25(4):1304-17.

313. Karageorge A, Rhodes P, Gray R, Papadopoulos R. Refugee and staff experiences of psychotherapeutic services: a qualitative systematic review. Intervention. 2017;15(1):51-69.

314. Karam M, Brault I, Van Durme T, Macq J. Comparing interprofessional and interorganizational collaboration in healthcare: A systematic review of the qualitative research. Int J Nurs Stud. 2018;79:70-83.

315. Karimi-Shahanjarini A, Shakibazadeh E, Rashidian A, Hajimiri K, Glenton C, Noyes J, et al. Barriers and facilitators to the implementation of doctor-nurse substitution strategies in primary care: a qualitative evidence synthesis. Cochrane Database Syst Rev. 2019;4(4):Cd010412.

316. Karlsen C, Ludvigsen MS, Moe CE, Haraldstad K, Thygesen E. Experiences of community-dwelling older adults with the use of telecare in home care services: a qualitative systematic review. JBI Database System Rev Implement Rep. 2017;15(12):2913-80.

317. Kars MC, van Thiel GJ, van der Graaf R, Moors M, de Graeff A, van Delden JJ. A systematic review of reasons for gatekeeping in palliative care research. Palliat Med. 2016;30(6):533-48.

318. Ke LS, Huang X, Hu WY, O'Connor M, Lee S. Experiences and perspectives of older people regarding advance care planning: A meta-synthesis of qualitative studies. Palliat Med. 2017;31(5):394-405.

319. Ke LS, Huang X, O'Connor M, Lee S. Nurses' views regarding implementing advance care planning for older people: a systematic review and synthesis of qualitative studies. J Clin Nurs. 2015;24(15-16):2057-73.

320. Keeping-Burke L, McCloskey R, Donovan C, Yetman L, Goudreau A. Nursing students' experiences with clinical placement in residential aged care facilities: a systematic review of qualitative evidence. JBI Evid Synth. 2020;18(5):986-1018.

321. Kennelly JD, Daveson BA, Baker FA. Effects of professional music therapy supervision on clinical outcomes and therapist competency: a systematic review involving narrative synthesis. Nord J Music Ther. 2016;25(2):185-208.

322. Kenny M, Darcy-Bewick S, Martin A, Eustace-Cook J, Hilliard C, Clinton F, et al. You are at rock bottom: A qualitative systematic review of the needs of bereaved parents as they journey through the death of their child to cancer. J Psychosoc Oncol. 2020:1-21.

323. Kerr C, Nixon A, Angalakuditi M. The impact of epilepsy on children and adult patients' lives: Development of a conceptual model from qualitative literature. Seizure. 2011;20(10):764-74.

324. Kerr ZY, Miller KR, Galos D, Love R, Poole C. Challenges, coping strategies, and recommendations related to the HIV services field in the HAART era: a systematic literature review of qualitative studies from the United States and Canada. AIDS Patient Care STDS. 2013;27(2):85-95.

325. Keyko K, Cummings GG, Yonge O, Wong CA. Work engagement in professional nursing practice: A systematic review. Int J Nurs Stud. 2016;61:142-64.

326. Khoiriyati A, Kusnanto, Kurniawati ND. Experiences of recovery from acute coronary syndrome: A systematic review. Indian Journal of Public Health Research and Development. 2019;10(8):2813-6.

327. Kilgour E, Kosny A, McKenzie D, Collie A. Healing or harming? Healthcare provider interactions with injured workers and insurers in workers' compensation systems. J Occup Rehabil. 2015;25(1):220-39.

328. Kilgour E, Kosny A, McKenzie D, Collie A. Interactions between injured workers and insurers in workers' compensation systems: a systematic review of qualitative research literature. J Occup Rehabil. 2015;25(1):160-81.

329. Killackey T, Lovrics E, Saunders S, Isenberg SR. Palliative care transitions from acute care to community-based care: A qualitative systematic review of the experiences and perspectives of health care providers. Palliat Med. 2020:269216320947601.

330. Killingback C, Ahmed O, Williams J. 'It was all in your voice' - Tertiary student perceptions of alternative feedback modes (audio, video, podcast, and screencast): A qualitative literature review. Nurse Education Today. 2019;72:32-9.

331. Kim H, Sefcik JS, Bradway C. Characteristics of Qualitative Descriptive Studies: A Systematic Review. Res Nurs Health. 2017;40(1):23-42.

332. King AJ, Evans M, Moore TH, Paterson C, Sharp D, Persad R, et al. Prostate cancer and supportive care: a systematic review and qualitative synthesis of men's experiences and unmet needs. Eur J Cancer Care (Engl). 2015;24(5):618-34.

333. King AJL, Johnson R, Cramer H, Purdy S, Huntley AL. Community case management and unplanned hospital admissions in patients with heart failure: A systematic review and qualitative evidence synthesis. J Adv Nurs. 2018;74(7):1463-73.

334. Kingdon C, Downe S, Betran AP. Non-clinical interventions to reduce unnecessary caesarean section targeted at organisations, facilities and systems: Systematic review of qualitative studies. PLoS One. 2018;13(9):e0203274.

335. Kingdon C, Downe S, Betran AP. Interventions targeted at health professionals to reduce unnecessary caesarean sections: A qualitative evidence synthesis. BMJ Open. 2018;8(12).

336. Kılıç Onar D, Armstrong H, Graham CA. What Does Research Tell Us About Women's Experiences, Motives and Perceptions of Masturbation Within a Relationship Context?: A Systematic Review of Qualitative Studies. J Sex Marital Ther. 2020:1-34.

337. Kleij KS, Tangermann U, Amelung VE, Krauth C. Patients' preferences for primary health care - a systematic literature review of discrete choice experiments. BMC Health Serv Res. 2017;17(1):476.

338. Koch G, Wakefield BJ, Wakefield DS. Barriers and facilitators to managing multiple chronic conditions: a systematic literature review. West J Nurs Res. 2015;37(4):498-516.

339. Koerting J, Smith E, Knowles MM, Latter S, Elsey H, McCann DC, et al. Barriers to, and facilitators of, parenting programmes for childhood behaviour problems: A qualitative synthesis of studies of parents' and professionals' perceptions. European Child and Adolescent Psychiatry. 2013;22(11):653-70.

340. Koivunen M, Saranto K. Nursing professionals' experiences of the facilitators and barriers to the use of telehealth applications: a systematic review of qualitative studies. Scand J Caring Sci. 2018;32(1):24-44.

341. Kok MC, Dieleman M, Taegtmeyer M, Broerse JE, Kane SS, Ormel H, et al. Which intervention design factors influence performance of community health workers in low- and middle-income countries? A systematic review. Health Policy Plan. 2015;30(9):1207-27.

342. Kornhaber RA, de Jong AEE, McLean L. Rigorous, robust and systematic: Qualitative research and its contribution to burn care. An integrative review. Burns. 2015;41(8):1619-26.

343. Krockow EM, Colman AM, Chattoe-Brown E, Jenkins DR, Perera N, Mehtar S, et al. Balancing the risks to individual and society: a systematic review and synthesis of qualitative research on antibiotic prescribing behaviour in hospitals. J Hosp Infect. 2019;101(4):428-39.

344. Krumm S, Checchia C, Koesters M, Kilian R, Becker T. Men's Views on Depression: A Systematic Review and Metasynthesis of Qualitative Research. Psychopathology. 2017;50(2):107-24.

345. Lakeman R, FitzGerald M. How people live with or get over being suicidal: a review of qualitative studies. J Adv Nurs. 2008;64(2):114-26.

346. Lall P, Rees R, Law GCY, Dunleavy G, Cotič Ž, Car J. Influences on the Implementation of Mobile Learning for Medical and Nursing Education: Qualitative Systematic Review by the Digital Health Education Collaboration. J Med Internet Res. 2019;21(2):e12895.

347. Lam M, Lam HR, Agarwal A, Chow R, Chow S, Chow E, et al. Clinicians' views on palliative sedation for existential suffering: A systematic review and thematic synthesis of qualitative studies. Journal of Pain Management. 2017;10(1):31-40.

348. Larsen SM, Mortensen RF, Kristensen HK, Hounsgaard L. Older adults' perspectives on the process of becoming users of assistive technology: a qualitative systematic review and meta-synthesis. Disabil Rehabil Assist Technol. 2019;14(2):182-93.

349. Lashewicz BM, Shipton L, Lien K. Meta-synthesis of fathers' experiences raising children on the autism spectrum. J Intellect Disabil. 2019;23(1):117-31.

350. Laugesen B, Groenkjaer M. Parenting experiences of living with a child with attention deficit hyperactivity disorder: a systematic review of qualitative evidence. JBI Database System Rev Implement Rep. 2015;13(11):169-234.

351. Laugesen B, Lauritsen MB, Jørgensen R, Sørensen EE, Rasmussen P, Grønkjær M. Living with a child with attention deficit hyperactivity disorder: a systematic review. Int J Evid Based Healthc. 2016;14(4):150-65.

352. Lauricella M, Valdez JK, Okamoto SK, Helm S, Zaremba C. Culturally Grounded Prevention for Minority Youth Populations: A Systematic Review of the Literature. J Prim Prev. 2016;37(1):11-32.

353. Lauritzen J, Pedersen PU, Sørensen EE, Bjerrum MB. The meaningfulness of participating in support groups for informal caregivers of older adults with dementia: a systematic review. JBI Database System Rev Implement Rep. 2015;13(6):373-433.

354. Lawn S, Roberts L, Willis E, Couzner L, Mohammadi L, Goble E. The effects of emergency medical service work on the psychological, physical, and social well-being of ambulance personnel: a systematic review of qualitative research. BMC Psychiatry. 2020;20(1):348.

355. Learmonth YC, Motl RW. Physical activity and exercise training in multiple sclerosis: a review and content analysis of qualitative research identifying perceived determinants and consequences. Disabil Rehabil. 2016;38(13):1227-42.

356. Lee H, Tamminen KA, Clark AM, Slater L, Spence JC, Holt NL. A meta-study of qualitative research examining determinants of children's independent active free play. Int J Behav Nutr Phys Act. 2015;12:5.

357. Lee Mortensen G, Strand AM, Almen L. Adherence to prophylactic haemophilic treatment in young patients transitioning to adult care: A qualitative review. Haemophilia. 2018;24(6):862-72.

358. Legg H, Tickle A. UK parents' experiences of their child receiving a diagnosis of autism spectrum disorder: A systematic review of the qualitative evidence. Autism. 2019;23(8):1897-910.

359. Lemmer B, Grellier R, Steven J. Systematic review of nonrandom and qualitative research literature: Exploring and uncovering an evidence base for health visiting and decision making. Qualitative Health Research. 1999;9(3):315-28.

360. Lever Taylor B, Billings J, Morant N, Johnson S. How do women's partners view perinatal mental health services? A qualitative meta-synthesis. Clin Psychol Psychother. 2018;25(1):112-29.

361. Lewinski AA, Shapiro A, Gierisch JM, Goldstein KM, Blalock DV, Luedke MW, et al. Barriers and facilitators to implementation of epilepsy self-management programs: a systematic review using qualitative evidence synthesis methods. Syst Rev. 2020;9(1):92.

362. Lewis SA, Noyes J, Hastings RP. Systematic review of epilepsy self-management interventions integrated with a synthesis of children and young people's views and experiences. J Adv Nurs. 2015;71(3):478-97.

363. Liao L, Xiao LD, Chen H, Wu XY, Zhao Y, Hu M, et al. Nursing home staff experiences of implementing mentorship programmes: A systematic review and qualitative meta-synthesis. J Nurs Manag. 2020;28(2):188-98.

364. Liddy C, Blazkho V, Mill K. Challenges of self-management when living with multiple chronic conditions: systematic review of the qualitative literature. Can Fam Physician. 2014;60(12):1123-33.

365. Lim S, Tan A, Madden S, Hill B. Health Professionals' and Postpartum Women's Perspectives on Digital Health Interventions for Lifestyle Management in the Postpartum Period: A Systematic Review of Qualitative Studies. Front Endocrinol (Lausanne). 2019;10:767.

366. Linnarsson JR, Bubini J, Perseius KI. A meta-synthesis of qualitative research into needs and experiences of significant others to critically ill or injured patients. J Clin Nurs. 2010;19(21-22):3102-11.

367. Lippiett KA, Richardson A, Myall M, Cummings A, May CR. Patients and informal caregivers' experiences of burden of treatment in lung cancer and chronic obstructive pulmonary disease (COPD): a systematic review and synthesis of qualitative research. BMJ Open. 2019;9(2):e020515.

368. Little H, Tickle A, das Nair R. Process and impact of dialectical behaviour therapy: A systematic review of perceptions of clients with a diagnosis of borderline personality disorder. Psychol Psychother. 2018;91(3):278-301.

369. Livsey L, Lewis K. Breast cancer survivors' perceptions of participating in a supervised exercise intervention: An exploratory review of the literature. Women Health. 2018;58(9):1017-36.

370. Lloyd A, Bannigan K, Sugavanam T, Freeman J. Experiences of stroke survivors, their families and unpaid carers in goal setting within stroke rehabilitation: a systematic review of qualitative evidence. JBI Database System Rev Implement Rep. 2018;16(6):1418-53.

371. Lloyd J, Patterson T, Muers J. The positive aspects of caregiving in dementia: A critical review of the qualitative literature. Dementia. 2014;15(6):1534-61.

372. Lo TLT, Lee JLC, Ho RTH. Creative Arts-Based Therapies for Stroke Survivors: A Qualitative Systematic Review. Front Psychol. 2018;9:1646.

373. Lohan M, Cruise S, O'Halloran P, Alderdice F, Hyde A. Adolescent men's attitudes in relation to pregnancy and pregnancy outcomes: a systematic review of the literature from 1980-2009. J Adolesc Health. 2010;47(4):327-45.

374. Long L, Moore D, Robinson S, Sansom A, Aylward A, Fletcher E, et al. Understanding why primary care doctors leave direct patient care: a systematic review of qualitative research. BMJ Open. 2020;10(5):e029846.

375. Lorenc T, Marshall D, Wright K, Sutcliffe K, Sowden A. Seasonal influenza vaccination of healthcare workers: systematic review of qualitative evidence. BMC Health Serv Res. 2017;17(1):732.

376. Lorenc T, Tyner EF, Petticrew M, Duffy S, Martineau FP, Phillips G, et al. Cultures of evidence across policy sectors: systematic review of qualitative evidence. Eur J Public Health. 2014;24(6):1041-7.

377. Lou S, Hvidman L, Uldbjerg N, Neumann L, Jensen TF, Haben JG, et al. Women's experiences of postterm induction of labor: A systematic review of qualitative studies. Birth. 2019;46(3):400-10.

378. Lou S, Jensen LG, Petersen OB, Vogel I, Hvidman L, Møller A, et al. Parental response to severe or lethal prenatal diagnosis: a systematic review of qualitative studies. Prenat Diagn. 2017;37(8):731-43.

379. Lovell N, Etkind SN, Bajwah S, Maddocks M, Higginson IJ. Control and Context Are Central for People With Advanced Illness Experiencing Breathlessness: A Systematic Review and Thematic Synthesis. J Pain Symptom Manage. 2019;57(1):140-55.e2.

380. Low LF, Swaffer K, McGrath M, Brodaty H. Do people with early stage dementia experience Prescribed Disengagement®? A systematic review of qualitative studies. Int Psychogeriatr. 2018;30(6):807-31.

381. Lu H, Xie J, Gerido LH, Cheng Y, Chen Y, Sun L. Information Needs of Breast Cancer Patients: Theory-Generating Meta-Synthesis. J Med Internet Res. 2020;22(7):e17907.

382. Lucas G, Olander EK, Ayers S, Salmon D. No straight lines - young women's perceptions of their mental health and wellbeing during and after pregnancy: a systematic review and meta-ethnography. BMC Womens Health. 2019;19(1):152.

383. Lucas PJ, Baird J, Arai L, Law C, Roberts HM. Worked examples of alternative methods for the synthesis of qualitative and quantitative research in systematic reviews. BMC Med Res Methodol. 2007;7:4.

384. Lucherini M, Hill S, Smith K. Inequalities, harm reduction and non-combustible nicotine products: a meta-ethnography of qualitative evidence. BMC public health. 2020;20(1):943.

385. Luckett T, Davidson PM, Green A, Boyle F, Stubbs J, Lovell M. Assessment and management of adult cancer pain: a systematic review and synthesis of recent qualitative studies aimed at developing insights for managing barriers and optimizing facilitators within a comprehensive framework of patient care. J Pain Symptom Manage. 2013;46(2):229-53.

386. Lyberg A, Dahl B, Haruna M, Takegata M, Severinsson E. Links between patient safety and fear of childbirth-A meta-study of qualitative research. Nursing Open. 2018.

387. Ma PHX, Chan ZCY, Loke AY. Conflicting identities between sex workers and motherhood: A systematic review. Women Health. 2019;59(5):534-57.

388. Ma S, Yu H, Liang N, Zhu S, Li X, Robinson N, et al. Components of complex interventions for healthcare: A narrative synthesis of qualitative studies. Journal of Traditional Chinese Medical Sciences. 2020;7(2):181-8.

389. Macdonald D, Snelgrove-Clarke E, Campbell-Yeo M, Aston M, Helwig M, Baker KA. The experiences of midwives and nurses collaborating to provide birthing care: a systematic review. JBI Database System Rev Implement Rep. 2015;13(11):74-127.

390. MacDonald S, Sampson C, Turley R, Biddle L, Ring N, Begley R, et al. Patients' Experiences of Emergency Hospital Care Following Self-Harm: Systematic Review and Thematic Synthesis of Qualitative Research. Qual Health Res. 2020;30(3):471-85.

391. MacEachen E, Kosny A, Scott-Dixon K, Facey M, Chambers L, Breslin C, et al. Workplace health understandings and processes in small businesses: a systematic review of the qualitative literature. J Occup Rehabil. 2010;20(2):180-98.

392. MacKinnon K, Marcellus L, Rivers J, Gordon C, Ryan M, Butcher D. Student and educator experiences of maternal-child simulation-based learning: a systematic review of qualitative evidence. JBI Database System Rev Implement Rep. 2017;15(11):2666-706.

393. MacMillan M, Tarrant M, Abraham C, Morris C. The association between children's contact with people with disabilities and their attitudes towards disability: a systematic review. Dev Med Child Neurol. 2014;56(6):529-46.

394. Macutkiewicz J, MacBeth A. Intended Adolescent Pregnancy: A Systematic Review of Qualitative Studies. Adolescent Research Review. 2017;2(2):113-29.

395. Maffoni M, Giardini A, Pierobon A, Ferrazzoli D, Frazzitta G. Stigma Experienced by Parkinson's Disease Patients: A Descriptive Review of Qualitative Studies. Parkinson's Disease. 2017;2017 (no pagination).

396. Majeed-Ariss R, Jackson C, Knapp P, Cheater FM. A systematic review of research into black and ethnic minority patients' views on self-management of type 2 diabetes. Health Expect. 2015;18(5):625-42.

397. Majid U, Kandasamy S, Farrah K, Vanstone M. Women's preferences and experiences of cervical cancer screening in rural and remote areas: a systematic review and qualitative meta-synthesis. Rural Remote Health. 2019;19(4):5190.

398. Makanjuola T, Taddese HB, Booth A. Factors associated with adherence to treatment with isoniazid for the prevention of tuberculosis amongst people living with HIV/AIDS: a systematic review of qualitative data. PLoS One. 2014;9(2):e87166.

399. Malli MA, Sams L, Forrester-Jones R, Murphy G, Henwood M. Austerity and the lives of people with learning disabilities. A thematic synthesis of current literature. Disability & Society. 2018;33(9):1412-35.

400. Manchaiah V, Bellon-Harn ML, Dockens AL, Azios JH, Harn WE. Communication between audiologist, patient, and patient's family members during initial audiology consultation and rehabilitation planning sessions: A descriptive review. Journal of the American Academy of Audiology. 2019;30(9):810-9.

401. Maragh-Bass AC, Appelson JR, Changoor NR, Davis WA, Haider AH, Morris MA. Prioritizing qualitative research in surgery: A synthesis and analysis of publication trends. Surgery (United States). 2016;160(6):1447-55.

402. Maribo T, Jensen CM, Madsen LS, Handberg C. Experiences with and perspectives on goal setting in spinal cord injury rehabilitation: a systematic review of qualitative studies. Spinal Cord. 2020.

403. Marshall IJ, Wolfe CD, McKevitt C. Lay perspectives on hypertension and drug adherence: systematic review of qualitative research. Bmj. 2012;345:e3953.

404. Marshall T. The concept of reflection: a systematic review and thematic synthesis across professional contexts. Reflect Pract. 2019;20(3):396-415.

405. Marston C, King E. Factors that shape young people's sexual behaviour: a systematic review. Lancet. 2006;368(9547):1581-6.

406. Mas Dalmau G, Sant Arderiu E, Enfedaque Montes MB, Solà I, Pequeño Saco S, Alonso Coello P. Patients' and physicians' perceptions and attitudes about oral anticoagulation and atrial fibrillation: a qualitative systematic review. BMC Fam Pract. 2017;18(1):3.

407. Mather MW, Hamilton D, Robalino S, Rousseau N. Going where other methods cannot: A systematic mapping review of 25 years of qualitative research in Otolaryngology. Clin Otolaryngol. 2018;43(6):1443-53.

408. Mathieson A, Grande G, Luker K. Strategies, facilitators and barriers to implementation of evidence-based practice in community nursing: a systematic mixed-studies review and qualitative synthesis. Prim Health Care Res Dev. 2019;20:e6.

409. Mathioudakis AG, Salakari M, Pylkkanen L, Saz-Parkinson Z, Bramesfeld A, Deandrea S, et al. Systematic review on women's values and preferences concerning breast cancer screening and diagnostic services. Psychooncology. 2019;28(5):939-47.

410. Mayston R, Frissa S, Tekola B, Hanlon C, Prince M, Fekadu A. Explanatory models of depression in sub-Saharan Africa: Synthesis of qualitative evidence. Social Science and Medicine. 2020;246 (no pagination).

411. Mbau R, Gilson L. Influence of organisational culture on the implementation of health sector reforms in low- and middle-income countries: a qualitative interpretive review. Global Health Action. 2018;11(1).

412. McCaffrey N, Bradley S, Ratcliffe J, Currow DC. What Aspects of Quality of Life Are Important From Palliative Care Patients' Perspectives? A Systematic Review of Qualitative Research. J Pain Symptom Manage. 2016;52(2):318-28.e5.

413. McCann E, Donohue G, de Jager J, Nugter A, Stewart J, Eustace-Cook J. Sexuality and intimacy among people with serious mental illness: a qualitative systematic review. JBI Database System Rev Implement Rep. 2019;17(1):74-125.

414. McCashin D, Coyle D, O'Reilly G. Qualitative Synthesis of Young People's Experiences With Technology-Assisted Cognitive Behavioral Therapy: Systematic Review. J Med Internet Res. 2019;21(11):e13540.

415. McCloskey RM, Furlong KE, Hansen L. Patient, family and nurse experiences with patient presence during handovers in acute care hospital settings: a systematic review of qualitative evidence. JBI Database System Rev Implement Rep. 2019;17(5):754-92.

416. McCrae N, Purssell E. Is it really theoretical? A review of sampling in grounded theory studies in nursing journals. J Adv Nurs. 2016;72(10):2284-93.

417. McEwan MJ, Espie CA, Metcalfe J. A systematic review of the contribution of qualitative research to the study of quality of life in children and adolescents with epilepsy. Seizure. 2004;13(1):3-14.

418. McGowan LJ, Devereux-Fitzgerald A, Powell R, French DP. How acceptable do older adults find the concept of being physically active? A systematic review and meta-synthesis. Int Rev Sport Exerc Psychol. 2018;11(1):1-24.

419. McGowan LJ, Powell R, French DP. How can use of the Theoretical Domains Framework be optimized in qualitative research? A rapid systematic review. Br J Health Psychol. 2020;25(3):677-94.

420. McInerney P, Slemming W, Basu J, Stewart A. Choosing to have an illegal abortion in Southern Africa: A comprehensive systematic review of the qualitative text and opinion-based evidence. JBI Database of Systematic Reviews and Implementation Reports. 2013;11(12):216-54.

421. McKevitt C, Redfern J, Mold F, Wolfe C. Qualitative studies of stroke: a systematic review. Stroke. 2004;35(6):1499-505.

422. McKnight P, Goodwin L, Kenyon S. A systematic review of asylum-seeking women's views and experiences of UK maternity care. Midwifery. 2019;77:16-23.

423. McPherson S, Wicks C, Tercelli I. Patient experiences of psychological therapy for depression: A qualitative metasynthesis. BMC Psychiatry. 2020;20(1).

424. McTavish JR, Kimber M, Devries K, Colombini M, MacGregor JCD, Wathen N, et al. Children's and caregivers' perspectives about mandatory reporting of child maltreatment: A meta-synthesis of qualitative studies. BMJ Open. 2019;9(4).

425. McWhirter JE, Hoffman-Goetz L. Visual images for skin cancer prevention: a systematic review of qualitative studies. J Cancer Educ. 2012;27(2):202-16.

426. Megnin-Viggars O, Symington I, Howard LM, Pilling S. Experience of care for mental health problems in the antenatal or postnatal period for women in the UK: a systematic review and meta-synthesis of qualitative research. Arch Womens Ment Health. 2015;18(6):745-59.

427. Meißner A, Schnepp W. Staff experiences within the implementation of computer-based nursing records in residential aged care facilities: a systematic review and synthesis of qualitative research. BMC Med Inform Decis Mak. 2014;14:54.

428. Melendez-Torres GJ, Grant S, Bonell C. A systematic review and critical appraisal of qualitative metasynthetic practice in public health to develop a taxonomy of operations of reciprocal translation. Res Synth Methods. 2015;6(4):357-71.

429. Melvin K, Meyer C, Scarinci N. What does "engagement" mean in early speech pathology intervention? A qualitative systematised review. Disability and Rehabilitation. 2019.

430. Mendes A, Hoga L, Gonçalves B, Silva P, Pereira P. Adult women's experiences of urinary incontinence: a systematic review of qualitative evidence. JBI Database System Rev Implement Rep. 2017;15(5):1350-408.

431. Mercer C, Byrth J, Jordan Z. The experiences of Aboriginal health workers and non-Aboriginal health professionals working collaboratively in the delivery of health care to Aboriginal Australians: A systematic review. JBI Database of Systematic Reviews and Implementation Reports. 2014;12(3):274-418.

432. Methley AM, Chew-Graham C, Campbell S, Cheraghi-Sohi S. Experiences of UK health-care services for people with Multiple Sclerosis: a systematic narrative review. Health Expect. 2015;18(6):1844-55.

433. Middleton TRF, Petersen B, Schinke RJ, Kao SF, Giffin C. Community sport and physical activity programs as sites of integration: A meta-synthesis of qualitative research conducted with forced migrants. Psychology of Sport and Exercise. 2020;51 (no pagination).

434. Mijovic H, McKnight J, English M. What does the literature tell us about health workers' experiences of task-shifting projects in sub-Saharan Africa? A systematic, qualitative review. J Clin Nurs. 2016;25(15-16):2083-100.

435. Miles C, Arden-Close E, Thomas M, Bruton A, Yardley L, Hankins M, et al. Barriers and facilitators of effective self-management in asthma: systematic review and thematic synthesis of patient and healthcare professional views. NPJ Prim Care Respir Med. 2017;27(1):57.

436. Mills K, Harte E, Martin A, MacLure C, Griffin SJ, Mant J, et al. Views of commissioners, managers and healthcare professionals on the NHS Health Check programme: a systematic review. BMJ Open. 2017;7(11):e018606.

437. Milner K, Crawford P, Edgley A, Hare-Duke L, Slade M. The experiences of spirituality among adults with mental health difficulties: a qualitative systematic review. Epidemiol Psychiatr Sci. 2019;29:e34.

438. Mohammed MA, Moles RJ, Chen TF. Medication-related burden and patients' lived experience with medicine: a systematic review and metasynthesis of qualitative studies. BMJ Open. 2016;6(2):e010035.

439. Möhler R, Meyer G. Attitudes of nurses towards the use of physical restraints in geriatric care: a systematic review of qualitative and quantitative studies. Int J Nurs Stud. 2014;51(2):274-88.

440. Montano AR. "All for One" experiences of interprofessional team members caring for older adults: A metasynthesis. International journal of older people nursing. 2020;15(1):e12290.

441. Moore D, Drey N, Ayers S. A meta-synthesis of women's experiences of online forums for maternal mental illness and stigma. Arch Womens Ment Health. 2020;23(4):507-15.

442. Moore DA, Gwernan-Jones R, Richardson M, Racey D, Rogers M, Stein K, et al. The experiences of and attitudes toward non-pharmacological interventions for attention-deficit/hyperactivity disorder used in school settings: a systematic review and synthesis of qualitative research. Emotional and Behavioural Difficulties. 2016;21(1):61-82.

443. Moral-Fernandez L, Frias-Osuna A, Moreno-Camara S, Palomino-Moral PA, Del-Pino-Casado R. The start of caring for an elderly dependent family member: A qualitative metasynthesis. BMC Geriatrics. 2018;18(1).

444. Moran M, Van Cauwenberg J, Hercky-Linnewiel R, Cerin E, Deforche B, Plaut P. Understanding the relationships between the physical environment and physical activity in older adults: a systematic review of qualitative studies. Int J Behav Nutr Phys Act. 2014;11:79.

445. Morgan GS, Willmott M, Ben-Shlomo Y, Haase AM, Campbell RM. A life fulfilled: positively influencing physical activity in older adults - a systematic review and meta-ethnography. BMC Public Health. 2019;19(1):362.

446. Morgan M, Kenten C, Deedat S. Attitudes to deceased organ donation and registration as a donor among minority ethnic groups in North America and the U.K.: a synthesis of quantitative and qualitative research. Ethn Health. 2013;18(4):367-90.

447. Morkunas B, Porritt K, Stephenson M. Experiences of mental health professionals and patients in the use of pro re nata medication in acute adult mental healthcare settings: a systematic review. JBI Database System Rev Implement Rep. 2016;14(10):209-50.

448. Morris H, Larsen J, Catterall E, Moss AC, Dombrowski SU. Peer pressure and alcohol consumption in adults living in the UK: a systematic qualitative review. BMC Public Health. 2020;20(1):1014.

449. Morrison SE, Bruce C, Wilson S. Children's Disclosure of Sexual Abuse: A Systematic Review of Qualitative Research Exploring Barriers and Facilitators. J Child Sex Abus. 2018;27(2):176-94.

450. Morrow KJ, Gustavson AM, Jones J. Speaking up behaviours (safety voices) of healthcare workers: A metasynthesis of qualitative research studies. International Journal of Nursing Studies. 2016;64:42-51.

451. Mu PF, Lee MY, Sheng CC, Tung PC, Huang LY, Chen YW. The experiences of family members in the year following the diagnosis of a child or adolescent with cancer: a qualitative systematic review. JBI Database System Rev Implement Rep. 2015;13(5):293-329.

452. Mu PF, Wang KK, Chen YC, Tsay SF. A systematic review of the experiences of adult ventilator-dependent patients. JBI Libr Syst Rev. 2010;8(8):344-81.

453. Mullet DR, Willerson A, Lamb KN, Kettler T. Examining teacher perceptions of creativity: A systematic review of the literature. Think Skills Creat. 2016;21:9-30.

454. Munabi-Babigumira S, Glenton C, Lewin S, Fretheim A, Nabudere H. Factors that influence the provision of intrapartum and postnatal care by skilled birth attendants in low- and middle-income countries: a qualitative evidence synthesis. Cochrane Database Syst Rev. 2017;11(11):Cd011558.

455. Munabi‐Babigumira S, Glenton C, Lewin S, Fretheim A, Nabudere H. Factors that influence the provision of intrapartum and postnatal care by skilled birth attendants in low‐ and middle‐income countries: a qualitative evidence synthesis. Cochrane Database of Systematic Reviews. 2017(11).

456. Munn Z, Jordan Z. The patient experience of high technology medical imaging: a systematic review of the qualitative evidence. JBI Libr Syst Rev. 2011;9(19):631-78.

457. Munn Z, Tufanaru C, Aromataris E. Recognition of the health assistant as a delegated clinical role and their inclusion in models of care: a systematic review and meta-synthesis of qualitative evidence. Int J Evid Based Healthc. 2013;11(1):3-19.

458. Munodawafa M, Mall S, Lund C, Schneider M. Process evaluations of task sharing interventions for perinatal depression in low and middle income countries (LMIC): a systematic review and qualitative meta-synthesis. BMC Health Serv Res. 2018;18(1):205.

459. Muñoz-Velandia O, Guyatt G, Devji T, Zhang Y, Li SA, Alexander PE, et al. Patient Values and Preferences Regarding Continuous Subcutaneous Insulin Infusion and Artificial Pancreas in Adults with Type 1 Diabetes: A Systematic Review of Quantitative and Qualitative Data. Diabetes Technol Ther. 2019;21(4):183-200.

460. Munro SA, Lewin SA, Smith HJ, Engel ME, Fretheim A, Volmink J. Patient adherence to tuberculosis treatment: a systematic review of qualitative research. PLoS Med. 2007;4(7):e238.

461. Murphy D, Irfan N, Barnett H, Castledine E, Enescu L. A systematic review and meta-synthesis of qualitative research into mandatory personal psychotherapy during training. Couns Psychother Res. 2018;18(2):199-214.

462. Murray CD, McDonald C, Atkin H. The communication experiences of patients with palliative care needs: A systematic review and meta-synthesis of qualitative findings. Palliat Support Care. 2015;13(2):369-83.

463. Musheke M, Ntalasha H, Gari S, McKenzie O, Bond V, Martin-Hilber A, et al. A systematic review of qualitative findings on factors enabling and deterring uptake of HIV testing in Sub-Saharan Africa. BMC Public Health. 2013;13:220.

464. Nalubega S, Evans C. Participant views and experiences of participating in HIV research in sub-Saharan Africa: a qualitative systematic review. JBI Database System Rev Implement Rep. 2015;13(5):330-420.

465. Neale EP, Middleton J, Lambert K. Barriers and enablers to detection and management of chronic kidney disease in primary healthcare: a systematic review. BMC Nephrol. 2020;21(1):83.

466. Newton BJ, Rothlingova Z, Gutteridge R, LeMarchand K, Raphael JH. No room for reflexivity? Critical reflections following a systematic review of qualitative research. J Health Psychol. 2012;17(6):866-85.

467. Nic Giolla Easpaig B, Tran Y, Bierbaum M, Arnolda G, Delaney GP, Liauw W, et al. What are the attitudes of health professionals regarding patient reported outcome measures (PROMs) in oncology practice? A mixed-method synthesis of the qualitative evidence. BMC Health Serv Res. 2020;20(1):102.

468. Nichols VP, Ellard DR, Griffiths FE, Kamal A, Underwood M, Taylor SJC. The lived experience of chronic headache: a systematic review and synthesis of the qualitative literature. BMJ Open. 2017;7(12):e019929.

469. Nichols VP, Toye F, Eldabe S, Sandhu HK, Underwood M, Seers K. Experiences of people taking opioid medication for chronic non-malignant pain: A qualitative evidence synthesis using meta-ethnography. BMJ Open. 2020;10(2).

470. Noble-Carr D, Moore T, McArthur M. The Nature and Extent of Qualitative Research Conducted With Children About Their Experiences of Domestic Violence: Findings From a Meta-Synthesis. Trauma, violence & abuse. 2019:1524838019888885.

471. Nofal SD, Peto TJ, Adhikari B, Tripura R, Callery J, Bui TM, et al. How can interventions that target forest-goers be tailored to accelerate malaria elimination in the Greater Mekong Subregion? A systematic review of the qualitative literature. Malar J. 2019;18(1):32.

472. Nolan M. Masculinity lost: a systematic review of qualitative research on men with spinal cord injury. Spinal Cord. 2013;51(8):588-95.

473. Noonan M, Doody O, Jomeen J, Galvin R. Midwives' perceptions and experiences of caring for women who experience perinatal mental health problems: An integrative review. Midwifery. 2017;45:56-71.

474. Notley C, Blyth A, Craig J, Edwards A, Holland R. Postpartum smoking relapse--a thematic synthesis of qualitative studies. Addiction. 2015;110(11):1712-23.

475. Noyes J, Popay J. Directly observed therapy and tuberculosis: how can a systematic review of qualitative research contribute to improving services? A qualitative meta-synthesis. J Adv Nurs. 2007;57(3):227-43.

476. O'Cathain A, Thomas KJ, Drabble SJ, Rudolph A, Hewison J. What can qualitative research do for randomised controlled trials? A systematic mapping review. BMJ Open. 2013;3(6).

477. O'Connor C, Kadianaki I, Maunder K, McNicholas F. How does psychiatric diagnosis affect young people's self-concept and social identity? A systematic review and synthesis of the qualitative literature. Soc Sci Med. 2018;212:94-119.

478. O'Connor L. How social workers understand and use their emotions in practice: A thematic synthesis literature review. Qualitative Social Work. 2019.

479. O'Donnell A, Addison M, Spencer L, Zurhold H, Rosenkranz M, McGovern R, et al. Which individual, social and environmental influences shape key phases in the amphetamine type stimulant use trajectory? A systematic narrative review and thematic synthesis of the qualitative literature. Addiction. 2019;114(1):24-47.

480. O'Malley M, Carter J, Stamou V, LaFontaine J, Oyebode J, Parkes J. Receiving a diagnosis of young onset dementia: a scoping review of lived experiences. Aging & mental health. 2019:1-12.

481. O'Rourke HM, Duggleby W, Fraser KD, Jerke L. Factors that affect quality of life from the perspective of people with dementia: a metasynthesis. J Am Geriatr Soc. 2015;63(1):24-38.

482. O'Shea LE, Hawkins JE, Lord J, Schmidt-Hansen M, Hasler E, Cameron S, et al. Access to and sustainability of abortion services: a systematic review and meta-analysis for the National Institute of Health and Care Excellence-new clinical guidelines for England. Hum Reprod Update. 2020.

483. Odendaal WA, Anstey Watkins J, Leon N, Goudge J, Griffiths F, Tomlinson M, et al. Health workers' perceptions and experiences of using mHealth technologies to deliver primary healthcare services: a qualitative evidence synthesis. Cochrane Database Syst Rev. 2020;3(3):Cd011942.

484. Ohly H, Gentry S, Wigglesworth R, Bethel A, Lovell R, Garside R. A systematic review of the health and well-being impacts of school gardening: synthesis of quantitative and qualitative evidence. BMC Public Health. 2016;16:286.

485. Okada M, Mitchell P, Finger RP, Eldem B, Talks SJ, Hirst C, et al. Non-adherence or non-persistence to intravitreal injection therapy for neovascular age-related macular degeneration: a mixed-methods systematic review. Ophthalmology. 2020.

486. Okello DR, Gilson L. Exploring the influence of trust relationships on motivation in the health sector: a systematic review. Hum Resour Health. 2015;13:16.

487. Olesen K, Folmann Hempler N, Drejer S, Valeur Baumgarten S, Stenov V. Impact of patient-centred diabetes self-management education targeting people with type 2 diabetes: an integrative review. Diabetic Medicine. 2020;37(6):909-23.

488. Oliveira RM, da Silva LMS, Guedes MVC, Oliveira ACS, Sanchez RG, Torres RAM. Analyzing the concept of disruptive behavior in healthcare work: An integrative review. Revista da Escola de Enfermagem. 2016;50(4):690-9.

489. Ong B, Barnes S, Buus N. Conversation Analysis and Family Therapy: A Critical Review of Methodology. Family Process. 2019.

490. Oni D. Foot Self-Care Experiences Among Patients With Diabetes: A Systematic Review of Qualitative Studies. Wound Manag Prev. 2020;66(4):16-25.

491. Orr N, Wagstaffe A, Briscoe S, Garside R. How do older people describe their sensory experiences of the natural world? A systematic review of the qualitative evidence. BMC Geriatr. 2016;16:116.

492. Ørtenblad L, Carstensen K, Væggemose U, Løvschall C, Sprehn M, Küchen S, et al. Users' Experiences With Home Mechanical Ventilation: A Review of Qualitative Studies. Respir Care. 2019;64(9):1157-68.

493. Owen J, Carroll C, Cooke J, Formby E, Hayter M, Hirst J, et al. School-linked sexual health services for young people (SSHYP): a survey and systematic review concerning current models, effectiveness, cost-effectiveness and research opportunities Introduction. Health Technology Assessment. 2010;14(30):1-+.

494. Ozodiegwu ID, Littleton MA, Nwabueze C, Famojuro O, Quinn M, Wallace R, et al. A qualitative research synthesis of contextual factors contributing to female overweight and obesity over the life course in sub-Saharan Africa. PLoS ONE. 2019;14(11).

495. Page P. Critical illness trajectory for patients, families and nurses - a literature review. Nursing in Critical Care. 2016;21(4):195-205.

496. Palacios-Ceña D, Losa-Iglesias ME, Alvarez-López C, Cachón-Pérez M, Reyes RA, Salvadores-Fuentes P, et al. Patients, intimate partners and family experiences of implantable cardioverter defibrillators: qualitative systematic review. J Adv Nurs. 2011;67(12):2537-50.

497. Parandeh A, Khaghanizade M, Mohammadi E, Mokhtari-Nouri J. Nurses' human dignity in education and practice: An integrated literature review. Iranian Journal of Nursing and Midwifery Research. 2019;21(1):1-8.

498. Parenti G, Tomaino SCM, Cipolletta S. The experience of living with rheumatoid arthritis: A qualitative metasynthesis. Journal of clinical nursing. 2020;23.

499. Park EJ, Kang H. Nurse educators' experiences with student incivility: a meta-synthesis of qualitative studies. J Educ Eval Health Prof. 2020;17:23.

500. Parke H, Michalska M, Russell A, Moss AC, Holdsworth C, Ling J, et al. Understanding drinking among midlife men in the United Kingdom: A systematic review of qualitative studies. Addict Behav Rep. 2018;8:85-94.

501. Parker D, Byng R, Dickens C, Kinsey D, McCabe R. Barriers and facilitators to GP-patient communication about emotional concerns in UK primary care: a systematic review. Fam Pract. 2020.

502. Parker G, Gridley K, Birks Y, Glanville J. Using a systematic review to uncover theory and outcomes for a complex intervention in health and social care: a worked example using life story work for people with dementia. J Health Serv Res Policy. 2020:1355819619897091.

503. Parker LS, Topcu G, De Boos D, das Nair R. The notion of "invisibility" in people's experiences of the symptoms of multiple sclerosis: a systematic meta-synthesis. Disability and rehabilitation. 2020:1-15.

504. Parker S, Hopkins G, Siskind D, Harris M, McKeon G, Dark F, et al. A systematic review of service models and evidence relating to the clinically operated community-based residential mental health rehabilitation for adults with severe and persisting mental illness in Australia. BMC Psychiatry. 2019;19(1):55.

505. Parry S, Simpson J. How Do Adult Survivors of Childhood Sexual Abuse Experience Formally Delivered Talking Therapy? A Systematic Review. J Child Sex Abus. 2016;25(7):793-812.

506. Parslow RM, Harris S, Broughton J, Alattas A, Crawley E, Haywood K, et al. Children's experiences of chronic fatigue syndrome/myalgic encephalomyelitis (CFS/ME): a systematic review and meta-ethnography of qualitative studies. BMJ Open. 2017;7(1):e012633.

507. Parsons K, Gaudine A, Swab M. Older nurses' experiences of providing direct care in hospital nursing units: a qualitative systematic review. JBI Database System Rev Implement Rep. 2018;16(3):669-700.

508. Patel T, Christy K, Grierson L, Shadd J, Farag A, O'Toole D, et al. Clinician responses to legal requests for hastened death: a systematic review and meta-synthesis of qualitative research. BMJ Support Palliat Care. 2020.

509. Patterson J, Hollins Martin C, Karatzias T. PTSD post-childbirth: a systematic review of women's and midwives' subjective experiences of care provider interaction. J Reprod Infant Psychol. 2019;37(1):56-83.

510. Patton DU, Hong JS, Patel S, Kral MJ. A Systematic Review of Research Strategies Used in Qualitative Studies on School Bullying and Victimization. Trauma Violence Abuse. 2017;18(1):3-16.

511. Paul J, Metcalfe S, Stirling L, Wilson B, Hodgson J. Analyzing communication in genetic consultations--a systematic review. Patient Educ Couns. 2015;98(1):15-33.

512. Paul JL, Leslie H, Trainer AH, Gaff C. A theory-informed systematic review of clinicians' genetic testing practices. Eur J Hum Genet. 2018;26(10):1401-16.

513. Pearson A, Laschinger H, Porritt K, Jordan Z, Tucker D, Long L. Comprehensive systematic review of evidence on developing and sustaining nursing leadership that fosters a healthy work environment in healthcare. Int J Evid Based Healthc. 2007;5(2):208-53.

514. Pearson A, Srivastava R, Craig D, Tucker D, Grinspun D, Bajnok I, et al. Systematic review on embracing cultural diversity for developing and sustaining a healthy work environment in healthcare. Int J Evid Based Healthc. 2007;5(1):54-91.

515. Peat G, Rodriguez A, Smith J. Social media use in adolescents and young adults with serious illnesses: An integrative review. BMJ Supportive and Palliative Care. 2018.

516. Peek ST, Wouters EJ, van Hoof J, Luijkx KG, Boeije HR, Vrijhoef HJ. Factors influencing acceptance of technology for aging in place: a systematic review. Int J Med Inform. 2014;83(4):235-48.

517. Pell C, Straus L, Andrew EV, Meñaca A, Pool R. Social and cultural factors affecting uptake of interventions for malaria in pregnancy in Africa: a systematic review of the qualitative research. PLoS One. 2011;6(7):e22452.

518. Perreira TA, Innis J, Berta W. Work motivation in health care: A scoping literature review. International Journal of Evidence-Based Healthcare. 2016;14(4):175-82.

519. Peters J, Parletta N, Campbell K, Lynch J. Parental influences on the diets of 2- to 5-year-old children: Systematic review of qualitative research. J Early Child Res. 2014;12(1):3-19.

520. Petersen MN, Jessen-Winge C, Møbjerg ACM. Scandinavian women's experiences with abortions on request: a systematic review. JBI Database System Rev Implement Rep. 2018;16(7):1537-63.

521. Peterson AJ, Bonell C. School experiences and young women's pregnancy and parenthood decisions: A systematic review and synthesis of qualitative research. Health Place. 2018;53:52-61.

522. Petit dit Dariel O, Cristofalo P. A meta-ethnographic review of interprofessional teamwork in hospitals: what it is and why it doesn't happen more often. Journal of Health Services Research and Policy. 2018;23(4):272-9.

523. Petriwskyj A, Parker D, O'Dwyer S, Moyle W, Nucifora N. Interventions to build resilience in family caregivers of people living with dementia: a comprehensive systematic review. JBI Database System Rev Implement Rep. 2016;14(6):238-73.

524. Pfeffer D, Wigginton B, Gartner C, Morphett K. Smokers' Understandings of Addiction to Nicotine and Tobacco: A Systematic Review and Interpretive Synthesis of Quantitative and Qualitative Research. Nicotine Tob Res. 2018;20(9):1038-46.

525. Pindus DM, Mullis R, Lim L, Wellwood I, Rundell AV, Abd Aziz NA, et al. Stroke survivors' and informal caregivers' experiences of primary care and community healthcare services - A systematic review and meta-ethnography. PLoS One. 2018;13(2):e0192533.

526. Pinheiro MAP, Seles B, Fiorini PD, Jugend D, Jabbour A, da Silva HMR, et al. The role of new product development in underpinning the circular economy A systematic review and integrative framework. Manag Decis. 2019;57(4):840-62.

527. Pinilla S, Lenouvel E, Strik W, Klöppel S, Nissen C, Huwendiek S. Entrustable Professional Activities in Psychiatry: A Systematic Review. Acad Psychiatry. 2020;44(1):37-45.

528. Pirnejad H, Bal R, Shahsavar N. The nature of unintended effects of health information systems concerning patient safety: a systematic review with thematic synthesis. Stud Health Technol Inform. 2010;160(Pt 1):719-23.

529. Plastow NA, Bester J. Embedding graduate attributes during occupational therapy curriculum development: A scoping review and qualitative research synthesis. Aust Occup Ther J. 2020.

530. Pocock M, Trivedi D, Wills W, Bunn F, Magnusson J. Parental perceptions regarding healthy behaviours for preventing overweight and obesity in young children: a systematic review of qualitative studies. Obes Rev. 2010;11(5):338-53.

531. Pollard TM, Guell C, Morris S. Communal therapeutic mobility in group walking: A meta-ethnography. Social Science and Medicine. 2020;262 (no pagination).

532. Pooya A, Chaghoushi AJ, Shokohyar S, Karimizand M. THE MODEL OF CHALLENGES OF SMART CONTRACT BASED ON BLOCKCHAIN TECHNOLOGY AND DISTRIBUTED LEDGER USING META-SYNTHESIS RESEARCH METHOD. Rev Genero Direito. 2020;9(3):821-44.

533. Popejoy E, Pollock K, Almack K, Manning JC, Johnston B. Decision-making and future planning for children with life-limiting conditions: a qualitative systematic review and thematic synthesis. Child Care Health Dev. 2017;43(5):627-44.

534. Poyser CA, Tickle A. Exploring the experience of the disclosure of a dementia diagnosis from a clinician, patient and carer perspective: a systematic review and Meta-ethnographic synthesis. Aging Ment Health. 2019;23(12):1605-15.

535. Pratt R, Stephenson J, Mann S. What influences contraceptive behaviour in women who experience unintended pregnancy? A systematic review of qualitative research. J Obstet Gynaecol. 2014;34(8):693-9.

536. Price A, Janssens A, Woodley AL, Allwood M, Ford T. Review: Experiences of healthcare transitions for young people with attention deficit hyperactivity disorder: a systematic review of qualitative research. Child Adolesc Ment Health. 2019;24(2):113-22.

537. Provenzi L, Santoro E. The lived experience of fathers of preterm infants in the Neonatal Intensive Care Unit: a systematic review of qualitative studies. J Clin Nurs. 2015;24(13-14):1784-94.

538. Pulst A, Fassmer AM, Schmiemann G. Experiences and involvement of family members in transfer decisions from nursing home to hospital: a systematic review of qualitative research. BMC Geriatr. 2019;19(1):155.

539. Punt MC, Aalders TH, Bloemenkamp KWM, Driessens MHE, Fischer K, Schrijvers MH, et al. The experiences and attitudes of hemophilia carriers around pregnancy: A qualitative systematic review. J Thromb Haemost. 2020;18(7):1626-36.

540. Pylyser C, Buysse A, Loeys T. Stepfamilies Doing Family: A Meta-Ethnography. Fam Process. 2018;57(2):496-509.

541. Qin Y, Han L, Babbitt A, Walker JS, Liu F, Thirumurthy H, et al. Experiences using and organizing HIV self-testing. Aids. 2018;32(3):371-81.

542. Qiu D, Hu M, Yu Y, Tang B, Xiao S. Acceptability of psychosocial interventions for dementia caregivers: a systematic review. BMC Psychiatry. 2019;19(1):23.

543. Quay TAW, Frimer L, Janssen PA, Lamers Y. Barriers and facilitators to recruitment of South Asians to health research: A scoping review. BMJ Open. 2017;7(5).

544. Raftery J, Bryant J, Powell J, Kerr C, Hawker S. Payment to healthcare professionals for patient recruitment to trials: systematic review and qualitative study. Health Technol Assess. 2008;12(10):1-128, iii.

545. Rai SK, Choi HK, Choi SHJ, Townsend AF, Shojania K, De Vera MA. Key barriers to gout care: a systematic review and thematic synthesis of qualitative studies. Rheumatology (Oxford). 2018;57(7):1282-92.

546. Rapaport P, Livingston G, Murray J, Mulla A, Cooper C. Systematic review of the effective components of psychosocial interventions delivered by care home staff to people with dementia. BMJ Open. 2017;7(2):e014177.

547. Raque-Bogdan TL, Lamphere B, Kostiuk M, Gissen M, Beranek M. Unpacking the layers: a meta-ethnography of cancer survivors' loneliness. J Cancer Surviv. 2019;13(1):21-33.

548. Rashid MA, Edwards D, Walter FM, Mant J. Medication taking in coronary artery disease: a systematic review and qualitative synthesis. Ann Fam Med. 2014;12(3):224-32.

549. Rashid MA, Llanwarne N, Heyns N, Walter F, Mant J. What are the implications for practice that arise from studies of medication taking? A systematic review of qualitative research. PLoS One. 2018;13(5):e0195076.

550. Rashidi A, Kaistha P, Whitehead L, Robinson S. Factors that influence adherence to treatment plans amongst people living with cardiovascular disease: A review of published qualitative research studies. Int J Nurs Stud. 2020;110:103727.

551. Rathbone AP, Todd A, Jamie K, Bonam M, Banks L, Husband AK. A systematic review and thematic synthesis of patients' experience of medicines adherence. Res Social Adm Pharm. 2017;13(3):403-39.

552. Raveel A, Schoenmakers B. Interventions to prevent aggression against doctors: a systematic review. BMJ Open. 2019;9(9):e028465.

553. Rawlings GH, Reuber M. What patients say about living with psychogenic nonepileptic seizures: A systematic synthesis of qualitative studies. Seizure. 2016;41:100-11.

554. Rawlings GH, Reuber M. Health care practitioners' perceptions of psychogenic nonepileptic seizures: A systematic review of qualitative and quantitative studies. Epilepsia. 2018;59(6):1109-23.

555. Rawlings GH, Williams RK, Clarke DJ, English C, Fitzsimons C, Holloway I, et al. Exploring adults' experiences of sedentary behaviour and participation in non-workplace interventions designed to reduce sedentary behaviour: A thematic synthesis of qualitative studies. BMC Public Health. 2019;19(1).

556. Rawson TM, Moore LSP, Hernandez B, Charani E, Castro-Sanchez E, Herrero P, et al. A systematic review of clinical decision support systems for antimicrobial management: are we failing to investigate these interventions appropriately? Clin Microbiol Infect. 2017;23(8):524-32.

557. Raybould G, Babatunde O, Evans AL, Jordan JL, Paskins Z. Expressed information needs of patients with osteoporosis and/or fragility fractures: a systematic review. Arch Osteoporos. 2018;13(1):55.

558. Raymond A, Lee SF, Bloomer MJ. Understanding the bereavement care roles of nurses within acute care: a systematic review. J Clin Nurs. 2017;26(13-14):1787-800.

559. Raynor DK, Blenkinsopp A, Knapp P, Grime J, Nicolson DJ, Pollock K, et al. A systematic review of quantitative and qualitative research on the role and effectiveness of written information available to patients about individual medicines. Health Technol Assess. 2007;11(5):iii, 1-160.

560. Reay G, Norris JM, Nowell L, Hayden KA, Yokom K, Lang ES, et al. Transition in Care from EMS Providers to Emergency Department Nurses: A Systematic Review. Prehosp Emerg Care. 2020;24(3):421-33.

561. Recine AG, Recine L, Paldon T. How People Forgive: A Systematic Review of Nurse-Authored Qualitative Research. J Holist Nurs. 2020;38(2):233-51.

562. Rees N, Rapport F, Snooks H. Perceptions of paramedics and emergency staff about the care they provide to people who self-harm: Constructivist metasynthesis of the qualitative literature. Journal of Psychosomatic Research. 2015;78(6):529-35.

563. Rees RW, Caird J, Dickson K, Vigurs C, Thomas J. 'It's on your conscience all the time': a systematic review of qualitative studies examining views on obesity among young people aged 12-18 years in the UK. BMJ Open. 2014;4(4):e004404.

564. Rees S, Williams A. Promoting and supporting self-management for adults living in the community with physical chronic illness: A systematic review of the effectiveness and meaningfulness of the patient-practitioner encounter. JBI Libr Syst Rev. 2009;7(13):492-582.

565. Regan L, Preston NJ, Eccles FJR, Simpson J. The views of adults with neurodegenerative diseases on end-of-life care: a metasynthesis. Aging Ment Health. 2019;23(2):149-57.

566. Rehnström Loi U, Gemzell-Danielsson K, Faxelid E, Klingberg-Allvin M. Health care providers' perceptions of and attitudes towards induced abortions in sub-Saharan Africa and Southeast Asia: a systematic literature review of qualitative and quantitative data. BMC Public Health. 2015;15:139.

567. Reis S, Hermoni D, Van-Raalte R, Dahan R, Borkan JM. Aggregation of qualitative studies--From theory to practice: Patient priorities and family medicine/general practice evaluations. Patient Educ Couns. 2007;65(2):214-22.

568. Renzi C, Whitaker KL, Wardle J. Over-reassurance and undersupport after a 'false alarm': a systematic review of the impact on subsequent cancer symptom attribution and help seeking. BMJ Open. 2015;5(2):e007002.

569. Rhea DJ, Lockwood S. Adults surviving lung cancer two or more years: A systematic review. JBI Libr Syst Rev. 2012;10(34):2297-349.

570. Rhoden MA, Macgowan MJ, Huang H. A Systematic Review of Psychological Trauma Interventions for Juvenile Offenders. Research on Social Work Practice. 2019;29(8):892-909.

571. Ricci-Cabello I, Bobrow K, Islam SMS, Chow CK, Maddison R, Whittaker R, et al. Examining Development Processes for Text Messaging Interventions to Prevent Cardiovascular Disease: Systematic Literature Review. JMIR Mhealth Uhealth. 2019;7(3):e12191.

572. Richards DA, Hanssen TA, Borglin G. The Second Triennial Systematic Literature Review of European Nursing Research: Impact on Patient Outcomes and Implications for Evidence-Based Practice. Worldviews Evid Based Nurs. 2018;15(5):333-43.

573. Ricoy-Cano AJ, Obrero-Gaitán E, Caravaca-Sánchez F, Fuente-Robles YM. Factors Conditioning Sexual Behavior in Older Adults: A Systematic Review of Qualitative Studies. J Clin Med. 2020;9(6).

574. Ridd M, Shaw A, Lewis G, Salisbury C. The patient-doctor relationship: a synthesis of the qualitative literature on patients' perspectives. Br J Gen Pract. 2009;59(561):e116-33.

575. Riggs DM, Killingback C. What factors influence physical activity participation in people with rheumatoid arthritis? Physical Therapy Reviews. 2019;24(6):298-307.

576. Ripat J, Verdonck M, Gacek C, McNicol S. A qualitative metasynthesis of the meaning of speech-generating devices for people with complex communication needs. AAC: Augmentative and Alternative Communication. 2019;35(2):69-79.

577. Rittenmeyer L, Huffman D. How professional nurses working in hospital environments experience moral distress: a systematic review. JBI Libr Syst Rev. 2009;7(28):1234-91.

578. Rittenmeyer L, Huffman D, Hopp L, Block M. A comprehensive systematic review on the experience of lateral/horizontal violence in the profession of nursing. JBI Library of Systematic Reviews. 2013;11(11):362-468.

579. Robards F, Kang M, Usherwood T, Sanci L. How Marginalized Young People Access, Engage With, and Navigate Health-Care Systems in the Digital Age: Systematic Review. J Adolesc Health. 2018;62(4):365-81.

580. Robart R, Boyle P. Supporting workers with lower back injuries to return to work: a meta-ethnography. British Journal of Occupational Therapy. 2020.

581. Robert R, Stavinoha P, Jones BL, Robinson J, Larson K, Hicklen R, et al. Spiritual assessment and spiritual care offerings as a standard of care in pediatric oncology: A recommendation informed by a systematic review of the literature. Pediatr Blood Cancer. 2019;66(9):e27764.

582. Roberts J, Evans K, Spiby H, Evans C, Pallotti P, Eldridge J. Women's information needs, decision-making and experiences of membrane sweeping to promote spontaneous labour. Midwifery. 2020;83:102626.

583. Robertson L, McGee R, Marsh L, Hoek J. A systematic review on the impact of point-of-sale tobacco promotion on smoking. Nicotine Tob Res. 2015;17(1):2-17.

584. Robinson L, Hutchings D, Corner L, Finch T, Hughes J, Brittain K, et al. Balancing rights and risks: Conflicting perspectives in the management of wandering in dementia. Health, Risk and Society. 2007;9(4):389-406.

585. Roche M, Higgs P, Aworinde J, Cooper C. A Review of Qualitative Research of Perception and Experiences of Dementia Among Adults From Black, African, and Caribbean Background: What and Whom Are We Researching? The Gerontologist. 2020;20.

586. Rocheleau JN, Cobigo V, Chalghoumi H, Jahan A, Jutai J, Lake J, et al. Factors affecting information technology use from the perspective of aging persons with cognitive disabilities: A scoping review of qualitative research. Technology and Disability. 2020;32(1):1-13.

587. Rodrigues P, Menten J, Gastmans C. Physicians' perceptions of palliative sedation for existential suffering: a systematic review. BMJ Support Palliat Care. 2020;10(2):136-44.

588. Rodríguez-Prat A, Balaguer A, Booth A, Monforte-Royo C. Understanding patients' experiences of the wish to hasten death: an updated and expanded systematic review and meta-ethnography. BMJ Open. 2017;7(9):e016659.

589. Rogers K, Coleman H, Brodtmann A, Darby D, Anderson V. Family members' experience of the pre-diagnostic phase of dementia: A synthesis of qualitative evidence. International Psychogeriatrics. 2017;29(9):1425-37.

590. Roll CL, Cheater F. Expectant parents' views of factors influencing infant feeding decisions in the antenatal period: A systematic review. Int J Nurs Stud. 2016;60:145-55.

591. Rooney JM. Compassion in mental health: a literature review. Mental Health and Social Inclusion. 2020.

592. Rose MA, Vukicevic M, Koklanis K, Rees G, Sandhu S, Itsiopoulos C. Experiences and perceptions of patients undergoing treatment and quality of life impact of diabetic macular edema: a systematic review. Psychol Health Med. 2019;24(4):383-401.

593. Ross-Walker C, Rogers-Clark C, Pearce S. A systematic review of Registered Nurses; experiences of the influence of workplace culture and climatic factors on nursing workloads. JBI Libr Syst Rev. 2012;10(49):3080-145.

594. Rowland E, Metcalfe A. Communicating inherited genetic risk between parent and child: a meta-thematic synthesis. Int J Nurs Stud. 2013;50(6):870-80.

595. Rubtsova AA, Kempf MC, Taylor TN, Konkle-Parker D, Wingood GM, Holstad MM. Healthy Aging in Older Women Living with HIV Infection: a Systematic Review of Psychosocial Factors. Curr HIV/AIDS Rep. 2017;14(1):17-30.

596. Rushforth B, McCrorie C, Glidewell L, Midgley E, Foy R. Barriers to effective management of type 2 diabetes in primary care: qualitative systematic review. Br J Gen Pract. 2016;66(643):e114-27.

597. Russell S, Ogunbayo OJ, Newham JJ, Heslop-Marshall K, Netts P, Hanratty B, et al. Qualitative systematic review of barriers and facilitators to self-management of chronic obstructive pulmonary disease: views of patients and healthcare professionals. NPJ Prim Care Respir Med. 2018;28(1):2.

598. S OD, Riordain RN. The patient experience of dental implant surgery: a literature review of pertinent qualitative studies. Irish Journal of Medical Science. 2020.

599. Sadler E, Potterton V, Anderson R, Khadjesari Z, Sheehan K, Butt F, et al. Service user, carer and provider perspectives on integrated care for older people with frailty, and factors perceived to facilitate and hinder implementation: A systematic review and narrative synthesis. PLoS One. 2019;14(5):e0216488.

600. Sagoe D, Andreassen CS, Pallesen S. The aetiology and trajectory of anabolic-androgenic steroid use initiation: a systematic review and synthesis of qualitative research. Subst Abuse Treat Prev Policy. 2014;9:27.

601. Sagoe D, McVeigh J, Bjørnebekk A, Essilfie MS, Andreassen CS, Pallesen S. Polypharmacy among anabolic-androgenic steroid users: a descriptive metasynthesis. Subst Abuse Treat Prev Policy. 2015;10:12.

602. Sakaguchi-Tang DK, Bosold AL, Choi YK, Turner AM. Patient Portal Use and Experience Among Older Adults: Systematic Review. JMIR Med Inform. 2017;5(4):e38.

603. Salami B, Hirani SAA, Meherali S, Amodu O, Chambers T. Parenting Practices of African Immigrants in Destination Countries: A Qualitative Research Synthesis. Journal of Pediatric Nursing. 2017;36:20-30.

604. Saleem A, Steadman KJ, Fejzic J. Utilisation of Healthcare Services and Medicines by Pakistani Migrants Residing in High Income Countries: A Systematic Review and Thematic Synthesis. J Immigr Minor Health. 2019;21(5):1157-80.

605. Saletti-Cuesta L, Aizenberg L, Ricci-Cabello I. Opinions and Experiences of Primary Healthcare Providers Regarding Violence against Women: a Systematic Review of Qualitative Studies. J Fam Violence. 2018;33(6):405-20.

606. Sam S, Sharma R, Corp N, Igwesi-Chidobe C, Babatunde OO. Shared decision making in musculoskeletal pain consultations in low- and middle-income countries: a systematic review. Int Health. 2020;12(5):455-71.

607. Sambrook Smith M, Lawrence V, Sadler E, Easter A. Barriers to accessing mental health services for women with perinatal mental illness: systematic review and meta-synthesis of qualitative studies in the UK. BMJ Open. 2019;9(1):e024803.

608. Sambunjak D, Straus SE, Marusic A. A systematic review of qualitative research on the meaning and characteristics of mentoring in academic medicine. J Gen Intern Med. 2010;25(1):72-8.

609. Samoborec S, Ruseckaite R, Ayton D, Evans S. Biopsychosocial factors associated with non-recovery after a minor transport-related injury: A systematic review. PLoS One. 2018;13(6):e0198352.

610. Sanchez RV, Speck PM, Patrician PA. A Concept Analysis of Trauma Coercive Bonding in the Commercial Sexual Exploitation of Children. Journal of pediatric nursing. 2019;46:48-54.

611. Sangaleti C, Schveitzer MC, Peduzzi M, Zoboli E, Soares CB. Experiences and shared meaning of teamwork and interprofessional collaboration among health care professionals in primary health care settings: a systematic review. JBI Database System Rev Implement Rep. 2017;15(11):2723-88.

612. Santer M, Ring N, Yardley L, Geraghty AW, Wyke S. Treatment non-adherence in pediatric long-term medical conditions: systematic review and synthesis of qualitative studies of caregivers' views. BMC Pediatr. 2014;14:63.

613. Sapthiang S, Van Gordon W, Shonin E. Health School-based Mindfulness Interventions for Improving Mental Health: A Systematic Review and Thematic Synthesis of Qualitative Studies. Journal of Child and Family Studies. 2019;28(10):2650-8.

614. Sarkies MN, Bowles KA, Skinner EH, Haas R, Lane H, Haines TP. The effectiveness of research implementation strategies for promoting evidence-informed policy and management decisions in healthcare: a systematic review. Implement Sci. 2017;12(1):132.

615. Sarmento VP, Gysels M, Higginson IJ, Gomes B. Home palliative care works: But how? A meta-ethnography of the experiences of patients and family caregivers. BMJ Supportive and Palliative Care. 2017;7(4):390-403.

616. Sathanapally H, Sidhu M, Fahami R, Gillies C, Kadam U, Davies MJ, et al. Priorities of patients with multimorbidity and of clinicians regarding treatment and health outcomes: a systematic mixed studies review. BMJ Open. 2020;10(2):e033445.

617. Satink T, Cup EH, Ilott I, Prins J, De Swart BJ, Nijhuis-Van Der Sanden MW. Patients' views on the impact of stroke on their roles and self: A thematic synthesis of qualitative studies. Archives of Physical Medicine and Rehabilitation. 2013;94(6):1171-83.

618. Scamell M, Ghumman A. The experience of maternity care for migrant women living with female genital mutilation: A qualitative synthesis. Birth. 2019;46(1):15-23.

619. Schiekirka S, Raupach T. A systematic review of factors influencing student ratings in undergraduate medical education course evaluations. BMC Med Educ. 2015;15:30.

620. Schjoedt I, Sommer I, Bjerrum MB. Experiences and management of fatigue in everyday life among adult patients living with heart failure: a systematic review of qualitative evidence. JBI Database System Rev Implement Rep. 2016;14(3):68-115.

621. Schmid W, Rosland JH, Von Hofacker S, Hunskar I, Bruvik F. Patient's and health care provider's perspectives on music therapy in palliative care - An integrative review. BMC Palliative Care. 2018;17(1).

622. Schmid-Mohler G, Yorke J, Spirig R, Benden C, Caress AL. Adult patients' experiences of symptom management during pulmonary exacerbations in cystic fibrosis: A thematic synthesis of qualitative research. Chronic Illness. 2019;15(4):245-63.

623. Schumann I, Schneider A, Kantert C, Löwe B, Linde K. Physicians' attitudes, diagnostic process and barriers regarding depression diagnosis in primary care: a systematic review of qualitative studies. Fam Pract. 2012;29(3):255-63.

624. Schunter BT, Cheng WS, Kendall M, Marais H. Lessons learned from a review of interventions for adolescent and young key populations in Asia Pacific and opportunities for programming. Journal of Acquired Immune Deficiency Syndromes. 2014;66(SUPPL. 2):S186-S92.

625. Scope A, Booth A, Sutcliffe P. Women's perceptions and experiences of group cognitive behaviour therapy and other group interventions for postnatal depression: a qualitative synthesis. J Adv Nurs. 2012;68(9):1909-19.

626. Scott S, Elamin W, Giles EL, Hillier-Brown F, Byrnes K, Connor N, et al. Socio-Ecological Influences on Adolescent (Aged 10-17) Alcohol Use and Unhealthy Eating Behaviours: A Systematic Review and Synthesis of Qualitative Studies. Nutrients. 2019;11(8).

627. Seidler ZE, Dawes AJ, Rice SM, Oliffe JL, Dhillon HM. The role of masculinity in men's help-seeking for depression: A systematic review. Clin Psychol Rev. 2016;49:106-18.

628. Selva Olid A, Zurro AM, Villa JJ, Hijar AM, Tuduri XM, Puime AO, et al. Medical students' perceptions and attitudes about family practice: a qualitative research synthesis. BMC Med Educ. 2012;12:81.

629. Senderovich H, Retnasothie S. A systematic review of the integration of palliative care in dementia management. Palliat Support Care. 2019:1-12.

630. Senra H, Ali Z, Balaskas K, Aslam T. Psychological impact of anti-VEGF treatments for wet macular degeneration-a review. Graefe's Archive for Clinical and Experimental Ophthalmology. 2016;254(10):1873-80.

631. Seo K, Song Y. Self-stigma among Korean patients with diabetes: A concept analysis. Journal of Clinical Nursing. 2019;28(9-10):1794-807.

632. Seow H, Bainbridge D. A Review of the Essential Components of Quality Palliative Care in the Home. J Palliat Med. 2018;21(S1):S37-s44.

633. Setiawan J, Mannix T, Sweet L. Understanding the Effects of Neonatal Early Discharge on Parents: A Literature Review. Journal of Perinatal and Neonatal Nursing. 2019;33(2):170-88.

634. Seyedfatemi N, Salsali M, Rezaee N, Rahnavard Z. Women's Health Concept: A Meta-Synthesis Study. Iran J Public Health. 2014;43(10):1335-44.

635. Shabnam J, Timm H, Nielsen DS, Raunkiaer M. Palliative care for older South Asian migrants: A systematic review. Palliat Support Care. 2020;18(3):346-58.

636. Shahmalak U, Blakemore A, Waheed MW, Waheed W. The experiences of lay health workers trained in task-shifting psychological interventions: a qualitative systematic review. Int J Ment Health Syst. 2019;13:64.

637. Shankar KN, Bhatia BK, Schuur JD. Toward patient-centered care: a systematic review of older adults' views of quality emergency care. Ann Emerg Med. 2014;63(5):529-50.e1.

638. Sharif MO, Callery P, Tierney S. The perspectives of children and young people living with cleft lip and palate: a review of qualitative literature. Cleft Palate Craniofac J. 2013;50(3):297-304.

639. Shaw RL, Holland C, Pattison HM, Cooke R. Patients' perceptions and experiences of cardiovascular disease and diabetes prevention programmes: A systematic review and framework synthesis using the Theoretical Domains Framework. Soc Sci Med. 2016;156:192-203.

640. Sheals K, Tombor I, McNeill A, Shahab L. A mixed-method systematic review and meta-analysis of mental health professionals' attitudes toward smoking and smoking cessation among people with mental illnesses. Addiction. 2016;111(9):1536-53.

641. Shen RZZ, Xiong P, Chou UI, Hall BJ. "We need them as much as they need us": A systematic review of the qualitative evidence for possible mechanisms of effectiveness of animal-assisted intervention (AAI). Complement Ther Med. 2018;41:203-7.

642. Shennan C, Payne S, Fenlon D. What is the evidence for the use of mindfulness-based interventions in cancer care? A review. Psychooncology. 2011;20(7):681-97.

643. Shersher V, Haines TP, Sturgiss L, Weller C, Williams C. Definitions and use of the teach-back method in healthcare consultations with patients: A systematic review and thematic synthesis. Patient Educ Couns. 2020.

644. Shi W, Shen Z, Wang S, Hall BJ. Barriers to Professional Mental Health Help-Seeking Among Chinese Adults: A Systematic Review. Front Psychiatry. 2020;11:442.

645. Shields L, Pratt J, Davis LM, Hunter J. Family-centred care for children in hospital. Cochrane Database Syst Rev. 2007(1):Cd004811.

646. Shiells K, Holmerova I, Steffl M, Stepankova O. Electronic patient records as a tool to facilitate care provision in nursing homes: an integrative review. Informatics for Health and Social Care. 2019;44(3):262-77.

647. Shiells K, Pivodic L, Holmerova I, Van den Block L. Self-reported needs and experiences of people with dementia living in nursing homes: a scoping review. Aging & mental health. 2019:1-16.

648. Shim J, Brindle L, Simon M, George S. A systematic review of symptomatic diagnosis of lung cancer. Fam Pract. 2014;31(2):137-48.

649. Shimpuku Y, Norr KF. Working with interpreters in cross-cultural qualitative research in the context of a developing country: systematic literature review. J Adv Nurs. 2012;68(8):1692-706.

650. Shipton L, Zahidie A, Rabbani F. Motivating and Demotivating Factors for Community Health Workers Engaged in Maternal, Newborn and Child Health Programs in Low and Middle-Income Countries: A Systematic Review. J Coll Physicians Surg Pak. 2017;27(3):157-65.

651. Shorey S, Chan V. Lessons from past epidemics and pandemics and a way forward for pregnant women, midwives and nurses during COVID-19 and beyond: A meta-synthesis. Midwifery. 2020;90:102821.

652. Shrestha P, Fick DM. Family caregiver's experience of caring for an older adult with delirium: A systematic review. Int J Older People Nurs. 2020:e12321.

653. Siddiqi Z, Tiro JA, Shuval K. Understanding impediments and enablers to physical activity among African American adults: a systematic review of qualitative studies. Health Educ Res. 2011;26(6):1010-24.

654. Siddiqui NY, Levin PJ, Phadtare A, Pietrobon R, Ammarell N. Perceptions about female urinary incontinence: a systematic review. Int Urogynecol J. 2014;25(7):863-71.

655. Siegner M, Hagerman S, Kozak R. Going deeper with documents: A systematic review of the application of extant texts in social research on forests. Forest Policy Econ. 2018;92:128-35.

656. Sierra Leguia L, Montoya Juarez R, Garcia Caro MP, Lopez Morales M, Montalvo Prieto A. Family caregivers experience with the palliative care's and end-of-life. [Spanish]. Index de Enfermeria. 2019;28(1-2):51-5.

657. Siltanen H, Jylhä V, Holopainen A, Paavilainen E. Family members' experiences and expectations of self-management counseling while caring for a person with chronic obstructive pulmonary disease: a systematic review of qualitative evidence. JBI Database System Rev Implement Rep. 2019;17(11):2214-47.

658. Simkhada B, Teijlingen ER, Porter M, Simkhada P. Factors affecting the utilization of antenatal care in developing countries: systematic review of the literature. J Adv Nurs. 2008;61(3):244-60.

659. Sin J, Norman I. Psychoeducational interventions for family members of people with schizophrenia: a mixed-method systematic review. J Clin Psychiatry. 2013;74(12):e1145-62.

660. Singh JS, Bunyak G. Autism Disparities: A Systematic Review and Meta-Ethnography of Qualitative Research. Qual Health Res. 2019;29(6):796-808.

661. Singh S, Ehsani-Chimeh N, Kornmehl H, Armstrong AW. Quality of life among dermatology patients: a systematic review of investigations using qualitative methods. G Ital Dermatol Venereol. 2019;154(1):72-8.

662. Sinković M, Towler L. Sexual Aging: A Systematic Review of Qualitative Research on the Sexuality and Sexual Health of Older Adults. Qual Health Res. 2019;29(9):1239-54.

663. Sinnott C, Kelly MA, Bradley CP. A scoping review of the potential for chart stimulated recall as a clinical research method. BMC Health Services Research. 2017;17(1).

664. Sirdifield C, Anthierens S, Creupelandt H, Chipchase SY, Christiaens T, Siriwardena AN. General practitioners' experiences and perceptions of benzodiazepine prescribing: systematic review and meta-synthesis. BMC Fam Pract. 2013;14:191.

665. Sirdifield C, Chipchase SY, Owen S, Siriwardena AN. A Systematic Review and Meta-Synthesis of Patients' Experiences and Perceptions of Seeking and Using Benzodiazepines and Z-Drugs: Towards Safer Prescribing. Patient. 2017;10(1):1-15.

666. Slade M, Leamy M, Bacon F, Janosik M, Le Boutillier C, Williams J, et al. International differences in understanding recovery: systematic review. Epidemiol Psychiatr Sci. 2012;21(4):353-64.

667. Small S, Porr C, Swab M, Murray C. Experiences and cessation needs of Indigenous women who smoke during pregnancy: a systematic review of qualitative evidence. JBI Database System Rev Implement Rep. 2018;16(2):385-452.

668. Smiddy MP, R OC, Creedon SA. Systematic qualitative literature review of health care workers' compliance with hand hygiene guidelines. Am J Infect Control. 2015;43(3):269-74.

669. Smit A, Coetzee BJ, Roomaney R, Bradshaw M, Swartz L. Women's stories of living with breast cancer: A systematic review and meta-synthesis of qualitative evidence. Soc Sci Med. 2019;222:231-45.

670. Smith AL, Carter SM, Dunlop SM, Freeman B, Chapman S. The views and experiences of smokers who quit smoking unassisted. A systematic review of the qualitative evidence. PLoS One. 2015;10(5):e0127144.

671. Smith EC, Holmes L, Burkle FM. Exploring the Physical and Mental Health Challenges Associated with Emergency Service Call-Taking and Dispatching: A Review of the Literature. Prehosp Disaster Med. 2019;34(6):619-24.

672. Smith EC, Holmes L, Burkle FM. The Physical and Mental Health Challenges Experienced by 9/11 First Responders and Recovery Workers: A Review of the Literature. Prehosp Disaster Med. 2019;34(6):625-31.

673. Smith JE, Richardson J, Hoffman C, Pilkington K. Mindfulness-Based Stress Reduction as supportive therapy in cancer care: systematic review. J Adv Nurs. 2005;52(3):315-27.

674. Smith LA, Kolokotroni KZ, Turner-Moore T. Making and Communicating Decisions About Sexual Consent During Drug-Involved Sex: A Thematic Synthesis. Journal of sex research. 2020:1-19.

675. Smith M, Signal L, Edwards R, Hoek J. Children's and parents' opinions on the sport-related food environment: a systematic review. Obes Rev. 2017;18(9):1018-39.

676. SmithBattle L, Punsuwun S, Phengnum W. An Umbrella Review of Qualitative Research on Teen Mothering. Western journal of nursing research. 2020:193945920943213.

677. Smithson J, Garside R, Pearson M. Barriers to, and facilitators of, the prevention of unintentional injury in children in the home: a systematic review and synthesis of qualitative research. Inj Prev. 2011;17(2):119-26.

678. Sng QW, He HG, Wang W, Taylor B, Chow A, Klainin-Yobas P, et al. A Meta-Synthesis of Children's Experiences of Postoperative Pain Management. Worldviews Evid Based Nurs. 2017;14(1):46-54.

679. Snippen NC, de Vries HJ, van der Burg-Vermeulen SJ, Hagedoorn M, Brouwer S. Influence of significant others on work participation of individuals with chronic diseases: a systematic review. BMJ Open. 2019;9(1):e021742.

680. Snow A, Cerel J, Loeffler DN, Flaherty C. Barriers to Mental Health Care for Transgender and Gender-Nonconforming Adults: A Systematic Literature Review. Health Soc Work. 2019;44(3):149-55.

681. Soekhai V, de Bekker-Grob EW, Ellis AR, Vass CM. Discrete Choice Experiments in Health Economics: Past, Present and Future. Pharmacoeconomics. 2019;37(2):201-26.

682. Soilemezi D, Drahota A, Crossland J, Stores R. The Role of the Home Environment in Dementia Care and Support: Systematic review of Qualitative Research. Dementia (London). 2019;18(4):1237-72.

683. Solstad SM, Castonguay LG, Moltu C. Patients' experiences with routine outcome monitoring and clinical feedback systems: A systematic review and synthesis of qualitative empirical literature. Psychother Res. 2019;29(2):157-70.

684. Song JE, Ahn JA, Kim T, Roh EH. A qualitative review of immigrant women's experiences of maternal adaptation in South Korea. Midwifery. 2016;39:35-43.

685. Song M, Kong EH. Older adults' definitions of health: A metasynthesis. Int J Nurs Stud. 2015;52(6):1097-106.

686. South J, Woodall J, Kinsella K, Bagnall AM. A qualitative synthesis of the positive and negative impacts related to delivery of peer-based health interventions in prison settings. BMC Health Serv Res. 2016;16(1):525.

687. Spaling MA, Currie K, Strachan PH, Harkness K, Clark AM. Improving support for heart failure patients: a systematic review to understand patients' perspectives on self-care. J Adv Nurs. 2015;71(11):2478-89.

688. Spence T, Kander I, Walsh J, Griffiths F, Ross J. Perceptions and Experiences of Internet-Based Testing for Sexually Transmitted Infections: Systematic Review and Synthesis of Qualitative Research. J Med Internet Res. 2020;22(8):e17667.

689. Spendelow JS, Eli Joubert H, Lee H, Fairhurst BR. Coping and adjustment in men with prostate cancer: a systematic review of qualitative studies. J Cancer Surviv. 2018;12(2):155-68.

690. Spendelow JS, Seidler ZE. Men's Self-Initiated Coping Strategies Across Medical, Psychological, and Psychosocial Issues: A Systematic Review. Psychol Men Masculinities. 2020;21(1):106-23.

691. Spreadbury JH, Kipps C. Measuring younger onset dementia: What the qualitative literature reveals about the 'lived experience' for patients and caregivers. Dementia. 2019;18(2):579-98.

692. Sprogis SK, Currey J, Considine J. Patient acceptability of wearable vital sign monitoring technologies in the acute care setting: A systematic review. J Clin Nurs. 2019;28(15-16):2732-44.

693. Stapleton A, Wright N. The experiences of people with borderline personality disorder admitted to acute psychiatric inpatient wards: a meta-synthesis. J Ment Health. 2019;28(4):443-57.

694. Steege R, Taegtmeyer M, McCollum R, Hawkins K, Ormel H, Kok M, et al. How do gender relations affect the working lives of close to community health service providers? Empirical research, a review and conceptual framework. Soc Sci Med. 2018;209:1-13.

695. Stellar C, Garcia-Moreno C, Temmerman M, van der Poel S. A systematic review and narrative report of the relationship between infertility, subfertility, and intimate partner violence. Int J Gynaecol Obstet. 2016;133(1):3-8.

696. Stephen JM, Zoucha R. Spanish Speaking, Limited English Proficient Parents whose Children are Hospitalized: An Integrative Review. Journal of pediatric nursing. 2020;52:30-40.

697. Stephenson MD, Campbell JM, Lisy K, Aromataris EC. Assessing healthcare professionals' experiences of integrated care: Do surveys tell the full story? International Journal of Evidence-Based Healthcare. 2017;15(3):90-101.

698. Stern C, Pearson A, Chur-Hansen A. The Appropriateness of Canine-Assisted Interventions (CAIs) on the Health and Social Care of Older People Residing in Long Term Care: A Systematic Review. JBI Libr Syst Rev. 2011;9(33):1367-92.

699. Stevens M, Manthorpe J, Martineau S. What motivates and discourages social workers from working as Approved Mental Health Professionals? Evidence about job resources and demands of the Approved Mental Health Professional role. Journal of Social Work. 2019.

700. Stevenson M, Achille M, Lugasi T. Pediatric palliative care in Canada and the United States: A qualitative metasummary of the needs of patients and families. Journal of Palliative Medicine. 2013;16(5):566-77.

701. Stewart M, Ryan SJ. Do metaphors have therapeutic value for people in pain? A systematic review. Pain and Rehabilitation. 2019;2020(48):10-23.

702. Stewart S, Guillen AG, Taylor WJ, Gaffo A, Slark J, Gott M, et al. The experience of a gout flare: a meta-synthesis of qualitative studies. Seminars in Arthritis and Rheumatism. 2020;50(4):805-11.

703. Stickley T, Wright N, Slade M. The art of recovery: outcomes from participatory arts activities for people using mental health services. Journal of Mental Health. 2018;27(4):367-73.

704. Stockford C, Stenfert Kroese B, Beesley A, Leung N. Women's recovery from anorexia nervosa: a systematic review and meta-synthesis of qualitative research. Eat Disord. 2019;27(4):343-68.

705. Stockton J, Nield L. An antenatal wish list: A qualitative systematic review and thematic synthesis of UK dietary advice for weight management and food borne illness. Midwifery. 2020;82:102624.

706. Stormon N, Kazantzis N, Ford PJ, Lalloo R. Children's oral health in Australia: The past decade's research agenda. Community Dent Oral Epidemiol. 2019;47(2):153-61.

707. Strachan PH, Currie K, Harkness K, Spaling M, Clark AM. Context matters in heart failure self-care: a qualitative systematic review. J Card Fail. 2014;20(6):448-55.

708. Straus SE, Soobiah C, Levinson W. The impact of leadership training programs on physicians in academic medical centers: a systematic review. Acad Med. 2013;88(5):710-23.

709. Striberger R, Axelsson M, Zarrouk M, Kumlien C. Illness perceptions in patients with peripheral arterial disease: A systematic review of qualitative studies. Int J Nurs Stud. 2020:103723.

710. Stuart SR, Tansey L, Quayle E. What we talk about when we talk about recovery: a systematic review and best-fit framework synthesis of qualitative literature. J Ment Health. 2017;26(3):291-304.

711. Stub T, Quandt SA, Arcury TA, Sandberg JC, Kristoffersen AE, Musial F, et al. Perception of risk and communication among conventional and complementary health care providers involving cancer patients' use of complementary therapies: A literature review. BMC Complementary and Alternative Medicine. 2016;16(1).

712. Su Y, Yuki M, Hirayama K. The experiences and perspectives of family surrogate decision-makers: A systematic review of qualitative studies. Patient Educ Couns. 2020;103(6):1070-81.

713. Subasinghe AK, Deb S, Mazza D. Primary care providers' knowledge, attitudes and practices of medical abortion: a systematic review. BMJ Sex Reprod Health. 2019.

714. Subramanian GS, Manoharan VS. IMPORTANCE OF PHYSICAL ACTIVITY IN OBESE CHILDREN IN RELATION TO MOTOR SKILLS A SYSTEMATIC REVIEW. Int J Physiother. 2014;1(5):304-9.

715. Sujatha G, Muruganandhan J, Priya VV, Srinivasan MR. Determination of reliability and practicality of saliva as a genetic source in forensic investigation by analyzing DNA yield and success rates: A systematic review. J Oral Maxillofac Surg Med Pathol. 2019;31(3):218-27.

716. Suleman Z, Evans C, Manning JC. Parents' and carers' experiences of transition and aftercare following a child's discharge from a paediatric intensive care unit to an in-patient ward setting: A qualitative systematic review. Intensive Crit Care Nurs. 2019;51:35-44.

717. Sulistyono RE, Wahyudi AS, Tristiana RD. Perceived burden of multidrugs- resistant tuberculosis patients and their family: A systematic review of the qualitative literature. International Journal of Psychosocial Rehabilitation. 2020;24(7):9050-8.

718. Svensson MK, Wahlberg A, Gislason GH. Chronic Paradoxes: A Systematic Review of Qualitative Family Perspectives on Living With Congenital Heart Defects. Qual Health Res. 2020;30(1):119-32.

719. Swaithes L, Paskins Z, Dziedzic K, Finney A. Factors influencing the implementation of evidence-based guidelines for osteoarthritis in primary care: A systematic review and thematic synthesis. Musculoskeletal Care. 2020;18(2):101-10.

720. Sweeney A, Clarke N, Higgs M. Shared Leadership in Commercial Organizations: A Systematic Review of Definitions, Theoretical Frameworks and Organizational Outcomes. Int J Manag Rev. 2019;21(1):115-36.

721. Sweeney A, Clement S, Gribble K, Jackson E, Carr S, Catty J, et al. A systematic review of qualitative studies of adults' experiences of being assessed for psychological therapies. Health Expect. 2019;22(2):133-48.

722. Tagoe N, Molyneux S, Pulford J, Murunga VI, Kinyanjui S. Managing health research capacity strengthening consortia: a systematised review of the published literature. BMJ Glob Health. 2019;4(2):e001318.

723. Tang Yan HS, Clemson LM, Jarvis F, Laver K. Goal setting with caregivers of adults in the community: a mixed methods systematic review. Disabil Rehabil. 2014;36(23):1943-63.

724. Tate KJ, Newbury-Birch D, McGeechan GJ. A systematic review of qualitative evidence of cancer patients' attitudes to mindfulness. Eur J Cancer Care (Engl). 2018;27(2):e12783.

725. Tay LH, Ong AKW, Lang DSP. Experiences of adult cancer patients receiving counseling from nurses: a qualitative systematic review. JBI Database System Rev Implement Rep. 2018;16(10):1965-2012.

726. Te V, Griffiths R, Law K, Hill PS, Annear PL. The impact of ASEAN economic integration on health worker mobility: A scoping review of the literature. Health Policy and Planning. 2018;33(8):957-65.

727. Teixeira CAB, Lasiuk G, Barton S, Fernandes MNF, Gherardi-Donato ECS. An exploration of addiction in adults experiencing early-life stress: A metasynthesis. Revista Latino-Americana de Enfermagem. 2017;25 (no pagination).

728. Tennyson RE, Griffiths HC. A Systematic Review of Professionals' Experiences of Discussing Fertility Issues with Adolescents and Young Adults with Cancer. J Adolesc Young Adult Oncol. 2019;8(4):387-97.

729. Teper MH, Vedel I, Yang XQ, Margo-Dermer E, Hudon C. Understanding Barriers to and Facilitators of Case Management in Primary Care: A Systematic Review and Thematic Synthesis. Ann Fam Med. 2020;18(4):355-63.

730. Terry DR, Nguyen H, Peck B, Smith A, Phan H. Communities of practice: A systematic review and meta-synthesis of what it means and how it really works among nursing students and novices. J Clin Nurs. 2020;29(3-4):370-80.

731. Teskereci G, Kulakaç O. Life experiences of caregivers of women with gynaecological cancer: a mixed-methods systematic review. Eur J Cancer Care (Engl). 2018;27(1).

732. Thomas A, Sowerbutts AM, Burden ST. The impact of home enteral feeding on the daily lives of people with head and neck cancer: a metasynthesis of qualitative studies. Journal of human nutrition and dietetics : the official journal of the British Dietetic Association. 2020;33(4):538-49.

733. Thomas BE, Shanmugam P, Malaisamy M, Ovung S, Suresh C, Subbaraman R, et al. Psycho-Socio-Economic Issues Challenging Multidrug Resistant Tuberculosis Patients: A Systematic Review. PLoS One. 2016;11(1):e0147397.

734. Thomas GEC, Crutch SJ, Camic PM. Measuring physiological responses to the arts in people with a dementia. International Journal of Psychophysiology. 2018;123:64-73.

735. Thomas H, Mitchell G, Rich J, Best M. Definition of whole person care in general practice in the English language literature: a systematic review. BMJ Open. 2018;8(12):e023758.

736. Thomas J, Harden A. Methods for the thematic synthesis of qualitative research in systematic reviews. BMC Medical Research Methodology. 2008;8 (no pagination).

737. Thomas J, Harden A, Oakley A, Oliver S, Sutcliffe K, Rees R, et al. Integrating qualitative research with trials in systematic reviews. British Medical Journal. 2004;328(7446):1010-2.

738. Thompson AP, Nesari M, Hartling L, Scott SD. Parents' experiences and information needs related to childhood fever: A systematic review. Patient Educ Couns. 2020;103(4):750-63.

739. Thompson AR, Sewards I, Baker SR. Cancer and changes in facial appearance: A meta-ethnography of qualitative studies. Br J Health Psychol. 2020;25(1):129-51.

740. Thompson J, Stansfeld JL, Cooper RE, Morant N, Crellin NE, Moncrieff J. Experiences of taking neuroleptic medication and impacts on symptoms, sense of self and agency: a systematic review and thematic synthesis of qualitative data. Soc Psychiatry Psychiatr Epidemiol. 2020;55(2):151-64.

741. Thompson J, Yoward S, Dawson P. The Role of Physiotherapy Extended Scope Practitioners in Musculoskeletal care with Focus on Decision Making and Clinical Outcomes: A Systematic Review of Quantitative and Qualitative Research. Musculoskeletal Care. 2017;15(2):91-103.

742. Thomson G, Flacking R, George K, Feeley N, Haslund-Thomsen H, De Coen K, et al. Parents' experiences of emotional closeness to their infants in the neonatal unit: A meta-ethnography. Early Hum Dev. 2020;149:105155.

743. Thomson L, Stanyon M, Dening T, Heron R, Griffiths A. Managing employees with dementia: a systematic review. Occup Med (Lond). 2019;69(2):89-98.

744. Thorn H, Uhrenfeldt L. Experiences of non-specialist nurses caring for patients and their significant others undergoing transitions during palliative end-of-life cancer care: a systematic review. JBI Database System Rev Implement Rep. 2017;15(6):1711-46.

745. Threapleton DE, Chung RY, Wong SYS, Wong E, Chau P, Woo J, et al. Integrated care for older populations and its implementation facilitators and barriers: A rapid scoping review. International Journal for Quality in Health Care. 2017;29(3):327-34.

746. Thurston MD, Allan S. Sexuality and sexual experiences during gender transition: A thematic synthesis. Clin Psychol Rev. 2018;66:39-50.

747. Tierney S, Mamas M, Skelton D, Woods S, Rutter MK, Gibson M, et al. What can we learn from patients with heart failure about exercise adherence? A systematic review of qualitative papers. Health Psychol. 2011;30(4):401-10.

748. Timulak L. Identifying core categories of client-identified impact of helpful events in psychotherapy: A qualitative meta-analysis. Psychotherapy Research. 2007;17(3):305-14.

749. Tindall RM, Simmons MB, Allott K, Hamilton BE. Essential ingredients of engagement when working alongside people after their first episode of psychosis: A qualitative meta-synthesis. Early Interv Psychiatry. 2018;12(5):784-95.

750. Tiranda Y, Siripul P, Sangchart B, Septiwi C. Perspectives of adult survivors of colorectal cancer with an ostomy on their needs: Synthesis of qualitative research studies. Central European Journal of Nursing and Midwifery. 2019;10(4):1155-66.

751. Tirmizi LIT, Son R, New CY, Brand H. The effectiveness of food handler training programmes in malaysia and ireland to prevent food-borne disease. Food Research. 2018;2(3):247-57.

752. Tobiano G, Bucknall T, Sladdin I, Whitty JA, Chaboyer W. Patient participation in nursing bedside handover: A systematic mixed-methods review. Int J Nurs Stud. 2018;77:243-58.

753. Tobin CL, Di Napoli P, Beck CT. Refugee and Immigrant Women's Experience of Postpartum Depression: A Meta-Synthesis. J Transcult Nurs. 2018;29(1):84-100.

754. Toft BS, Uhrenfeldt L. The lived experiences of being physically active when morbidly obese: A qualitative systematic review. Int J Qual Stud Health Well-being. 2015;10:28577.

755. Tomagova M, Lepiesova M, Borikova I, Cap J, Nemcova J, Zanovitova M. Real-life experiences of patients with Parkinson's disease. Kontakt. 2019;21(3):269-78.

756. Tombor I, Shahab L, Herbec A, Neale J, Michie S, West R. Smoker Identity and Its Potential Role in Young Adults' Smoking Behavior: A Meta-ethnography. Health Psychology. 2015;26.

757. Topcu G, Buchanan H, Aubeeluck A, Garip G. Caregiving in multiple sclerosis and quality of life: A meta-synthesis of qualitative research. Psychology and Health. 2016:1-18.

758. Townsend IM, Berger EP, Reupert AE. Systematic review of the educational experiences of children in care: Children's perspectives. Children and Youth Services Review. 2020;111:10.

759. Townsend L, Flisher AJ, King G. A systematic review of the relationship between high school dropout and substance use. Clin Child Fam Psychol Rev. 2007;10(4):295-317.

760. Toye F, Seers K, Allcock N, Briggs M, Carr E, Andrews J, et al. Patients' experiences of chronic non-malignant musculoskeletal pain: a qualitative systematic review. Br J Gen Pract. 2013;63(617):e829-41.

761. Toye F, Seers K, Barker KL. Living life precariously with rheumatoid arthritis - a mega-ethnography of nine qualitative evidence syntheses. BMC Rheumatol. 2019;3:5.

762. Tranberg R, Alexander S, Hatcher D, Mackey S, Shahid S, Holden L, et al. Factors influencing cancer treatment decision-making by indigenous peoples: a systematic review. Psychooncology. 2016;25(2):131-41.

763. Travis E, Ashley L, Pownall M, O'Connor DB. Barriers to flexible sigmoidoscopy colorectal cancer screening in low uptake socio-demographic groups: A systematic review. Psychooncology. 2020;29(8):1237-47.

764. Troyer L, Brady W. Barriers to effective EMS to emergency department information transfer at patient handover: A systematic review. Am J Emerg Med. 2020;38(7):1494-503.

765. Truglio-Londrigan M, Slyer JT, Singleton JK, Worral PS. A qualitative systematic review of internal and external influences on shared decision-making in all health care settings. JBI Database of Systematic Reviews and Implementation Reports. 2014;12(5):121-94.

766. Tsai C, Blinkhorn A, Irving M. Oral Health Programmes in Indigenous Communities Worldwide-Lessons learned from the field: A qualitative systematic review. Community Dent Oral Epidemiol. 2017;45(5):389-97.

767. Tseliou E, Burck C, Forbat L, Strong T, O'Reilly M. The Discursive Performance of Change Process in Systemic and Constructionist Therapies: A Systematic Meta-Synthesis Review of In-Session Therapy Discourse. Family process. 2020;30.

768. Tuijt R, Rees J, Frost R, Wilcock J, Manthorpe J, Rait G, et al. Exploring how triads of people living with dementia, carers and health care professionals function in dementia health care: A systematic qualitative review and thematic synthesis. Dementia. 2020.

769. Uhrenfeldt L, Fegran L, Aagaard H, Ludvigsen MS. Significant others' experience of hospitalized patients' transfer to home: A systematic review and meta-synthesis. Z Evid Fortbild Qual Gesundhwes. 2018;139:1-9.

770. Underwood F, Burrows L, Gegg R, Latour JM, Kent B. The meaning of confidence for older people living with frailty: a qualitative systematic review. JBI Database System Rev Implement Rep. 2017;15(5):1316-49.

771. Usher-Smith JA, Harte E, MacLure C, Martin A, Saunders CL, Meads C, et al. Patient experience of NHS health checks: a systematic review and qualitative synthesis. BMJ Open. 2017;7(8):e017169.

772. Valdez RS, McGuire KM, Rivera AJ. Qualitative ergonomics/human factors research in health care: Current state and future directions. Appl Ergon. 2017;62:43-71.

773. van Buijtene A, Foster D. Does a hospital culture influence adherence to infection prevention and control and rates of healthcare associated infection? A literature review. Journal of Infection Prevention. 2018.

774. van den Bogaard K, Lugtenberg M, Nijs S, Embregts P. Attributions of People with Intellectual Disabilities of Their Own or Other Clients' Challenging Behavior: A Systematic Review of Qualitative Studies. J Ment Health Res Intellect Disabil. 2019;12(3-4):126-51.

775. Van Dort BA, Zheng WY, Baysari MT. Prescriber perceptions of medication-related computerized decision support systems in hospitals: A synthesis of qualitative research. Int J Med Inform. 2019;129:285-95.

776. Van Hecke L, Ghekiere A, Veitch J, Van Dyck D, Van Cauwenberg J, Clarys P, et al. Public open space characteristics influencing adolescents' use and physical activity: A systematic literature review of qualitative and quantitative studies. Health Place. 2018;51:158-73.

777. van Leeuwen A, Janssen J. A systematic review of teacher guidance during collaborative learning in primary and secondary education. Educ Res Rev. 2019;27:71-89.

778. Van Ryswyk E, Middleton P, Hague W, Crowther C. Clinician views and knowledge regarding healthcare provision in the postpartum period for women with recent gestational diabetes: a systematic review of qualitative/survey studies. Diabetes Res Clin Pract. 2014;106(3):401-11.

779. Van Wesel F, Boeije H, Alisic E, Drost S. I'll be working my way back: A qualitative synthesis on the trauma experience of children. Psychological Trauma: Theory, Research, Practice, and Policy. 2012;4(5):516-26.

780. Vanassche E, Kelchtermans G. The state of the art in Self-Study of Teacher Education Practices: a systematic literature review. J Curric Stud. 2015;47(4):508-28.

781. Vandewalle J, Debyser B, Beeckman D, Vandecasteele T, Van Hecke A, Verhaeghe S. Peer workers' perceptions and experiences of barriers to implementation of peer worker roles in mental health services: A literature review. International Journal of Nursing Studies. 2016;60:234-50.

782. Vanstone M, Cernat A, Majid U, Trivedi F, De Freitas C. Perspectives of Pregnant People and Clinicians on Noninvasive Prenatal Testing: A Systematic Review and Qualitative Meta-synthesis. Ont Health Technol Assess Ser. 2019;19(5):1-38.

783. Vanstone M, Kandasamy S, Giacomini M, DeJean D, McDonald SD. Pregnant women's perceptions of gestational weight gain: A systematic review and meta-synthesis of qualitative research. Matern Child Nutr. 2017;13(4).

784. Vanstone M, Rewegan A, Brundisini F, Dejean D, Giacomini M. Patient Perspectives on Quality of Life With Uncontrolled Type 1 Diabetes Mellitus: A Systematic Review and Qualitative Meta-synthesis. Ont Health Technol Assess Ser. 2015;15(17):1-29.

785. Vanstone M, Rewegan A, Brundisini F, Giacomini M, Kandasamy S, DeJean D. Diet modification challenges faced by marginalized and nonmarginalized adults with type 2 diabetes: A systematic review and qualitative meta-synthesis. Chronic Illn. 2017;13(3):217-35.

786. Vasileiou K, Barnett J, Thorpe S, Young T. Characterising and justifying sample size sufficiency in interview-based studies: systematic analysis of qualitative health research over a 15-year period. BMC Med Res Methodol. 2018;18(1):148.

787. Vass C, Gray E, Payne K. Discrete choice experiments of pharmacy services: a systematic review. Int J Clin Pharm. 2016;38(3):620-30.

788. Vass C, Rigby D, Payne K. The Role of Qualitative Research Methods in Discrete Choice Experiments. Med Decis Making. 2017;37(3):298-313.

789. Vassie C, Smith S, Leedham-Green K. Factors impacting on retention, success and equitable participation in clinical academic careers: A scoping review and meta-thematic synthesis. BMJ Open. 2020;10(3).

790. Vaughn LM, Whetstone C, Boards A, Busch MD, Magnusson M, Määttä S. Partnering with insiders: A review of peer models across community-engaged research, education and social care. Health Soc Care Community. 2018;26(6):769-86.

791. Vaughn VM, Saint S, Krein SL, Forman JH, Meddings J, Ameling J, et al. Characteristics of healthcare organisations struggling to improve quality: results from a systematic review of qualitative studies. BMJ Qual Saf. 2019;28(1):74-84.

792. Venning A, Eliott J, Wilson A, Kettler L. Understanding young peoples' experience of chronic illness: a systematic review. Int J Evid Based Healthc. 2008;6(3):321-36.

793. Ventura-Garcia L, Roura M, Pell C, Posada E, Gascón J, Aldasoro E, et al. Socio-cultural aspects of Chagas disease: a systematic review of qualitative research. PLoS Negl Trop Dis. 2013;7(9):e2410.

794. Verbrugghe M, Verhaeghe S, Lauwaert K, Beeckman D, Van Hecke A. Determinants and associated factors influencing medication adherence and persistence to oral anticancer drugs: a systematic review. Cancer Treat Rev. 2013;39(6):610-21.

795. Viken B, Solum EM, Lyberg A. Foreign educated nurses' work experiences and patient safety-A systematic review of qualitative studies. Nurs Open. 2018;5(4):455-68.

796. Vindrola-Padros C, Johnson GA. Rapid Techniques in Qualitative Research: A Critical Review of the Literature. Qual Health Res. 2020;30(10):1596-604.

797. Vindrola-Padros C, Vindrola-Padros B. Quick and dirty? A systematic review of the use of rapid ethnographies in healthcare organisation and delivery. BMJ Qual Saf. 2018;27(4):321-30.

798. Völlm B, Konappa N. The dangerous and severe personality disorder experiment--review of empirical research. Crim Behav Ment Health. 2012;22(3):165-80.

799. von der Lippe C, Diesen PS, Feragen KB. Living with a rare disorder: a systematic review of the qualitative literature. Mol Genet Genomic Med. 2017;5(6):758-73.

800. Voss I, Bartlett R. Seeking freedom: A systematic review and thematic synthesis of the literature on patients' experience of absconding from hospital. J Psychiatr Ment Health Nurs. 2019;26(9-10):289-300.

801. Vottero B, Rittenmeyer L. The hospitalised patients' experience of being in protective/source isolation: A systematic review of qualitative evidence. JBI Libr Syst Rev. 2012;10(16):935-76.

802. Vrbnjak D, Denieffe S, O'Gorman C, Pajnkihar M. Barriers to reporting medication errors and near misses among nurses: A systematic review. Int J Nurs Stud. 2016;63:162-78.

803. Vrinten C, McGregor LM, Heinrich M, von Wagner C, Waller J, Wardle J, et al. What do people fear about cancer? A systematic review and meta-synthesis of cancer fears in the general population. Psychooncology. 2017;26(8):1070-9.

804. Wainwright M, Colvin CJ, Swartz A, Leon N. Self-management of medical abortion: a qualitative evidence synthesis. Reprod Health Matters. 2016;24(47):155-67.

805. Waite S, Ecker J, Ross LE. A systematic review and thematic synthesis of Canada's LGBTQ2S+ employment, labour market and earnings literature. PLoS One. 2019;14(10):e0223372.

806. Walczak A, McDonald F, Patterson P, Dobinson K, Allison K. How does parental cancer affect adolescent and young adult offspring? A systematic review. Int J Nurs Stud. 2018;77:54-80.

807. Walker S, Rossi D, Anastasi J, Gray-Ganter G, Tennent R. Indicators of undergraduate nursing students' satisfaction with their learning journey: An integrative review. Nurse Education Today. 2016;43:40-8.

808. Walker SB, Rossi DM, Sander TM. Women's successful transition to motherhood during the early postnatal period: A qualitative systematic review of postnatal and midwifery home care literature. Midwifery. 2019;79:102552.

809. Wall P, Fetherston C, Browne C. Understanding the enrolled nurse to registered nurse journey through a model adapted from Schlossberg's transition theory. Nurse Education Today. 2018;67:6-14.

810. Walpole G, Clark H, Dowling M. Myeloma patients' experiences of haematopoietic stem cell transplant: A qualitative thematic synthesis. European Journal of Oncology Nursing. 2018;35:15-21.

811. Walsh M, Galvin R, Horgan NF. Fall-related experiences of stroke survivors: a meta-ethnography. Disabil Rehabil. 2017;39(7):631-40.

812. Walsh RA, Tzelepis F. Adolescents and tobacco use: systematic review of qualitative research methodologies and partial synthesis of findings. Subst Use Misuse. 2007;42(8):1269-321.

813. Walter FM, Emery J, Braithwaite D, Marteau TM. Lay understanding of familial risk of common chronic diseases: a systematic review and synthesis of qualitative research. Ann Fam Med. 2004;2(6):583-94.

814. Wanat M, Boulton M, Watson E. Patients' experience with cancer recurrence: A meta-ethnography. Psycho-Oncology. 2016;25(3):242-52.

815. Wand APF, Peisah C, Draper B, Brodaty H. Understanding self-harm in older people: a systematic review of qualitative studies. Aging Ment Health. 2018;22(3):289-98.

816. Wang H, Swain S, Luo J, Blake H, Chattopadhyay K. Barriers and facilitators to physical activity among ethnic Chinese children: a qualitative systematic review. JBI Evid Synth. 2020.

817. Wang HF, Yeh MC. Psychological resistance to insulin therapy in adults with type 2 diabetes: mixed-method systematic review. J Adv Nurs. 2012;68(4):743-57.

818. Wardle H. Perceptions, people and place: Findings from a rapid review of qualitative research on youth gambling. Addict Behav. 2019;90:99-106.

819. Watkins R, Goodwin VA, Abbott RA, Backhouse A, Moore D, Tarrant M. Attitudes, perceptions and experiences of mealtimes among residents and staff in care homes for older adults: A systematic review of the qualitative literature. Geriatr Nurs. 2017;38(4):325-33.

820. Watson J, Fossey E, Harvey C. A home but how to connect with others? A qualitative meta-synthesis of experiences of people with mental illness living in supported housing. Health & social care in the community. 2019;27(3):546-64.

821. Watts LL, Todd EM, Mulhearn TJ, Medeiros KE, Mumford MD, Connelly S. Qualitative Evaluation Methods in Ethics Education: A Systematic Review and Analysis of Best Practices. Account Res. 2017;24(4):225-42.

822. Weaver AJ, Flannelly LT, Garbarino J, Figley CR, Flannelly KJ. A systematic review of research on religion and spirituality in the Journal of Traumatic Stress: 1990-1999. Mental Health, Religion and Culture. 2003;6(3):215-28.

823. Webbe J, Brunton G, Ali S, Longford N, Modi N, Gale C. Parent, patient and clinician perceptions of outcomes during and following neonatal care: a systematic review of qualitative research. BMJ Paediatr Open. 2018;2(1):e000343.

824. Weckesser A, Denny E. Women living with epilepsy, experiences of pregnancy and reproductive health: A review of the literature. Seizure. 2013;22(2):91-8.

825. Westergren T, Berntsen S, Ludvigsen MS, Aagaard H, Hall EOC, Ommundsen Y, et al. Relationship between physical activity level and psychosocial and socioeconomic factors and issues in children and adolescents with asthma: A scoping review. JBI Database of Systematic Reviews and Implementation Reports. 2017;15(8):2182-222.

826. Wetzler S, Hackmann C, Peryer G, Clayman K, Friedman D, Saffran K, et al. A framework to conceptualize personal recovery from eating disorders: A systematic review and qualitative meta-synthesis of perspectives from individuals with lived experience. Int J Eat Disord. 2020;53(8):1188-203.

827. Wheelwright S, Darlington AS, Hopkinson JB, Fitzsimmons D, Johnson C. A systematic review and thematic synthesis of quality of life in the informal carers of cancer patients with cachexia. Palliat Med. 2016;30(2):149-60.

828. Wheelwright SJ, Darlington AS, Hopkinson JB, Fitzsimmons D, White A, Johnson CD. A systematic review to establish health-related quality-of-life domains for intervention targets in cancer cachexia. BMJ Support Palliat Care. 2016;6(3):307-14.

829. Whiteley D, Elliott L, Cunningham-Burley S, Whittaker A. Health-Related Quality of Life for individuals with hepatitis C: A narrative review. International Journal of Drug Policy. 2015;26(10):936-49.

830. Whitley GA, Hemingway P, Law GR, Jones AW, Curtis F, Siriwardena AN. The predictors, barriers and facilitators to effective management of acute pain in children by emergency medical services: A systematic mixed studies review. Journal of Child Health Care. 2020.

831. Whittemore R, Jaser S, Chao A, Jang M, Grey M. Psychological experience of parents of children with type 1 diabetes: a systematic mixed-studies review. Diabetes Educ. 2012;38(4):562-79.

832. Wilcox S, Ananian CD, Sharpe PA, Robbins J, Brady T. Correlates of physical activity in persons with arthritis: Review and recommendations. Journal of Physical Activity and Health. 2005;2(2):230-52.

833. Wiles R, Cott C, Gibson BE. Hope, expectations and recovery from illness: A narrative synthesis of qualitative research. Journal of Advanced Nursing. 2008;64(6):564-73.

834. Wilkinson A, Whitehead L, Ritchie L. Factors influencing the ability to self-manage diabetes for adults living with type 1 or 2 diabetes. Int J Nurs Stud. 2014;51(1):111-22.

835. Willcox M, Donovan E, Hu XY, Elboray S, Jerrard N, Roberts N, et al. Views regarding use of complementary therapies for acute respiratory infections: Systematic review of qualitative studies. Complement Ther Med. 2020;50:102382.

836. Williams JE, Gifford W, Vanderspank-Wright B, Phillips JC. Violence and Health Promotion Among First Nations, Métis, and Inuit Women: A Systematic Review of Qualitative Research. Trauma Violence Abuse. 2019:1524838019875696.

837. Williamson LM, Parkes A, Wight D, Petticrew M, Hart GJ. Limits to modern contraceptive use among young women in developing countries: a systematic review of qualitative research. Reprod Health. 2009;6:3.

838. Willis E, Lawn S, Roberts L, Couzner L, Mohammadi L, Goble E. The impact of emergency call taking on the mental health and wellbeing of ambulance call-takers: A systematic thematic narrative of qualitative research. Australasian Journal of Paramedicine. 2020;17:1-11.

839. Wilson C, Cariola LA. LGBTQI+ Youth and Mental Health: A Systematic Review of Qualitative Research. Adolescent Research Review. 2020;5(2):187-211.

840. Wilson PA, Valera P, Martos AJ, Wittlin NM, Muñoz-Laboy MA, Parker RG. Contributions of Qualitative Research in Informing HIV/AIDS Interventions Targeting Black MSM in the United States. J Sex Res. 2016;53(6):642-54.

841. Winn A, Hetherington E, Tough S. Systematic Review of Immigrant Women's Experiences With Perinatal Care in North America. J Obstet Gynecol Neonatal Nurs. 2017;46(5):764-75.

842. Winnette R, Hess LM, Nicol SJ, Tai DF, Copley-Merriman C. The Patient Experience with Soft Tissue Sarcoma: A Systematic Review of the Literature. Patient. 2017;10(2):153-62.

843. Winsor S, Smith A, Vanstone M, Giacomini M, Brundisini FK, DeJean D. Experiences of patient-centredness with specialized community-based care: a systematic review and qualitative meta-synthesis. Ont Health Technol Assess Ser. 2013;13(17):1-33.

844. Wion RK, Loeb SJ. CE: Original Research: End-of-Life Care Behind Bars: A Systematic Review. Am J Nurs. 2016;116(3):24-36; quiz 7.

845. Wittenberg Y, Kwekkeboom R, Staaks J, Verhoeff A, de Boer A. Informal caregivers' views on the division of responsibilities between themselves and professionals: A scoping review. Health Soc Care Community. 2018;26(4):e460-e73.

846. Wolff RF, Reid K, di Nisio M, Aune D, Truyers C, Hernandez AV, et al. Systematic review of adverse events of buprenorphine patch versus fentanyl patch in patients with chronic moderate-to-severe pain. Pain Manag. 2012;2(4):351-62.

847. Wood E, Ohlsen S, Ricketts T. What are the barriers and facilitators to implementing Collaborative Care for depression? A systematic review. J Affect Disord. 2017;214:26-43.

848. Wood L, Alsawy S. Recovery in Psychosis from a Service User Perspective: A Systematic Review and Thematic Synthesis of Current Qualitative Evidence. Community Ment Health J. 2018;54(6):793-804.

849. Wood L, Burke E, Byrne R, Pyle M, Chapman N, Morrison A. Stigma in psychosis: A thematic synthesis of current qualitative evidence. Psychosis. 2015;7(2):152-65.

850. Wood L, Burke E, Morrison A. Individual cognitive behavioural therapy for psychosis (CBTp): a systematic review of qualitative literature. Behav Cogn Psychother. 2015;43(3):285-97.

851. Woodman C, Baillie J, Sivell S. The preferences and perspectives of family caregivers towards place of care for their relatives at the end-of-life. A systematic review and thematic synthesis of the qualitative evidence. BMJ Support Palliat Care. 2016;6(4):418-29.

852. Woods P, Schindel TJ, King MA, Mey A. Pharmacy practice in the domain of assisted dying: A mapping review of the literature. Research in Social and Administrative Pharmacy. 2019.

853. Wray F, Clarke D. Longer-term needs of stroke survivors with communication difficulties living in the community: a systematic review and thematic synthesis of qualitative studies. BMJ Open. 2017;7(10):e017944.

854. Wright DK, Gastmans C, Vandyk A, de Casterlé BD. Moral identity and palliative sedation: A systematic review of normative nursing literature. Nurs Ethics. 2020;27(3):868-86.

855. Wu MB, Levitt HM. A Qualitative Meta-analytic Review of the Therapist Responsiveness Literature: Guidelines for Practice and Training. Journal of Contemporary Psychotherapy. 2020;50(3):161-75.

856. Yaghobian M, Farhan R, Navipour H, Vanaki Z. Competencies of charge nurses: a systematic review and thematic synthesis. J Pak Med Assoc. 2020;70(7):1225-31.

857. Yao J, Tang H, Gao XL, McGrath C, Mattheos N. Patients' expectations to dental implant: a systematic review of the literature. Health Qual Life Outcomes. 2014;12:153.

858. Yim SH, Schmidt U. Experiences of computer-based and conventional self-help interventions for eating disorders: A systematic review and meta-synthesis of qualitative research. Int J Eat Disord. 2019;52(10):1108-24.

859. Yin M, Li Z, Zhou C. Experience of stigma among family members of people with severe mental illness: A qualitative systematic review. Int J Ment Health Nurs. 2020;29(2):141-60.

860. Yong FR, Garcia-Cardenas V, Williams KA, Benrimoj SI. Factors affecting community pharmacist work: A scoping review and thematic synthesis using role theory. Research in Social and Administrative Pharmacy. 2020;16(2):123-41.

861. Yoshikawa K, Brady B, Perry MA, Devan H. Sociocultural factors influencing physiotherapy management in culturally and linguistically diverse people with persistent pain: a scoping review. Physiotherapy (United Kingdom). 2020;107:292-305.

862. Young A. HPV vaccine acceptance among women in the Asian Pacific: a systematic review of the literature. Asian Pac J Cancer Prev. 2010;11(3):641-9.

863. Young K, Fisher J, Kirkman M. Women's experiences of endometriosis: a systematic review and synthesis of qualitative research. J Fam Plann Reprod Health Care. 2015;41(3):225-34.

864. Yuill C, McCourt C, Cheyne H, Leister N. Women's experiences of decision-making and informed choice about pregnancy and birth care: a systematic review and meta-synthesis of qualitative research. BMC Pregnancy Childbirth. 2020;20(1):343.

865. Zhou W, Yu Y, Yang M, Chen L, Xiao S. Policy development and challenges of global mental health: a systematic review of published studies of national-level mental health policies. BMC Psychiatry. 2018;18(1):138.

866. Zhu MN, Sari A, Lee MM. A systematic review of research methods and topics of the empirical MOOC literature (2014-2016). Internet High Educ. 2018;37:31-9.

867. Zhu MN, Sari AR, Lee MM. A comprehensive systematic review of MOOC research: Research techniques, topics, and trends from 2009 to 2019. ETR&D-Educ Tech Res Dev. 2020;68(4):1685-710.

868. Ziakova K, Cap J, Miertova M, Gurkova E. Dimensions of personal dignity of patients with multiple sclerosis: A qualitative narrative review. Central European Journal of Nursing and Midwifery. 2019;10(1):993-1004.

869. Zimmermann S, Forstmeier S. From fragments to identity: reminiscence, life review and well-being of holocaust survivors. An integrative review. Aging & mental health. 2020;24(4):525-49.

870. Zitomer MR, Goodwin D. Gauging the quality of qualitative research in adapted physical activity. Adapt Phys Activ Q. 2014;31(3):193-218.

871. Zwakman M, Jabbarian LJ, van Delden J, van der Heide A, Korfage IJ, Pollock K, et al. Advance care planning: A systematic review about experiences of patients with a life-threatening or life-limiting illness. Palliat Med. 2018;32(8):1305-21.

872. DePape AM, Lindsay S. Lived Experiences from the Perspective of Individuals with Autism Spectrum Disorder: A Qualitative Meta-Synthesis. Focus on Autism and Other Developmental Disabilities. 2016;31(1):60-71.

873. Derges J, Kidger J, Fox F, Campbell R, Kaner E, Hickman M. Alcohol screening and brief interventions for adults and young people in health and community-based settings: a qualitative systematic literature review. BMC Public Health. 2017;17(1):562.

874. Dheensa S, Metcalfe A, Williams RA. Men's experiences of antenatal screening: a metasynthesis of the qualitative research. Int J Nurs Stud. 2013;50(1):121-33.

875. Dickson K, Melendez-Torres GJ, Fletcher A, Hinds K, Thomas J, Stansfield C, et al. How Do Contextual Factors Influence Implementation and Receipt of Positive Youth Development Programs Addressing Substance Use and Violence? A Qualitative Meta-Synthesis of Process Evaluations. Am J Health Promot. 2018;32(4):1110-21.

876. Didier A, Dzemaili S, Perrenoud B, Campbell J, Gachoud D, Serex M, et al. Patients' perspectives on interprofessional collaboration between health care professionals during hospitalization: a qualitative systematic review. JBI Evid Synth. 2020;18(6):1208-70.

877. DiFusco LA, Schell KA, Saylor JL. Risk-taking behaviors in adolescents with chronic cardiac conditions: A scoping review. Journal of Pediatric Nursing. 2019;48:98-105.

878. Dillane I, Doody O. Nursing people with intellectual disability and dementia experiencing pain: An integrative review. J Clin Nurs. 2019;28(13-14):2472-85.

879. Disler RT, Green A, Luckett T, Newton PJ, Inglis S, Currow DC, et al. Experience of advanced chronic obstructive pulmonary disease: Metasynthesis of qualitative research. Journal of Pain and Symptom Management. 2014;48(6):1182-99.

880. Dixon-Woods M, Sutton A, Shaw R, Miller T, Smith J, Young B, et al. Appraising qualitative research for inclusion in systematic reviews: a quantitative and qualitative comparison of three methods. J Health Serv Res Policy. 2007;12(1):42-7.

881. Djiadeu P, Yusuf A, Ongolo-Zogo C, Nguemo J, Odhiambo AJ, Mukandoli C, et al. Barriers in accessing HIV care for Francophone African, Caribbean and Black people living with HIV in Canada: a scoping review. BMJ Open. 2020;10(8):e036885.

882. Dolman C, Jones I, Howard LM. Pre-conception to parenting: a systematic review and meta-synthesis of the qualitative literature on motherhood for women with severe mental illness. Arch Womens Ment Health. 2013;16(3):173-96.

883. Domeij H, Fahlström G, Bertilsson G, Hultcrantz M, Munthe-Kaas H, Gordh CN, et al. Experiences of living with fetal alcohol spectrum disorders: a systematic review and synthesis of qualitative data. Dev Med Child Neurol. 2018;60(8):741-52.

884. Doran C, Noonan M, Doody O. Life-story work in long-term care facilities for older people: An integrative review. J Clin Nurs. 2019;28(7-8):1070-84.

885. Dore-Smith E, Killingback C. What are the postoperative experiences of patients who have undergone hip and knee joint replacement? A literature review. Physical Therapy Reviews. 2018;23(4-5):250-8.

886. Doroud N, Fossey E, Fortune T. Place for being, doing, becoming and belonging: A meta-synthesis exploring the role of place in mental health recovery. Health Place. 2018;52:110-20.

887. Dowlati M, Seyedin H, Moslehi S. Hospital Preparedness Measures for Biological Hazards: A Systematic Review and Meta-Synthesis. Disaster Med Public Health Prep. 2020:1-14.

888. Dowlen R, Keady J, Milligan C, Swarbrick C, Ponsillo N, Geddes L, et al. The personal benefits of musicking for people living with dementia: a thematic synthesis of the qualitative literature. Arts Health. 2018;10(3):197-212.

889. Downe S, Finlayson K, Tunçalp Ö, Gülmezoglu AM. Provision and uptake of routine antenatal services: a qualitative evidence synthesis. Cochrane Database Syst Rev. 2019;6(6):Cd012392.

890. Drew K, Morris R, Tod D, Eubank M. A meta-study of qualitative research on the junior-to-senior transition in sport. Psychology of Sport and Exercise. 2019;45:20.

891. Driesen L, Patton R, John M. The impact of multiple chemical sensitivity on people's social and occupational functioning; a systematic review of qualitative research studies. J Psychosom Res. 2020;132:109964.

892. Due-Christensen M, Zoffmann V, Willaing I, Hopkins D, Forbes A. The Process of Adaptation Following a New Diagnosis of Type 1 Diabetes in Adulthood: A Meta-Synthesis. Qual Health Res. 2018;28(2):245-58.

893. Dutta O, Tan-Ho G, Choo PY, Ho AHY. Lived experience of a child's chronic illness and death: A qualitative systematic review of the parental bereavement trajectory. Death Stud. 2019;43(9):547-61.

894. Dwyer D. Experiences of registered nurses as managers and leaders in residential aged care facilities: a systematic review. Int J Evid Based Healthc. 2011;9(4):388-402.

895. Dyer E, Bell R, Graham R, Rankin J. Pregnancy decisions after fetal or perinatal death: systematic review of qualitative research. BMJ Open. 2019;9(12):e029930.

896. Dyer K, das Nair R. Why don't healthcare professionals talk about sex? A systematic review of recent qualitative studies conducted in the United kingdom. J Sex Med. 2013;10(11):2658-70.

897. Eakle R, Bourne A, Jarrett C, Stadler J, Larson H. Motivations and barriers to uptake and use of female-initiated, biomedical HIV prevention products in sub-Saharan Africa: an adapted meta-ethnography. BMC Public Health. 2017;17(1):968.

898. Earle S, Hadley R. Men's views and experiences of infant feeding: A qualitative systematic review. Matern Child Nutr. 2018;14(3):e12586.

899. Earle S, O'Dell L, Davies A, Rixon A. Views and Experiences of Sex, Sexuality and Relationships Following Spinal Cord Injury: A Systematic Review and Narrative Synthesis of the Qualitative Literature. Sexuality and Disability. 2020.

900. Eassey D, Reddel HK, Foster JM, Kirkpatrick S, Locock L, Ryan K, et al. "…I've said I wish I was dead, you'd be better off without me": A systematic review of people's experiences of living with severe asthma. J Asthma. 2019;56(3):311-22.

901. Eassey D, Reddel HK, Ryan K, Smith L. 'It is like learning how to live all over again' A systematic review of people's experiences of living with a chronic illness from a self-determination theory perspective. Health Psychology and Behavioral Medicine. 2020;8(1):270-91.

902. Eassom E, Giacco D, Dirik A, Priebe S. Implementing family involvement in the treatment of patients with psychosis: a systematic review of facilitating and hindering factors. BMJ Open. 2014;4(10):e006108.

903. Eccleston L, Williams J, Knowles S, Soulsby L. Adolescent experiences of living with a diagnosis of ADHD: a systematic review and thematic synthesis. Emotional and Behavioural Difficulties. 2019;24(2):119-35.

904. Eddy K, Jordan Z, Stephenson M. Health professionals' experience of teamwork education in acute hospital settings: a systematic review of qualitative literature. JBI Database System Rev Implement Rep. 2016;14(4):96-137.

905. Edwards A, Pang N, Shiu V, Chan C. Review: The understanding of spirituality and the potential role of spiritual care in end-of-life and palliative care: A meta-study of qualitative research. Palliative Medicine. 2010;24(8):753-70.

906. Edwards DJ, Sakellariou D, Anstey S. Barriers to, and facilitators of, access to cancer services and experiences of cancer care for adults with a physical disability: A mixed methods systematic review. Disabil Health J. 2020;13(1):100844.

907. Egilstrod B, Ravn MB, Petersen KS. Living with a partner with dementia: a systematic review and thematic synthesis of spouses' lived experiences of changes in their everyday lives. Aging Ment Health. 2019;23(5):541-50.

908. Elamin W, Mason-Jones AJ. Female Genital Mutilation/Cutting: A Systematic Review and Meta-Ethnography Exploring Women's Views of Why It Exists and Persists. International Journal of Sexual Health. 2020;32(1):1-21.

909. Elmore J, Wright DK, Paradis M. Nurses' moral experiences of assisted death: A meta-synthesis of qualitative research. Nursing Ethics. 2018;25(8):955-72.

910. Engbers RA. Students' perceptions of interventions designed to foster empathy: An integrative review. Nurse Education Today. 2020;86 (no pagination).

911. Ennals P, Fossey E, Howie L. Postsecondary study and mental ill-health: A meta-synthesis of qualitative research exploring students' lived experiences. Journal of Mental Health. 2015;24(2):111-9.

912. Erku DA, Gartner CE, Morphett K, Steadman KJ. Beliefs and Self-reported Practices of Health Care Professionals Regarding Electronic Nicotine Delivery Systems: A Mixed-Methods Systematic Review and Synthesis. Nicotine Tob Res. 2020;22(5):619-29.

913. Etkind SN, Bone AE, Lovell N, Higginson IJ, Murtagh FEM. Influences on Care Preferences of Older People with Advanced Illness: A Systematic Review and Thematic Synthesis. J Am Geriatr Soc. 2018;66(5):1031-9.

914. Etxeandia-Ikobaltzeta I, Zhang Y, Brundisini F, Florez ID, Wiercioch W, Nieuwlaat R, et al. Patient values and preferences regarding VTE disease: a systematic review to inform American Society of Hematology guidelines. Blood Adv. 2020;4(5):953-68.

915. Evans C, Nalubega S, McLuskey J, Darlington N, Croston M, Bath-Hextall F. The views and experiences of nurses and midwives in the provision and management of provider-initiated HIV testing and counseling: a systematic review of qualitative evidence. JBI Database System Rev Implement Rep. 2016;13(12):130-286.

916. Evans D, Fitzgerald M. The experience of physical restraint: a systematic review of qualitative research. Contemp Nurse. 2002;13(2-3):126-35.

917. Evans D, Lee E. Impact of dementia on marriage: a qualitative systematic review. Dementia (London). 2014;13(3):330-49.

918. Evans L, Randle-Phillips C. People with intellectual disabilities' experiences of psychological therapy: A systematic review and meta-ethnography. J Intellect Disabil. 2020;24(2):233-52.

919. Evans M, Bryant S, Huntley AL, Feder G. Cancer Patients' Experiences of Using Mistletoe (Viscum album): A Qualitative Systematic Review and Synthesis. J Altern Complement Med. 2016;22(2):134-44.

920. Evans R, Hurrell C. The role of schools in children and young people's self-harm and suicide: systematic review and meta-ethnography of qualitative research. BMC Public Health. 2016;16:401.

921. Fair F, Raben L, Watson H, Vivilaki V, van den Muijsenbergh M, Soltani H. Migrant women's experiences of pregnancy, childbirth and maternity care in European countries: A systematic review. PLoS One. 2020;15(2):e0228378.

922. Falahee M, Simons G, Raza K, Stack RJ. Healthcare professionals' perceptions of risk in the context of genetic testing for the prediction of chronic disease: a qualitative metasynthesis. J Risk Res. 2018;21(2):129-66.

923. Fayette R, Bond C. A systematic literature review of qualitative research methods for eliciting the views of young people with ASD about their educational experiences. European Journal of Special Needs Education. 2018;33(3):349-65.

924. Fenech G, Thomson G. Tormented by ghosts from their past': a meta-synthesis to explore the psychosocial implications of a traumatic birth on maternal well-being. Midwifery. 2014;30(2):185-93.

925. Fernández-Basanta S, Coronado C, Movilla-Fernández MJ. Multicultural coping experiences of parents following perinatal loss: A meta-ethnographic synthesis. J Adv Nurs. 2020;76(1):9-21.

926. Ferreira SMB, Yonekura T, Ignotti E, Oliveira LB, Takahashi J, Soares CB. Effectiveness of rifampicin chemoprophylaxis in preventing leprosy in patient contacts: a systematic review of quantitative and qualitative evidence. JBI Database System Rev Implement Rep. 2017;15(10):2555-84.

927. Fielden HG, Brown SL, Saini P, Beesley H, Salmon P. How do women at increased breast cancer risk perceive and decide between risks of cancer and risk-reducing treatments? A synthesis of qualitative research. Psycho-Oncology. 2017;26(9):1254-62.

928. Finfgeld-Connett D, Johnson ED. Substance abuse treatment for women who are under correctional supervision in the community: a systematic review of qualitative findings. Issues Ment Health Nurs. 2011;32(10):640-8.

929. Finlayson K, Crossland N, Bonet M, Downe S. What matters to women in the postnatal period: A meta-synthesis of qualitative studies. PLoS One. 2020;15(4):e0231415.

930. Fioretti C, Mazzocco K, Riva S, Oliveri S, Masiero M, Pravettoni G. Research studies on patients' illness experience using the Narrative Medicine approach: a systematic review. BMJ Open. 2016;6(7):e011220.

931. Fisher H, Harding S, Hickman M, Macleod J, Audrey S. Barriers and enablers to adolescent self-consent for vaccination: A mixed-methods evidence synthesis. Vaccine. 2019;37(3):417-29.

932. Flemming K. Synthesis of quantitative and qualitative research: an example using Critical Interpretive Synthesis. J Adv Nurs. 2010;66(1):201-17.

933. Flemming K. The use of morphine to treat cancer-related pain: a synthesis of quantitative and qualitative research. J Pain Symptom Manage. 2010;39(1):139-54.

934. Flemming K, Graham H, Heirs M, Fox D, Sowden A. Smoking in pregnancy: a systematic review of qualitative research of women who commence pregnancy as smokers. J Adv Nurs. 2013;69(5):1023-36.

935. Flynn AP, Carter B, Bray L, Donne AJ. Parents' experiences and views of caring for a child with a tracheostomy: A literature review. International Journal of Pediatric Otorhinolaryngology. 2013;77(10):1630-4.

936. Forster AS, Rockliffe L, Chorley AJ, Marlow LAV, Bedford H, Smith SG, et al. Ethnicity-specific factors influencing childhood immunisation decisions among Black and Asian Minority Ethnic groups in the UK: a systematic review of qualitative research. J Epidemiol Community Health. 2017;71(6):544-9.

937. Foster MJ, Whitehead L, Maybee P, Cullens V. The parents', hospitalized child's, and health care providers' perceptions and experiences of family centered care within a pediatric critical care setting: a metasynthesis of qualitative research. J Fam Nurs. 2013;19(4):431-68.

938. Francia L, Millear P, Sharman R. Mothers and fathers' experiences of high conflict past two years post separation: A systematic review of the qualitative literature. J Child Custody. 2019;16(2):170-96.

939. Frankis JS, Flowers P. Public sexual cultures: a systematic review of qualitative research investigating men's sexual behaviors with men in public spaces. J Homosex. 2009;56(7):861-93.

940. Franklin M, Lewis S, Willis K, Bourke-Taylor H, Smith L. Patients' and healthcare professionals' perceptions of self-management support interactions: Systematic review and qualitative synthesis. Chronic Illn. 2018;14(2):79-103.

941. Franzen SR, Chandler C, Lang T. Health research capacity development in low and middle income countries: reality or rhetoric? A systematic meta-narrative review of the qualitative literature. BMJ Open. 2017;7(1):e012332.

942. Friedman JA. Experiences of left ventricular assist device-destination therapy recipients: A systematic review and meta-synthesis. Heart Lung. 2020;49(5):463-74.

943. Frost R, Beattie A, Bhanu C, Walters K, Ben-Shlomo Y. Management of depression and referral of older people to psychological therapies: a systematic review of qualitative studies. Br J Gen Pract. 2019;69(680):e171-e81.

944. Fryer C, Mackintosh S, Stanley M, Crichton J. Qualitative studies using in-depth interviews with older people from multiple language groups: methodological systematic review. J Adv Nurs. 2012;68(1):22-35.

945. Fulford H, McSwiggan L, Kroll T, MacGillivray S. Exploring the Use of Information and Communication Technology by People With Mood Disorder: A Systematic Review and Metasynthesis. JMIR Ment Health. 2016;3(3):e30.

946. Galdas P, Darwin Z, Kidd L, Blickem C, McPherson K, Hunt K, et al. The accessibility and acceptability of self-management support interventions for men with long term conditions: a systematic review and meta-synthesis of qualitative studies. BMC Public Health. 2014;14:1230.

947. Gallacher K, Morrison D, Jani B, Macdonald S, May CR, Montori VM, et al. Uncovering treatment burden as a key concept for stroke care: a systematic review of qualitative research. PLoS Med. 2013;10(6):e1001473.

948. Gallacher KI, Quinn T, Kidd L, Eton D, Dillon M, Elliot J, et al. Systematic review of patient-reported measures of treatment burden in stroke. BMJ Open. 2019;9(9):e029258.

949. Gamondi C, Fusi-Schmidhauser T, Oriani A, Payne S, Preston N. Family members' experiences of assisted dying: A systematic literature review with thematic synthesis. Palliat Med. 2019;33(8):1091-105.

950. Garcia-Rueda N, Carvajal Valcarcel A, Saracibar-Razquin M, Arantzamendi Solabarrieta M. The experience of living with advanced-stage cancer: a thematic synthesis of the literature. European Journal of Cancer Care. 2016;25(4):551-69.

951. Garip G, Yardley L. A synthesis of qualitative research on overweight and obese people's views and experiences of weight management. Clinical Obesity. 2011;1(2-3):110-26.

952. Garside R, Britten N, Stein K. The experience of heavy menstrual bleeding: a systematic review and meta-ethnography of qualitative studies. J Adv Nurs. 2008;63(6):550-62.

953. Garside R, Pearson M, Moxham T. What influences the uptake of information to prevent skin cancer? A systematic review and synthesis of qualitative research. Health Educ Res. 2010;25(1):162-82.

954. Gdanetz LM, Hamer MK, Thomas E, Tarasenko LM, Horton-Deutsch S, Jones J. Technology, educator intention, and relationships in virtual learning spaces: A qualitative metasynthesis. Journal of Nursing Education. 2018;57(4):197-202.

955. Gee B, Wilson J, Clarke T, Farthing S, Carroll B, Jackson C, et al. Review: Delivering mental health support within schools and colleges - a thematic synthesis of barriers and facilitators to implementation of indicated psychological interventions for adolescents. Child and Adolescent Mental Health. 2020.

956. Gentry S, Craig J, Holland R, Notley C. Smoking cessation for substance misusers: A systematic review of qualitative studies on participant and provider beliefs and perceptions. Drug Alcohol Depend. 2017;180:178-92.

957. Gentry S, Forouhi NG, Notley C. Are Electronic Cigarettes an Effective Aid to Smoking Cessation or Reduction Among Vulnerable Groups? A Systematic Review of Quantitative and Qualitative Evidence. Nicotine Tob Res. 2019;21(5):602-16.

958. Gewirtz-Meydan A, Hafford-Letchfield T, Ayalon L, Benyamini Y, Biermann V, Coffey A, et al. How do older people discuss their own sexuality? A systematic review of qualitative research studies. Cult Health Sex. 2019;21(3):293-308.

959. Ghiasi A. Health information needs, sources of information, and barriers to accessing health information among pregnant women: a systematic review of research. J Matern Fetal Neonatal Med. 2019:1-11.

960. Giacomini M, DeJean D, Simeonov D, Smith A. Experiences of living and dying with COPD: a systematic review and synthesis of the qualitative empirical literature. Ont Health Technol Assess Ser. 2012;12(13):1-47.

961. Gibbons C, Singh S, Gibbons B, Clark C, Torres J, Cheng MY, et al. Using qualitative methods to understand factors contributing to patient satisfaction among dermatology patients: a systematic review. J Dermatolog Treat. 2018;29(3):290-4.

962. Gibbor L, Yates L, Volkmer A, Spector A. Cognitive stimulation therapy (CST) for dementia: a systematic review of qualitative research. Aging Ment Health. 2020:1-11.

963. Giles TM, Hall KL. Qualitative systematic review: the unique experiences of the nurse-family member when a loved one is admitted with a critical illness. J Adv Nurs. 2014;70(7):1451-64.

964. Glassman HS, Rhodes P, Buus N. A Critical Review of Qualitative Interview Studies with Alcoholics Anonymous Members. Subst Use Misuse. 2020;55(3):387-98.

965. Athanasakis E. A meta-synthesis of how registered nurses make sense of their lived experiences of medication errors. Journal of Clinical Nursing. 2019;28(17-18):3077-95.

966. Betz ME, Scott K, Jones J, Diguiseppi C. "Are you still driving?" Metasynthesis of patient preferences for communication with health care providers. Traffic Inj Prev. 2016;17(4):367-73.

967. Bootsma TI, Schellekens MPJ, van Woezik RAM, van der Lee ML, Slatman J. Experiencing and responding to chronic cancer-related fatigue: A meta-ethnography of qualitative research. Psychooncology. 2020;29(2):241-50.

968. Bradbury-Jones C, Breckenridge J, Clark MT, Herber OR, Wagstaff C, Taylor J. The state of qualitative research in health and social science literature: a focused mapping review and synthesis. International Journal of Social Research Methodology. 2017;20(6):627-45.

969. Brown B, Gude WT, Blakeman T, van der Veer SN, Ivers N, Francis JJ, et al. Clinical Performance Feedback Intervention Theory (CP-FIT): a new theory for designing, implementing, and evaluating feedback in health care based on a systematic review and meta-synthesis of qualitative research. Implement Sci. 2019;14(1):40.

970. Carolan CM, Smith A, Davies GR, Forbat L. Seeking, accepting and declining help for emotional distress in cancer: A systematic review and thematic synthesis of qualitative evidence. European Journal of Cancer Care. 2018;27(2).

971. Chatfield SL, DeBois K, Nolan R, Crawford H, Hallam JS. Hand hygiene among healthcare workers: A qualitative meta summary using the GRADE-CERQual process. J Infect Prev. 2017;18(3):104-20.

972. Fletcher C, Flight I, Chapman J, Fennell K, Wilson C. The information needs of adult cancer survivors across the cancer continuum: A scoping review. Patient Education and Counseling. 2017;100(3):383-410.

973. Fox L, Wiseman T, Cahill D, Beyer K, Peat N, Rammant E, et al. Barriers and facilitators to physical activity in men with prostate cancer: A qualitative and quantitative systematic review. Psycho-Oncology.

974. Habib A, Stevelink SAM, Greenberg N, Williamson V. Post-traumatic growth in (ex-) military personnel: review and qualitative synthesis. Occupational Medicine-Oxford. 2018;68(9):617-25.

975. Harwood L, Clark AM. Understanding pre-dialysis modality decision-making: A meta-synthesis of qualitative studies. International Journal of Nursing Studies. 2013;50(1):109-20.

976. Junger S, Payne SA, Brine J, Radbruch L, Brearley SG. Guidance on Conducting and REporting DElphi Studies (CREDES) in palliative care: Recommendations based on a methodological systematic review. Palliative Medicine. 2017;31(8):684-706.

977. Katz IT, Ryu AE, Onuegbu AG, Psaros C, Weiser SD, Bangsberg DR, et al. Impact of HIV-related stigma on treatment adherence: systematic review and meta-synthesis. J Int AIDS Soc. 2013;16(3 Suppl 2):18640.

978. Kebbe M, Damanhoury S, Browne N, Dyson MP, McHugh TLF, Ball GDC. Barriers to and enablers of healthy lifestyle behaviours in adolescents with obesity: a scoping review and stakeholder consultation. Obesity Reviews. 2017;18(12):1439-53.

979. Kien C, Nußbaumer B, Thaler KJ, Griebler U, Van Noord MG, Wagner P, et al. Barriers to and facilitators of interventions to counter publication bias: thematic analysis of scholarly articles and stakeholder interviews. BMC Health Serv Res. 2014;14:551.

980. Licqurish S, Phillipson L, Chiang P, Walker J, Walter F, Emery J. Cancer beliefs in ethnic minority populations: a review and meta-synthesis of qualitative studies. European Journal of Cancer Care. 2017;26(1).

981. Marshall S, Grinyer A, Limmer M. The Experience of Adolescents and Young Adults Treated for Cancer in an Adult Setting: A Review of the Literature. Journal of Adolescent and Young Adult Oncology. 2018;7(3):283-91.

982. McDermott E, Selman LE. Cultural Factors Influencing Advance Care Planning in Progressive, Incurable Disease: A Systematic Review With Narrative Synthesis. Journal of Pain and Symptom Management. 2018;56(4):613-36.

983. McHugh TLF, Deal CJ, Blye CJ, Dimler AJ, Halpenny EA, Sivak A, et al. A Meta-Study of Qualitative Research Examining Sport and Recreation Experiences of Indigenous Youth. Qualitative Health Research. 2019;29(1):42-54.

984. Merry L, Pelaez S, Edwards NC. Refugees, asylum-seekers and undocumented migrants and the experience of parenthood: a synthesis of the qualitative literature. Global Health. 2017;13(1):75.

985. Mhatre SK, Sansgiry SS. Development of a conceptual model of health-related quality of life among hepatitis C patients: A systematic review of qualitative studies. Hepatology Research. 2016;46(1):29-39.

986. Ndumele CD, Ableman G, Russell BE, Gurrola E, Hicks LS. Publication of Recruitment Methods in Focus Group Research of Minority Populations with Chronic Disease: A Systematic Review. Journal of Health Care for the Poor and Underserved. 2011;22(1):5-23.

987. Nicolopoulos A, Boydell K, Shand F, Christensen H. Why Suicide? Reasons for Suicide Attempts as Self- Reported by Youth: A Systematic Evaluation of Qualitative Studies. Adolescent Research Review. 2018;3(2):155-72.

988. Noyes J, Hendry M, Lewin S, Glenton C, Chandler J, Rashidian A. Qualitative "trial-sibling" studies and "unrelated" qualitative studies contributed to complex intervention reviews. Journal of Clinical Epidemiology. 2016;74:133-43.

989. Ottrey E, Jong J, Porter J. Ethnography in Nutrition and Dietetics Research: A Systematic Review. Journal of the Academy of Nutrition and Dietetics. 2018;118(10):1903-+.

990. Papanastasiou N, Hill S, Amos A. Evidence From Qualitative Studies of Youth About the Impacts of Tobacco Control Policy on Young People in Europe: A Systematic Review. Nicotine & Tobacco Research. 2019;21(7):863-70.

991. Peeters G, Barker AL, Talevski J, Ackerman I, Ayton DR, Reid C, et al. Do patients have a say? A narrative review of the development of patient-reported outcome measures used in elective procedures for coronary revascularisation. Qual Life Res. 2018;27(5):1369-80.

992. Penny RA, Bradford NK, Langbecker D. Registered nurse and midwife experiences of using videoconferencing in practice: A systematic review of qualitative studies. Journal of Clinical Nursing. 2018;27(5-6):E739-E52.

993. Pinnock H, Epiphaniou E, Sheikh A, Griffiths C, Eldridge S, Craig P, et al. Developing standards for reporting implementation studies of complex interventions (StaRI): a systematic review and e-Delphi. Implement Sci. 2015;10:42.

994. Pocock T, Miyahara M. Inclusion of students with disability in physical education: a qualitative meta-analysis. International Journal of Inclusive Education. 2018;22(7):751-66.

995. Raskind IG, Shelton RC, Comeau DL, Cooper HLF, Griffith DM, Kegler MC. A Review of Qualitative Data Analysis Practices in Health Education and Health Behavior Research. Health Educ Behav. 2019;46(1):32-9.

996. Sariman JA, Harris NM, Harvey D, Sansom-Daly UM. Experiences of Young People Living with Cancer in Nonmetropolitan Areas: A Review of the Literature. Journal of Adolescent and Young Adult Oncology. 2020;9(2):133-44.

997. Slade SC, Patel S, Underwood M, Keating JL. What Are Patient Beliefs and Perceptions About Exercise for Nonspecific Chronic Low Back Pain? A Systematic Review of Qualitative Studies. Clinical Journal of Pain. 2014;30(11):995-1005.

998. SmithBattle L, Lorenz R, Reangsing C, Palmer JL, Pitroff G. A methodological review of qualitative longitudinal research in nursing. Nursing Inquiry. 2018;25(4).

999. Snijder M, Shakeshaft A, Wagemakers A, Stephens A, Calabria B. A systematic review of studies evaluating Australian indigenous community development projects: the extent of community participation, their methodological quality and their outcomes. BMC Public Health. 2015;15:1154.

1000. Stuckey H, Peyrot M. Living with diabetes: literature review and secondary analysis of qualitative data. Diabetic Medicine. 2020;37(3):493-503.

1001. Taylor J, Bradbury-Jones C, Breckenridge JP, Jones C, Herber OR. Risk of vicarious trauma in nursing research: a focused mapping review and synthesis. Journal of Clinical Nursing. 2016;25(19-20):2768-77.

1002. Vogel JA, Rising KL, Jones J, Bowden ML, Ginde AA, Havranek EP. Reasons Patients Choose the Emergency Department over Primary Care: a Qualitative Metasynthesis. Journal of General Internal Medicine. 2019;34(11):2610-9.

1003. Wells MB, Lang SN. Supporting same-sex mothers in the Nordic child health field: a systematic literature review and meta-synthesis of the most gender equal countries. Journal of Clinical Nursing. 2016;25(23-24):3469-83.

1004. Yamamoto R, Keogh B. Children's experiences of living with a parent with mental illness: A systematic review of qualitative studies using thematic analysis. Journal of Psychiatric and Mental Health Nursing. 2018;25(2):131-41.

1005. Zorbas C, Palermo C, Chung A, Iguacel I, Peeters A, Bennett R, et al. Factors perceived to influence healthy eating: a systematic review and meta-ethnographic synthesis of the literature. Nutrition Reviews. 2018;76(12):861-74.

1006. Cruz-Martínez RR, Wentzel J, Asbjørnsen RA, Noort PD, van Niekerk JM, Sanderman R, et al. Supporting Self-Management of Cardiovascular Diseases Through Remote Monitoring Technologies: Metaethnography Review of Frameworks, Models, and Theories Used in Research and Development. J Med Internet Res. 2020;22(5):e16157.

1007. van Leeuwen KM, van Loon MS, van Nes FA, Bosmans JE, de Vet HCW, Ket JCF, et al. What does quality of life mean to older adults? A thematic synthesis. PLoS One. 2019;14(3):e0213263.

1008. Vseteckova J, Deepak-Gopinath M, Borgstrom E, Holland C, Draper J, Pappas Y, et al. Barriers and facilitators to adherence to group exercise in institutionalized older people living with dementia: a systematic review. Eur Rev Aging Phys Act. 2018;15:11.

1009. Miller MJ, Jones J, Anderson CB, Christiansen CL. Factors influencing participation in physical activity after dysvascular amputation: a qualitative meta-synthesis. Disabil Rehabil. 2019;41(26):3141-50.

1010. Simpson-Adkins GJ, Daiches A. How Do Children Make Sense of their Parent's Mental Health Difficulties: A Meta-Synthesis. J Child Fam Stud. 2018;27(9):2705-16.

1011. Pescheny JV, Pappas Y, Randhawa G. Facilitators and barriers of implementing and delivering social prescribing services: a systematic review. BMC Health Serv Res. 2018;18(1):86.

1012. Brooks HL, Rushton K, Lovell K, Bee P, Walker L, Grant L, et al. The power of support from companion animals for people living with mental health problems: a systematic review and narrative synthesis of the evidence. BMC Psychiatry. 2018;18(1):31.

1013. Wickremasinghe D, Hashmi IE, Schellenberg J, Avan BI. District decision-making for health in low-income settings: a systematic literature review. Health Policy Plan. 2016;31 Suppl 2(Suppl 2):ii12-ii24.

1014. Fudge N, Sadler E, Fisher HR, Maher J, Wolfe CD, McKevitt C. Optimising Translational Research Opportunities: A Systematic Review and Narrative Synthesis of Basic and Clinician Scientists' Perspectives of Factors Which Enable or Hinder Translational Research. PLoS One. 2016;11(8):e0160475.

1015. Mathers J, Rick C, Jenkinson C, Garside R, Pall H, Mitchell R, et al. Patients' experiences of deep brain stimulation for Parkinson's disease: a qualitative systematic review and synthesis. BMJ Open. 2016;6(6):e011525.

1016. Tso LS, Best J, Beanland R, Doherty M, Lackey M, Ma Q, et al. Facilitators and barriers in HIV linkage to care interventions: a qualitative evidence review. Aids. 2016;30(10):1639-53.

1017. Knowles SE, Toms G, Sanders C, Bee P, Lovell K, Rennick-Egglestone S, et al. Qualitative meta-synthesis of user experience of computerised therapy for depression and anxiety. PLoS One. 2014;9(1):e84323.

1018. Charitou M, Quayle E, Sutherland A. Supporting Adults with Intellectual Disabilities with Relationships and Sex: A Systematic Review and Thematic Synthesis of Qualitative Research with Staff. Sexuality and Disability.

1019. Sweeney L, Clarke C, Wolverson E. The use of everyday technologies to enhance well-being and enjoyment for people living with dementia: A systematic literature review and narrative synthesis. Dementia-International Journal of Social Research and Practice.

1020. Villa D, Causer H, Riley GA. Experiences that challenge self-identity following traumatic brain injury: a meta-synthesis of qualitative research. Disability and Rehabilitation.

1021. Meek C, Topcu G, Moghaddam N, das Nair R. Experiences of adjustment to secondary progressive multiple sclerosis: a meta-ethnographic systematic review. Disability and Rehabilitation.

1022. Rousseva C, Kammath V, Tancred T, Smith H. Health workers' views on audit in maternal and newborn healthcare in LMICs: a qualitative evidence synthesis. Tropical Medicine & International Health. 2020;25(5):525-39.

1023. Forde R, Peters S, Wittkowski A. Recovery from postpartum psychosis: a systematic review and metasynthesis of women's and families' experiences. Archives of Womens Mental Health.

1024. Tuomikoski AM, Ruotsalainen H, Mikkonen K, Kaariainen M. Nurses' experiences of their competence at mentoring nursing students during clinical practice: A systematic review of qualitative studies. Nurse Education Today. 2020;85.

1025. Raybin JL, Barr E, Krajicek M, Jones J. How Does Creative Arts Therapy Reduce Distress for Children With Cancer? A Metasynthesis of Extant Qualitative Literature. Journal of Pediatric Oncology Nursing. 2020;37(2):91-104.

1026. McGaw VE, Reupert AE, Maybery D. Military Posttraumatic Stress Disorder: A Qualitative Systematic Review of the Experience of Families, Parents and Children. Journal of Child and Family Studies. 2019;28(11):2942-52.

1027. Boyle S, Vseteckova J, Higgins M. Impact of Motivational Interviewing by Social Workers on Service Users: A Systematic Review. Research on Social Work Practice. 2019;29(8):863-75.

1028. Luchsinger JS, Jones J, McFarland AK, Kissler K. Examining nurse/patient relationships in care coordination: A qualitative metasynthesis. Applied Nursing Research. 2019;49:41-9.

1029. Ruffell B, Smith DM, Wittkowski A. The Experiences of Male Partners of Women with Postnatal Mental Health Problems: A Systematic Review and Thematic Synthesis. Journal of Child and Family Studies. 2019;28(10):2772-90.

1030. Turkmani S, Homer CSE, Dawson A. Maternity care experiences and health needs of migrant women from female genital mutilation-practicing countries in high-income contexts: A systematic review and meta-synthesis. Birth-Issues in Perinatal Care. 2019;46(1):3-14.

1031. Behzadifar M, Behzadifar M, Heidarvand S, Gorji HA, Aryankhesal A, Moghadam ST, et al. The challenges of the family physician policy in Iran: a systematic review and meta-synthesis of qualitative researches. Family Practice. 2018;35(6):652-60.

1032. Arai N, Takimoto Y, Nakazawa E, Akabayashi A. Considerations on the Factors Influencing Living Kidney Donors' Autonomous Decision-Making: A Systematic Literature Review. Transplantation Proceedings. 2018;50(10):3036-44.

1033. Harris M, Thomas G, Thomas M, Cafarella P, Stocks A, Greig J, et al. Supporting wellbeing in motor neurone disease for patients, carers, social networks, and health professionals: A scoping review and synthesis. Palliative & Supportive Care. 2018;16(2):228-37.

1034. Pitt E, Gallegos D, Comans T, Cameron C, Thornton L. Exploring the influence of local food environments on food behaviours: a systematic review of qualitative literature. Public Health Nutrition. 2017;20(13):2393-405.

1035. Toye F, Seers K, Hannink E, Barker K. A mega-ethnography of eleven qualitative evidence syntheses exploring the experience of living with chronic non-malignant pain. Bmc Medical Research Methodology. 2017;17.

1036. Leyva-Moral JM, Piscoya-Angeles PN, Edwards JE, Palmieri PA. The Experience of Pregnancy in Women Living With HIV: A Meta-Synthesis of Qualitative Evidence. Janac-Journal of the Association of Nurses in Aids Care. 2017;28(4):587-602.

1037. Willis C, Girdler S, Thompson M, Rosenberg M, Reid S, Elliott C. Elements contributing to meaningful participation for children and youth with disabilities: a scoping review. Disability and Rehabilitation. 2017;39(17):1771-84.

1038. Cambon B, Vorilhon P, Michel L, Cadwallader JS, Aubin-Auger I, Pereira B, et al. Quality of qualitative studies centred on patients in family practice: a systematic review. Family Practice. 2016;33(6):580-7.

1039. Davies S, Young B, Salmon P. Towards understanding problems in the parent-practitioner relationship when a child has cancer: meta-synthesis of the qualitative literature. Psycho-Oncology. 2016;25(11):1252-60.

1040. Mikkonen K, Elo S, Tuomikoski AM, Kaariainen M. Mentor experiences of international healthcare students' learning in a clinical environment: A systematic review. Nurse Education Today. 2016;40:87-94.

1041. Pozzebon M, Douglas J, Ames D. Spouses' experience of living with a partner diagnosed with a dementia: a synthesis of the qualitative research. International Psychogeriatrics. 2016;28(4):537-56.

1042. Toye F, Seers K, Allcock N, Briggs M, Carr E, Barker K. A synthesis of qualitative research exploring the barriers to staying in work with chronic musculoskeletal pain. Disability and Rehabilitation. 2016;38(6):566-72.
